# Electrochemical Hydrogenation of Aza-Arenes Using H_2_O as H Source

**DOI:** 10.1021/jacs.5c21117

**Published:** 2025-12-23

**Authors:** Subhabrata Dutta, Rok Narobe, Siegfried R. Waldvogel

**Affiliations:** † Max-Planck Institute for Chemical Energy Conversion, Department of Electrosynthesis, Stiftstraße 34−36, Mülheim an der Ruhr 45479, Germany; ‡ Karlsruhe Institute of Technology, Institute of Biological and Chemical Systems—Functional Molecular Systems (IBCS-FMS), Kaiserstraße 12, 76131 Karlsruhe, Germany

## Abstract

Electrochemical hydrogenation of aza-arenes is an appealing strategy to gain access to privileged saturated heterocycles for drug discovery, overcoming the limitations of classical hydrogenations that often suffer from energy-intensive conditions and safety hazards. Herein, we demonstrate an operationally simple, sustainable, and general electrochemical hydrogenation of aza-arenes with commercialized Ni foam electrodes and setup. With water as the hydrogen donor under acidic conditions, the reaction proceeds at ambient temperature and pressure to deliver broad substrate generality, excellent functional group tolerance, and excellent selectivity. The method tolerates a wide range of aza-arenes—including (iso)­quinolines, quinoxalines, pyridines, and their nium salts—highlighting its generality and robustness. Synthetic utility was showcased through the preparation of bioactive molecules, while scalability was achieved up to 25 g of product, highlighting the method’s technical applicability with stable 22 h operation without changes in the cell voltage or significant electrode degradation. Extensive mechanistic investigations using a combination of cyclic and RDE linear sweep voltammetry suggest two plausible routes based on the substrate’s redox properties: hydrogenation by chemisorbed hydrogen (*H*_ads_) or initial substrate reduction followed by *H*_ads_ transfer. This work sets a clean, practical, and versatile platform for aza-arene dearomatization, bridging academic interest with industrial targets in electrochemical hydrogenation.

## Graphical abstract

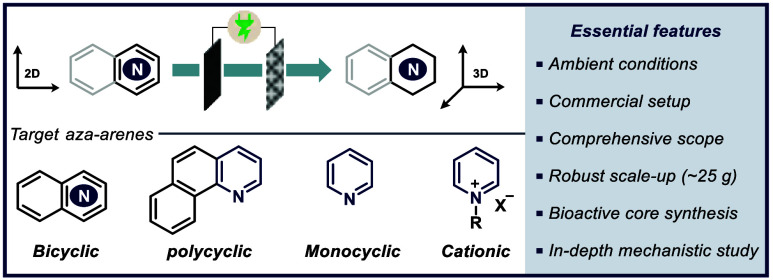

## Introduction

Saturation of heteroaromatic cores can significantly change the compound’s dynamics, reactivity, and physiological properties, creating new avenues in drug discovery and chemical biology. According to the past decade report, approximately 82% of the FDA small molecule approved drugs contained at least one N-containing ring (Scheme 1A,B).^1^ Within the subset of six-membered rings, ∼58% are saturated. All these statistics stem from the fact that saturation enhances lipophilicity, polarity, and kinetic solubility, often improving pharmacokinetic profiles and modulating biological activity.^2^ This is reflected in the recent surge of dearomatization reports, often quantified via Fsp^3^ values in the context of the “escape from flatland” concept.^3^ In this regard, a sustainable, efficient, and chemoselective synthesis of saturated aza rings from their aromatic parent core remains a high-value transformation in synthetic chemistry.^4^ Partial or complete hydrogenation is the most direct approach to hydrogenated derivatives.^5–7^ However, the ground-state aromatic stabilization of aza-arenes,^8^ combined with the presence of other reactive functionalities (e.g., halides, free amines, hydroxyl, carboxylic acid groups), makes their selective hydrogenation less robust and challenging. Uncontrolled reduction, dehalogenation, and undesired hydrogenolysis are common with conventional settings.

**Scheme 1:**
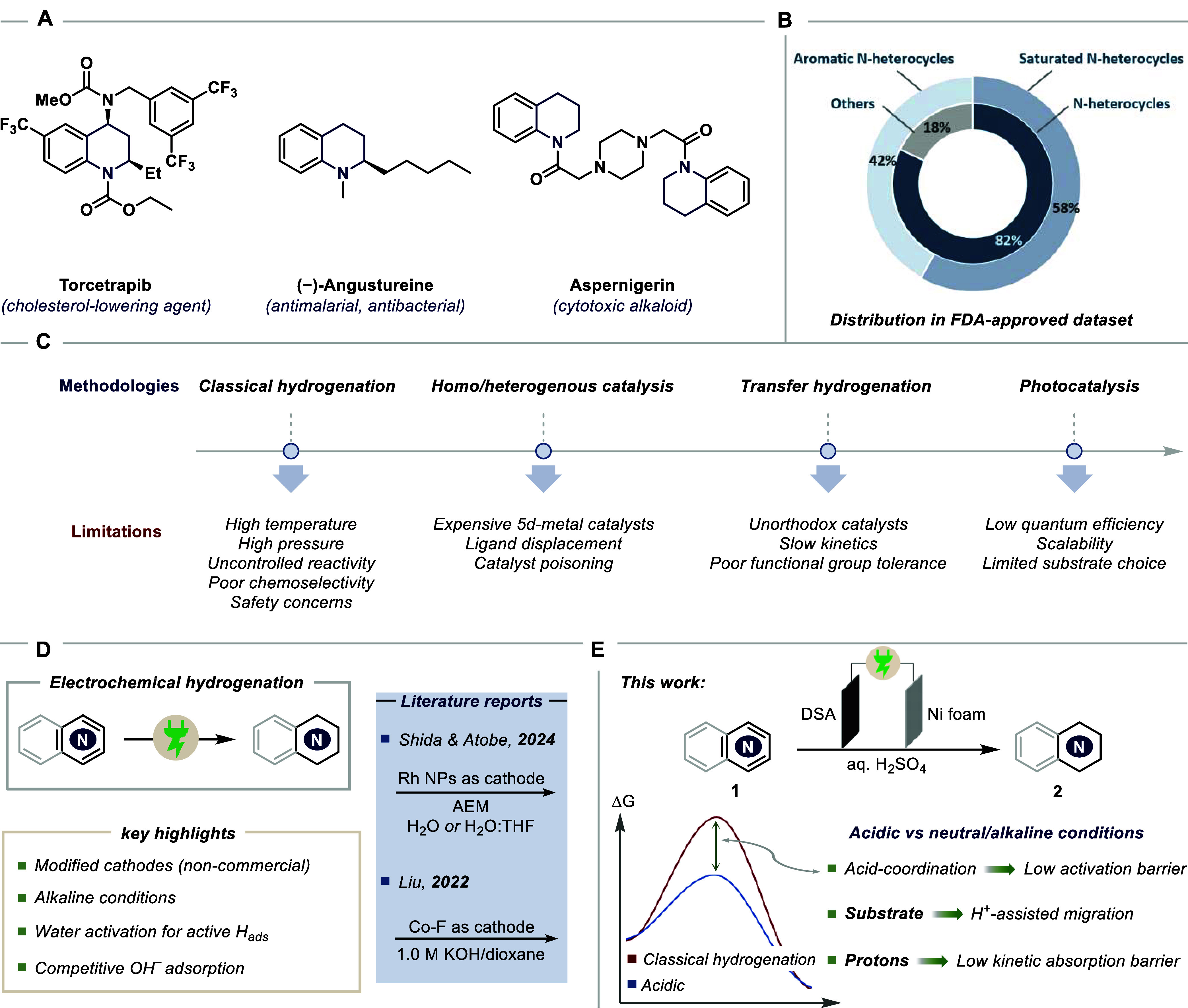
(A) Hydrogenated Aza-Arene Core in Pharmaceutically Relevant Drugs; (B) Statistics Based on FDA-approved Drug Database (*Inner Circle: Number of Drugs Having at Least One N-Heterocycle Ring; Outer Circle: Distribution in Six-Membered Ring*); (C) Methodologies for Aza-Arene Hydrogenation and Their Limitations; (D) Literature Precedence on Electrochemical Aza-Arene Hydrogenations and Related General Challenges; and (E) This Work: Electrochemical Hydrogenation of Diverse Aza-Arenes, Highlighting Benefits of Acidic Conditions; AEM: Anion Exchange Membrane; DSA: Dimensionally Stable Anode

Historically, aza-arene hydrogenation has evolved through several methodological phases and upgradation (Scheme 1C).^9^ The early emergence of classical thermal hydrogenation employed Raney Ni, Pd/C, or Pt catalysts under high H_2_ pressures and elevated temperatures. While the setup seems operationally straightforward, the harsh reaction conditions often suffer from poor chemoselectivity, low functional group tolerance, and safety concerns, which become even more pronounced when scaling up the process. Homogeneous catalytic hydrogenation later achieved improved selectivity using unorthodox metal complexes (Rh, Ir, Ru) with tailored ligands, but these systems are expensive, air- and moisture-sensitive, and difficult to recycle.^10^ Similar challenges are reported with nanoparticle-based catalysts^11^ and transfer hydrogenation methods.^12^ More recently, photocatalytic hydrogenation has enabled hydrogenation under visible light at ambient condition, but most methods work on preactivated cores (aryl substituents for radical stabilization), require superstoichiometric sacrificial organic donors, and offer low yields.^13^ Moreover, the low quantum efficiencies often downgrade the economic perspective and continue to pose challenges for scale-up.

Electrochemistry can be leveraged as a sustainable alternative, using electrons as the reductant and, in principle, eliminating the need for compressed H_2_ and precious metal catalysts.^14^ Conversely, water, being an ideal source for H_2_ and O_2_, can be envisaged to serve as a clean and benign H source, achieved by proton/electron delivery to the substrate at the cathode.^15,16^ The scientific community has already shown advancements in this direction (Scheme 1D).^17,18^ Shida, Atobe, and co-workers successfully used carbon-supported Rh nanoparticles as a cathode for the reduction of pyridines.^19,20^ Liu and co-workers employed a fluorine-modified Co-catalyst for the quinoline hydrogenation.^21^ Despite the impressive developments, in practice, state-of-the-art electrochemical literature reports exhibit significant limitations. For example, the use of H_2_ with proton-exchange membrane (PEM) reactors downgrades the sustainability aspect, albeit benefiting from the zero-gap low-voltage operations.^17^ Although synthetically valuable transformation, certain substrates display a poor yield-to-applied charge ratio, sometimes exceeding 100 *F*.^20^ More recently, a shift toward using alkaline conditions has surfaced in the regime of electrochemical hydrogenations. This, however, demands substantial advances in catalyst engineering to precisely fine-tune the electronic structure, composition, and morphology for stability in strongly alkaline environments, as nonprecious metal catalysts are prone to surface degradation.^22^ Consequently, this diminishes the potential for achieving industrially relevant productivity. In terms of reaction metrics, alkaline conditions often suffer from impeded adsorbed hydrogen kinetics, ohmic losses due to the lower mobility of OH^–^ ions, and catalyst poisoning from competitive *OH*_ads_ adsorption.^15,23^

Furthermore, functional group tolerance also becomes questionable with pH above 13, targeting free amines, hydroxyls, and halides with over-reduction or dehalogenation being a common issue. In contrast, beyond offering broad substrate compatibility, acidic conditions also enhance reaction kinetics and substrate migration via N-coordination, while significantly destabilizing the aromaticity of aza rings to facilitate hydrogenation.^24^ By comparison, neutral or alkaline media are largely diffusion-limited and lack this dual activation effect. In this regard, there is a pressing need for a similar operationally simple, sustainable, and globally scalable method that offers broad substrate compatibility, mild reaction conditions, and exceptional versatility. Here, we report a chemoselective electrochemical hydrogenation of a diverse set of aza-arenes using water as the clean hydrogen source under acidic conditions (Scheme 1E). In contrast to established E-hydrogenation of alkenes,^25,26^ alkynes,^27^ ketones,^28^ and nitriles,^29^ the hydrogenation of arenes involves an additional layer of barrier, aromatic stabilization energy.^5^ We used an inexpensive, earth-abundant, unmodified, commercially available, and highly reusable Ni foam as the cathode.^25,29–31^ This desired transformation was achieved under ambient temperature and pressure. Together, these features deliver the broadest, most functionally tolerant, and most practically scalable aqueous electro-hydrogenation of aza-arenes reported to date (see SI for detailed analysis and comparison with state-of-the-art methods).

## Results and Discussion

We commenced our study with quinoline **1a** using commercially available IrO_*x*_@Ti (DSA) and Ni foam as anode and cathode, respectively (Scheme 2).^27,31,32^ A high current density of 50 mA cm^–2^ was applied, targeting the rapidness and industrial relevance.^33^ With initial screening of acids, we obtained a 43% ^1^H NMR yield for **2a** (see SI). With further optimization, we reached the optimum yield of 83% with MeOH:H_2_O as the solvent combination. Interestingly, when a different substrate, isoquinoline **1b**, was tested under the developed conditions, it only gave a 39% yield. This prompted comprehensive optimization efforts. After multiple stages of screening and optimization,^34^ we obtained the corresponding tetrahydroisoquinoline **2b** in 86% yield with NBu_4_BF_4_ as supporting electrolyte under acetone:H_2_O conditions (see SI for more details).^35^ Graphite performed comparably well as an anode under both conditions.

**Scheme 2:**
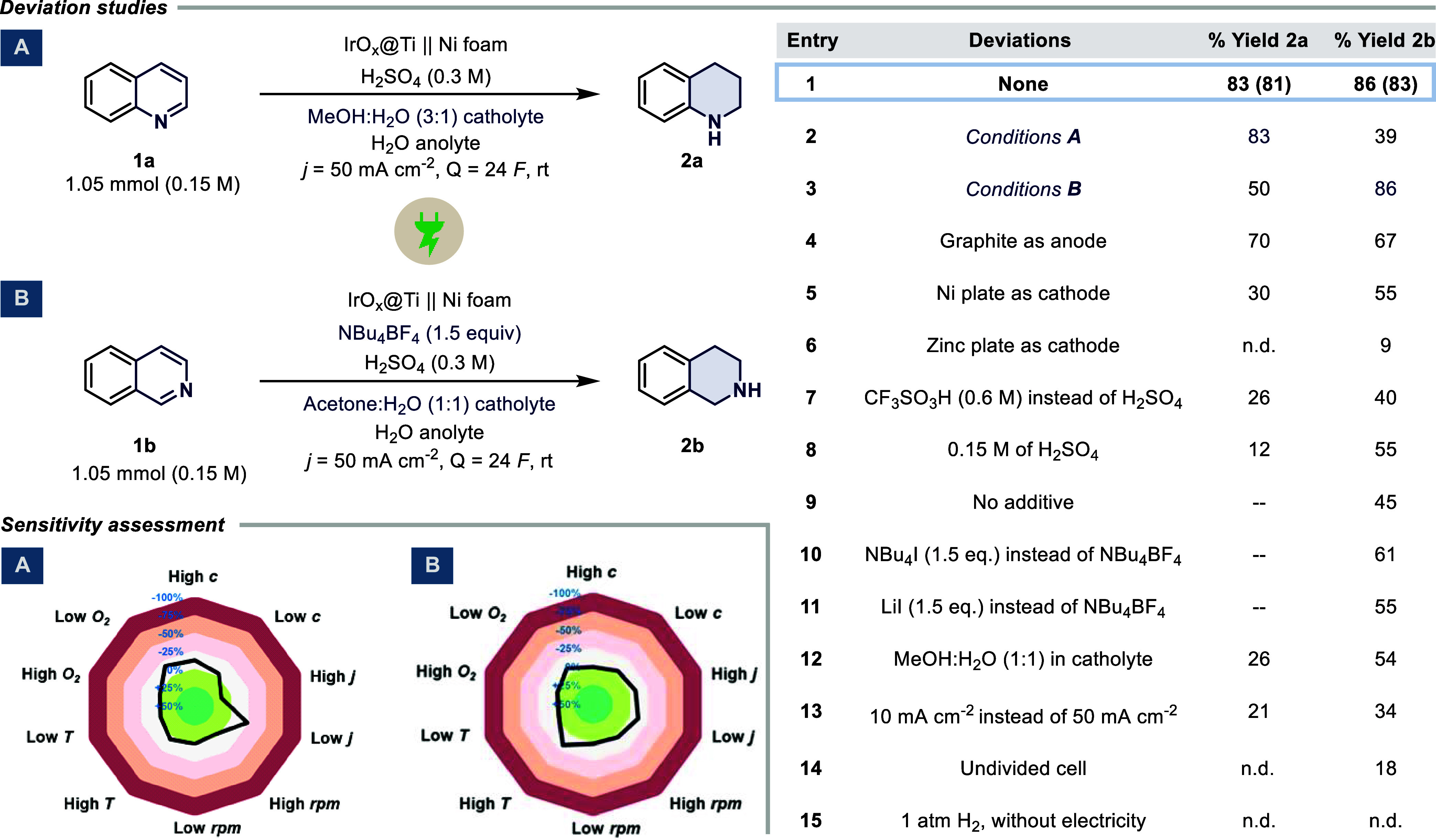
Deviation Study for Both Reaction Conditions (A and B); Condition-Based Sensitivity Screening is Shown as Radar Diagrams for Better Reproducibility (*Left:* **1a**; *Right*: **1b**; See SI for More Details); (--) = Not Applicable; N.D. = Not Detected

However, it was unsuitable due to material decomposition (entry 4). Using a Ni plate as the cathode led to lower product yields. Similarly, only trace amounts were obtained with Zn as cathode, despite its use in previous dearomatization reports (entries 5 and 6).^7^ Changing the acid to TfOH also proved unfavorable to the reaction outcome (entry 7). A similar negative trend was observed when the concentration of acid was lowered, attributed to the lower conductivity and therefore higher voltages (entry 8). The need for additional supporting electrolyte **2b** can be judged from entry 9. Given that condition B involves the use of a supporting electrolyte, we evaluated both an organic electrolyte (entry 10) and an inorganic supporting electrolyte (entry 11) for the synthesis of **2b**. Both alternatives performed less effectively than the optimized electrolyte system. Altering the polarity of medium with a more polar solvent mixture, MeOH:H_2_O (1:1), led to diminished performance (entry 12). Performing the reaction at 10 mA cm^–2^ instead of the standard 50 mA cm^–2^ resulted in a yield of 21% (**2a**) and 34% (**2b**) (entry 13). As a part of the protocol, a few control reactions were placed. The reaction in the undivided setup resulted in low to no detectable product under both conditions (entry 14). A final analysis confirmed that the reaction did not proceed in the absence of electricity, despite the presence of 1 atm of H_2_ (entry 15). The sensitivity diagram highlights the robustness and reproducibility of the reaction toward variations in most parameters.^36^ In condition A, the outcome was negatively affected by low current density, high concentration, and the presence of inert gas, with a positive trend at high current density; in conditions B, negative effects were observed only at elevated temperature and low current density.

With both optimized conditions developed, we set out to evaluate the generality of this protocol (Scheme 3). As the methodology is intended for the synthesis of essential building blocks and active pharmaceutical ingredients, achieving high yields is particularly critical.^17^ Accordingly, the amount of applied charge was increased for selected substrates of interest to ensure complete conversion. Beginning with aliphatic substitutions on the quinoline core, modifications at both the C2 (**2c**, **2e**) and C3 (**2d**) positions proceeded efficiently despite the expected steric hindrance close to the nitrogen center.

**Scheme 3:**
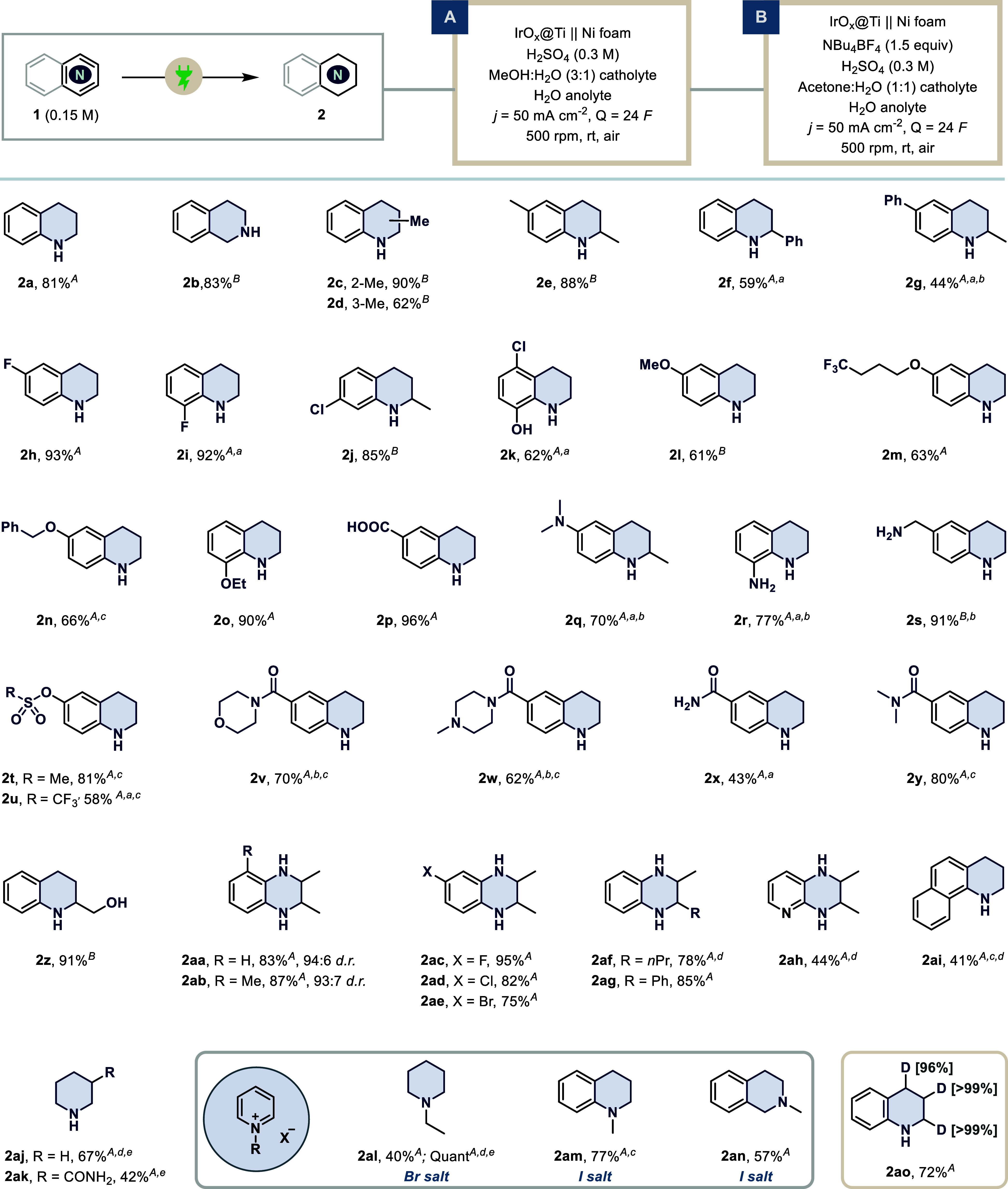
Substrate Scope of Electrochemical Hydrogenation with Isolated Yields Reported; Substrates are Mentioned with Conditions *A* or *B* as Superscript

a TfOH (4.0 equiv) was used instead of H_2_SO_4_.
b Reaction was performed with 48 *F*.
c 50 °C.
d
^1^H NMR yield is mentioned.
e Reaction was performed with 72 *F*. The quinoxaline series (**2aa**–**2ah**) was processed with 12 *F* of applied charge. **2ac**–**2ah** was obtained with >95:5 diastereoselectivity. **2ao** was synthesized using D_2_O, D_2_SO_4_, and MeOD with *conditions A*.

Incorporation of phenyl groups at the C2 (**2f**) and C6 (**2g**) positions afforded the desired chemoselective hydrogenation of the aza-ring in moderate yield, with the out-of-plane orientation relative to the electrode surface likely contributing to the diminished performance.^37^ Conventional hydrogenation under high temperature and pressure typically poses a significant risk to halide tolerance.^4,5^ In contrast, this mild protocol enabled the successful hydrogenation of aza-arenes while retaining fluorine (**2h**, **2i**) and chlorine (**2j**) substituents. Notably, Cloxiquine, an antituberculosis agent, was selectively reduced to **2k** with 62% isolated yield.^38^ This outcome also demonstrates the excellent tolerance of hydroxy groups, an otherwise challenging feature to target under basic conditions. Aliphatic ethers were also stable under the operative conditions, highlighting the suitability of methoxy (**2l**), trifluoromethyl (**2m**), benzyl (**2n**), and ethoxy (**2o**) groups. The retention of the benzyl protection is particularly interesting given its well-known susceptibility to cleavage under high-pressure classical hydrogenation. A quinoline containing carboxylic acid (**2p**) also performed exceptionally well (96% isolated yield) under the optimized condition, albeit a small amount of esterification product formed under conditions A. Generally, amines as functional groups tend to poison the hydrogenation catalysts under high temperature, thereby blocking the active sites.^39^ In contrast, this method becomes advantageous when amines, whether protected or unprotected, are subjected to the reaction conditions. We obtained the target tetrahydroquinoline core in good to excellent yields, retaining protected amines (**2q**) as well as free aromatic (**2r**) and aliphatic (**2s**) amines. Sulfonate-containing quinoline, another labile functional group, was efficiently hydrogenated under our conditions. Both mesylated (**2t**) and triflate-handles (**2u**) were well tolerated under the reaction condition. We also incorporated valuable nitrogen-rich motifs such as morpholine (**2v**) and piperazine (**2w**). These substrates also underwent smooth hydrogenation, producing the corresponding products in good yield. Similarly, free amide (**2x**) and *N,N*-dimethylamide (**2y**) also afforded the desired product in moderate to excellent yield, respectively. The preservation of the benzylic alcohol at the C2 position (**2z**) further underscores the mildness our transformation, as harsher conditions would lead to its hydrogenolysis.^40^ Apart from quinolines and isoquinolines, we also tested the suitability of the quinoxalines. Furthermore, to check the selectivity, we introduced substituents at the C2 and C3 positions. Surprisingly, the class of quinoxalines performed extremely well, offering the tetrahydroquinoxalines in excellent yields and diastereoselectivity with only 12 *F* as amounts of applied charge. This constitutes aliphatic substitutions (**2aa**, **2ab**), halo-substituents (**2ac**, **2ad**, **2ae**), and sterically demanding substituents (**2af**, **2ag**). Notably, the high diastereomeric ratio warrants a deeper discussion in the mechanistic section. This protocol also proved to be suitable for other different sets of aza-arenes, such as pyrido­[2,3-*b*]­pyrazine (**2ah**) and benzo­[*h*]­quinoline (**2ai**). Unfortunately, hydrogenation of pyridine derivatives required a higher amount of applied charge than usual, delivering the piperidines (**2aj**, **2ak**) in decent amounts. Remarkably, a new class of molecules—N-alkylated aza-arene salts—has been successfully incorporated into the reaction scope, directly yielding tertiary amines with no prior precedent in electrochemical literature. Using this approach, we successfully obtained 1-ethylpiperidine (**2al**), 1-methyl-tetrahydroquinoline (**2am**), and 2-methyl-tetrahydroisoquinoline (**2an**) in good yields. Given the importance of D incorporation in the context of drug discovery and tagging, we obtained the D3-tetrahydroquinoline **2ao** in 72% yield with an excellent level of D incorporation.^41^ Having explored the extravagant set of substrates, we turned our attention to specific drug molecule synthesis using our protocol (Scheme 4). An antifungal agent **3a** was synthesized *via* coupling of **2a** with morpholine-4-carbamoyl chloride in an overall 71% yield. Quinaldine was converted to both an antimicrobial (**3b**) and antitrypanosomal agent (**3c**) using the corresponding sulfonyl chloride as the coupling partner. Successful synthesis of Aspernigerin (**3d**) was achieved from the three-component coupling of **2a**, chloroacetyl chloride, and piperazine.^42^ Finally, (±)-Angustureine (**3e**), a prominent example of Hancock alkaloids, was prepared starting from 2-pentyl quinoline in an overall 73% yield.^43^ One of the obvious challenges in past methodologies has been scaling up reactions beyond the one-gram level.^44^ Our electrochemical setup, when integrated with a tailored flow reactor, offered a practical route to multigram-scale synthesis—an important step toward meeting industry demands for easy, efficient, and sustainable production (Scheme 5A).^45^ Running the reactions on a 4.0 mmol scale upon 10 consecutive times revealed only minor fluctuations in the yield (96 ± 3%), highlighting the excellent reusability and durability of the setup (Scheme 5B). With this in mind, we started experimenting with flow conditions for both reaction conditions. After minimal attempts, we arrived at optimized conditions of flow rate and reactor type. We selected two scalability levels, 0.5 and 2 g (Scheme 5C). In the case of condition A with **1aa**, both scales worked equally well. Intrigued by the exceptional reactivity, we pushed further to 160 mmol scale.^46^ As a highlight, we procured the corresponding hydrogenated scaffold in a 95% isolated yield (24.8 g). Isoquinoline **1b** underwent efficient hydrogenation of the aza-ring at both scales, affording the product in 79% and 89%, respectively. Moreover, the Ni foam exhibited a highly stable current–voltage profile during large-scale synthesis, indicating excellent electrode durability. This observation was further supported by SEM images, which revealed only minor surface irregularities (slight roughening and pitting), confirming the good mechanical and structural stability of the core electrode under the optimized reaction conditions.

**Scheme 4:**
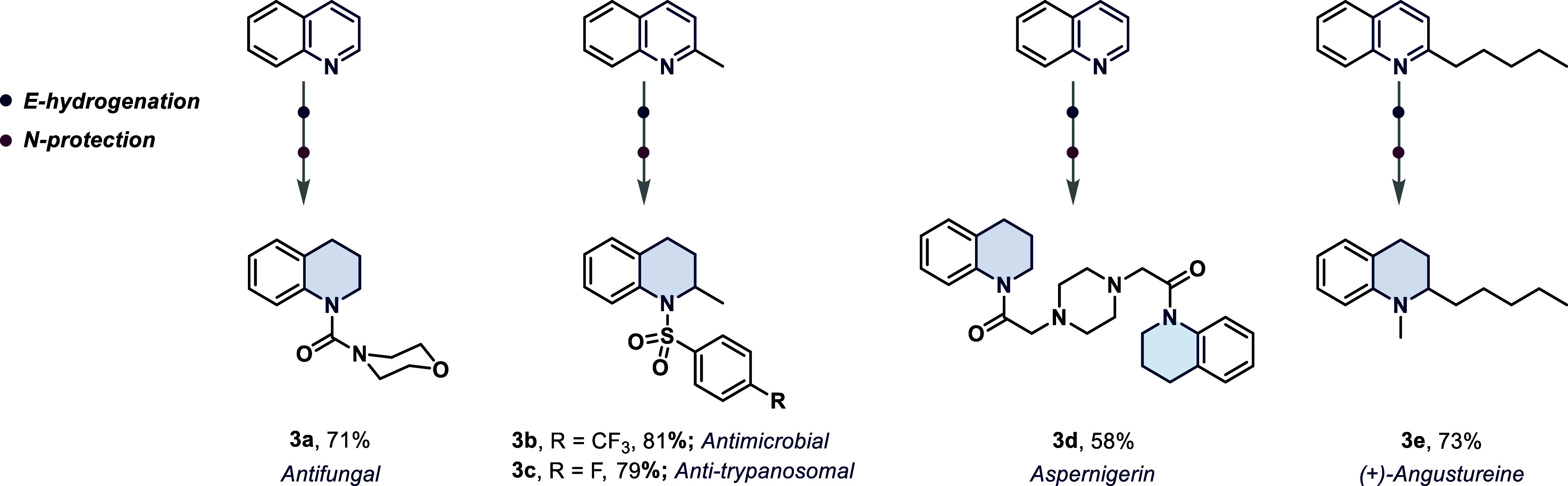
E-Hydrogenation and Postmodifications of Aza-Arene Motifs to Access Pharmaceutically Relevant Molecules^a^

a Multistep combined yields are mentioned (see SI).

**Scheme 5:**
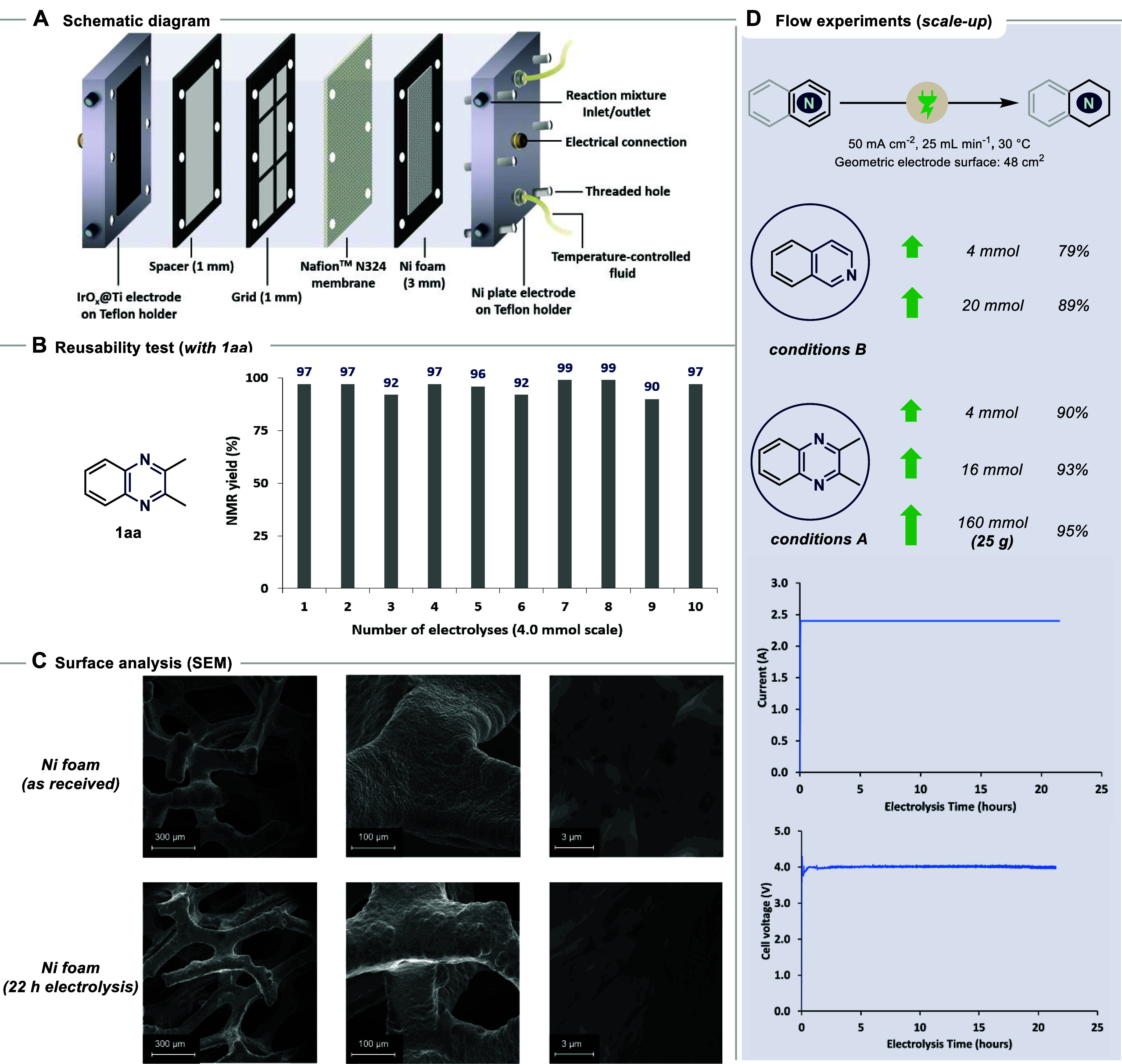
(A) Schematic Diagram for 48 cm^2^ Flow Cell Showing all the Components; (B) Reusability of Electrodes in Flow, Performed on 4.0 mmol Scale under Condition A; (C) Surface Analysis of the Ni Foam before and after Use; and (D) Scale-Up Experiments in Flow with **1aa** (with 12 *F*) and **1b** (with 48 *F*), Showing Stable Current-Voltage Profile

Following the extensive synthetic efforts, we focused on elucidating the mechanism of electrochemical hydrogenation.^47^ As a first step, cyclic voltammetry studies were conducted for all the reaction partners (H_2_SO_4_, **1a**, **1b**, **1aa**).^48^ The CV data for H_2_SO_4_ demonstrates a reduction wave at around −1.30 V *vs* FcH^+^/FcH and an oxidative wave at −0.14 V *vs* FcH^+^/FcH corresponding to oxidation of *H*_ads_ to protons (Scheme 6A). For protonated quinoline and isoquinoline, the reduction peaks were observed at approximately −1.41 V and −1.54 V, respectively (Scheme 6B,C). Interestingly, in the case of quinoxaline **1aa**, the CV data show two reduction waves, −1.0 V and −1.45 V *vs* FcH^+^/FcH (Scheme 6D). Therefore, competition between direct reduction and reduction via adsorbed hydrogen cannot be excluded.^49^ Additionally, RDE analysis of H^+^ reduction in the presence of different substrates was conducted (Scheme 6E).^50^ Quinoxaline shows an earlier onset and higher cathodic current than the blank Ni electrode, indicating a faster direct reduction of protonated quinoxaline as the initial step. In contrast, pyridine and quinoline decrease the current density, consistent with surface blocking and probable consumption of in situ-generated *H*_ads_ that slow proton reduction. Ethylpyridinium bromide, a cationic salt, mainly thickens the electrical double layer and delays the onset without changing the initial kinetic slope. Isoquinoline forms a nonproductive adsorbed layer that inhibits *H*_ads_ formation, requiring modified reaction conditions for efficient reduction. To get more insights, we performed the kinetics for the reaction with **1aa** in flow (Scheme 6F). While the reaction worked as smoothly as in batch, we observed intermediate **1aa**′ that formed and was consumed over the course of the reaction. Characterizing the NMR of crude product revealed this intermediate to be a partially hydrogenated form. Owing to the nearly perfect diastereomeric ratio, a new facet of the reaction mechanism can be proposed. It begins with the anodic oxidation of water under acidic conditions (oxygen evolution reaction),^51^ followed by the transportation of protons through the Nafion membrane.

**Scheme 6:**
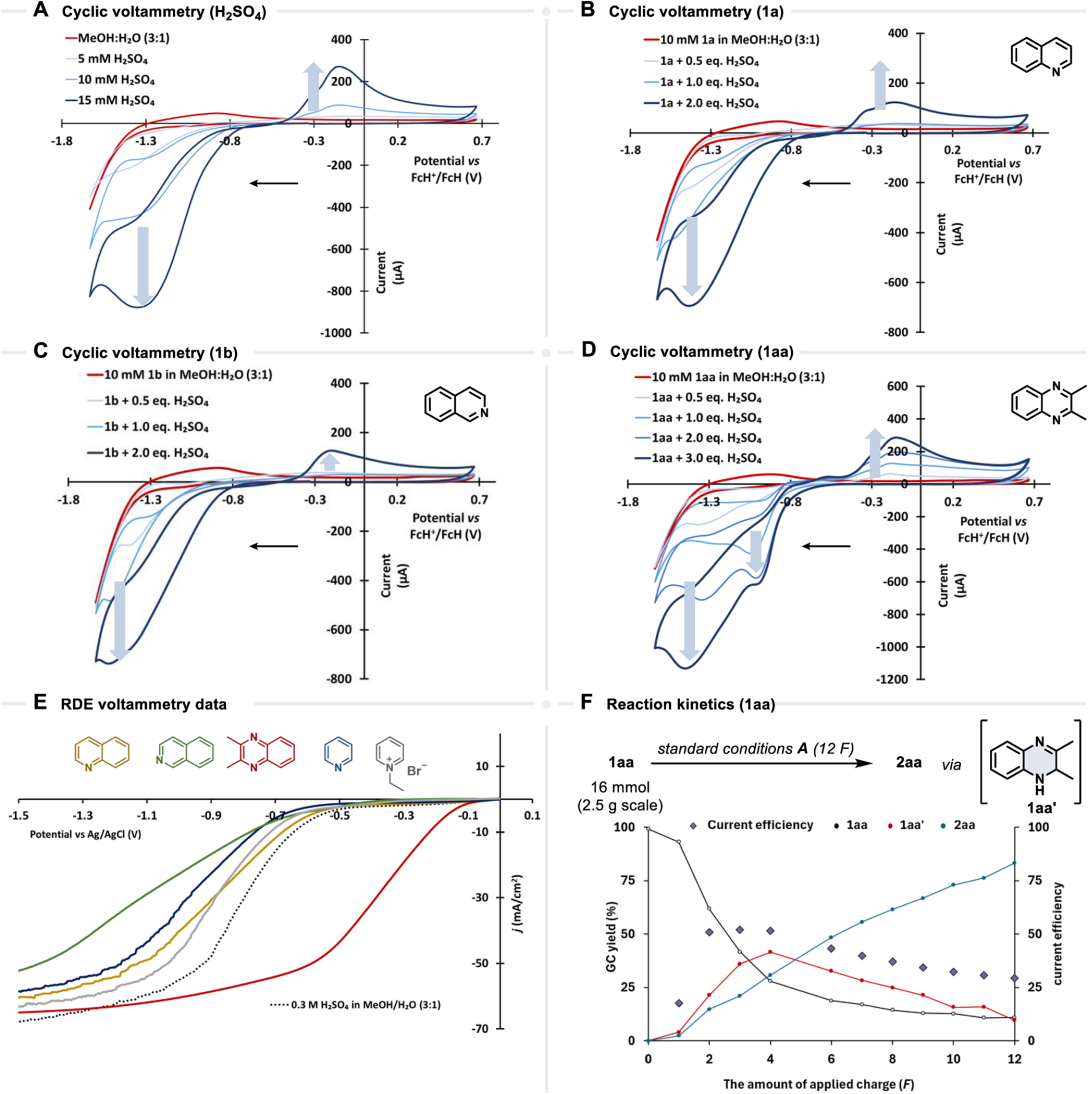
Cyclic Voltammetry Studies with Changing Concentrations (The Black Arrow Shows the Scan Direction); (A) H_2_SO_4_, (B) **1a**, (C) **1b**, (D) **1aa**; (E) RDE analysis with and without Substrates with Ni as Working Electrode; (F) Reaction Kinetics of **1aa** Showing the Intermediate **1aa**′

To investigate the role of H_2_O as a H source, a deuteration experiment was conducted with D_2_O in the anolyte as the only change, resulting in ∼32% D incorporation (see SI, Section 7.2). Then, the cathodic reduction of protons produces chemisorbed hydrogen atom (*H*_ads_) on the active sites of cathode.^15^ Similar sequential interactions with the adsorbed protonated substrate on the surface result in the formation of hydrogenated aza-arene. In the case of quinoxaline **1aa**, its lower negative reduction potential (also early onset on RDE analysis) advocates for a facile first direct reduction over hydrogenation with *H*_ads_, generating **1aa**′. The high *d.r*. ratio can be rationalized from a competing hydrogenation of **1aa**′ via *H*_ads_ in preference to the second direct reduction of protonated **1aa**′. The former pathway proceeds from the less sterically hindered site, yielding the *cis*-product as the major isomer (Scheme 7). To assert the role of *H*_ads_, the same reaction was performed on glassy carbon (GC) electrodes. This resulted in a diminished yield of 27% with 52:48 as the diastereomeric ratio, stemming from the two consecutive direct reductions of the **1aa**, in comparison to our proposed *H*_ads_-mediated transformation with 94:6 *d.r*. (see SI for more detailed analysis).

**Scheme 7:**
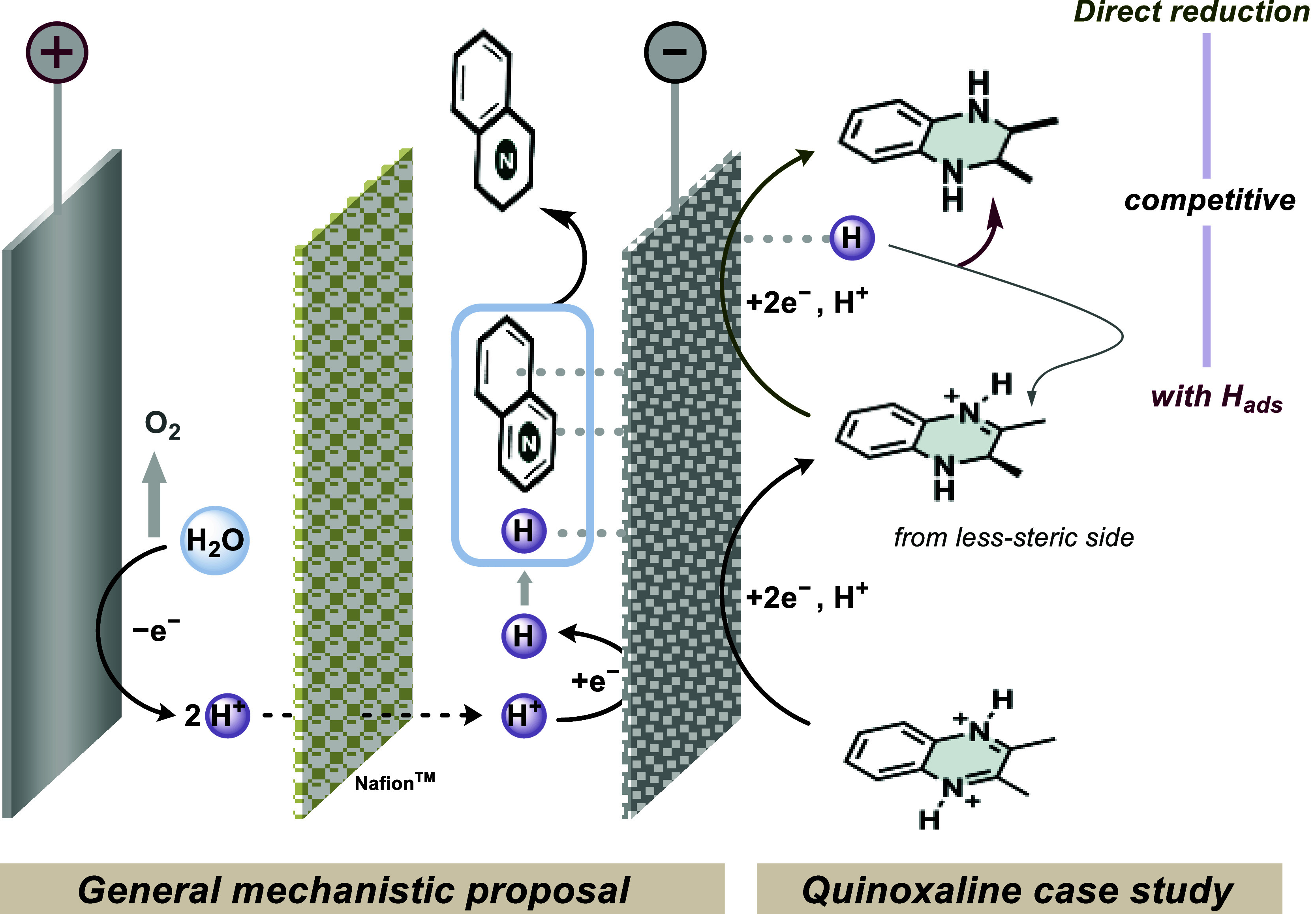
Plausible Mechanistic Proposal

In conclusion, we have established a simple, clean, and sustainable way of hydrogenating diverse sets of aza-arene with the broadest generality in terms of substrates. Utilizing water as the hydrogen source under acidic conditions strongly emphasizes the method’s alignment with green chemistry principles. Moreover, the choice of readily available electrodes further highlights its practicality. Our approach enabled the effortless synthesis of pharmaceutically relevant compounds, and its scalability—demonstrated up to a 25 g scale—highlights its industrial potential. As a mechanistic highlight, two plausible routes were proposed based on the redox window for the protonated substrate: one involving hydrogenation with *H*_ads_, while the other begins with the direct substrate reduction followed by reaction with *H*_ads_. Overall, we anticipate that the current methodology will attract considerable interest from both academic and industrial communities and instigate further developments in the field of sustainable electrochemical hydrogenation.

## Supplementary Material



## Notes

All experimental data and characterization of compounds synthesized (PDF)

## References

[1] Vitaku E., Smith D. T., Njardarson J. T. (2014). Analysis
of the structural diversity, substitution patterns, and frequency
of nitrogen heterocycles among U.S. FDA approved pharmaceuticals. J. Med. Chem..

[2] Testa B., Crivori P., Reist M., Carrupt P.-A. (2000). The influence
of lipophilicity on the pharmacokinetic behavior of drugs: Concepts
and examples. Perspect. Drug Discovery Des..

[3] Wei W., Cherukupalli S., Jing L., Liu X., Zhan P. (2020). Fsp3: A new
parameter for drug-likeness. Drug
Discovery Today.

[4] Lückemeier L., Pierau M., Glorius F. (2023). Asymmetric
arene hydrogenation: towards
sustainability and application. Chem. Soc. Rev..

[5] Wiesenfeldt M. P., Nairoukh Z., Dalton T., Glorius F. (2019). Selective Arene Hydrogenation
for Direct Access to Saturated Carbo- and Heterocycles. Angew. Chem. Int. Ed..

[6] Faheem, Karan Kumar B., Chandra Sekhar K. V.
G., Chander S., Kunjiappan S., Murugesan S. (2021). Medicinal chemistry perspectives
of 1,2,3,4-tetrahydroisoquinoline analogs - biological activities
and SAR studies. RSC Adv..

[7] Peters B. K., Rodriguez K. X., Reisberg S. H., Beil S. B., Hickey D. P., Kawamata Y., Collins M., Starr J., Chen L., Udyavara S., Klunder K., Gorey T. J., Anderson S. L., Neurock M., Minteer S. D., Baran P. S. (2019). Scalable
and safe
synthetic organic electroreduction inspired by Li-ion battery chemistry. Science.

[8] Cyrański M. K. (2005). Energetic
aspects of cyclic pi-electron delocalization: evaluation of the methods
of estimating aromatic stabilization energies. Chem. Rev..

[9] Wang D.-S., Chen Q.-A., Lu S.-M., Zhou Y.-G. (2012). Asymmetric hydrogenation
of heteroarenes and arenes. Chem. Rev..

[10] Johnson N.
B., Lennon I. C., Moran P. H., Ramsden J. A. (2007). Industrial-scale synthesis and applications
of asymmetric
hydrogenation catalysts. Acc. Chem. Res..

[11] Zhang L., Zhou M., Wang A., Zhang T. (2020). Selective Hydrogenation over Supported Metal Catalysts: From Nanoparticles
to Single Atoms. Chem. Rev..

[12] Wang D., Astruc D. (2015). The golden age of transfer
hydrogenation. Chem. Rev..

[13] Zhang J., Spreckelmeyer N., Lammert J., Wiethoff M.-A., Milner M. J., Mück-Lichtenfeld C., Studer A. (2025). Photocatalytic Hydrogenation of Quinolines to Form
1,2,3,4-Tetrahdyroquinolines Using Water as the Hydrogen Atom Donor. Angew. Chem. Int. Ed..

[14] Kärkäs M. D. (2018). Electrochemical
strategies for C-H functionalization and C-N bond formation. Chem. Soc. Rev..

[15] Liu C., Chen F., Zhao B.-H., Wu Y., Zhang B. (2024). Electrochemical
hydrogenation and oxidation of organic species involving water. Nat. Rev. Chem..

[16] Li M., Liu C., Zhang B. (2021). Using water
as the hydrogen source for electrocatalytic transfer hydrogen storage. Sci. Bull..

[17] Mitsudo K., Osaki A., Inoue H., Sato E., Shida N., Atobe M., Suga S. (2024). Electrocatalytic hydrogenation
of
cyanoarenes, nitroarenes, quinolines, and pyridines under mild conditions
with a proton-exchange membrane reactor. Beilstein
J. Org. Chem..

[18] Pan Y., Bao Z., Wang C., Wang Z., Xu P., Bai X., Shi X., Zheng H., Wang H.-E., Zheng L. (2025). Electrochemical
Hydrogenation
of Quinoline Enabled by Cu 0 -Cu + Dual Sites Coupled with Efficient
Biomass Valorization in Aqueous Solution. Adv.
Funct. Mater..

[19] Fukazawa A., Shimizu Y., Shida N., Atobe M. (2021). Electrocatalytic hydrogenation
of benzoic acids in a proton-exchange membrane reactor. Org. Biomol. Chem..

[20] Shida N., Shimizu Y., Yonezawa A., Harada J., Furutani Y., Muto Y., Kurihara R., Kondo J. N., Sato E., Mitsudo K., Suga S., Iguchi S., Kamiya K., Atobe M. (2024). Electrocatalytic Hydrogenation of Pyridines and Other Nitrogen-Containing
Aromatic Compounds. J. Am. Chem. Soc..

[21] Guo S., Wu Y., Wang C., Gao Y., Li M., Zhang B., Liu C. (2022). Electrocatalytic hydrogenation
of quinolines with water over a fluorine-modified
cobalt catalyst. Nat. Commun..

[22] Kwon J., Choi S., Park C., Han H., Song T. (2023). Critical challenges
and opportunities for the commercialization of alkaline electrolysis:
high current density, stability, and safety. Mater. Chem. Front..

[23] Wang J., Xu F., Jin H., Chen Y., Wang Y. (2017). Non-Noble Metal-based Carbon Composites
in Hydrogen Evolution Reaction: Fundamentals to Applications. Adv. Mater..

[24] Garcia-Torres E., Herbert D. E. (2024). Electrochemical
Hydrogenation of N- Heterocycles and Related Substrates: A Mini-Review. Electrochem. Sci. Adv..

[25] Tortajada P. J., Kärnman T., Martínez-Pardo P., Nilsson C., Holmquist H., Johansson M. J., Martín-Matute B. (2024). Electrochemical
hydrogenation of alkenes over a nickel foam guided by life cycle,
safety and toxicological assessments. Green
Chem..

[26] Fukazawa A., Minoshima J., Tanaka K., Hashimoto Y., Kobori Y., Sato Y., Atobe M. (2019). A New Approach to Stereoselective
Electrocatalytic Semihydrogenation
of Alkynes to Z -Alkenes using a Proton-Exchange Membrane Reactor. ACS Sustainable Chem. Eng..

[27] Valiente A., Martínez-Pardo P., Kaur G., Johansson M. J., Martín-Matute B. (2022). Electrochemical Proton Reduction over Nickel Foam for
Z-Stereoselective Semihydrogenation/deuteration of Functionalized
Alkynes. ChemSusChem.

[28] Green S. K., Tompsett G. A., Kim H. J., Bae Kim W., Huber G. W. (2012). Electrocatalytic
reduction of acetone in a proton-exchange-membrane reactor: a model
reaction for the electrocatalytic reduction of biomass. ChemSusChem.

[29] Narobe R., Perner M. N., Gálvez-Vázquez M. d. J., Kuhwald C., Klein M., Broekmann P., Rösler S., Cezanne B., Waldvogel S. R. (2024). Practical
electrochemical hydrogenation of nitriles at the nickel foam cathode. Green Chem..

[30] Ratsoma M. S., Poho B. L. O., Makgopa K., Raju K., Modibane K. D., Jafta C. J., Oyedotun K. O. (2023). Application
of Nickel Foam in Electrochemical Systems: A Review. J. Electron. Mater..

[31] Zheng W., Liu M., Lee L. Y. S. (2020). Best Practices in Using Foam-Type Electrodes for Electrocatalytic
Performance Benchmark. ACS Energy Lett..

[32] Heard D. M., Lennox A. J. J. (2020). Electrode Materials
in Modern Organic Electrochemistry. Angew. Chem.
Int. Ed..

[33] Leow W. R., Lum Y., Ozden A., Wang Y., Nam D.-H., Chen B., Wicks J., Zhuang T.-T., Li F., Sinton D., Sargent E. H. (2020). Chloride-mediated selective electrosynthesis of ethylene
and propylene oxides at high current density. Science.

[34] Dörr M., Hielscher M. M., Proppe J., Waldvogel S. R. (2021). Electrosynthetic
Screening and Modern Optimization Strategies for Electrosynthesis
of Highly Value-added Products. ChemElectroChem.

[35] Mast F., Hielscher M. M., Wirtanen T., Erichsen M., Gauss J., Diezemann G., Waldvogel S. R. (2024). Choice of the Right Supporting Electrolyte
in Electrochemical Reductions: A Principal Component Analysis. J. Am. Chem. Soc..

[36] Schäfer F., Lückemeier L., Glorius F. (2024). Improving reproducibility through condition-based sensitivity
assessments: application, advancement and prospect. Chem. Sci..

[37] Förster H., Vögtle F. (1977). Steric Interactions in Organic Chemistry: Spatial Requirements
of Substituents. Angew. Chem. Int. Ed..

[38] Zhang W., Shao W., Dong Z., Zhang S., Liu C., Chen S. (2019). Cloxiquine, a traditional
antituberculosis agent, suppresses the growth and metastasis of melanoma
cells through activation of PPARγ. Cell
Death Dis..

[39] Philippov A.
A., Martyanov O. N. (2021). Poisoning
effect of N-containing compounds on performance of Raney nickel in
transfer hydrogenation. Catal. Commun..

[40] Cheng G., Zhang W., Jentys A., Ember E. E., Gutiérrez O. Y., Liu Y., Lercher J. A. (2022). Importance of interface open circuit potential on aqueous
hydrogenolytic reduction of benzyl alcohol over Pd/C. Nat. Commun..

[41] J Pagliero R., Mercado R., McCracken V., R Mazzieri M., J Nieto M. (2011). Rapid and Facile Synthesis
of N-Benzenesulfonyl
Derivatives of Heterocycles and their Antimicrobial Properties. Lett. Drug Des. Discovery.

[42] Shen L., Ye Y.-H., Wang X.-T., Zhu H.-L., Xu C., Song Y.-C., Li H., Tan R.-X. (2006). Structure and total
synthesis of aspernigerin: a novel cytotoxic endophyte metabolite. Chem. Eur. J..

[43] Davies S. G., Fletcher A. M., Roberts P. M., Thomson J. E. (2019). The Hancock
Alkaloids Angustureine, Cuspareine, Galipinine,
and Galipeine: A Review of their Isolation, Synthesis, and Spectroscopic
Data. Eur. J. Org. Chem..

[44] McMullen J. P., Marton C. H., Sherry B. D., Spencer G., Kukura J., Eyke N. S. (2018). Development and
Scale-Up of a Continuous Reaction for Production of an Active Pharmaceutical
Ingredient Intermediate. Org. Process Res. Dev..

[45] Kleinhaus J. T., Wolf J., Pellumbi K., Wickert L., Viswanathan S. C., Junge Puring K., Siegmund D., Apfel U.-P. (2023). Developing electrochemical hydrogenation
towards industrial application. Chem. Soc. Rev..

[46] Lehnherr D., Chen L. (2024). Overview of Recent
Scale-Ups in Organic Electrosynthesis (2000–2023). Org. Process Res. Dev..

[47] Sandford C., Edwards M. A., Klunder K. J., Hickey D. P., Li M., Barman K., Sigman M. S., White H. S., Minteer S. D. (2019). A synthetic
chemist’s guide to electroanalytical tools for studying reaction
mechanisms. Chem. Sci..

[48] Espinoza E.
M., Clark J. A., Soliman J., Derr J. B., Morales M., Vullev V. I. (2019). Practical
Aspects
of Cyclic Voltammetry: How to Estimate Reduction Potentials When Irreversibility
Prevails. J. Electrochem. Soc..

[49] Kulisch J., Nieger M., Stecker F., Fischer A., Waldvogel S. R. (2011). Efficient and stereodivergent electrochemical
synthesis of optically pure menthylamines. Angew.
Chem. Int. Ed..

[50] Mondal R., Galmidi L., Tzaguy A., Sason T., Feller M., Iron M. A., Avram L., Neumann R., Gnaim S. (2025). J. Am. Chem. Soc..

[51] Klein M., Waldvogel S. R. (2022). Counter
Electrode Reactions-Important Stumbling Blocks
on the Way to a Working Electro-organic Synthesis. Angew. Chem. Int. Ed..

