# Supplementary material for: Electrochemical Hydrogenation of Aza-Arenes Using H2O as H Source
Source: J Am Chem Soc. 2025 Dec 23;148(1):2048–58. doi: 10.1021/jacs.5c21117 (PMC12814348; doi:10.1021/jacs.5c21117)
Supplement: Supplementary file 1 [file ja5c21117_si_001.pdf]

Supporting Information

Electrochemical Hydrogenation of Aza-arenes using H_2O as H source

Subhabrata Dutta,[a,§] Rok Narobe,[a,§] Siegfried R. Waldvogel[a,b,*]

[§] *denotes equal contribution*

[a]*Max-Planck-Institute for Chemical Energy Conversion, Department of Electrosynthesis, Stiftstraße 34–36, 45470 Mülheim an der Ruhr, Germany.*

[b]*Karlsruhe Institute of Technology, Institute of Biological and Chemical Systems – Functional Molecular Systems (IBCS-FMS), Kaiserstraße 12, 76131 Karlsruhe, Germany.*

Corresponding author email: siegfried.waldvogel@cec.mpg.de

Table of Contents

1 General information ... S3
2 General experimental procedures and characterization data ... S8
2.1 Starting material synthesis ... S8
2.2 Screening and optimization in batch-type cells ... S12
2.3 Deviation study ... S20
2.4 Sensitivity assessment ... S21
3 Scope ... S23
4 Synthetic applications ... S40
5 Flow experiments ... S45
6 Mechanistic studies ... S49
6.1 Cyclic voltammetry ... S49
6.2 RDE linear sweep voltammetry ... S53
6.3 Deuteration and Scavenging studies ... S55
6.4 Kinetic monitoring and identification of the intermediate ... S58
7 Reusability and reproducibility study ... S65
8 Surface analysis ... S67
8.1 SEM analysis ... S67
8.2 XPS analysis ... S69
9 Quinoxaline case study ... S70
10 Comparison with the state-of-the-art methodologies ... S74
11 NMR spectra ... S75
12 References ... S121

1 General information

Starting materials and reagents were purchased from commercial suppliers (Sigma Aldrich, TCI, Alfa Aesar, Acros, BLD Pharma, and Fluka) and were used without further purification. Solvents were used as p.a. grade whereas high purity water was obtained by circulating deionized water through a Milli-Q® water purification system. Reactions were monitored by GC-FID, GC-MS and UPLC-MS. Analytical thin layer chromatography (TLC) was performed on silica gel 60 F254 aluminum plates (Merck). TLC plates were visualized by exposure to short wave ultraviolet light (254 nm and 356 nm) and/or were dipped into a solution of $KMnO_4$ stain. The stain mentioned was prepared with $KMnO_4$ (1.5 g), K_2CO_3 (10.0 g) and KOH (1.5 mL of 10% solution) in H_2O (200 mL).

Power supply

Galvanostat Rohde & Schwarz - HMP4040 was used as a DC power supply in all the electrochemical reactions. The experiments were performed under galvanostatic conditions using a simple two-electrode reaction setup.

Electrodes

- Nickel foam was purchased from the company Recemat BV, Netherlands. The foam was cut to the desired dimensions with a regular disc-shaped rotating blade saw.
    - In batch-type screening cells, 3.0 mm thick nickel foam "Ni4753" were used with an average pore size of 0.40 mm.
    - In a large 4 cm x 12 cm flow cell, we used 3.0 mm thick nickel foam "Ni4753" with an average pore size of 0.40 mm.
- Nickel plate electrode was obtained from IKA Werke GmbH & Co. KG, Germany.
- Iridium oxide coated titanium dimensionally stable anodes (DSA) were obtained from DeNora, Italy with 12 g Ir/m^2 on titanium as support.
- Zinc electrodes were obtained from Grillo-Werke AG, Duisburg, Germany
- Carbon graphite electrode (Highly isostatic, V2100) was obtained from SGL Carbon, Bonn, Germany.
- Boron-doped diamond (BDD) with a DIACHEM™ 15 µm diamond layer on silicon were obtained from CONDIAS GmbH, Itzehoe, Germany.

Cation exchange membrane

Sulfonic acid-based cation exchange membrane Nafion^{TM} N324 membrane was obtained from Ion Power GmbH, Munich, Germany. Prior to use it was conditioned in ca. 5% aq. H_2SO_4. We kept reusing the membranes unless we noticed any visible damage or dark staining of the pores.

NMR spectroscopy

^{1}H NMR and ^{13}C{^{1}H} NMR spectra were recorded at 25 °C on a Bruker AVANCE III HD 400 MHz NMR spectrometer with a Bruker Prodigy probe (Bruker BioSpin GmbH, Rheinstetten, Germany). Chemicals shifts (δ) are quoted in ppm downfield of tetramethylsilane. The residual solvent signals purchased form Deutero were used as references for ^{1}H and ^{13}C NMR spectra (relative to Tetramethylsilane at 0.0 ppm, $CDCl_3$: δH = 7.26 ppm, δC = 77.16 ppm, CD_2Cl_2: δH = 5.32 ppm, δC = 54.00 ppm). Chemical shifts (*δ*) are reported in parts per million (ppm). ^{19}F NMR chemical shifts are reported in ppm relative to $CFCl_3$ (δ = 0.0 ppm) using the IUPAC-recommended Ξ value for indirect referencing. All chemical shifts are reported in *δ*-scale as parts per million [ppm] (multiplicity, coupling constant *J*, number of protons), relative to the solvent residual peaks as the internal standard. Coupling constants *J* are given in Hertz [Hz]. Abbreviations used for signal multiplicity: ^{1}H NMR: s = singlet, d = doublet, t = triplet, q = quartet and m = multiplet. All the NMRs were processed using Mestrenova 14 applying standard phase and baseline corrections. Coupling constants (*J*) are quoted in Hz.

Ultra-performance liquid chromatography – mass spectrometry (UPLC-MS)

Ultra-performance liquid chromatography – mass spectrometry (UPLC-MS) was performed on a Waters™ ACQUITY™ UPLC™ H-Class PLUS System (Waters Corporation, Milford, USA) using a quaternary solvent manager (ACQ H-CLASS QSM PLUS), a sample manager with flow-through needle (ACQ H-Class FTN-H PLUS) design, a column heater (ACQUITY UPLC CM-A) and an ACQUITY UPLC® BEH C18 1.7 µm 2.1 x 50 mm column. Mass spectra were measured using a single quadrupole mass detection (ACQUITY QDa Detector) employing ESI+. Acetonitrile (HPLC-MS grade) and water (Milli-Q®) were used as eluents, eluent with 0.1% (v/v) formic acid was added directly before mass detection using a second isocratic solvent manager (Waters™ ACQ Isoc Solvent Mgr).

Gas chromatography (GC-FID and GC-MS)

GC measurements were performed on a GCMS QP2010SE (Shimadzu, Kyoto, Japan) equipped with either flame ionization detector (FID) or an electron ionization (EI) source and a quadrupole mass analyzer. A quartz capillary column Zebron ZB-5PLUS (Avantor VWR, Radnor, USA) with the following specification was used: length 30 m, int. diam 0.25 mm, film 0.25 µm as dimensions. Helium was used as carrier gas (1.2 mL/ min, constant flow). The GC temperature ramp started at 50 °C (holding for 1 min) and heated to

300 °C (holding for 4.71 min) with a temperature ramp of 17.5 °C/min (total program time: 20.0 min). Measurements were performed at an injector temperature of 270 °C and a temperature of the EI source of 250 °C.

Cyclic voltammetry (CV)

Cyclic voltammetry was conducted using a three-electrode setup consisting of a nickel disc working electrode ‘WE’ (r = 2 mm, Parafilm® wrapped Ni rod with exposed tip), Ag/AgCl reference electrode ‘RE’ (eDAQ) and a glassy carbon rod counter-electrode ‘CE’. Before use and between measurements, the working electrode was mechanically cleaned by polishing with diamond polishing suspension (Buehler Metadi, 1 Micron particle size) followed by rinsing with MeOH and distilled water. Supporting electrolyte NBu_4BF_4 was used as obtained commercially from TCI (98%+). Unless otherwise stated, all analytes were prepared as 10 mM solution in 0.1 M NBu_4BF_4 in 10 mL MeOH:H_2O (3:1). The solutions were purged with Ar for 15 min prior to measurement. All data is displayed against the half-wave potential of ferrocene/ferrocenium redox couple (FcH/FcH^+) as internal reference which was measured directly before the presented set of measurements in MeOH/H_2O. For the measurement of (FcH/FcH^+) redox couple we dissolved ca. 15 mg of ferrocene in 0.1 M NBu_4BF_4 in MeOH/H_2O 3:1 using ultrasonification. All analytes were prepared as 10 mM solutions in 0.1 M NBu_4BF_4 in 3:1 MeOH-H_2O. The solutions were purged with Ar for 15 min prior to measurement. Electrochemical measurements were carried out using an Autolab PGSTAT204 potentiostat at room temperature (ca. 298 K). The scanning rate was 100 mV/sec and each measurement were performed in 2 scans starting in a reductive direction, unless otherwise stated. Data acquisition and processing were performed with Metrohm Autolab Nova 42.1.10.4.1.11.

Linear sweep voltammetry (LSV)

All RDE analyses were carried out using a Metrohm Autolab PGSTAT302N multi-channel potentiostat with a Metrohm RDE80790 rotating disk electrode. For the measurements we used Ni (d = 4 mm), GC (d = 3 mm), or Pt (d = 3 mm) rotating disc working electrode in a three-electrode setup in combination with Pt disc counter electrode (d = 3 mm) and “leakless” Ag/AgCl reference electrode under inert Ar atmosphere at relatively slow 10 mV/sec scan rate. We measured currents in 0.3 M H_2SO_4 in MeOH/H_2O (3:1) solution without any supporting electrolyte in the presence of 0.15 M substrates, directly mimicking reaction conditions. The measurements were performed between 100-2000 rpm values. Before every LSV, the system was purged using Ar.

Mass spectrometry

High resolution Mass Spectrometry (HRMS) experiments were performed on a Thermo Scientific™ Q Exactive Plus or a Thermo Scientific™ Q Exactive GC Orbitrap device.

XPS

The XPS is built by SPECS. A Phoibos 150 R3 NAP HR analyzer was used with a 1D-DLD detector. For X-Ray excitation, a monochromated XR50MF aluminium source (Al k-alpha = 1486,6eV) was used. The spot size on the sample is about 1–1.5 mm, and the nozzle diameter of the Analyzer is 800 µm. Step size and pass energy settings are 1eV and 100eV for Survey, and 0.1 eV and 20eV for high-resolution scans.

Scanning electron microscopy

The morphology of the nickel foam was examined using a Phenom Pharos G1 scanning electron microscope (SEM) operated at an accelerating voltage of 10 kV and a chamber pressure of 0.1 Pa, with a working distance ranging from 7.10 to 7.67 mm. Image acquisition and elemental analysis were performed using Phenom ProSuite and Phenom Element Identification software.

Electrolysers

Batch-type screening experiments were conducted in divided Teflon™ electrolysis cells. (**Figure S1**) which are commercially available as IKA Screening S8 system package (IKA®-Werke GmbH & Co. KG, Germany). Nafion™ N324 membrane (Ion Power GmbH, Munich, Germany) was used as a separator material between the two compartments. The membrane was conditioned in 5% aq. H_2SO_4 for at least 4 hours, washed with fresh H_2O and wiped before being installed in the electrochemical cell. Each cell compartment was equipped with a magnetic stirring bar. Both electrodes, each with exposed geometric surface of 3 cm^2 (submerged part of the electrode in a typical experiment) were arranged at 20 mm distance relative to each other. The reactions were typically performed at room temperature and constant stirring rate 500 rpm.

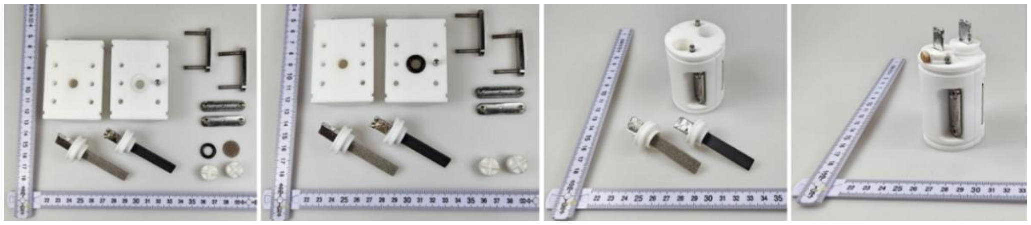

Figure S1: Batch-type cells and assembly process. Ruler scale in centimeters.

Experiments in recirculating mode were conducted in a modular flow cell designed by the Waldvogel research group and built by the workshop at University of Mainz (**Figure S2**). As a cathode we used 40 mm x 120 mm nickel plate electrode which is in contact with nickel foam ("Ni 4753", 3.0 mm thick, average pore size 0.40 mm). The foam was cut with a cutter knife to fit tightly into a 36 mm x 131 mm opening in

a 3 mm thick Teflon™ spacer. The other half-cell contains 40 mm x 120 mm DSA electrode (IrO_x on Ti).The two half-cell compartments were separated by Nafion™ N324 cation exchange membrane (Ion Power GmbH, Munich, Germany). On the anolyte side of the membrane, Teflon grid was used to prevent contact between DSA electrode and Nafion™ membrane. The interelectrode gap between DSA electrode and nickel foam is approx. 2.1 mm. Both catholyte and anolyte were pumped through the cell with the same flow rate 25 mL/min using a peristaltic pump with two channels.[1]

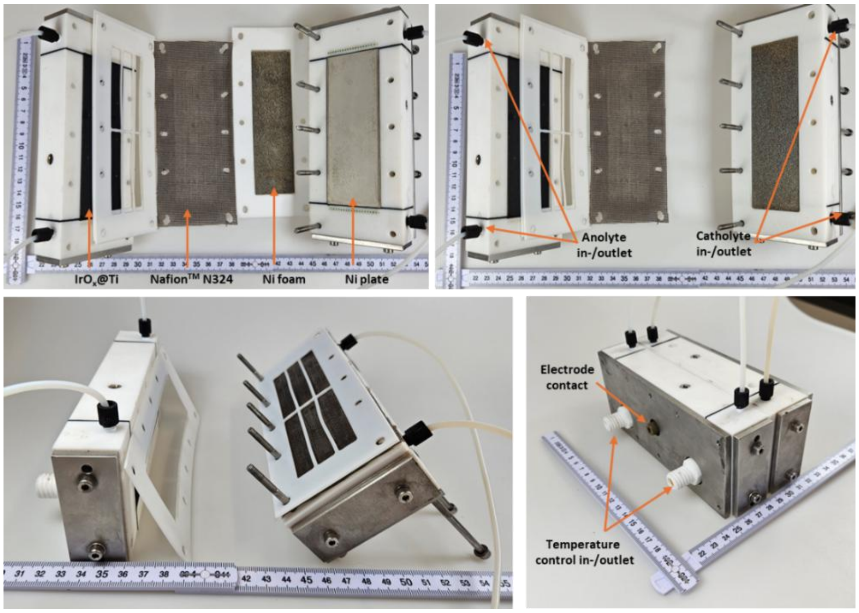

Figure S2: Flow-type reactor and assembly process. Ruler scale in centimeters.

Note: *A smaller version of the electrolyser with 2 cm × 6 cm electrode surface is commercially available as ElectraSyn flow (IKA®-Werke GmbH & Co. KG, Germany).*

2 General experimental procedures and characterization data

2.1 Starting material synthesis

Aza-arenes (used in the substrate screening)

Aza-arenes used in the scope (commercially available, lab-synthesized)

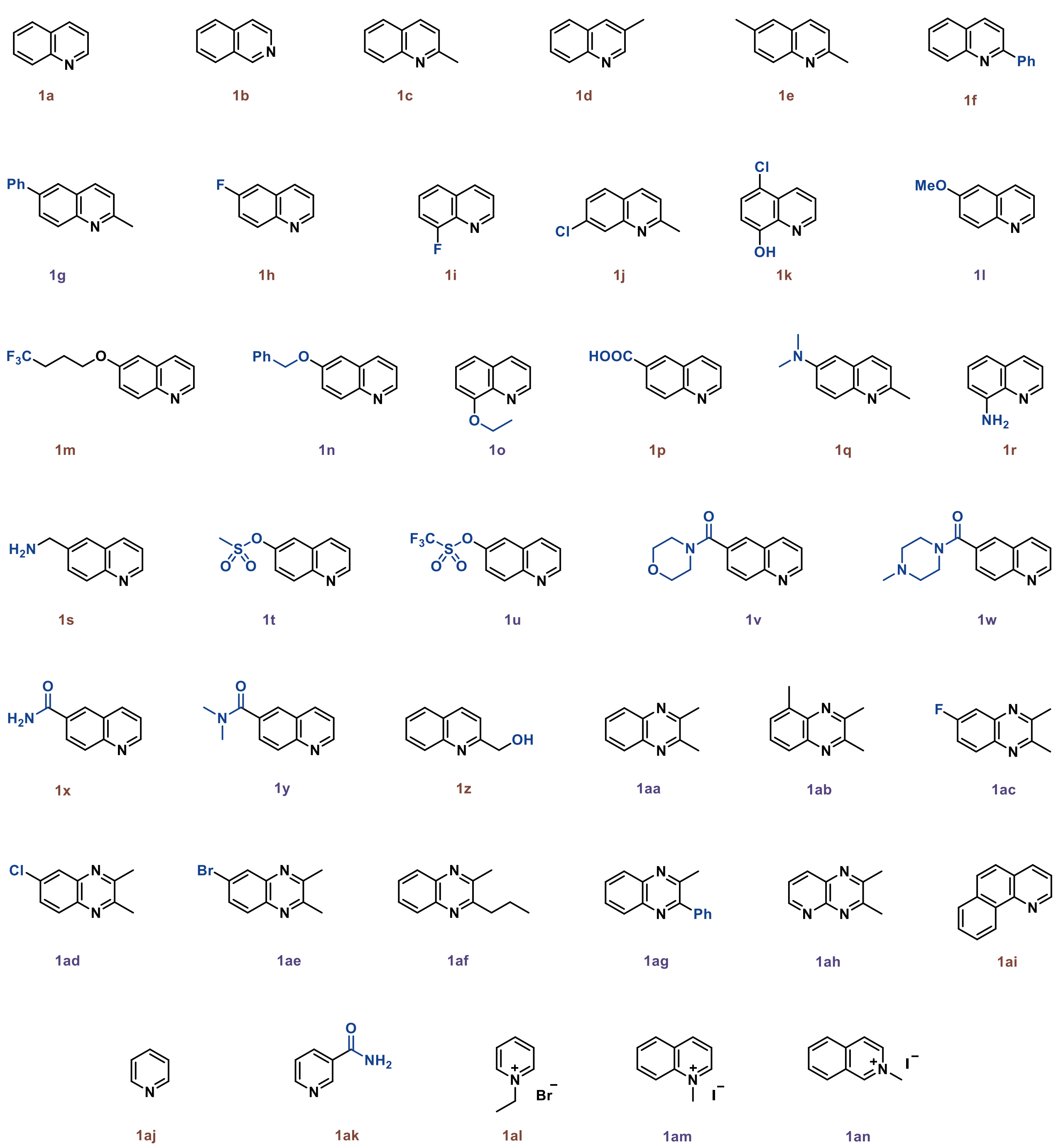

***Note I**: Compound **1g** is prepared following the literature report from Yin and coworkers.*[2]

Note II: *Compounds* ***1n*** *(with benzyl bromide as alkyl halide source) and* ***1o*** *(Bromoethane as alkyl halide source) are prepared following the literature report from Glorius and coworkers.* [3]

Note III: *Compound* ***1t*** *and* ***1u*** *are obtained following the protocol from Ritter's lab*[4] *and Liu's lab*[5] *respectively.*

6-(4,4,4-Trifluorobutoxy)quinoline (1l)

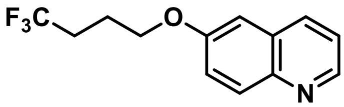

The title compound was synthesized using a modified literature protocol by Glorius and coworkers.[3] To an oven dried Schlenk tube equipped with a Teflon-coated magnetic stir bar were added 6-quinolinol (726 mg, 5.0 mmol, 1.0 equiv) and K_2CO_3 (920 mg, 6.7 mmol, 1.3 equiv.). The tube was then evacuated and backfilled with nitrogen thrice. Dry DMF (10 mL) was added, and the mixture was stirred before adding 1,1,1-trifluoro-4-iodobutane (1.4 equiv.). The reaction mixture was stirred at 80 °C overnight and then diluted with H_2O (10 mL). The aq. layer was diluted with brine and then extracted with EtOAc (3×40 mL). The combined org. layers were dried with $MgSO_4$, filtered, and the solvents were removed in vacuo. The crude was purified via column chromatography, giving the desired product (650 mg, 51%) as a brown solid.

R_f: 0.4 (80:20 pentane:diethyl ether)

^{1}H NMR (400 MHz, $CDCl_3$) δ 8.77 (dd, *J* = 4.3, 1.7 Hz, 1H), 8.07 – 7.99 (m, 2H), 7.40 – 7.31 (m, 2H), 7.05 (d, *J* = 2.8 Hz, 1H), 4.13 (t, *J* = 6.0 Hz, 2H), 2.44 – 2.28 (m, 2H), 2.18 – 2.06 (m, 2H).

^{13}C NMR (101 MHz, $CDCl_3$) δ 156.9, 148.0, 144.4, 135.1, 131.0, 129.4, 127.2 (q, *J* = 276.1 Hz), 122.5, 121.6, 106.1, 66.4, 30.9 (q, *J* = 29.3 Hz), 22.3 (q, *J* = 3.1 Hz).

^{19}F NMR (376 MHz, $CDCl_3$) δ -66.3 (t, *J* = 11.0 Hz).

GCMS(EI) m/z: calc'd for $C_{13}H_{12}F_3NO$ 255.0865; found 255.0866.

Note IV: *Compounds* ***1v, 1w***, *and* ***1y*** *are synthesized according to the general procedure reported by Zhou and coworkers*[6]

General procedure to prepare amides: To a suspension of 6-quinoline carboxylic acid (865 mg, 5 mmol) in dry CH_2Cl_2 (15 mL) was added oxalyl chloride (700 µL, 8.25 mmol) and DMF (5 drops). The reaction mixture was stirred at ambient temperature for 4 h. Then reaction mixture was cooled to 0 °C before adding triethylamine (2.1 mL, 15 mmol) and the amine source (5 equiv.) dropwise, and the reaction mixture was

stirred overnight at room temperature. The reaction was then quenched with water and organic materials were extracted twice with CH_2Cl_2. The combined organic layers were washed with saturated aqueous $NaHCO_3$ solution and then dried over $MgSO_4$. All the volatiles were removed under reduced pressure and the resulting crude mixture was purified by column chromatography to offer the desired amides.

A. 6-(Morpholine-4-carbonyl)quinoline (1v)

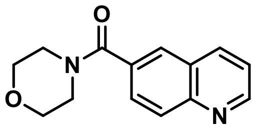

The title compound was synthesized using the general procedure mentioned above with morpholine as the aminating agent. *The analytical data matches with the literature precedence.*[7]

^{1}H NMR (400 MHz, DMSO) δ 8.96 (dd, *J* = 4.2, 1.7 Hz, 1H), 8.44 (dd, *J* = 8.4, 1.7 Hz, 1H), 8.11 – 8.04 (m, 2H), 7.77 (dd, *J* = 8.4, 1.7 Hz, 1H), 7.60 (dd, *J* = 8.4, 4.2 Hz, 1H), 3.83 – 3.22 (m, 8H).

^{13}C NMR (101 MHz, DMSO) δ 168.5, 151.7, 147.7, 136.6, 133.5, 129.3, 128.2, 127.3, 127.0, 122.2, 66.1, 47.8, 42.2.

***Note**: two aliphatic CH_2 carbon signals are merged into the signal at 66.1 ppm.*

B. 6-(1-Methylpiperazine-4-carbonyl)quinoline (1w)

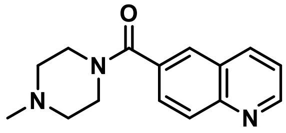

The title compound was synthesized using the general procedure mentioned above with 4-methyl piperazine as the aminating agent.

^{1}H NMR (400 MHz, CD_3OD) δ 8.9 (dd, *J* = 4.4, 1.7 Hz, 1H), 8.5 (ddd, *J* = 8.4, 1.7, 0.8 Hz, 1H), 8.1 (dd, *J* = 8.4, 0.8 Hz, 1H), 8.1 (d, *J* = 1.7 Hz, 1H), 7.8 (dd, *J* = 8.4, 1.7 Hz, 1H), 7.6 (dd, *J* = 8.4, 4.4 Hz, 1H), 3.8 (s, 4H), 2.6 – 2.4 (m, 4H), 2.3 (s, 3H).

^{13}C NMR (101 MHz, CD_3OD) δ 171.4, 152.7, 148.9, 138.9, 135.1, 129.9, 129.4, 129.4, 128.3, 123.5, 55.9, 55.5, 46.0, 43.0.

GCMS(EI) m/z: calc'd for $C_{15}H_{17}N_3O$ 255,1366; found 255,1367.

***Note**: two aliphatic CH_2 carbon signals are merged into the signal at 43.0 ppm.*

C. *N,N*-Dimethylquinoline-6-carboxamide (1y)

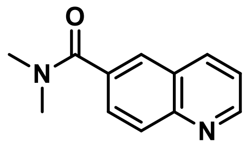

The title compound was synthesized using the general procedure mentioned above with dimethylamine as the aminating agent (without NEt_3). *The analytical data matches with the literature precedence*[6]

^{1}H NMR (400 MHz, CD_3OD) δ 8.93 (dd, *J* = 4.4, 1.7 Hz, 1H), 8.45 (ddd, *J* = 8.4, 1.7, 0.8 Hz, 1H), 8.11 (d, *J* = 8.4 Hz, 1H), 8.05 (d, *J* = 1.7 Hz, 1H), 7.82 (dd, *J* = 8.4, 1.7 Hz, 1H), 7.61 (dd, *J* = 8.4, 4.4 Hz, 1H), 3.17 (s, 3H), 3.05 (s, 3H).

^{13}C NMR (101 MHz, CD_3OD) δ 172.8, 152.6, 148.8, 138.9, 135.8, 129.7, 129.5, 129.3, 128.2, 123.4, 40.0, 35.7.

General procedure to prepare 2,3-dialkyl quinoxalines: To a stirred solution of 2,3-dione (13 mmol) in 50 mL of methanol were added benzene-1,2-diamine (10 mmol) and ammonium chloride (50 mol%). The reaction mixture was stirred at room temperature for 1 hour, then washed with water and extracted with dichloromethane (CH_2Cl_2). The organic phase was concentrated under vacuum and purified using column chromatography (90:10 pentane:diethyl ether).

Note V: Compounds **1ab–1ah** (known in literature) are synthesized according to the general procedure reported by Fan and coworkers[8] as mentioned above.

2.2 Screening and optimization in batch-type cells

- General protocol for screening and optimization studies in batch-type cells (***GP1***)

The reaction setup was chosen as shown in **Figure S1**. The anodic compartment was filled with 7 mL H_2O and the cathodic compartment was filled with 7 mL 3:1 MeOH:H_2O (*or* 1:1 acetone/H_2O mixture). Then H_2SO_4 (113 µL, 2.1 mmol, 2.0 eq.) was added to each compartment, followed by the addition of aza-arene (1.05 mmol) and additive (if any) into the catholyte. The reaction mixtures were stirred at high rpm for 2 mins for complete homogenization. The electrolysis started using DSA (IrO_x on Ti) as anode and nickel foam as cathode with 1 cm × 3 cm dipped inside the reaction mixtures. In a standard electrolysis experiment, 2432 C of electrical charge were applied under galvanostatic conditions at current density 50 mA/cm^2 (150 mA). After electrolysis, the catholyte was transferred into a 50 mL centrifuge tube along with the nickel foam electrode and internal standard (maleic acid). The catholyte was rinsed with MeOH. The centrifuge tube was then closed and shaken for approximately 2 minutes to ensure good homogenization of the reaction mixture "trapped" in a porous structure of nickel foam cathode. For ^{1}H NMR analysis, 150 µL of the prepared sample was transferred into a vial and dried under reduced pressure (75 mbar at 50 °C for 3 min). Then, the ^{1}H NMR was measured with *d6*-DMSO as the NMR solvent. The signal at 6.26 ppm was assigned to the two olefinic protons of maleic acid.

Optimization studies for quinoline 1a:

Table S1: *Screening of different acids (strong electrolyte).[a]*

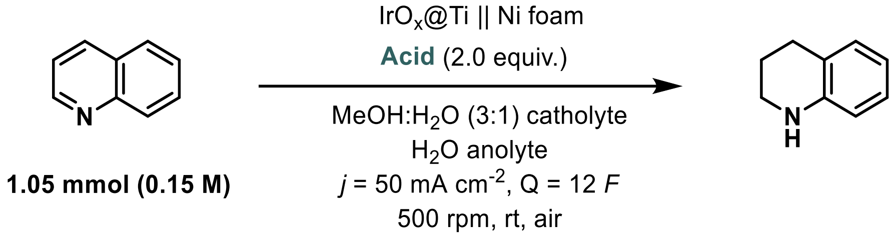

Entry	Acid	Yield (%)
**1**	H_2SO_4	**43**
**2**	*p*-TsOH·H_2O	26
**3**	CF_3SO_3H	22

^{1}H NMR yield using maleic acid as internal standard.

Table S2: *Optimization of acid equivalents.[a]*

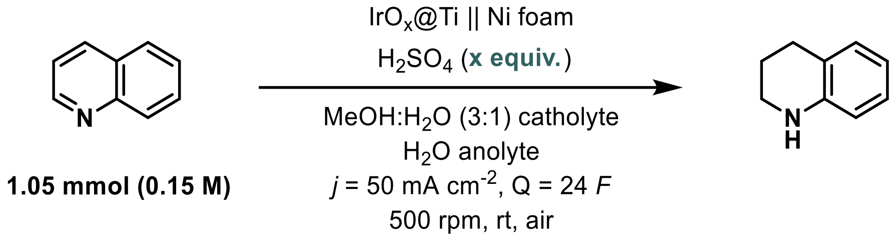

Entry	H_2SO_4 equivalent	Yield (%)
**1**	1.0*	12
**2**	2.0	**83**
**3**	3.0	58

**The Ni foam underwent degradation due to insufficient electrolyte volume and elevated applied voltage. Additionally, blue-coloured deposits were observed on the membrane surface. ^{1}H NMR yield using maleic acid as internal standard.*

Table S3: *Optimization of current density.*[a]

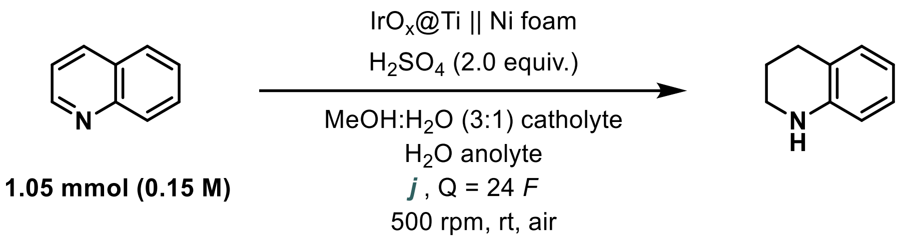

Entry	Current density (mA cm^{-2})	Yield (%)
**1**	25	62
**2**	50	**83**
**3**	75*	90

[*]A rise in reaction temperature caused Ni foam degradation. ^{1}H NMR yield using maleic acid as internal standard.

Table S4: *Optimization of solvent ratio.*[a]

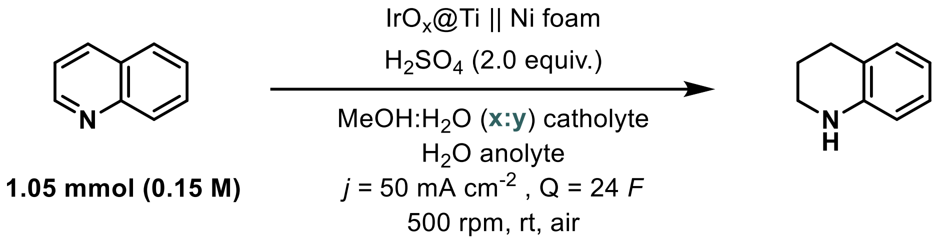

Entry	MeOH:H_2O (x:y)	Yield (%)
**1**	100:0	23
**2**	3:1	**83**
**3**	1:1	26
**4**	0:100	16

^{1}H NMR yield using maleic acid as internal standard.

Optimization studies for isoquinoline 1b:

Table S5: *Screening of different solvents.*[a]

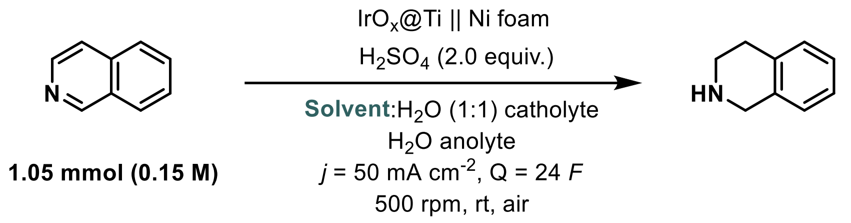

Entry	Solvent	Yield (%)	Nafion stability
**1**	H_2O	18	stable
**2**	HCOOH/H_2O (1:1)	22	stable
**3**	HFIP/H_2O (1:1)	11	stable
**4**	TFE/H_2O (1:1)	15	stable
**5**	Acetone/H_2O (1:1)	**45**	stable
**6**	MeOH/H_2O (1:1)	32	bright blue deposit
**7**	EtOH/H_2O (1:1)	36	stable
**8**	*n*PrOH/H_2O (1:1)	40	stable
**9**	*i*PrOH/H_2O (1:1)	43	stable
**10**	*n*BuOH/H_2O (1:1)	42	stable
**11**	*t*BuOH/H_2O (1:1)	37	stable
**12**	Ethylene glycol/H_2O (1:1)	30	stable

^{1}H NMR yield using maleic acid as internal standard.

Table S6: *Screening of acid equivalents.*[a]

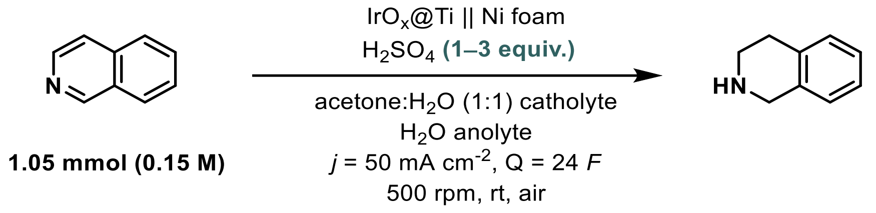

Entry	Acid equivalent	Yield (%)
**1**	1.0	40
**2**	2.0	**45**
**3**	3.0	39

^{1}H NMR yield using maleic acid as internal standard.

Table S7: *Screening of additional supporting electrolyte.*[a]

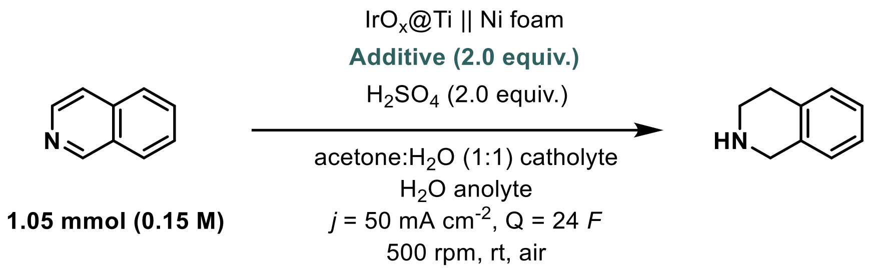

Entry	Additive (2.0 equiv.)	Yield (%)	Nafion stability
**1**	NH_4BF_4	64	stable
**2**	NEt_4BF_4	62	stable
**3**	NBu_4BF_4	**73**	stable
**4**	NBu_4Br	64	stable
**5**	$LiBF_4$	59	stable
**6**	$NaBF_4$	53	stable
**7**	LiCl	41	stable
**8**	LiBr	51	stable
**9**	LiI	72	Brown deposit
**10**	LiOTf	55	stable
**11**	NaI	62	Brown deposit
**12**	KI	42	Brown deposit
**13**	NBu_4I	54	Brown deposit
**14**	NPr_4Br	48	stable
**15**	NBu_4NO_3	31	stable

^{1}H NMR yield using maleic acid as internal standard.

Table S8: *Optimization of substrate concentration.*[a]

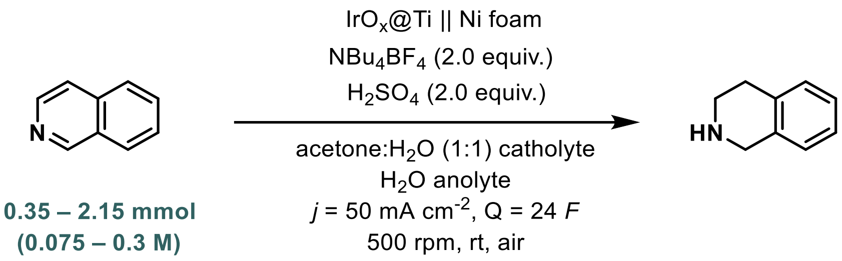

Entry	Concentration (M)	Yield (%)
**1**	0.075	63
**2**	0.15	**73**
**3**	0.3	70

^{1}H NMR yield using maleic acid as internal standard.

Table S9: *Optimization of reaction temperature.*[a]

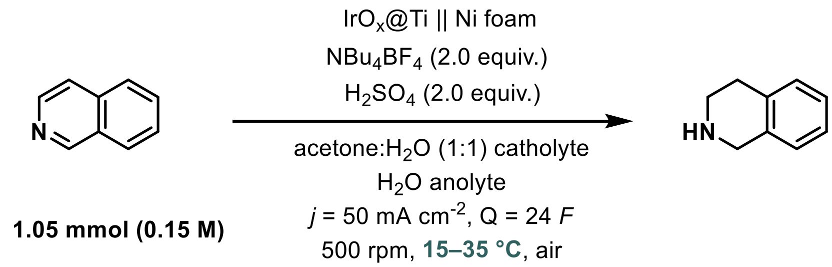

Entry	Temperature (°C)	Yield (%)
1	15	70
2	25	**73**
3	35	51

^{1}H NMR yield using maleic acid as internal standard.

Table S10: *Optimization of reaction stirring rate.*[a]

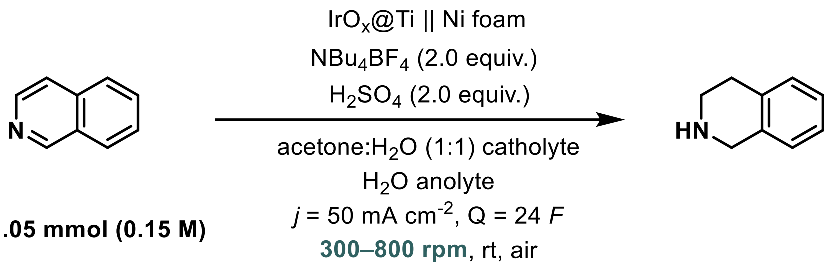

1.05 mmol (0.15 M)

Entry	Stirring rate (rpm)	Yield (%)
**1**	300	57
**2**	500	**73**
**3**	800	68

^{1}H NMR yield using maleic acid as internal standard.

Table S11: *Optimization of solvent ratio.*[a]

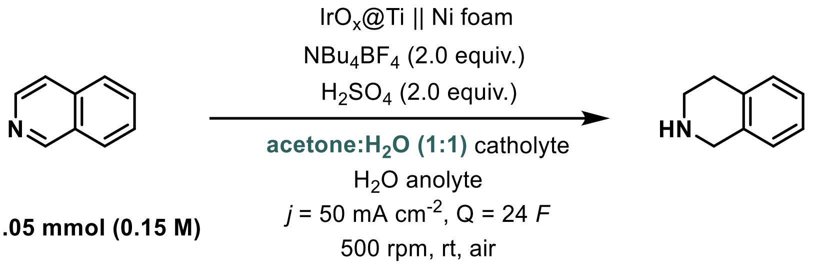

1.05 mmol (0.15 M)

Entry	Solvent ratio (acetone:H_2O)	Yield (%)
**1**	1:4	58
**2**	1:1	**73**
**3**	4:1	66

^{1}H NMR yield using maleic acid as internal standard.

Table S12: *Optimization of current density.*[a]

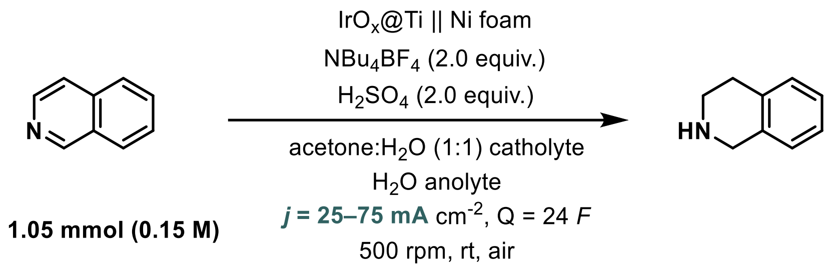

Entry	Current density (mA cm^{-2})	Yield (%)
**1**	25	64
**2**	50	**75**
**3**	75	61

^{1}H NMR yield using maleic acid as internal standard.

Table S13: *Optimization of additive equivalents.* [a]

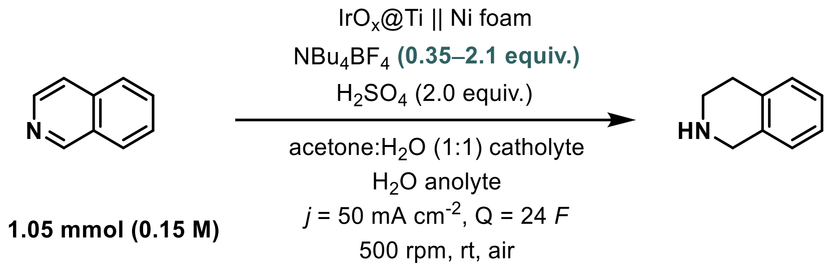

Entry	Equivalents	Concentration (M)	Yield (%)
**1**	0.35	0.05	63
**2**	0.7	0.1	52
**3**	1.05	0.15	61
**4**	1.5	0.2	86
**5**	2.1	0.3	76

^{1}H NMR yield using maleic acid as internal standard.

2.3 Deviation study

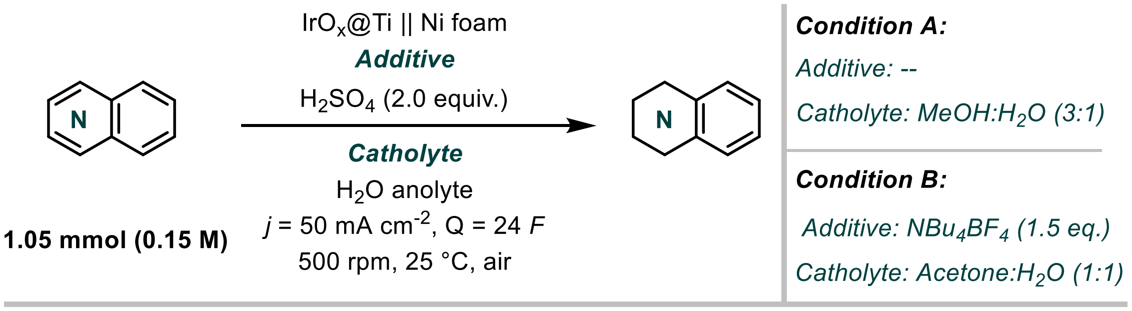

Entry	Deviations	% Yield 2a	% Yield 2b
**1**	**None**	**83 (81)**	**86 (83)**
**2**	*Condition **A***	83	39
**3**	*Condition **B***	50	86
**4**	Graphite as anode	70	67
**5**	Ni plate as cathode	30	55
**6**	Zinc plate as cathode	n.d.	9
**7**	CF_3SO_3H (0.6 M) instead of H_2SO_4	26	40
**8**	0.15 M of H_2SO_4	12	55
**9**	No additive	--	45
**10**	NBu_4I (1.5 eq.) instead of NBu_4BF_4	--	61
**11**	LiI (1.5 eq.) instead of NBu_4BF_4	--	55
**12**	MeOH:H_2O (1:1) in catholyte	26	54
**13**[b]	10 mA cm^{-2} instead of 50 mA cm^{-2}	21	34
**14**	Undivided cell	n.d.	18
**15**	1 atm H_2, without electricity	n.d.	n.d.

^{1}H NMR yields using maleic acid as internal standard. [b]The concentration changed over the long course of reaction. n.d.: not detected.

2.4 Sensitivity assessment

To check the reproducibility of the presented transformation, a series of sensitivity tests were conducted. These experiments were conducted with **1a** under *condition A*.

Table S14: *Sensitivity assessment.*[9]

Entry	Modification	Execution	Deviation (%)
**1**	High concentration (*c*)	0.3 M instead of 0.15 M	−13
**2**	Low concentration (*c*)	0.075 M instead of 0.15 M	−5
**3**	High current density (*j*)	75 mA cm^{-2}	+12
**4**	Low current density (*j*)	25 mA cm^{-2}	−25
**5**	High stirring rate (*rpm*)	300 rpm	+2
**6**	Low stirring rate (*rpm*)	800 rpm	−2
**7**	High temperature (*°C*)	35 °C	−7
**8**	Low temperature (*°C*)	15 °C	+1
**9**	High O_2	7 mL O_2 purged into both compartments	0
**10**	Low O_2	7 mL Ar purged into both compartments	−18

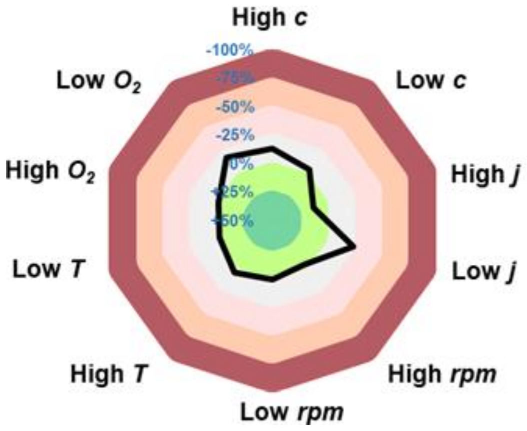

Figure S3: Graphical depiction of sensitivity assessment.

To check the reproducibility of the presented transformation, a series of sensitivity tests were conducted. These experiments were conducted with **1b** under *condition B*.

Table S15: *Sensitivity assessment.*[9]

Entry	Modification	Execution	Deviation (%)
**1**	High concentration (*c*)	0.3 M instead of 0.15 M	0
**2**	Low concentration (*c*)	0.075 M instead of 0.15 M	−5
**3**	High current density (*j*)	75 mA cm^{-2}	−9
**4**	Low current density (*j*)	25 mA cm^{-2}	−13
**5**	High stirring rate (*rpm*)	300 rpm	−5
**6**	Low stirring rate (*rpm*)	800 rpm	−4
**7**	High temperature (°*C*)	35 °C	−18
**8**	Low temperature (°*C*)	15 °C	+2
**9**	High O_2	7 mL O_2 purged into both compartments	0
**10**	Low O_2	7 mL Ar purged into both compartments	−10

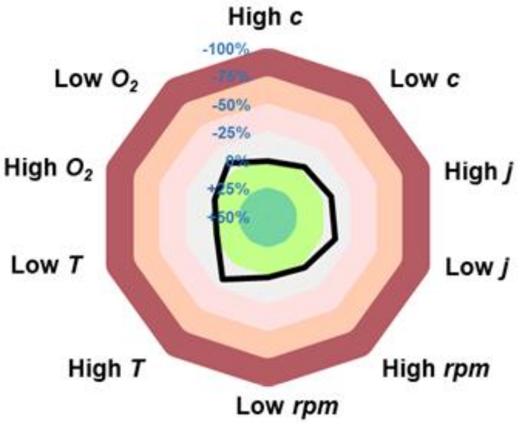

Figure S4: Graphical depiction of sensitivity assessment.

3 Scope

General protocol for preparative electrolysis in batch-type divided cell used in synthetic scope investigations on 1.05 mmol scale (GP2)

We used the reaction setup as described and shown in **Figure S1**. The anodic compartment was filled with 7 mL H_2O and the cathodic compartment was filled with 7 mL 3:1 MeOH/H_2O mixture (*condition A*) or 7 mL 1:1 acetone/H_2O mixture (*condition B*). Then H_2SO_4 (113 µL, 2.1 mmol, 2.0 equiv.) was added to each compartment, and in the catholyte additionally aza-arene substrate (1.05 mmol) was added. The reaction mixture was allowed to stir at elevated stirring rate for 2 mins for complete homogenization. The electrolysis was started using DSA (IrO_x on Ti) as anode and nickel foam as cathode. The current density was set to 50 mA/cm^2, and the amount of applied charge was set to 2432 C. The reaction was performed at room temperature and constant stirring rate 500 rpm. After electrolysis, the catholyte was transferred into a 50 mL centrifuge tube. The catholyte was rinsed two more times using ca. 5 mL MeOH per compartment. Also nickel foam electrode was excessively rinsed with MeOH to remove any "trapped" reaction mixture from a porous structure of nickel foam cathode. The reaction mixture was concentrated to remove the organic volatiles, followed by dilution with 10 mL water. Then, the reaction mixture was transferred to 100 mL separating flask for separation purposes. The aqueous phase containing protonated product and starting material was neutralized by NaOH until pH>13 as indicated by the pH paper. The basified reaction mixture was extracted three times with 20 mL Et_2O. Combined Et_2O fraction were washed with brine, dried over Na_2SO_4, concentrated under vacuum. The crude was purified using column chromatography.

***Note I**: Changes in the reaction condition are mentioned alongside the scope characterization data.*

***Note II**: In cases when the substrate didn't dissolve well in reaction solvent mixture MeOH/H_2O 3:1, we modified the procedure a bit by first dissolving the substrate in MeOH and then added H_2O gradually.*

2-Methyl-1,2,3,4-tetrahydroquinoline (2c)

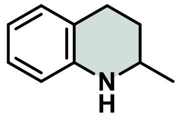

The title compound was synthesized with **1c** using GP-2 (*condition A)* on 1.05 mmol scale. *The analytical data matches with the literature precedence.[10]*

R$_f$: 0.7 (80:20 pentane:diethyl ether)

Yield: 137 mg, 90% (appearance: colorless oil)

^{1}**H NMR** (400 MHz, $CDCl_3$) δ 7.04 – 6.94 (m, 2H), 6.64 (td, *J* = 7.4, 1.2 Hz, 1H), 6.54 – 6.48 (m, 1H), 3.83 – 3.53 (m, 1H), 3.48 – 3.36 (m, 1H), 2.92 – 2.71 (m, 2H), 2.01 – 1.90 (m, 1H), 1.69 – 1.56 (m, 1H), 1.24 (d, *J* = 6.3 Hz, 3H).

^{13}**C NMR** (101 MHz, $CDCl_3$) δ 144.8, 129.4, 126.8, 121.3, 117.2, 114.2, 47.3, 30.2, 26.7, 22.7.

3-Methyl-1,2,3,4-tetrahydroquinoline (2d)

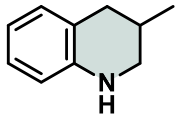

The title compound was synthesized with **1d** using GP-2 (*condition B)* on 1.05 mmol scale. *The analytical data matches with the literature precedence.[11]*

R$_f$: 0.7 (80:20 pentane:diethyl ether)

Yield: 95 mg, 62% (appearance: colorless oil)

^{1}**H NMR** (400 MHz, $CDCl_3$) δ 7.01 – 6.91 (m, 2H), 6.61 (td, *J* = 7.9, 1.2 Hz, 1H), 6.50 (dd, *J* = 7.9, 1.2 Hz, 1H), 3.27 (ddd, *J* = 11.0, 3.7, 2.0 Hz, 1H), 2.90 (dd, *J* = 11.0, 9.6 Hz, 1H), 2.78 (ddd, *J* = 16.0, 5.0, 2.0 Hz, 1H), 2.49 – 2.37 (m, 1H), 2.07 (ddd, *J* = 6.6, 5.0, 3.7 Hz, 1H), 1.05 (d, *J* = 6.6 Hz, 3H).

^{13}**C NMR** (101 MHz, $CDCl_3$) δ 144.3, 129.7, 126.8, 121.4, 117.2, 114.1, 49.0, 35.6, 27.3, 19.2.

2,6-Dimethyl-1,2,3,4-tetrahydroquinoline (2e)

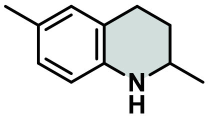

The title compound was synthesized with **1e** using GP-2 (*condition B)* on 1.05 mmol scale. *The analytical data matches with the literature precedence.[12]*

R$_f$: 0.7 (80:20 pentane:diethyl ether)

Yield: 148 mg, 88% (appearance: colorless oil)

^{1}**H NMR** (400 MHz, $CDCl_3$) δ 6.87 (dd, *J* = 7.6, 1.8 Hz, 2H), 6.52 – 6.46 (m, 1H), 3.63 (s, 1H), 3.44 (m, 1H), 2.96 – 2.84 (m, 1H), 2.78 (ddd, *J* = 16.4, 5.9, 3.1 Hz, 1H), 2.30 (s, 3H), 2.00 (ddt, *J* = 12.9, 5.9, 3.1 Hz, 1H), 1.67 (dddd, *J* = 12.9, 11.5, 10.0, 5.9 Hz, 1H), 1.28 (d, *J* = 5.9 Hz, 3H).

^{13}**C NMR** (101 MHz, $CDCl_3$) δ 142.5, 129.9, 127.3, 126.3, 121.3, 114.3, 47.4, 30.4, 26.6, 22.6, 20.5.

2-Phenyl-1,2,3,4-tetrahydroquinoline (2f)

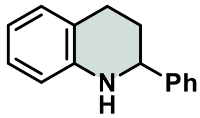

The title compound was synthesized with **1f** using GP-2 (*condition A with TfOH)* on 1.05 mmol scale. *The analytical data matches with the literature precedence.*[11]

R$_f$: 0.7 (90:10 pentane:diethyl ether)

Yield: 131 mg, 59% (appearance: pale yellow oil)

^{1}H NMR (400 MHz, CD_2Cl_2) δ 7.5 – 7.4 (m, 4H), 7.4 – 7.3 (m, 1H), 7.0 (ddd, J = 7.2, 4.1, 2.7 Hz, 2H), 6.7 (td, J = 7.2, 1.2 Hz, 1H), 6.6 – 6.5 (m, 1H), 4.5 (ddd, J = 9.2, 3.3, 1.2 Hz, 1H), 4.1 (s, 1H), 3.0 (ddd, J = 16.2, 10.5, 4.9 Hz, 1H), 2.8 (dt, J = 16.2, 4.9 Hz, 1H), 2.2 – 2.1 (m, 1H), 2.1 – 2.0 (m, 1H).

^{13}C NMR (101 MHz, CD_2Cl_2) δ 145.5, 145.3, 129.6, 128.9, 127.7, 127.2, 126.9, 121.4, 117.4, 114.3, 56.6, 31.5, 26.7.

2-Methyl-6-phenyl-1,2,3,4-tetrahydroquinoline (2g)

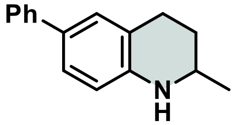

The title compound was synthesized with **1g** using GP-2 (*condition A with TfOH and 48 F)* on 1.05 mmol scale at 50 °C.

R$_f$: 0.8 (80:20 pentane:diethyl ether)

Yield: 103 mg, 44% (appearance: pale yellow solid)

^{1}H NMR (400 MHz, $CDCl_3$) δ 7.65 – 7.56 (m, 2H), 7.52 – 7.41 (m, 2H), 7.31 (ddt, J = 6.7, 5.6, 2.9 Hz, 3H), 6.63 – 6.58 (m, 1H), 3.90 – 3.63 (s, 1H), 3.55 – 3.43 (m, 1H), 3.00 – 2.81 (m, 2H), 2.02 (dddd, J = 12.8, 5.6, 3.6, 2.9 Hz, 1H), 1.69 (dddd, J = 12.8, 11.4, 9.3, 5.6 Hz, 1H), 1.29 (d, J = 6.3 Hz, 3H).

^{13}C NMR (101 MHz, $CDCl_3$) δ 144.3, 141.6, 130.1, 128.7, 128.0, 126.3, 126.0, 125.6, 121.4, 114.4, 47.4, 30.2, 26.8, 22.6.

GCMS(EI) m/z: calc'd for $C_{16}H_{17}N$ 223.1355; found 223.1358.

6-Fluoro-1,2,3,4-tetrahydroquinoline (2h)

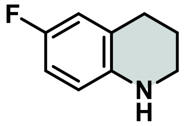

The title compound was synthesized with **1h** using GP-2 (*condition A)* on 1.05 mmol scale. *The characterization matches well with the reported data.*[10]

R$_f$: 0.7 (80:20 pentane:diethyl ether)

Yield: 147 mg, 93% (appearance: brown oil)

^{1}H NMR (400 MHz, $CDCl_3$) δ 6.75 – 6.61 (m, 2H), 6.42 (dd, *J* = 9.5, 4.9 Hz, 1H), 3.78 – 3.59 (m, 1H), 3.33 – 3.21 (m, 2H), 2.75 (t, *J* = 6.5 Hz, 2H), 1.99 – 1.88 (m, 2H).

^{13}C NMR (101 MHz, $CDCl_3$) δ 155.7 (d, *J* = 234.9 Hz), 140.8 (d, *J* = 1.8 Hz), 123.1 (d, *J* = 6.8 Hz), 115.7 (d, *J* = 22.3 Hz), 115.2 (d, *J* = 7.6 Hz), 113.3 (d, *J* = 22.3 Hz), 42.2, 27.1 (d, *J* = 1.8 Hz), 22.1.

^{19}F NMR (376 MHz, $CDCl_3$) δ -128.2.

8-Fluoro-1,2,3,4-tetrahydroquinoline (2i)

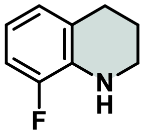

The title compound was synthesized with **1i** using GP-2 (*condition A with TfOH)* on 1.05 mmol scale. *The characterization matches well with the reported data.*[10]

R$_f$: 0.7 (80:20 pentane:diethyl ether)

Yield: 145 mg, 92% (appearance: colorless oil)

^{1}H NMR (400 MHz, $CDCl_3$) δ 6.86 – 6.71 (m, 2H), 6.52 (td, *J* = 7.8, 5.3 Hz, 1H), 4.00 (s, 1H), 3.40 – 3.30 (m, 2H), 2.80 (t, *J* = 6.4 Hz, 2H), 1.97 (dt, *J* = 11.2, 6.4 Hz, 2H).

^{13}C NMR (101 MHz, $CDCl_3$) δ 151.1 (d, *J* = 237.6 Hz), 133.2 (d, *J* = 12.2 Hz), 124.6 (d, *J* = 2.9 Hz), 123.7 (d, *J* = 3.7 Hz), 115.7 (d, *J* = 7.4 Hz), 112.2 (d, *J* = 18.2 Hz), 41.4, 26.7 (d, *J* = 2.9 Hz), 21.9.

^{19}F NMR (376 MHz, $CDCl_3$) δ -139.0.

7-Chloro-2-methyl-1,2,3,4-tetrahydroquinoline (2j)

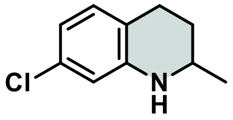

The title compound was synthesized with **1j** using GP-2 (*condition B)* on 1.05 mmol scale. *The analytical data matches with the literature precedence.*[10]

R$_f$: 0.8 (80:20 pentane:diethyl ether)

Yield: 161 mg, 85% (appearance: off-white solid)

^{1}H NMR (400 MHz, $CDCl_3$) δ 6.86 (dt, *J* = 8.0, 1.0 Hz, 1H), 6.56 (dd, *J* = 8.0, 2.1 Hz, 1H), 6.45 (d, *J* = 2.1 Hz, 1H), 3.96 – 3.55 (m, 1H), 3.40 (dqd, *J* = 9.4, 6.3, 3.0 Hz, 1H), 2.83 – 2.64 (m, 2H), 1.93 (m, 1H), 1.63 – 1.51 (m, 1H), 1.22 (d, *J* = 6.3 Hz, 3H).

^{13}C NMR (101 MHz, $CDCl_3$) δ 145.7, 132.0, 130.3, 119.5, 116.8, 113.5, 47.1, 29.8, 26.2, 22.5.

GCMS(EI) m/z: calc'd (isotope ^{35}Cl) for $C_{10}H_{12}ClN$ 181.0654; found 181.0657.

5-Chloro-8-hydroxy-1,2,3,4-tetrahydroquinoline (2k)

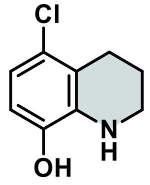

The title compound was synthesized with **1k** using GP-2 (*condition A with TfOH)* on 1.05 mmol scale. *The analytical data matches with the literature precedence.[13]*

R$_f$: 0.4 (40:60 pentane:diethyl ether)

Yield: 119 mg, 62% (appearance: brown oil)

^{1}H NMR (400 MHz, CD_3CN) δ 6.52 (d, *J* = 8.4 Hz, 1H), 6.45 (d, *J* = 8.4 Hz, 1H), 3.28 – 3.20 (m, 2H), 2.70 (t, *J* = 6.5 Hz, 2H), 1.91 – 1.84 (m, 2H).

^{13}C NMR (101 MHz, CD_3CN) δ 142.7, 136.6, 125.9, 119.9, 116.1, 113.2, 41.4, 25.5, 22.5.

HRMS(ESI) m/z: calc'd (isotope ^{35}Cl) for [$C_9H_{10}ClNO$ + H] 184.0524; found 184.0526.

6-Methoxy-1,2,3,4-tetrahydroquinoline (2l)

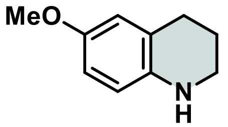

The title compound was synthesized with **1l** using GP-2 (*condition B)* on 1.05 mmol scale. *The analytical data matches with the literature precedence.[10]*

R$_f$: 0.4 (80:20 pentane:diethyl ether)

Yield: 104 mg, 61%

^{1}**H NMR** (400 MHz, $CDCl_3$) δ 6.64 – 6.55 (m, 2H), 6.48 (d, J = 8.5 Hz, 1H), 3.73 (s, 3H), 3.68 – 3.45 (m, 1H), 3.30 – 3.22 (m, 2H), 2.76 (t, J = 6.5 Hz, 2H), 2.00 – 1.88 (m, 2H).

^{13}**C NMR** (101 MHz, $CDCl_3$) δ 152.2, 138.5, 123.3, 116.0, 115.0, 113.0, 56.0, 42.5, 27.2, 22.5.

6-(4,4,4-Trifluorobutoxy)-1,2,3,4-tetrahydroquinoline (2m)

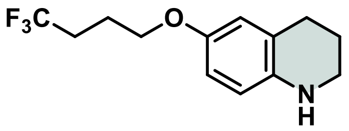

The title compound was synthesized with **1m** using GP-2 (*condition A)* on 1.05 mmol scale at 50 °C.

R$_f$: 0.5 (80:20 pentane:diethyl ether)

Yield: 171 mg, 63% (appearance: pale brown oil)

^{1}**H NMR** (400 MHz, $CDCl_3$) δ 6.63 – 6.56 (m, 2H), 6.46 (d, J = 8.4 Hz, 1H), 3.93 (t, J = 6.0 Hz, 2H), 3.55 (s, 1H), 3.31 – 3.22 (m, 2H), 2.76 (t, J = 6.5 Hz, 2H), 2.41 – 2.20 (m, 2H), 2.06 – 1.89 (m, 4H).

^{13}**C NMR** (101 MHz, $CDCl_3$) δ 151.0, 139.1, 127.4 (q, J = 276.0 Hz), 123.2, 116.1, 115.7, 113.9, 67.0, 42.4, 30.9 (q, J = 29.1 Hz), 27.2, 22.5.

^{19}**F NMR** (376 MHz, $CDCl_3$) δ -66.3.

GCMS(EI) m/z: calc'd for $C_{13}H_{16}F_3NO$ 259.1178; found 259.1181.

6-(Benzyloxy)-1,2,3,4-tetrahydroquinoline (2n)

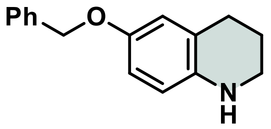

The title compound was synthesized with **1n** using GP-2 (*condition A)* on 1.05 mmol scale at 50 °C.

R$_f$: 0.5 (80:20 pentane:diethyl ether)

Yield: 163 mg, 66% (appearance: brown oil)

^{1}**H NMR** (400 MHz, CD_2Cl_2) δ 7.44 – 7.28 (m, 5H), 6.66 – 6.56 (m, 2H), 6.40 (d, J = 9.4 Hz, 1H), 4.95 (s, 2H), 3.28 – 3.19 (m, 2H), 2.72 (t, J = 6.5 Hz, 2H), 1.95 – 1.85 (m, 2H).

^{13}**C NMR** (101 MHz, CD_2Cl_2) δ 151.2, 139.9, 138.5, 128.8, 128.1, 127.9, 123.1, 116.5, 115.5, 114.3, 71.1, 42.7, 27.6, 22.9.

GCMS(EI) m/z: calc'd for $C_{16}H_{17}NO$ 239.1304; found 239.1304.

8-Ethoxy-1,2,3,4-tetrahydroquinoline (2o)

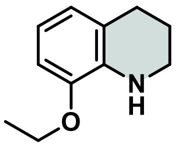

The title compound was synthesized with **1o** using GP-2 (*condition A)* on 1.05 mmol scale. *The analytical data matches with the literature precedence.* [14]

R$_f$: 0.5 (80:20 pentane:diethyl ether)

Yield: 167 mg, 90% (appearance: yellow oil)

^{1}H NMR (400 MHz, $CDCl_3$) δ 6.70 – 6.55 (m, 3H), 4.07 (q, J = 7.0 Hz, 2H), 3.41 – 3.33 (m, 2H), 2.82 (t, J = 6.4 Hz, 2H), 2.07 – 1.95 (m, 2H), 1.46 (t, J = 7.0 Hz, 3H).

^{13}C NMR (101 MHz, $CDCl_3$) δ 145.7, 134.6, 121.6, 121.5, 115.8, 108.4, 63.6, 41.6, 26.8, 22.2, 15.1.

Methyl 1,2,3,4-tetrahydroquinoline-6-carboxylate (2p)

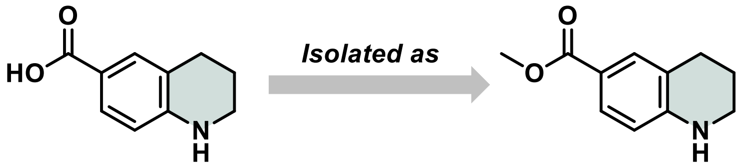

The title compound was synthesized on a 1.05 mmol scale using **1p** and GP-2 (*condition A)*, employing 100% MeOH as solvent in the catholyte. *The analytical data matches with the literature precedence.*[15]

R$_f$: 0.4 (90:10 pentane:diethyl ether)

Yield: 192 mg, 96% (appearance: yellow solid)

^{1}H NMR (400 MHz, CD_2Cl_2) δ 7.64 – 7.55 (m, 2H), 6.44 – 6.37 (m, 1H), 4.47 (s, 1H), 3.80 (s, 3H), 3.37 – 3.29 (m, 2H), 2.76 (t, J = 6.3 Hz, 2H), 1.94 – 1.87 (m, 2H).

^{13}C NMR (101 MHz, CD_2Cl_2) δ 167.6, 149.4, 131.4, 129.2, 120.4, 117.6, 112.8, 51.6, 42.0, 27.3, 21.8.

6-*N,N*-dimethylamino-1,2,3,4-tetrahydroquinoline (2q)

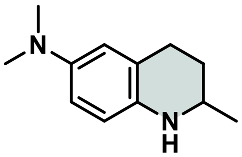

The title compound was synthesized with **1q** using GP-2 (*condition A with TfOH and 48 F)* on 1.05 mmol scale at 50 °C.

R$_f$: 0.2 (96:4 CH_2Cl_2:MeOH)

Yield: 139 mg, 70% (appearance: brown oil)

^{1}H NMR (400 MHz, CD_2Cl_2) δ 6.44 (m, 3H), 3.03 (m, 10H), 1.97 – 1.84 (m, 1H), 1.54 (ddd, J = 17.1, 8.8, 3.9 Hz, 1H), 1.17 (d, J = 6.3 Hz, 3H).

^{13}C NMR (101 MHz, CD_2Cl_2) δ 144.4, 138.0, 122.6, 116.0, 115.6, 114.2, 48.0, 42.5, 31.1, 27.5, 22.8.

GCMS(EI) m/z: calc'd for $C_{12}H_{18}N_2$ 190.1464; found 190.1466.

8-Amino-1,2,3,4-tetrahydroquinoline (2r)

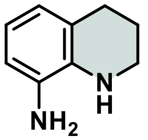

The title compound was synthesized with **1r** using GP-2 (*condition A with TfOH and 48 F)* on 1.05 mmol scale. *The analytical data matches with the literature precedence.*[16]

R$_f$: 0.2 (100% Ethyl acetate)

Yield: 120 mg, 77% (appearance: brown oil)

^{1}H NMR (400 MHz, CD_2Cl_2) δ 6.54 – 6.46 (m, 3H), 3.45 – 3.17 (m, 5H), 2.75 (t, J = 6.4 Hz, 2H), 1.92 – 1.85 (m, 2H).

^{13}C NMR (101 MHz, CD_2Cl_2) δ 134.4, 134.2, 123.4, 121.2, 118.1, 114.1, 42.9, 27.5, 22.9.

6-Aminomethyl-1,2,3,4-tetrahydroquinoline (2s)

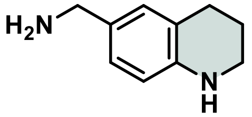

The title compound was synthesized with **1s** using GP-2 (*condition B and 48 F)* on 1.05 mmol scale.

R$_f$: 0.2 (93:7 CH_2Cl_2:MeOH)

Yield: 155 mg, 91% (appearance: pale brown solid)

^{1}**H NMR** (400 MHz, CD_2Cl_2) δ 6.85 (d, *J* = 6.8 Hz, 2H), 6.44 – 6.36 (m, 1H), 3.64 (s, 2H), 3.33 – 3.23 (m, 2H), 2.73 (t, *J* = 6.4 Hz, 2H), 2.66 – 1.29 (m, 5H).

^{13}**C NMR** (101 MHz, CD_2Cl_2) δ 144.1, 132.4, 128.7, 125.9, 121.7, 114.3, 46.4, 42.3, 27.3, 22.7.

HRMS(ESI) m/z: calc'd for [$C_{10}H_{14}N_2$ + Na] 185.1049; found 185.1049.

6-(Methylsulfonyloxy)-1,2,3,4-tetrahydroquinoline (2t)

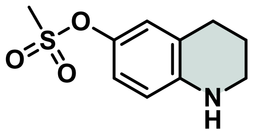

The title compound was synthesized with **1t** using GP-2 (*condition A)* on 1.05 mmol scale at 50 °C.

$\mathbf{R_f}$: 0.6 (70:30 pentane:diethyl ether)

Yield: 193 mg, 81% (appearance: yellow oil)

^{1}**H NMR** (400 MHz, $CDCl_3$) δ 6.92 – 6.81 (m, 2H), 6.42 (dd, *J* = 8.0, 1.0 Hz, 1H), 3.97 – 3.57 (s, 1H), 3.35 – 3.22 (m, 2H), 3.06 (s, 3H), 2.74 (t, *J* = 6.4 Hz, 2H), 1.91 (m, 2H).

^{13}**C NMR** (101 MHz, $CDCl_3$) δ 143.9, 140.1, 122.8, 122.5, 120.3, 114.5, 41.9, 36.8, 27.1, 21.6.

GCMS(EI) m/z: calc'd for $C_{10}H_{13}NO_3S$ 227.0610; found 227.0613.

6-(1,1,1-Trifluoromethanesulfonyloxy)-1,2,3,4-tetrahydroquinoline (2u)

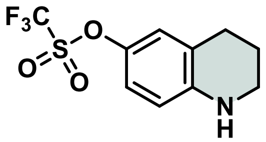

The title compound was synthesized with **1u** using GP-2 (*condition A with TfOH)* on 1.05 mmol scale at 50 °C.

$\mathbf{R_f}$: 0.6 (70:30 pentane:diethyl ether)

Yield: 171 mg, 58% (appearance: pale yellow solid)

^{1}**H NMR** (400 MHz, CD_2Cl_2) δ 6.91 – 6.80 (m, 2H), 6.43 (dt, *J* = 8.6, 1.2 Hz, 1H), 4.05 (s, 1H), 3.34 – 3.27 (m, 2H), 2.75 (t, *J* = 6.4 Hz, 2H), 1.96 – 1.87 (m, 2H).

^{13}**C NMR** (101 MHz, CD_2Cl_2) δ 145.3, 140.5, 122.6, 122.2, 119.8, 118.8 (q, *J* = 320.9 Hz), 114.2, 42.0, 27.4, 21.7.

^{19}**F NMR** (376 MHz, CD_2Cl_2) δ -73.4.

GCMS(EI) m/z: calc'd for $C_{10}H_{10}F_3NO_3S$ 281.0328; found 281.0329.

6-(Morpholine-4-carbonyl)-1,2,3,4-tetrahydroquinoline (2v)

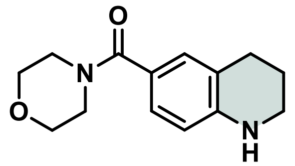

The title compound was synthesized with **1v** using GP-2 (*condition A)* on 1.05 mmol scale at 50 °C.

R_f: 0.4 (100% diethyl ether)

Yield: 181 mg, 70% (appearance: colorless oil)

^{1}H NMR (400 MHz, $CDCl_3$) δ 7.06 (d, *J* = 2.1 Hz, 1H), 7.01 (dd, *J* = 8.2, 2.1 Hz, 1H), 6.39 (d, *J* = 8.2 Hz, 1H), 3.75 – 3.55 (m, 9H), 3.33 – 3.26 (m, 2H), 2.72 (t, *J* = 6.3 Hz, 2H), 1.95 – 1.85 (m, 2H).

^{13}C NMR (101 MHz, $CDCl_3$) δ 171.4, 146.4, 129.6, 126.9, 122.7, 120.9, 113.1, 67.1, 46.0, 41.8, 27.0, 21.6.

GCMS(EI) m/z: calc'd for $C_{14}H_{18}N_2O_2$ 246.1363; found 246.1364.

6-(1-Methylpiperazine-4-carbonyl)-1,2,3,4-tetrahydroquinoline (2w)

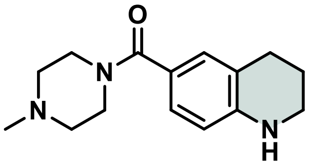

The title compound was synthesized with **1w** using GP-2 (*condition A)* on 1.05 mmol scale at 50 °C.

R_f: 0.3 (85:15 CH_2Cl_2:MeOH)

Yield: 168 mg, 62% (appearance: colorless oil)

^{1}H NMR (400 MHz, CD_2Cl_2) δ 7.08 – 6.95 (m, 2H), 6.40 (d, *J* = 8.1 Hz, 1H), 4.21 (s, 1H), 3.70 – 3.60 (m, 4H), 3.36 – 3.26 (m, 2H), 2.74 (t, *J* = 6.3 Hz, 2H), 2.51 (t, *J* = 5.1 Hz, 4H), 2.38 (s, 3H), 1.91 (qdd, *J* = 6.3, 4.1, 2.7 Hz, 2H).

^{13}C NMR (101 MHz, CD_2Cl_2) δ 171.3, 147.1, 129.8, 127.2, 123.1, 120.9, 113.0, 55.4, 45.9, 45.1, 42.2, 27.4, 22.1.

GCMS(EI) m/z: calc'd for $C_{15}H_{21}N_3O$ 259.1679; found 259.1679.

1,2,3,4-Tetrahydroquinoline-6-carboxamide (2x)

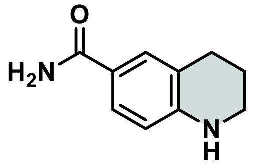

The title compound was synthesized with **1x** using GP-2 (*condition A with TfOH)* on 1.05 mmol scale. *The analytical data matches with the literature precedence.[15] The absence of N-H protons could be attributed to the exchange with MeOD.*

R$_f$: 0.5 (95:5 CH_2Cl_2:MeOH)

Yield: 79 mg, 43% (appearance: brown solid)

^{1}H NMR (400 MHz, MeOD) δ 7.48 – 7.42 (m, 2H), 6.43 (d, J = 9.0 Hz, 1H), 3.32 – 3.25 (m, 3H), 2.73 (t, J = 6.3 Hz, 2H), 1.88 (dtd, J = 9.0, 6.3, 5.0 Hz, 2H).

^{13}C NMR (101 MHz, MeOD) δ 173.0, 150.3, 130.2, 128.1, 120.8, 120.5, 113.6, 42.4, 28.2, 22.6.

***N,N*-Dimethyl-1,2,3,4-tetrahydroquinoline-6-carboxamide (2y)**

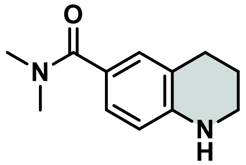

The title compound was synthesized with **1y** using GP-2 (*condition A)* on 1.05 mmol scale at 50 °C.

R$_f$: 0.5 (100% EtOAc)

Yield: 172 mg, 80% (appearance: colorless oil)

^{1}H NMR (400 MHz, CD_2Cl_2) δ 7.10 – 6.96 (m, 2H), 6.39 (d, J = 8.1 Hz, 1H), 4.38 (s, 1H), 3.35 – 3.23 (m, 2H), 3.01 (s, 6H), 2.73 (t, J = 6.4 Hz, 2H), 2.00 – 1.83 (m, 2H).

^{13}C NMR (101 MHz, CD_2Cl_2) δ 172.3, 146.8, 129.6, 127.0, 123.9, 120.5, 112.9, 42.1, 39.0, 27.4, 22.2.

GCMS(EI) m/z: calc'd for $C_{12}H_{16}N_2O$ 204.1257; found 204.1258.

2-Hydroxymethyl-1,2,3,4-tetrahydroquinoline (2z)

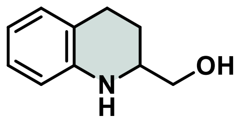

The title compound was synthesized with **1z** using GP-2 (*condition B)* on 1.05 mmol scale. *The analytical data matches with the literature precedence.[17]*

R$_f$: 0.3 (60:40 pentane:diethyl ether)

Yield: 159 mg, 93% (appearance: colorless oil)

^{1}H NMR (400 MHz, $CDCl_3$) δ 7.03 – 6.93 (m, 2H), 6.66 (td, J = 7.9, 1.2 Hz, 1H), 6.57 (dd, J = 7.9, 1.2 Hz, 1H), 3.75 (dd, J = 10.5, 3.8 Hz, 1H), 3.57 (dd, J = 10.5, 7.8 Hz, 1H), 3.45 (m, 1H), 3.21 (s, 2H), 2.94 – 2.67 (m, 2H), 1.97 – 1.86 (m, 1H), 1.72 (m, 1H).

^{13}C NMR (101 MHz, $CDCl_3$) δ 143.9, 129.4, 127.0, 121.8, 117.9, 114.9, 66.7, 53.0, 26.0, 24.4.

2,3-Dimethyl-1,2,3,4-tetrahydroquinoxaline (2aa)

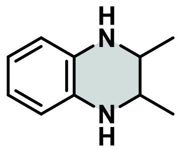

The title compound was synthesized with **1aa** using GP-2 (*condition A and 12 F)* on 1.05 mmol scale. *The analytical data matches with the literature precedence.*[18]

R$_f$: 0.5 (95:5 pentane:diethyl ether)

Yield: 141 mg, 83%; *d.r.* = 94:6 (major diastereomer: *cis*); (appearance: pale brown solid)

^{1}H NMR (400 MHz, CD_2Cl_2) δ 6.56 – 6.51 (m, 2H), 6.49 – 6.44 (m, 2H), 3.71 – 3.50 (m, 2H), 3.50 – 3.43 (m, 2H), 1.12 (d, J = 6.4 Hz, 6H).

^{13}C NMR (101 MHz, CD_2Cl_2) δ 133.2, 118.6, 114.5, 49.4, 17.4.

2,3,5-Trimethyl-1,2,3,4-tetrahydroquinoxaline (2ab)

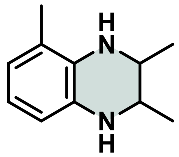

The title compound was synthesized with **1ab** using GP-2 (*condition A and 12 F)* on 1.05 mmol scale. *The analytical data matches with the literature precedence.*[19]

R$_f$: 0.5 (95:5 pentane:diethyl ether)

Yield: 160 mg, 87%; *d.r.* = 93:7 (major diastereomer: *cis*); (appearance: pale brown solid)

^{1}H NMR (400 MHz, CD_2Cl_2) δ 6.50 (d, J = 5.7 Hz, 2H), 6.39 (dd, J = 5.9, 3.4 Hz, 1H), 3.64 – 3.34 (m, 4H), 2.11 (s, 3H), 1.17 (d, J = 6.5 Hz, 3H), 1.13 (d, J = 6.5 Hz, 3H).

^{13}C NMR (101 MHz, CD_2Cl_2) δ 132.7, 131.1, 121.9, 120.3, 118.0, 112.7, 49.8, 49.2, 17.7, 17.3, 17.1.

6-Fluoro-1,2,3,4-tetrahydro-2,3-dimethylquinoxaline (2ac)

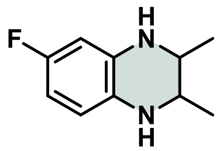

The title compound was synthesized with **1ac** using GP-2 (*condition A and 12 F)* on 1.05 mmol scale. *The analytical data matches with the literature precedence.[19]*

R$_f$: 0.5 (85:15 pentane:diethyl ether)

Yield: 180 mg, 95%; *d.r.* = >95:5 (major diastereomer: *cis*); (appearance: pale yellow solid)

^{1}H NMR (400 MHz, CD_2Cl_2) δ 6.40 – 6.33 (m, 1H), 6.24 – 6.16 (m, 2H), 3.82 – 3.19 (m, 4H), 1.11 – 1.06 (m, 6H).

^{13}C NMR (101 MHz, CD_2Cl_2) δ 157.2 (d, *J* = 232.5 Hz), 134.5 (d, *J* = 10.2 Hz), 129.1 (d, *J* = 1.8 Hz), 114.7 (d, *J* = 9.4 Hz), 103.6 (d, *J* = 22.5 Hz), 100.9 (d, *J* = 25.9 Hz), 49.5, 49.3, 17.2.

^{19}F NMR (376 MHz, CD_2Cl_2) δ -127.1.

6-Chloro-1,2,3,4-tetrahydro-2,3-dimethylquinoxaline (2ad)

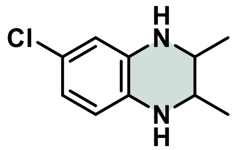

The title compound was synthesized with **1ad** using GP-2 (*condition A and 12 F)* on 1.05 mmol scale. *The analytical data matches with the literature precedence.[19]*

R$_f$: 0.5 (95:5 pentane:diethyl ether)

Yield: 169 mg, 82%; *d.r.* = >95:5 (major diastereomer: *cis*); (appearance: off-white solid)

^{1}H NMR (400 MHz, CD_3CN) δ 6.42 – 6.37 (m, 2H), 6.35 (d, *J* = 8.8 Hz, 1H), 4.36 (s, 2H), 3.43 – 3.30 (m, 2H), 1.03 (s, 3H), 1.02 (s, 3H).

^{13}C NMR (101 MHz, CD_3CN) δ 135.6, 133.0, 122.4, 117.5, 115.1, 113.5, 49.4, 49.3, 17.2, 17.1.

HRMS(ESI) m/z: calc'd (isotope ^{35}Cl) for [$C_{10}H_{13}ClN_2$ + H] 197.0841; found 197.0839.

6-Bromo-1,2,3,4-tetrahydro-2,3-dimethylquinoxaline (2ae)

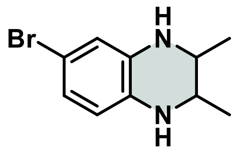

The title compound was synthesized with **1ae** using GP-2 (*condition A and 12 F)* on 1.05 mmol scale. *The analytical data matches with the literature precedence.[19]*

R_f: 0.7 (95:5 pentane:diethyl ether)

Yield: 189 mg, 75%; *d.r.* = >95:5 (major diastereomer: *cis*); (appearance: brown solid)

^{1}H NMR (400 MHz, CD_2Cl_2) δ 6.60 (dd, J = 8.3, 2.2 Hz, 1H), 6.56 (d, J = 2.2 Hz, 1H), 6.33 (d, J = 8.3 Hz, 1H), 3.69 (s, 2H), 3.47 – 3.40 (m, 2H), 1.11 – 1.07 (m, 6H).

^{13}C NMR (101 MHz, CD_2Cl_2) δ 134.8, 132.3, 120.7, 116.4, 115.4, 109.9, 49.3, 49.2, 17.3, 17.2.

HRMS(ESI) m/z: calc'd (isotope ^{79}Br) for [$C_{10}H_{13}BrN_2$ + H] 241.0332; found 241.0335.

2-Methyl-3-propyl-1,2,3,4-tetrahydroquinoxaline (2af)

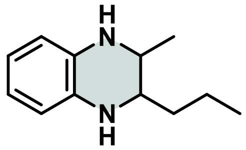

The title compound was synthesized with **1af** using GP-2 (*condition A and 12 F)* on 1.05 mmol scale. *The analytical data matches with the literature precedence.[19]*

R_f: 0.8 (90:10 pentane:diethyl ether)

^{1}H NMR Yield: 155 mg, 78%; *d.r.* = >95:5 (major diastereomer: *cis*);

^{1}H NMR (400 MHz, CD_2Cl_2) δ 6.60 – 6.58 (m, 2H), 6.50 – 6.49 (m, 2H), 3.64 (m, 2H), 3.51 – 3.49 (m, 1H), 3.36 – 3.33 (m, 1H), 1.51 – 1.35 (m, 4H), 1.15 (d, J = 6.5 Hz, 3H), 0.96 (t, J = 7.0 Hz, 3H).

^{13}C NMR (101 MHz, CD_2Cl_2) δ 133.0, 132.7, 118.6, 118.6, 114.5, 114.4, 53.3, 48.7, 33.4, 19.3, 17.0, 14.4.

2-Methyl-3-phenyl-1,2,3,4-tetrahydroquinoxaline (2ag)

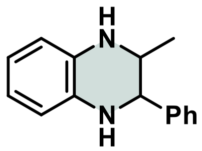

The title compound was synthesized with **1ag** using GP-2 (*condition A and 12 F)* on 1.05 mmol scale. *The analytical data matches with the literature precedence.[19]*

R_f: 0.8 (90:10 pentane:diethyl ether)

Yield: 200 mg, 85%; *d.r.* = >95:5 (major diastereomer: *cis*); (appearance: colorless oil)

^{1}H NMR (400 MHz, CD_2Cl_2) δ 7.42 – 7.28 (m, 5H), 6.72 – 6.63 (m, 2H), 6.61 – 6.54 (m, 2H), 4.52 (d, J = 3.2 Hz, 1H), 3.73 (qd, J = 6.5, 3.2 Hz, 1H), 0.97 (d, J = 6.5 Hz, 3H).

^{13}C NMR (101 MHz, CD_2Cl_2) δ 142.5, 133.6, 133.0, 128.5, 128.0, 127.6, 119.3, 118.6, 114.9, 114.1, 58.8, 49.8, 17.8.

2,3-Dimethyl-1,2,3,4-tetrahydropyrido[2,3-*b*]pyrazine (2ah)

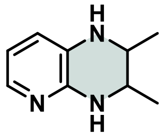

The title compound was synthesized with **1ah** using GP-2 (*condition A)* on 1.05 mmol scale. *The analytical data matches with the literature precedence.[20]*

^{1}H NMR Yield: 44%; *d.r.* = >95:5 (major diastereomer: *cis*)

^{1}H NMR (400 MHz, $CDCl_3$) δ 8.53 (d, *J* = 5.0 Hz, 1H), 7.62 (d, *J* = 7.7 Hz, 1H), 7.30 (d, *J* = 7.7 Hz, 1H), 3.70 (brs, 2H), 3.60 – 3.54 (m, 2H), 1.20 (m, 6H).

^{13}C NMR (101 MHz, $CDCl_3$) 151.0, 141.0, 139.3, 115.7, 115.0, 48.9, 48.8, 17.4, 17.2.

1,2,3,4-Tetrahydrobenzo[*h*]quinoline (2ai)

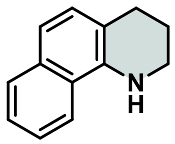

The title compound was synthesized with **1ai** using GP-2 (*condition A)* on 1.05 mmol scale. *The analytical data matches with the literature precedence.[10]*

R$_f$: 0.7 (95:5 pentane:diethyl ether)

^{1}H NMR Yield: 41%;

^{1}H NMR (400 MHz, CD_2Cl_2) δ 7.71 (d, *J* = 7.7 Hz, 1H), 7.64 (d, *J* = 7.7 Hz, 1H), 7.40 – 7.31 (m, 2H), 7.13 (d, *J* = 8.2 Hz, 1H), 7.11 (d, *J* = 8.2 Hz, 1H), 3.46 (t, *J* = 5.5 Hz, 2H), 2.91 (t, *J* = 6.4 Hz, 2H), 2.07 – 1.94 (m, 2H);

^{13}C NMR (101 MHz, CD_2Cl_2) 139.3, 133.1, 128.7, 128.6, 124.9, 124.7, 123.5, 119.1, 116.7, 115.8, 42.5, 25.6, 22.0.

1-Tosylpiperidine-3-carboxamide (2ak)

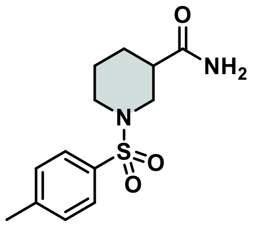

The title compound was synthesized with **1ak** using GP-2 (*condition A and 72 F)* on 1.05 mmol scale. *The hydrogenated nicotinamide was isolated as tosyl-protected compound due to the poor separation issues of the former with the starting material.*

R$_f$: 0.65 (90:10 CH_2Cl_2:MeOH)

Yield: 124 mg, 42% (combined yield of two steps); (appearance: off-white solid)

^{1}H NMR (400 MHz, DMSO) δ 7.65 – 7.57 (m, 2H), 7.50 – 7.42 (m, 2H), 7.42 (brs, 1H), 6.92 (brs, 1H), 3.65 – 3.48 (m, 2H), 2.41 (s, 3H), 2.38 – 2.30 (m, 1H), 2.17 – 2.03 (m, 2H), 1.79 – 1.67 (m, 2H), 1.51 – 1.36 (m, 1H), 1.28 – 1.13 (m, 1H);

^{13}C NMR (101 MHz, DMSO) δ 174.2, 143.6, 132.2, 129.9, 127.5, 48.3, 46.1, 41.3, 26.5, 23.8, 21.0.

HRMS(ESI) m/z: calc'd for [$C_{13}H_{18}N_2O_3S$+Na] 305.0930; found 305.0929.

1-Methyl-1,2,3,4-tetrahydroquinoline (2am)

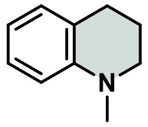

The title compound was synthesized with the corresponding iodide salt **(1am)** using GP-2 (*condition A)* on 1.05 mmol scale at 50 °C. *The analytical data matches with the literature precedence.[21]*

R$_f$: 0.7 (90:10 pentane:diethyl ether)

Yield: 109 mg, 70%; (appearance: colorless liquid)

^{1}H NMR (400 MHz, CD_2Cl_2) δ 7.11 – 7.02 (m, 1H), 7.00 – 6.92 (m, 1H), 6.63 – 6.57 (m, 2H), 3.28 – 3.20 (m, 2H), 2.91 (s, 3H), 2.79 (t, *J* = 6.5 Hz, 2H), 2.06 – 1.96 (m, 2H).

^{13}C NMR (101 MHz, CD_2Cl_2) δ 147.4, 129.1, 127.4, 123.4, 116.5, 111.3, 51.7, 39.3, 28.3, 23.0.

2-Methyl-1,2,3,4-tetrahydroisoquinoline (2an)

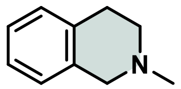

The title compound was synthesized with the corresponding iodide salt using GP-2 (*condition A)* on 1.05 mmol scale. *The analytical data matches with the literature precedence.[22]*

R$_f$: 0.2 (100% EtOAc)

Yield: 88 mg, 57%; (appearance: colorless solid)

^{1}H NMR (400 MHz, CD_2Cl_2) δ 7.37 – 7.25 (m, 2H), 7.24 (dd, *J* = 7.5, 1.6 Hz, 1H), 7.15 (dd, *J* = 7.2, 1.9 Hz, 1H), 4.29 (s, 2H), 3.46 (t, *J* = 6.4 Hz, 2H), 3.32 (t, *J* = 6.4 Hz, 2H), 2.89 (s, 3H).

^{13}C NMR (101 MHz, CD_2Cl_2) δ 130.8, 129.3, 129.0, 127.8, 127.1, 126.8, 54.5, 51.6, 42.7, 25.1.

- ***Faradaic efficiencies with batch-type cells***

Scope	F.E (%)
**2a**	13.5
**2b**	13.9
**2c**	15.0
**2d**	10.4
**2e**	14.7
**2f**	9.9
**2g**	3.7
**2h**	15.5
**2i**	15.4
**2j**	14.2
**2k**	10.4
**2l**	10.2
**2m**	10.5
**2n**	11.0
**2o**	15.0
**2p**	16.0
**2q**	5.8
**2r**	6.4
**2s**	7.6
**2t**	13.5
**2u**	9.7
**2v**	11.7
**2w**	10.4
**2x**	7.2
**2y**	13.4
**2z**	15.2
**2aa**	27.4
**2ab**	28.7
**2ac**	31.4
**2ad**	27.1
**2ae**	24.8
**2af**	25.7
**2ag**	28.0
**2ah**	7.3
**2ai**	6.8
**2aj**	5.6
**2ak**	3.5
**2al**	6.7
**2am**	12.9
**2an**	9.5
**2ao**	12.0

4 Synthetic applications

The versatility of this protocol has been demonstrated through the synthesis of medicinally important molecules, thereby augmenting their synthetic utility.

1. 1-(Morpholine-4-carbonyl)-1,2,3,4-tetrahydroquinoline (3a)

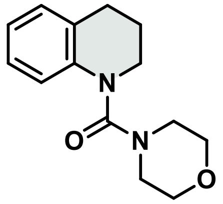

The title compound was synthesized starting from **2a** using an established literature procedure on 2 mmol scale. *The characterization data matches with the literature report.[23]*

R_f: 0.3 (20:80 pentane:diethyl ether)

Yield: 432 mg, 88%; (appearance: colorless oil)

^{1}H NMR (400 MHz, CD_2Cl_2) δ 7.15 – 7.04 (m, 3H), 6.96 – 6.88 (m, 1H), 3.65 – 3.52 (m, 6H), 3.35 – 3.24 (m, 4H), 2.76 (t, *J* = 6.7 Hz, 2H), 1.99 – 1.89 (m, 2H).

^{13}C NMR (101 MHz, CD_2Cl_2) δ 160.2, 141.0, 129.3, 128.1, 126.5, 122.2, 120.2, 66.7, 46.5, 45.9, 27.2, 23.7.

2. 2-Methyl-1-((4-(trifluoromethyl)phenyl)sulfonyl)-1,2,3,4-tetrahydroquinoline (3b)

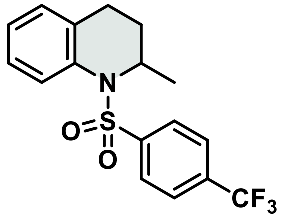

The title compound was synthesized starting from **2c** using an established literature procedure on 2 mmol scale. *The characterization data matches with the literature report.[24]*

R_f: 0.6 (90:10 pentane:diethyl ether)

Yield: 640 mg, 90%; (appearance: colorless oil)

^{1}H NMR (400 MHz, CD_2Cl_2) δ 7.7 – 7.6 (m, 5H), 7.3 – 7.2 (m, 1H), 7.2 (td, *J* = 7.5, 1.3 Hz, 1H), 7.1 – 7.0 (m, 1H), 4.43 – 4.32 (m, , 1H), 2.46 – 2.33 (m, 1H), 1.8 (m, 1H), 1.8 – 1.7 (m, 1H), 1.4 – 1.3 (m, 1H), 1.3 (d, *J* = 6.5 Hz, 3H).

^{13}C NMR (101 MHz, CD_2Cl_2) δ 143.2, 135.0, 134.3 (q, *J* = 32.9 Hz), 134.2, 128.6, 127.9, 127.6, 127.1, 126.4 (q, *J* = 3.7 Hz), 126.4, 123.8 (q, *J* = 272.8 Hz), 53.3, 30.6, 25.0, 21.8.

^{19}F NMR (376 MHz, CD_2Cl_2) δ -63.4.

3. 1-((4-Fluorophenyl)sulfonyl)-2-methyl-1,2,3,4-tetrahydroquinoline (3c)

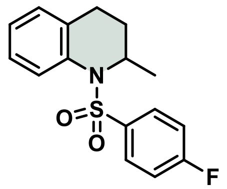

The title compound was synthesized starting from **2c** using an established literature procedure on 2 mmol scale. *The characterization data matches with the literature report.*[24]

R$_f$: 0.6 (90:10 pentane:diethyl ether)

Yield: 536 mg, 88%; (appearance: colorless solid)

^{1}H NMR (400 MHz, CD_2Cl_2) δ 7.68 (dd, J = 8.1, 1.2 Hz, 1H), 7.51 – 7.43 (m, 2H), 7.28 – 7.20 (m, 1H), 7.12 (td, J = 7.5, 1.3 Hz, 1H), 7.10 – 7.03 (m, 2H), 7.02 – 6.98 (m, J = 7.5, 1.4 Hz, 1H), 4.40 – 4.28 (m, 1H), 2.46 – 2.36 (m, 1H), 1.88 – 1.70 (m, 2H), 1.42 – 1.28 (m, 1H), 1.26 (d, J = 6.5 Hz, 3H).

^{13}C NMR (101 MHz, CD_2Cl_2) δ 165.5 (d, J = 253.8 Hz), 135.7 (d, J = 3.2 Hz), 135.3, 134.2, 130.0, 129.9, 128.5, 127.7, 127.0, 126.1, 116.5, 116.3, 53.0, 30.5, 25.0, 21.8.

^{19}F NMR (376 MHz, CD_2Cl_2) δ -106.25.

4. Aspernigerin (3d)

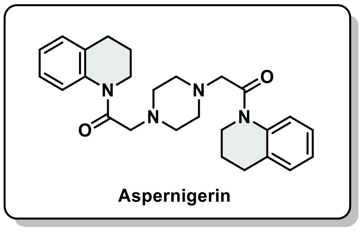

Synthetic route:

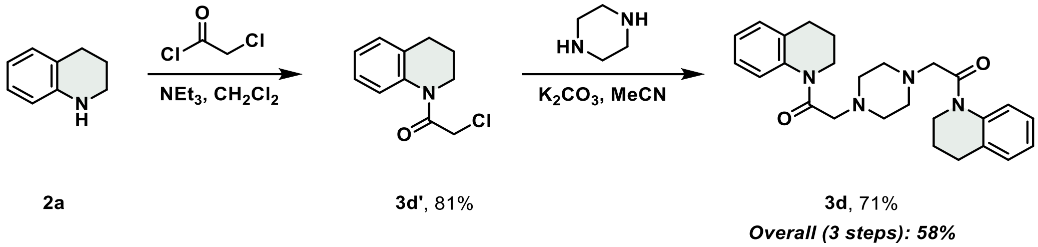

1-(Chloroacetyl)-1,2,3,4-tetrahydroquinoline (3d’)

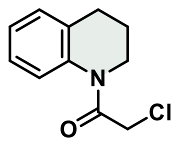

The title compound was synthesized using quinoline (**2a**) and chloroacetylchloride on 2 mmol scale using modified literature procedure. *The characterization data matches with the literature report.*[25]

R$_f$: 0.65 (50:50 pentane:diethyl ether)

Yield: 338 mg, 81%; (appearance: dark yellow oil)

^{1}H NMR (400 MHz, CD_2Cl_2) δ 7.69 – 6.94 (m, 4H), 4.25 (s, 2H), 3.78 (t, J = 6.6 Hz, 2H), 2.74 (t, J = 6.6 Hz, 2H), 1.98 (p, J = 6.6 Hz, 2H).

^{13}C NMR (101 MHz, CD_2Cl_2) δ 166.0, 138.5, 134.3, 129.0, 126.6, 126.1, 124.0, 44.1, 42.6, 26.9, 24.1.

GCMS(EI) m/z: calc'd (isotope ^{35}Cl) for [$C_{11}H_{12}ClNO$] 209.0607; found 209.0610.

Aspernigerin (3d)

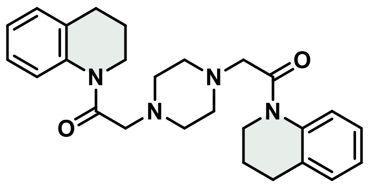

The title compound was synthesized starting from **3d'** using an established literature procedure on 0.65 mmol scale. *The characterization data matches with the literature report.[24]*

R$_f$: 0.65 (90:10 CH_2Cl_2:MeOH)

Yield: 199 mg, 71% (appearance: pale brown solid)

^{1}H NMR (400 MHz, CD_2Cl_2) δ 7.75 – 7.04 (m, 8H), 3.77 (t, J = 6.8 Hz, 4H), 3.26 (s, 4H), 2.73 (t, J = 6.8 Hz, 4H), 2.53 (s, 8H), 1.95 (p, J = 6.6 Hz, 4H).

^{13}C NMR (101 MHz, CD_2Cl_2) δ 169.1, 139.3, 128.7, 126.0, 125.1, 124.7, 61.2, 53.2, 43.9, 27.0, 24.3.

5. (±)-Angustureine (3e)

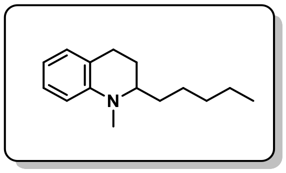

(±)–Angustureine

Anti-malarial, cytotoxic

Synthetic route:

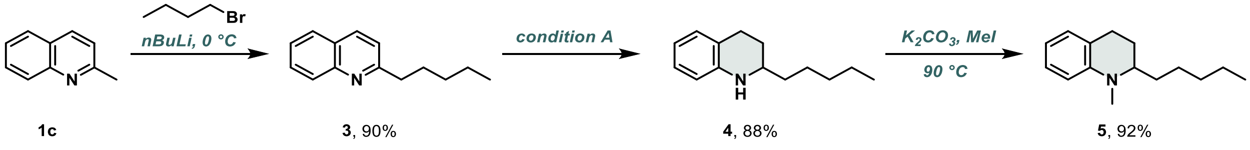

2-Pentylquinoline (3e')

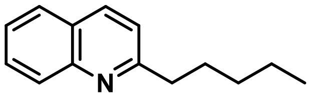

The title compound was synthesized using quinaldine (**1c**) and bromobutane on 10 mmol scale using modified literature procedure. *The characterization data matches with the literature report.*[25]

R$_f$: 0.6 (80:20 pentane:diethyl ether)

Yield: 1.8 g, 90%; (appearance: yellow oil)

^{1}H NMR (400 MHz, CD_2Cl_2) δ 8.06 (d, J = 8.4 Hz, 2H), 7.76 (dd, J = 8.1, 1.5 Hz, 1H), 7.67 (ddd, J = 8.4, 6.8, 1.5 Hz, 1H), 7.47 (ddd, J = 8.1, 6.8, 1.5 Hz, 1H), 7.33 – 7.25 (m, 1H), 3.01 – 2.93 (m, 2H), 1.89 – 1.74 (m, 2H), 1.48 – 1.30 (m, 4H), 0.97 – 0.83 (m, 3H).

^{13}C NMR (101 MHz, CD_2Cl_2) δ 144.7, 129.1, 126.6, 121.1, 116.9, 114.0, 51.7, 36.4, 31.9, 28.2, 26.4, 25.3, 22.7, 14.0.

2-Pentyl-1,2,3,4-tetrahydroquinoline (3e'')

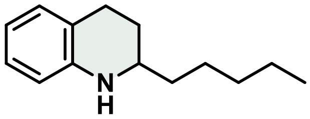

The title compound was synthesized starting from **3e'** using GP-2 (*condition A)* on 1.05 mmol scale at 50 °C. *The characterization data matches with the literature report.*[11]

R$_f$: 0.7 (80:20 pentane:diethyl ether)

Yield: 188 mg, 88%; (appearance: colorless oil)

^{1}H NMR (400 MHz, CD_2Cl_2) δ 6.96 – 6.87 (m, 2H), 6.55 (td, J = 7.4, 1.2 Hz, 1H), 6.48 – 6.41 (m, 1H), 3.82 (s, 1H), 3.28 – 3.16 (m, 1H), 2.87 – 2.66 (m, 2H), 1.97 (m, 1H), 1.66 – 1.27 (m, 9H), 0.93 (t, J = 6.7 Hz, 3H).

^{13}C NMR (101 MHz, CD_2Cl_2) δ 145.4, 129.5, 126.9, 121.7, 117.0, 114.2, 52.0, 37.1, 32.4, 28.5, 26.8, 25.8, 23.1, 14.3.

1-Methyl-2-pentyl-1,2,3,4-tetrahydroquinoline (3e)

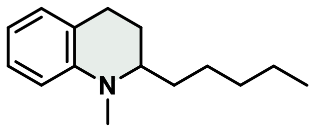

The title compound was synthesized starting from **3e''** using an established literature procedure on 2 mmol scale. *The characterization data matches with the literature report.*[26]

R_f: 0.6 (92:8 pentane:diethyl ether)

Yield: 400 mg, 92%;

^{1}H NMR (400 MHz, CD_2Cl_2) δ 7.06 – 6.97 (m, 1H), 6.96 – 6.86 (m, 1H), 6.58 – 6.41 (m, 2H), 3.23 (tt, *J* = 8.6, 4.2 Hz, 1H), 2.91 (s, 3H), 2.84 – 2.71 (m, 1H), 2.69 – 2.57 (ddd, *J* = 16.3, 5.1, 3.4 Hz, 1H), 1.99 – 1.77 (m, 2H), 1.63 – 1.54 (m, 1H), 1.41 – 1.25 (m, 7H), 0.90 (t, *J* = 6.9 Hz, 3H).

^{13}C NMR (101 MHz, CD_2Cl_2) δ 145.9, 128.8, 127.3, 122.3, 115.4, 110.7, 59.3, 38.1, 32.4, 31.4, 26.1, 24.8, 23.9, 23.1, 14.2.

5 Flow experiments

- ***Scale-up of hydrogenation of 2,3-dimethylquinoxaline (1aa) to 25-gram scale***

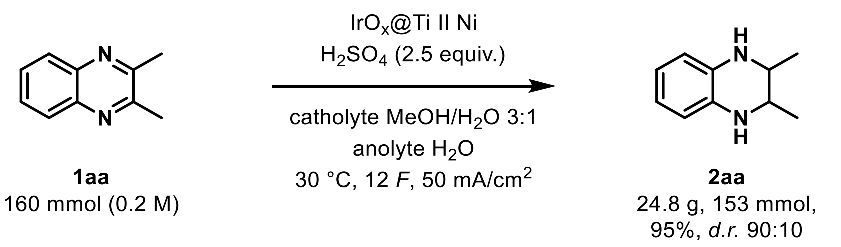

We used the reaction setup as described and shown in **Figure S2** and **Figure S5**. Two 1 L Schott bottles were filled with 800 mL H_2O (anolyte) and 800 mL 3:1 MeOH/H_2O mixture (catholyte) separately. Then H_2SO_4 (21.4 mL, 400 mmol, 2.5 equiv.) was slowly added to each flask while stirring and cooling, and in the catholyte additionally 2,3-dimethylquinoxaline **1aa** (25.3 g, 160 mmol, 1.0 equiv.) was added. The flasks were connected to the flow cell and electrolysis was started. For electrolysis, 185251 C amount of applied charge (12 *F*) were applied under galvanostatic conditions at current density of 50 mA/cm^2 (t = 21.5 h) at 30 °C. After the set amount of electric charge has passed, both compartments were immediately emptied to prevent any diffusion of the product from catholyte into the anolyte. Nickel foam in the cathodic compartment was rinsed by pumping 50 mL of MeOH through the cell. Then, methanol was removed from the collected reaction mixture under reduced pressure by evaporation at 100 mbar at 50 °C for 15 min. The aqueous phase containing protonated product was then basified. The basification was carried out by slowly adding concentrated aq. NaOH while stirring and cooling the reaction mixture on ice and frequently monitoring pH with indicator papers. The addition of base was stopped once the pH reached >13. Additional 100 mL of H_2O was added to dilute and transfer the neutralized reaction mixture into a separatory funnel. The aqueous layer was extracted two times with 200 mL Et_2O. The combined Et_2O fractions were dried over Na_2SO_4. The drying agent was filtered, and the solvent was evaporated to obtain a beige solid which still contained some water. The crude product was dried by evaporation of water with toluene and two evaporations with acetonitrile. After additional drying using a rotary evaporator (6 mbar at 50 °C) we obtained 24.8 g of 2,3-dimethyl-1,2,3,4-tetrahydroquinoxaline (**2aa**) as a beige powder (153 mmol, 95% in 90:10 *d.r.*). The purity was confirmed by ^{1}H NMR.

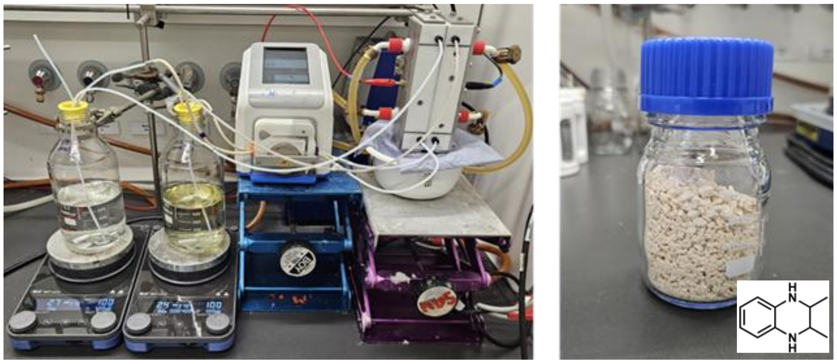

Figure S5: Flow electrolyser during operation (*left*); obtained product **2aa** (25 g, in a 100 mL Schott bottle (*right*).

Current-Voltage profile: The performance was also analysed with the stability in the current-voltage profile and it looks stable throughout the entire electrolysis period.[27]

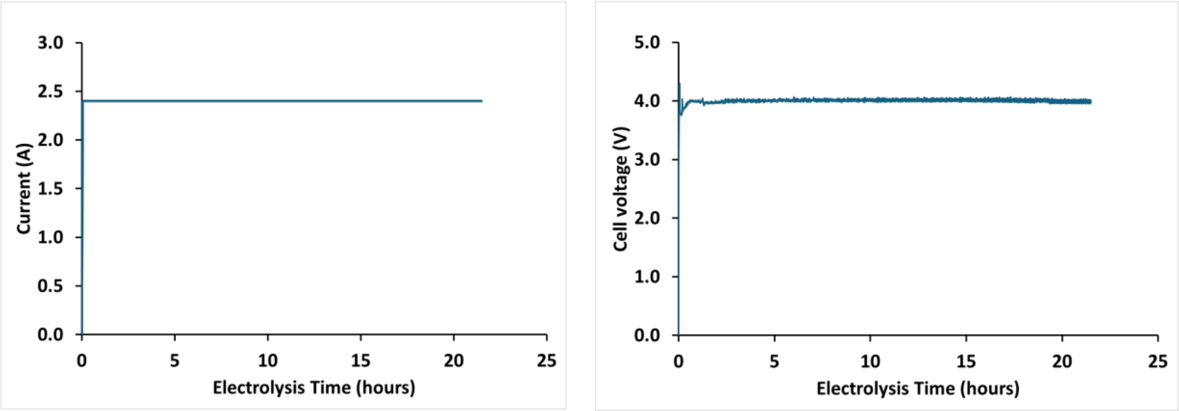

***Productivity**:* The overall productivity (normalized) and the energy requirement of the large-scale reaction were calculated:

Parameter	Value	Unit
Product amount	0.153	mol
Molar mass	162	g mol^{-1}
Current	2.4	A
Cell voltage (V)	4.0	V
Current density	50	mA cm^{-2}
Total Volume (V_{Total})	821.4	mL or 0.8214 L
Flow rate	25	mL min^{-1} = 1.5 L h^{-1}
Run time	21.5	h
Electrode area	48	cm^2
Theoretical e^-	4	F mol^{-1}
Experimental e^-	12	F mol^{-1}

Metric	Formula	Result	Units
Theoretical faradaic efficiency	$\frac{4}{12} \times 100$	33 %	—
Experimental faradaic efficiency	$\frac{4}{12} \times yield$	31.4 %	—
Space–time yield (reactor-based)	$\frac{n}{V \times t}$	0.00866	mol L^{-1} h^{-1}
	---	1.40	g L^{-1} h^{-1}
Area-normalized productivity	$\frac{n}{A \times t}$	1.48×10^{-4}	mol cm^{-2} h^{-1}
	---	1.48	mol m^{-2} h^{-1}
Charge normalized	$\frac{n}{I \times t}$	0.00297	mol $A^{-1}h^{-1}$
Energy consumption	$\frac{V \times I \times t}{n}$	4.86 MJ mol^{-1}	(≈ 1.35 kWh mol^{-1})
Energy-normalized productivity	$\frac{n \times 1000}{V \times I \times t}$	0.74	mol kWh^{-1}

- ***Scale-up of hydrogenation of 2,3-dimethylquinoxaline (1aa) to 2-gram scale***

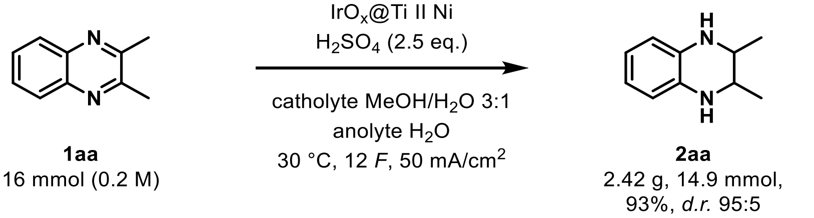

The product was obtained follow the same procedure as described above for 25-gram scale reaction with proportionally lower quantities of the solvents. We prepared catholyte in 100 mL Schott bottle with 2,3-dimethylquinoxaline **1aa** (2.53 g, 16 mmol, 1.0 equiv.) and H_2SO_4 (2.14 mL, 40 mmol, 2.5 eq.) in 80 mL MeOH:H_2O (3:1), whereas the anolyte was prepared by mixing H_2SO_4 (2.14 mL, 40 mmol, 2.5 eq.) in 80 mL H_2O. The electrolysis was performed at 30 °C passing 18525 C of charge (12 *F*) at current density of 50 mA/cm^2 which corresponds to 2.4 A in our 48 cm^2 electroylser (t = 2.1 h). After the electrolysis, we followed previously described isolation procedure (for 160 mmol scale reaction) and obtained 2.42 g of the desired product **2aa** (14.9 mmol, 93%, *d.r.* 95:5).

- ***Upscale of hydrogenation of isoquinoline (1b) to 2-gram scale***

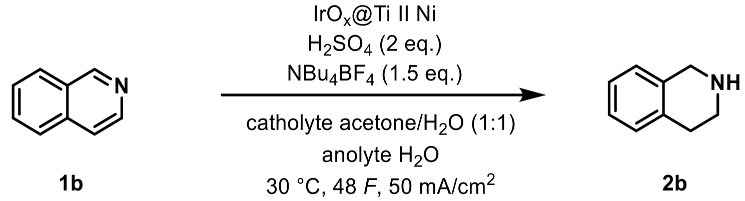

1b
20 mmol (0.2 M)

2b
2.39 g, 17.9 mmol, 89%

We used the reaction setup as described and shown in **Figure S5**. A 250 mL Schott bottle was filled with 100 mL H_2O (anolyte) and another 250 mL Schott bottle was filled with 100 mL 1:1 acetone/H_2O mixture (catholyte). Then H_2SO_4 (2.15 mL, 20 mmol, 2.0 equiv.) was slowly added to each flask while stirring and cooling, and in the catholyte additionally isoquinoline **1b** (2.58 g, 20 mmol, 1.0 equiv.) and NBu_4BF_4 (9.88 g, 30 mmol, 1.5 equiv.) were added. The flasks were connected to the flow cell and electrolysis was started. For electrolysis, 92626 C amount of applied charge (48 *F*) were applied under galvanostatic conditions at current density 50 mA/cm^2 (t = 10.7 h) at 30 °C. After the set amount of electric charge has passed, both compartments were immediately emptied to prevent any diffusion of the product from catholyte into the anolyte. Nickel foam in the cathodic compartment was rinsed by pumping 50 mL of MeOH through the cell. Then, methanol and acetone were removed from the collected reaction mixture under reduced pressure at 100 mbar at 50 °C for 15 min. The aqueous phase containing protonated products was then basified. The basification was carried out by slowly adding concentrated aq. NaOH while stirring and cooling the reaction mixture on ice and frequently monitoring pH with indicator papers. The addition of base was stopped once the pH reached >13. Additional 10 mL of H_2O was added to dilute and transfer the neutralized reaction mixture into a separatory funnel. The aqueous phase was extracted three times with 100 mL Et_2O. The combined Et_2O fractions were dried over Na_2SO_4 and kept standing for 2 hours to let the dissolved electrolyte NBu_4BF_4 crystallize out. The crystalized electrolyte and the drying agent were filtered, and the solvent was evaporated to obtain a slightly yellowish oil which still contained some water. The crude product was dried by evaporation of water with toluene and two evaporations with acetonitrile. After additional drying using a rotary evaporator (6 mbar at 50 °C) we obtained 2.392 g of 1,2,3,4-tetrahydroisoquinoline as yellowish oil (17.96 mmol, 89%). The purity was confirmed by ^{1}H NMR.

6 Mechanistic studies

6.1 Cyclic voltammetry

We investigated the behavior of Ni working electrode in a medium mimicking reaction mixture containing 15 mM sulfuric acid and 0.1 M NBu_4BF_4 supporting electrolyte in MeOH:H_2O (3:1). As a reference electrode we used "leakless" Ag/AgCl electrode and as a counter electrode we used glassy carbon rod.

First, we conducted cyclic voltammetry (CV) experiments using $Ni(BF_4)_2$ as a well-soluble Ni(II) source to verify the possibility of Ni(0)/Ni(II) oxidation and reduction (Figure S6). These experiments were conducted in the absence of sulfuric acid. CV measurements showed no additional oxidation or reduction peaks when using Ni as the working electrode. This observation shows that the leached Ni^{2+} ions cannot be reduced under the given conditions.

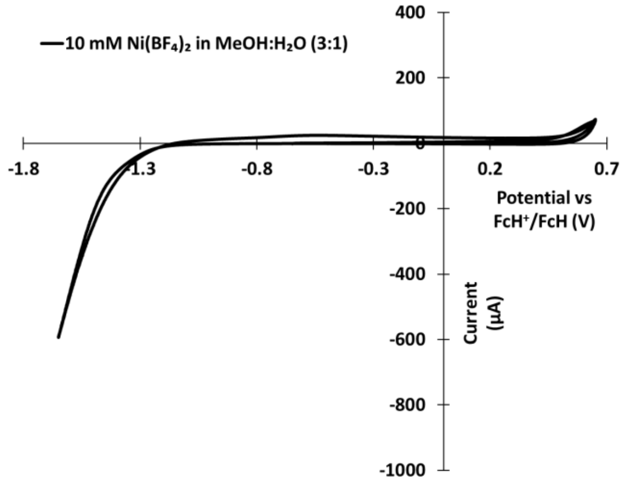

Figure S6: CV of 10 mM $Ni(BF_4)_2$ in MeOH:H_2O (3:1) solution.

After this control CV, we focused our investigation on the redox behavior of protons and N-aza heterocycles at nickel electrode´s surface.

- *Investigation of electrochemical formation and consumption of H_{ads}.*

Two sequential cycles in oxidative direction revealed formation of a new species which sticks to the electrode surface after reduction of protons (**Figure S7**A). An oxidative wave, associated with the oxidation of the newly formed species, appeared in the second cycle (C), only after its formation in the cycle before (B). The newly formed species is highly likely chemisorbed hydrogen (H_{ads}).

A) B) C)

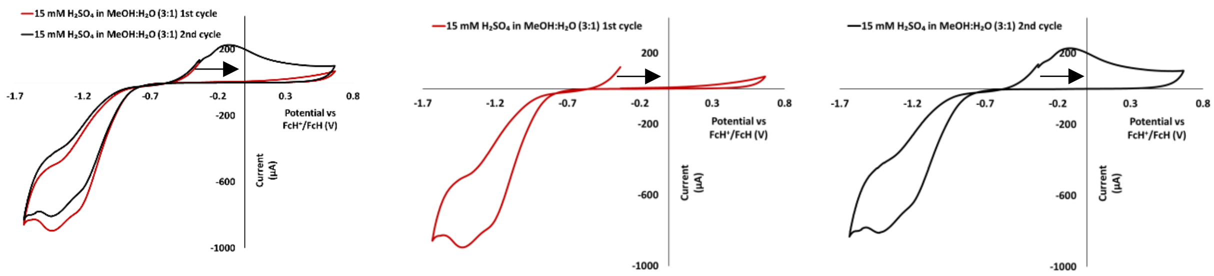

Figure S7: A) Observation of H_{ads} by conducting CV cycles with Ni working electrode in aq. H_2SO_4. The new oxidative peak in the second cycle (C) appeared only after the reduction of protons in the first cycle (B). The black arrows indicate the scanning direction.

In the further experiments, we investigated the properties of H_{ads} buildup and its consumption. For buildup of adsorbed hydrogen on Ni surface we performed four consecutive CV cycles in the region from -0.61 to -1.65 V *vs* FcH^+/FcH (**Figure S8**A). After a few scans the intensity of the reduction peaks starts decreasing likely because of surface saturation with adsorbed hydrogen. To get more information about consumption of the H_{ads} on nickel surface we performed four consecutive CV in the region from –0.61 to 0.35 V vs FcH^+/FcH. Adsorbed hydrogen gets almost quantitively consumed (oxidized) after one scan in oxidative direction. Electrochemical buildup of adsorbed hydrogen on nickel surface and its consumption seems to be very well reversible after the first scan (C).

A) B) C)

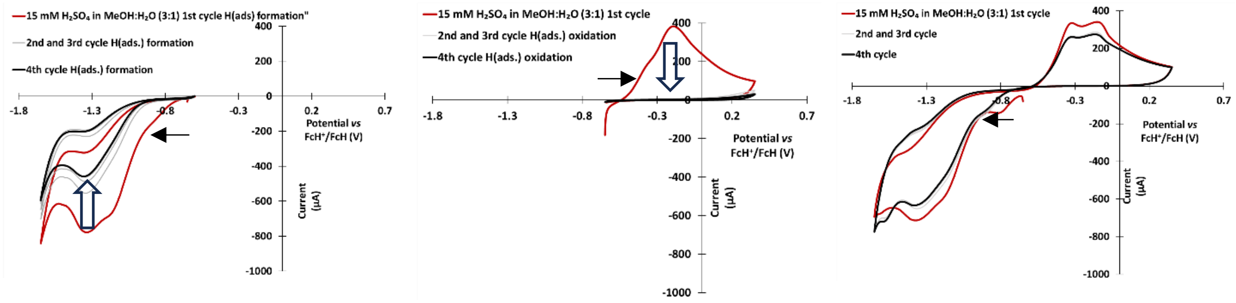

Figure S8: Study of the behavior of nickel working electrode in a reaction medium with (1) formation of H_{ads} in the reductive phase; (2) consumption of H_{ads} in the oxidative cycle; (3) reproducibility of the H_{ads} formation and consumption cycle over 2 scans. The black arrows indicate the scanning direction.

- *CV investigation of chemical reactivity of H_{ads} with different substrates.*

We took advantage of the obtained knowledge about formation and consumption of H_{ads} and performed a sequence of experiments in which we investigated chemical reactions of H_{ads} with different substrates. The experiment design was like previous work of Atobe and Shida.[28]

In these experiments (**Figure S9**A**),** we covered nickel rod electrode´s surface with H_{ads} by performing 5 CV cycles from -0.3 to -1.3 V vs Ag/AgCl in 20 mL 15 mM H_2SO_4 in MeOH/H_2O (3:1). Then 3 CV cycles from -0.3 to 0.7 vs. Ag/AgCl electrode have been performed to estimate the amount of H_{ads} to get a value for a comparison with the following experiments with a substrate. Immediately after, we repeated 5 CV cycles for buildup of H_{ads}, and after their completion, we added 2 mmol of an indicated substrate. The reaction was then stirred for 10 minutes at a potential -0.1 V vs Ag/AgCl at which no currents are flowing. Afterwards, we performed 3 CV cycles to analyze the amount of remaining H_{ads} on the electrode´s surface. For all these experiments we used 100 mV/sec scanning rate. In the cases when the amount of H_{ads} decreased significantly we attributed that to its reactivity with the added substrate (**Figure S9**B).

A. Design of the quenching experiments

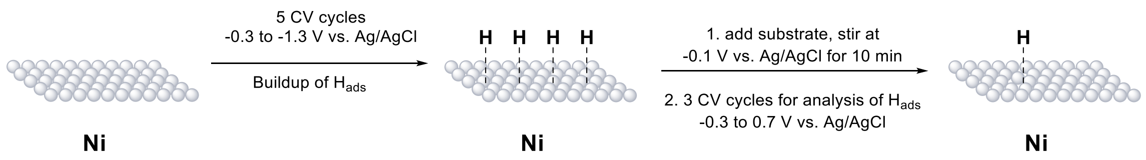

B. Quenching experiments with different substrates

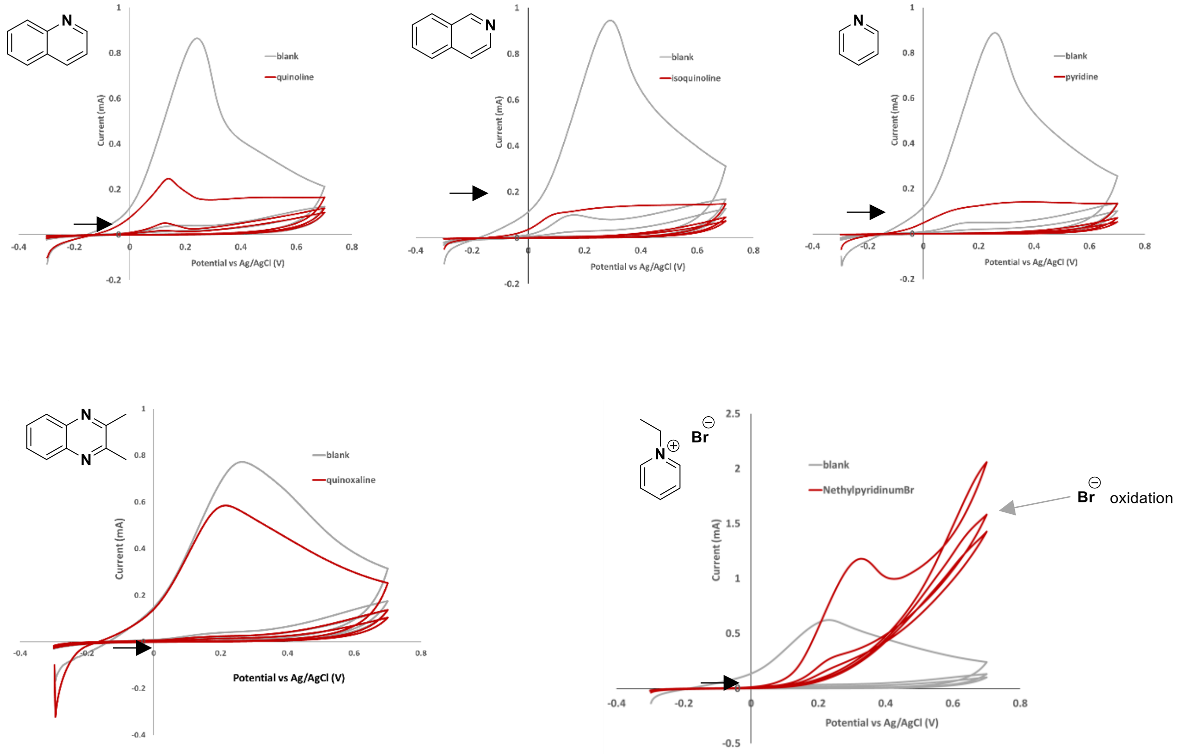

Figure S9: A) Experimental design for evaluation of reaction of H_{ads} and quenching; B) experimental results.

Quinoline, isoquinoline and pyridine were found to quench the H_{ads} layer very efficiently, whereas in the cases of quinoxaline and ethylpyridinum bromide we surprisingly did not observe any significant quenching of the H_{ads} layer. This could indicate a different mechanism leading to product formation.

- *CV investigation of reduction behavior of N-heterocycles under reaction conditions*

Next, we investigated whether the direct reduction of protonated N-heterocycles competes with the reduction of protons. To get more insight into this, we performed a series of CV measurements (**Figure S**). In these measurements we measured CV after each addition of sulfuric acid to the solution of N-heterocycle. In a blank (without any N-heterocycle) solution containing methanol, water and NBu_4BF_4 supporting electrolyte, the addition of acid led to increase in the intensity of the both observed waves corresponding to reduction of protons and oxidation of adsorbed hydrogen. This was observed in all the measured samples. Interestingly, in the case of 2,3-dimethylquinoxaline also a new wave appeared at -1.0 V *vs* FcH/FcH^+ which corresponds to the direct reduction of the protonated heterocycle. This measurement strongly hints at a competitive mechanism of a direct reduction of protonated heterocycle.

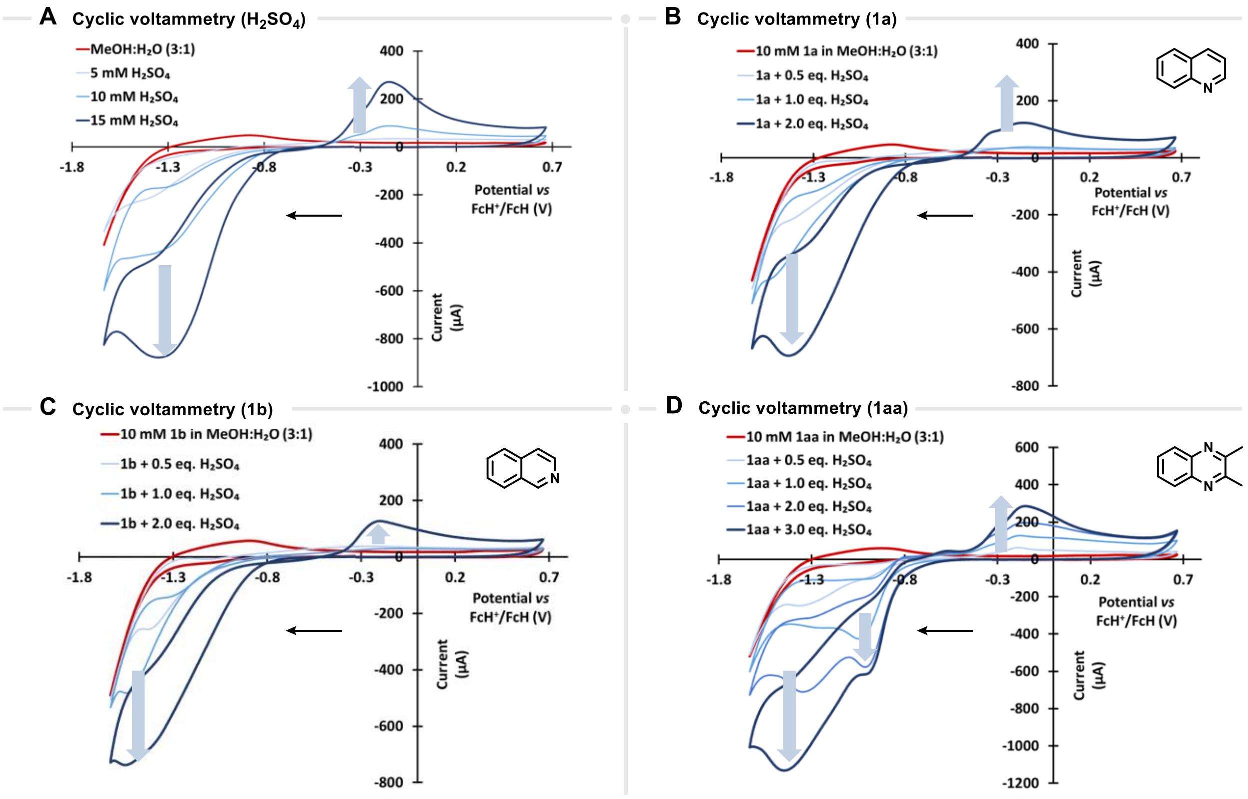

Figure S10: Changes in the CVs upon gradual addition of sulfuric acid to $MeOH:H_2O$ (3:1) solvent mixture containing (B) quinoline **1a**; (C) isoquinoline **1b**; and (D) 2,3-dimethylquinoxaline **1aa**.

6.2 RDE linear sweep voltammetry

To get an additional insight into the kinetics of the reduction processes, we performed a series of experiments using nickel rotating disc electrode (d = 4 mm). In these experiments, we measured currents in 0.3 M H_2SO_4 in MeOH/H_2O (3:1) solution without any supporting electrolyte in the presence of 0.15 M substrates, directly mimicking reaction conditions (**Figure S11**). The measurements were performed at different rpm values, but as the results were in all the similar cases, we show only the results for each substrate at 860 rpm rate. Measurements were performed at relatively slow 10 mV/sec scan rate using Pt disc CE (d = 3 mm) and "leakless" Ag/AgCl reference electrode under inert Ar atmosphere.

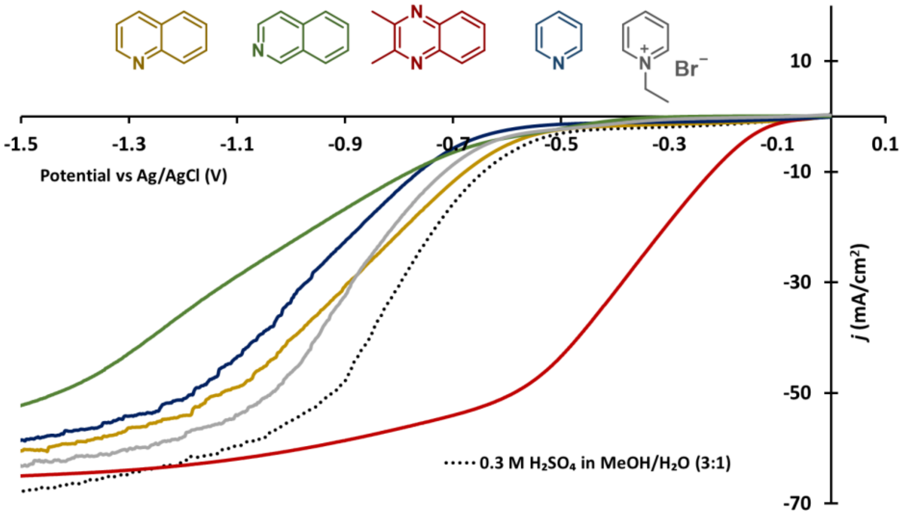

Figure S11: Monitoring of reduction processes in the presence of different substrates using nickel RDE.

- *Quinoxaline*: Much larger cathodic current (early steeper onset) as compared to pure electrolyte of Ni shows that the onset of reaction mechanism is different. Direct reduction of protonated quinoxaline is faster than the proton reduction, indicating this is likely the first reaction step.
- As shown in previous reports[28,29], addition of substrate leads to decrease in the current density (or quenching). We also observed a similar trend when quinoline and pyridine were added to the baseline electrolyte system. Plausibly, this suggests that these substrates are actively blocking the surface and consuming the in situ generated H_{ads}, slowing down the kinetics of ensuing proton reduction and thereby lowering the current density.
- *Ethylpyridinium bromide*: Being a cationic salt, it primarily influences the electrode interface through electrostatic effects rather than π–metal interactions. The ethylpyridinium cations accumulate near the negatively charged Ni surface, thickening the electrical double layer and limiting proton access to active sites. As a result, the onset of cathodic current is shifted to more

negative potentials and appears delayed, without significant surface blocking or direct adsorption on Ni (as kinetic slope is same as baseline).

- *Isoquinoline*: In contrast, isoquinoline forms a non-productive adsorbed layer on the Ni surface, which inhibits proton reduction and alters the overall kinetics. This prompted the use of modified reaction conditions for isoquinoline (with additional electrolyte).

As the mechanism of 2,3-dimethylquinoxaline hydrogenation does not seem to depend on the reduction of protons and buildup of H_{ads} layer, we verified that observation by performing hydrogenation using glassy carbon cathode (under *condition A*) which cannot form H_{ads} very efficiently. Compared to the hydrogenation on nickel foam cathode, the ratio dropped from 94:6 to 52:48 with GC cathode (**Figure S12**A). In addition, the yield dropped from 87% to 27%. This shows that the glassy carbon is not the proper catalyst for building adsorbed H* on the electrode surface. Moreover, the observed diastereomeric ratios in this experiment further supports the reactivity of nickel via H_{ads}. The onset of hydrogen evolution (after H_{ads} formation) is earlier in case of Ni foam in comparison to glassy carbon (B). This makes direct reduction more probable in case of GC since higher potential is required for the H_{ads} formation.

A)

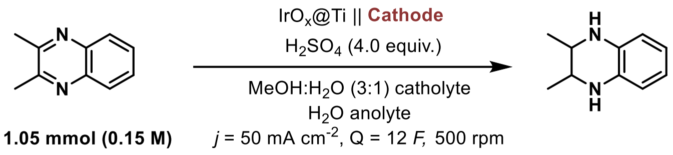

Entry	Cathode	Yield %	*d.r.*
1	Ni foam	87	94:6
2	GC	27	52:48

B)

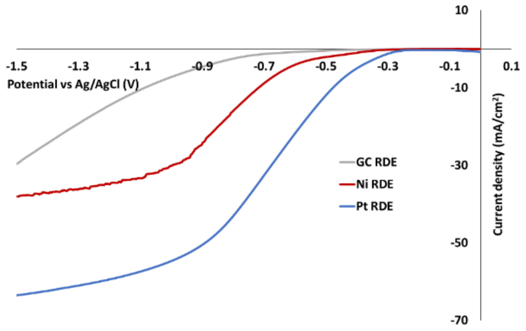

Figure S12: A) Testing glassy carbon (GC) cathode for quinoxaline hydrogenation; B) evaluation of different electrodes for proton reduction under reaction conditions using RDE linear sweep voltammetry.

6.3 Deuteration and Scavenging studies

1,2,3,4-tetrahydroquinoline-2,3,4-*d3* (4)

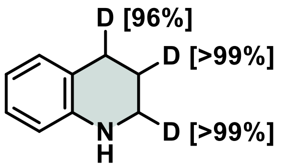

The title compound was synthesized with **1c** using GP-2 (*condition A)* on 1.05 mmol scale.

R_f: 0.8 (80:20 pentane:diethyl ether)

Yield: 97 mg, 68%

^{1}H NMR (400 MHz, CD_3CN) δ 6.93 – 6.81 (m, 2H), 6.56 – 6.39 (m, 2H), 4.60 – 4.17 (m, 1H), 3.27 – 3.15 (m, 1H), 2.67 (t, J = 4.9 Hz, 1H), 1.90 – 1.74 (m, 1H).

^{13}C NMR (101 MHz, CD_3CN) δ 146.4, 130.2, 127.5, 121.8, 117.0, 114.7, 42.3 – 41.4 (m), 27.7 – 26.8 (m), 22.6 – 21.9 (m).

GCMS(EI) m/z: calc'd for $C_9H_8ND_3$ 136.1074; found 136.1075.

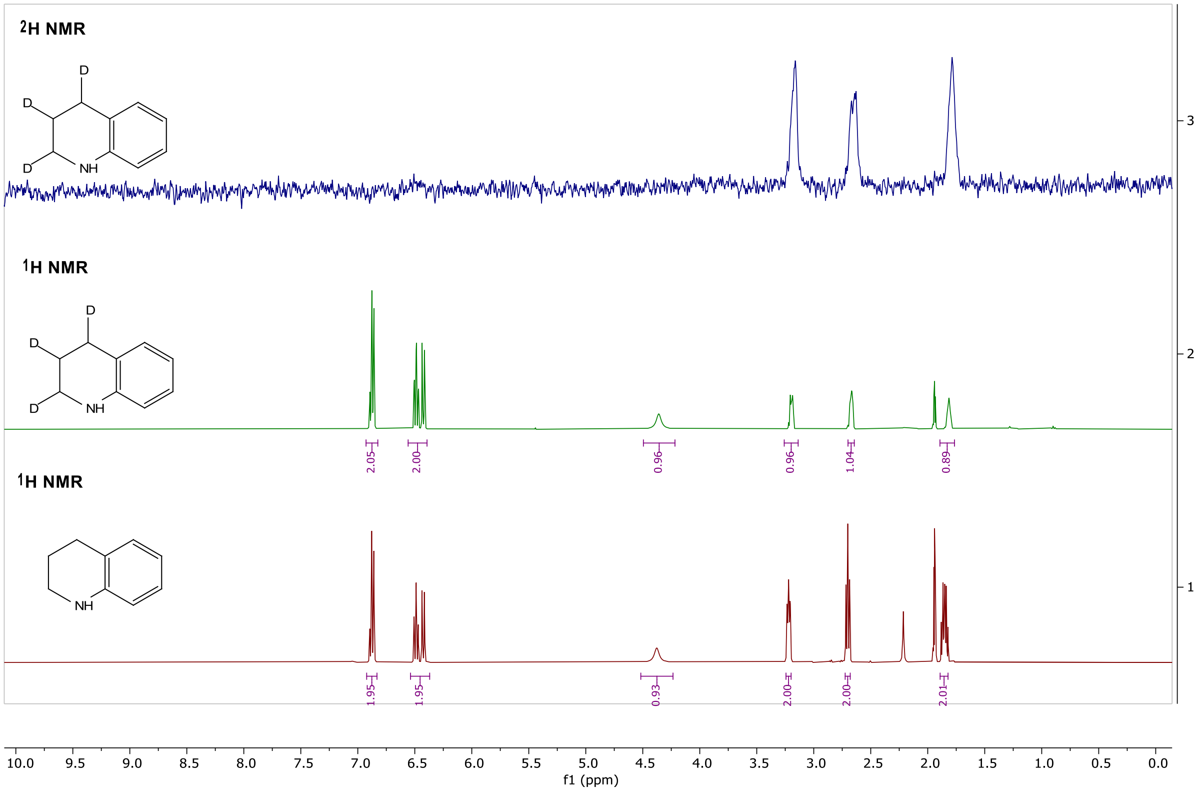

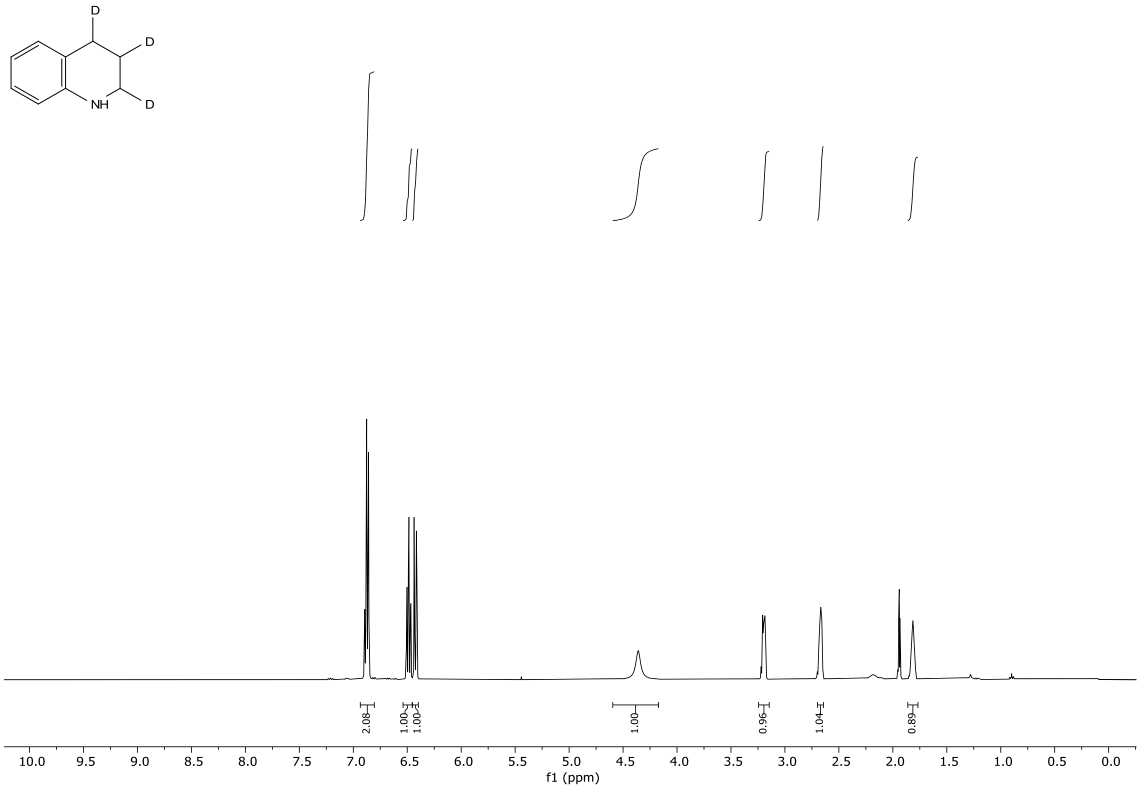
D
D
D
NH
2.08
1.00
1.00
1.00
0.96
1.04
0.89
10.0 9.5 9.0 8.5 8.0 7.5 7.0 6.5 6.0 5.5 5.0 4.5 4.0 3.5 3.0 2.5 2.0 1.5 1.0 0.5 0.0
f1 (ppm)

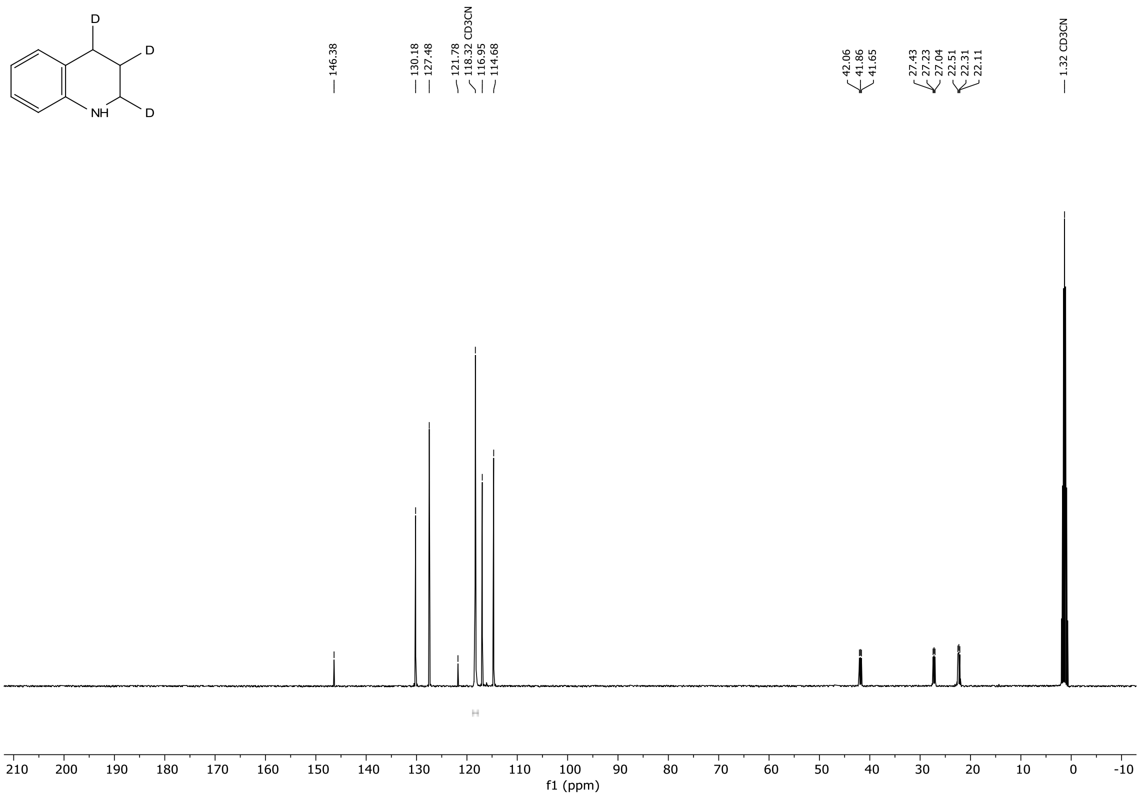
D
D
D
NH
146.38
130.18
127.48
121.78
118.32 CD3CN
116.95
114.68
42.06
41.86
41.65
27.43
27.23
27.04
22.51
22.31
22.11
1.32 CD3CN
210 200 190 180 170 160 150 140 130 120 110 100 90 80 70 60 50 40 30 20 10 0 -10
f1 (ppm)

To verify that H_2O serves as the hydrogen source—originating from its oxidation and subsequent reduction across the membrane—we conducted a batch reaction in which the only change was replacing the anolyte H_2O with D_2O.

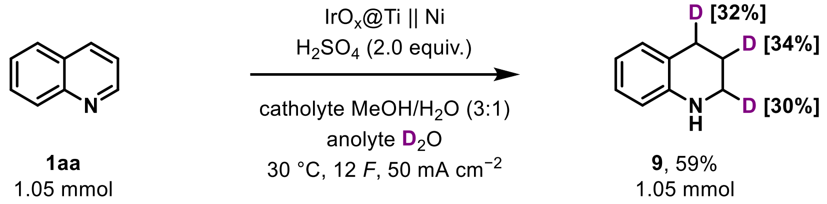

The ~30–34% deuterium incorporation (measured from crude ^{1}H NMR) provides strong evidence that H_2O contributes as a hydrogen source, though not exclusively. A combination of both acid and H_2O contributes to the H source.

Scavenging test: According to literature reports, *tert*-butanol acts as H* scavenger.[30] To further demonstrate H* being the active species, the standard reaction was performed in presence of 5.0 equiv. of *t*BuOH. The study displayed a significant loss in the yield of formation of hydrogenated products. The loss of yield suggests H* being the active species.

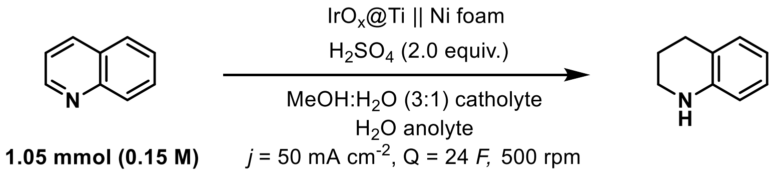

Entry	*tert*-butanol (equiv.)	Yield %
1	No	80
2	5.0	55

6.4 Kinetic monitoring and identification of the intermediate

- ***Progress of reaction***

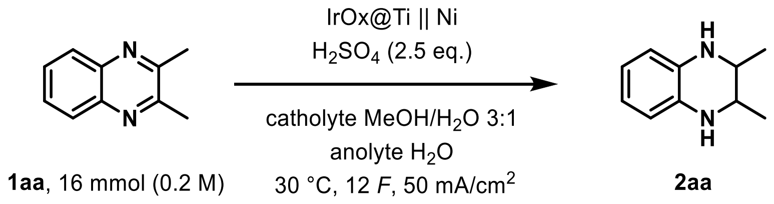

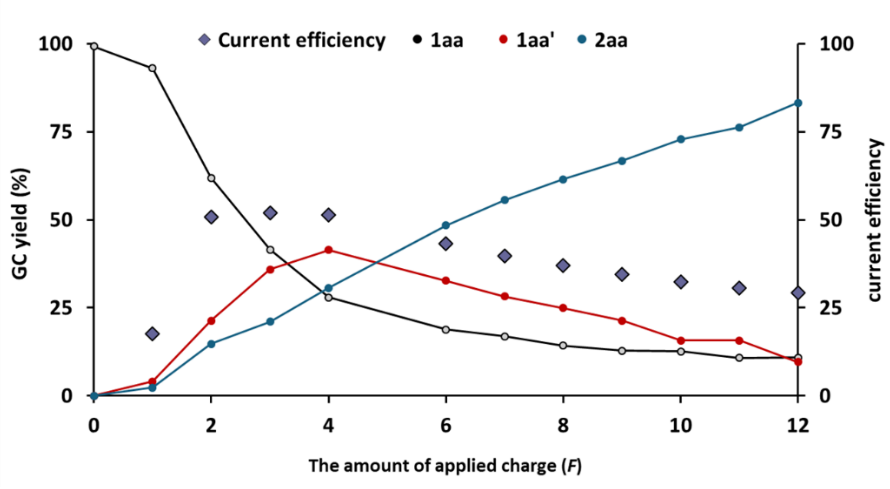

Figure S13: Monitoring progress of the electrochemical hydrogenation of **1aa**.

We used the reaction setup as described and shown in **Figure S5**. A 250 mL Schott bottle was filled with 80 mL H_2O (anolyte) and another 250 mL Schott bottle was filled with 80 mL 3:1 MeOH/H_2O mixture (catholyte). Then H_2SO_4 (2.14 mL, 40 mmol, 2.5 equiv.) was slowly added to each flask while stirring and cooling. In the catholyte flask additionally 2,3-dimethylquinoxaline **1aa** (2.53 g, 16 mmol, 1.0 equiv.) and internal standard anisole (865 mg, 8.0 mmol) were added. The catholyte bottle was intensely shaken to achieve complete homogenization before the flasks were connected to the flow cell and electrolysis was started. For electrolysis, 18525 C of electric charge (12 *F*) were applied under galvanostatic conditions at current density 50 mA/cm^2 at 30 °C. We took 0.1 mL aliquots of the reaction mixture after every time 1 *F* of charge has passed (every 10 min) without stopping the electrolysis in between. The 0.1 mL reaction mixture aliquots were transferred into 1.5 mL conical bottom Eppendorf vials filled with 1.0 mL 1,4-dioxane and 0.1 mL of conc. NaOH. The vials were mixed for 2 minutes using vortex mixer to ensure freebasing and extraction of amines in the organic phase. Then, most of the organic phase was transferred into a GC vial, diluted to a 2 mL mark with additional 1,4-dioxane and submitted for GC analysis.

- ***Characterization of the reaction intermediate '1aa'***

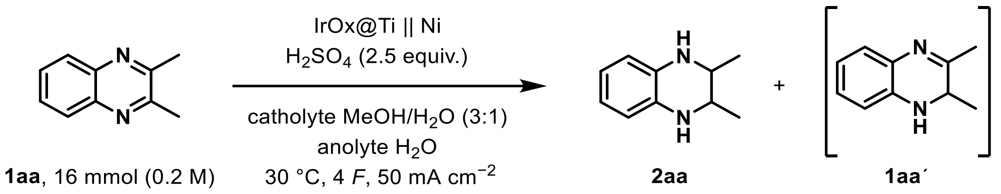

As we observed relatively high concentration of the intermediate in the kinetics monitoring, we repeated the reaction on 16 mmol scale and stopped it after 4 *F* to identify the intermediate. After the set amount of charge has passed, we transferred the reaction mixture into a round bottom flask and removed methanol under reduced pressure by evaporation at 100 mbar at 50 °C for 10 min. The aqueous phase containing the protonated product was then basified. The basification was carried out by slowly adding concentrated aq. NaOH while stirring and cooling the reaction mixture on ice and frequently monitoring pH with indicator papers. The addition of the base was stopped once the pH reached >13. Additional 10 mL of H_2O was added to dilute and transfer the neutralized reaction mixture into a separatory funnel. The aqueous layer was extracted two times with 50 mL Et_2O. The combined Et_2O fractions were dried over Na_2SO_4. The drying agent was filtered, and the solvent was evaporated to obtain light brown oil. The sample was dissolved in CD_2Cl_2 and submitted for NMR analysis.

Our purification attempts of the intermediate by silica gel column chromatography were unfortunately not successful. Nevertheless, the measured NMR spectra of the reaction mixture are relatively clean as they contain only the substrate, intermediate and both isomers of the product. By comparison of the signals in the crude reaction mixture with the reference spectra, we managed to extract the signals corresponding to the reaction intermediate. ^{1}H NMR of the intermediate, clearly shows that the double bond doesn't rearrange to the position between carbons C2 and C3 because we see multiple aromatic signals which correspond to some unsymmetrical species along with a set of aliphatic signals which match well (The rearranged species would, in contrast, have a very symmetric set of signals in the aromatic region and only one aliphatic signal). Carbon spectrum additionally supports the observation of the double bond between nitrogen and carbon atom with a very specific imine carbon peak at high 165 ppm (compared to a lower ppm value expected for enamine at approx. 135 ppm).

2,3-Dimethyl-1,2-dihydroquinoxaline (*from crude mixture*)

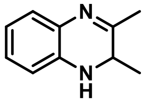

Not isolated. Spectral data obtained by deconvolution of the spectra of a crude reaction mixture after 4 *F* has passed.

^{1}H NMR (400 MHz, CD_2Cl_2) δ 7.26 (dd, *J* = 7.6, 1.5 Hz, 1H), 7.05 (td, *J* = 7.6, 1.5 Hz, 1H), 6.79 (td, *J* = 7.6, 1.5 Hz, 1H), 6.60 (dd, *J* = 7.6, 1.5 Hz, 1H), 4.01 (q, *J* = 6.7 Hz, 1H), 2.19 (s, 3H), 1.27 (d, *J* = 6.7 Hz, 3H).

^{13}C NMR (101 MHz, CD_2Cl_2) δ 165.3, 136.5, 132.5, 127.7, 126.7, 118.3, 113.6, 49.3, 24.0, 18.7.

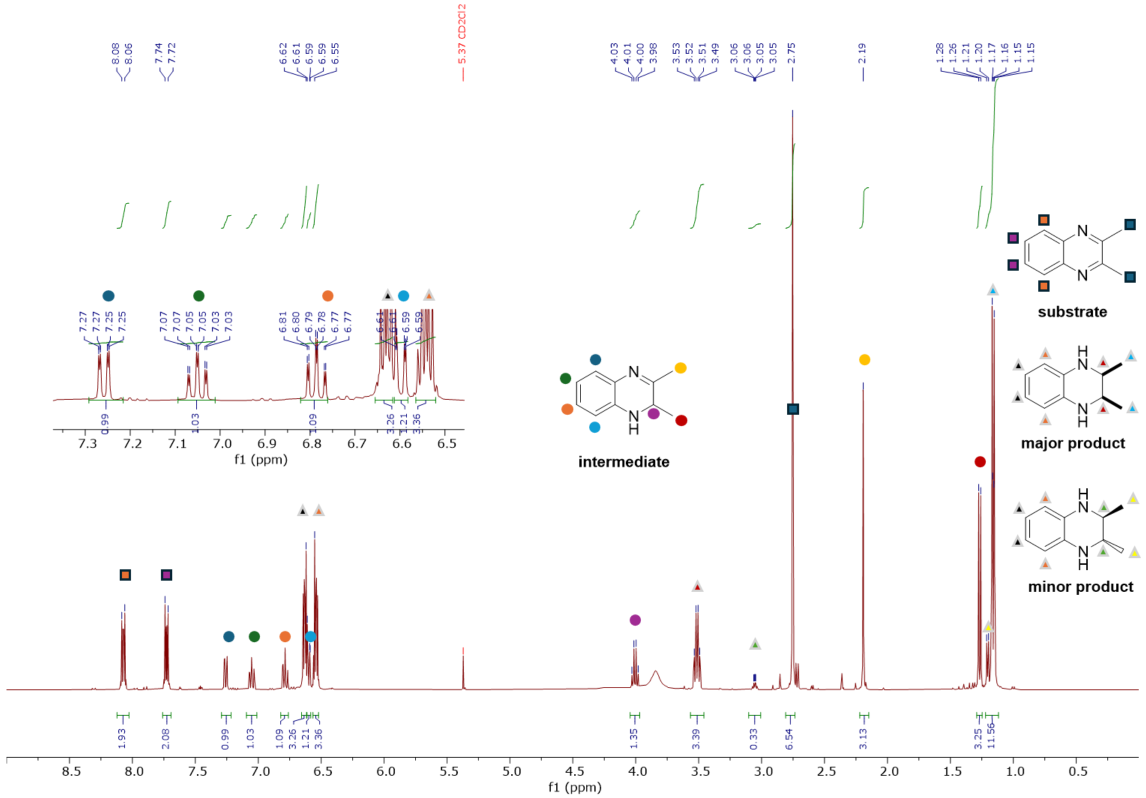

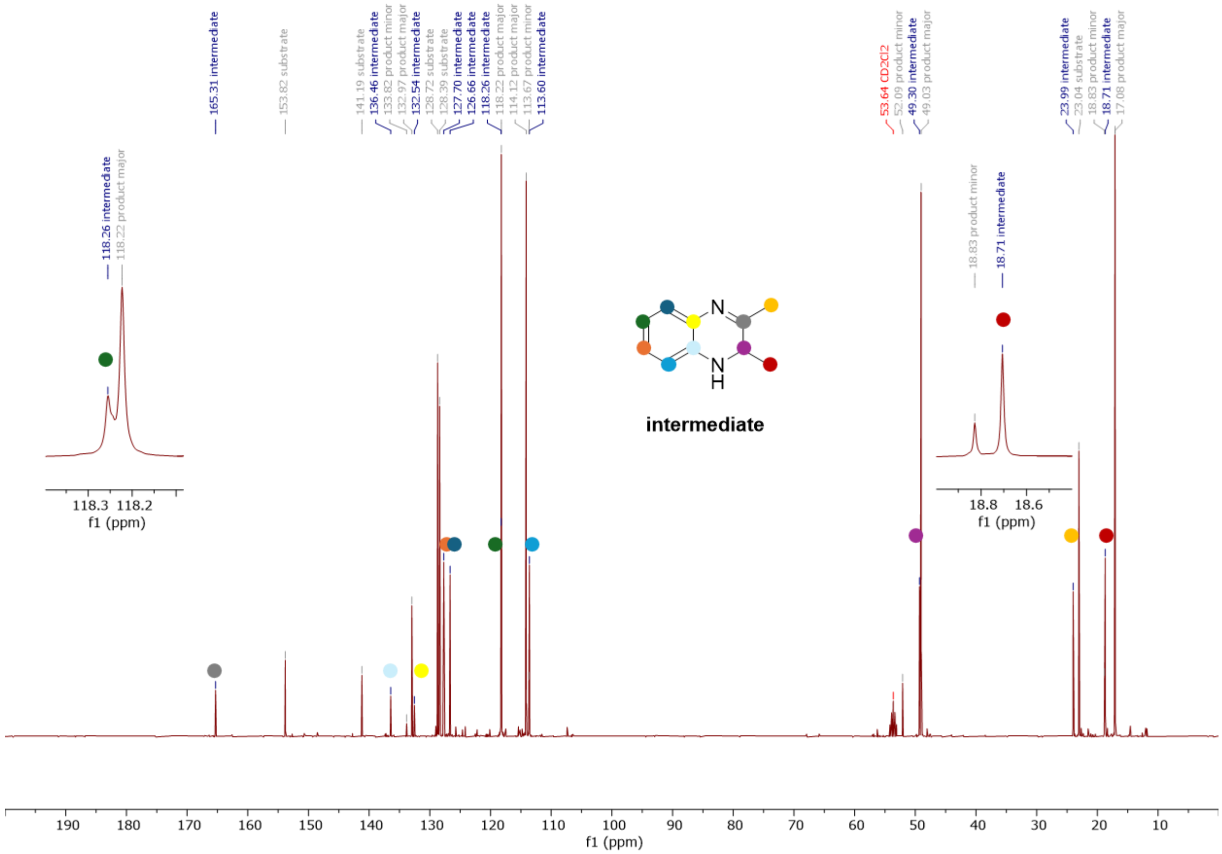

- ***Mathematical modelling of the reaction kinetics:***

  We have now included the whole kinetics for **1aa'**/**2aa** generation (consumption). To begin with the calculations, we considered the kinetics of consecutive reaction under non-steady state approximation:

$$A \xrightarrow{k_1} B \xrightarrow{k_2} C$$

The dependence of time and initial concentration is:

$$A = A_0 e^{-k_1 t}$$

$$B = \frac{k_1 A_0}{k_2 - k_1}\left(e^{-k_1 t} - e^{-k_2 t}\right)$$

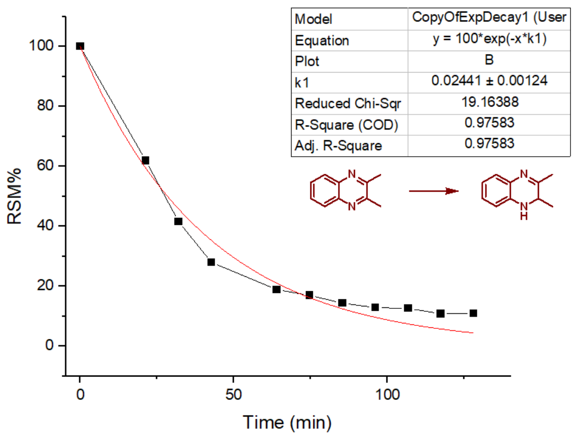

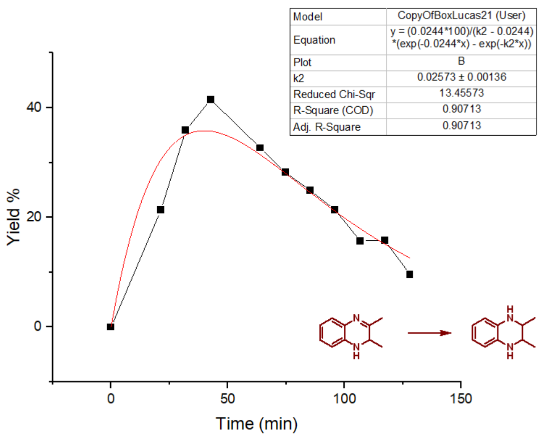

After, remodeling the equations and curve-fit (*the best approximation possible in OriginPro*), the rate constants are:

$$\boldsymbol{k_1 = 0.0244\ min^{-1}} \qquad\qquad \boldsymbol{k_2 = 0.0257\ min^{-1}}$$

The close magnitude of rates for the formation of **1aa** and the rates of formation/disappearance of **1aa'** shows that:

1) intermediate rises gradually and decays slowly (a non-steady regime);

2) $t_{max} = \frac{ln\frac{k_2}{k_1}}{k_2 - k_1}$. According to this equation, the intermediate concentration would go max at around **40 mins**. This is also matching with our kinetic data (41.5 mins). This shows that the curve is fitted properly.

3) Based on the similar rate constants, another alternate/plausible mechanism for quinoxaline hydrogenation is shown here. It is based on proton-coupled H_{ads} transfer. Nevertheless, the substantial stereocontrol loss (against the observed >95:5 d.r. ratio) in the second step suggests this is not the predominant pathway:

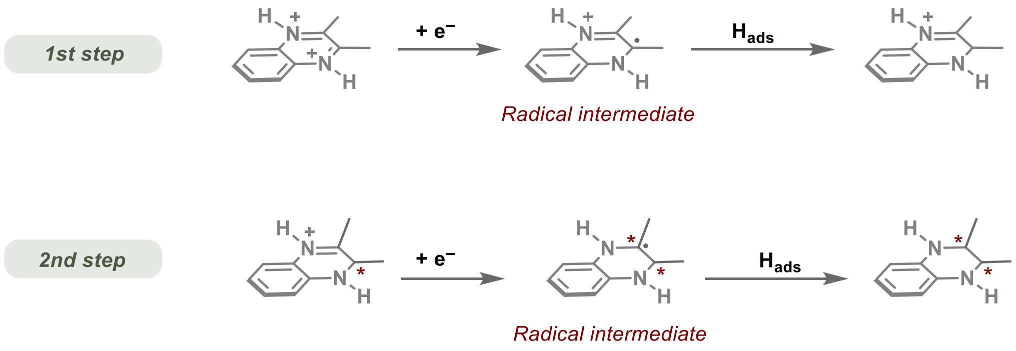

- ***Faradaic efficiency vs conversion:***

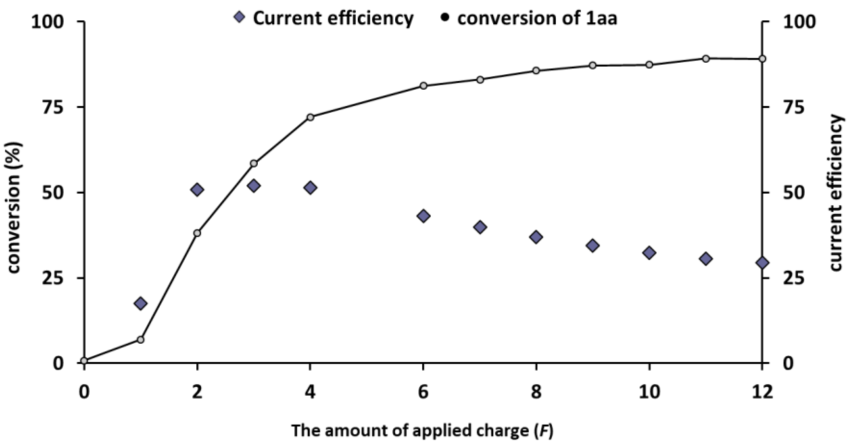

Overall, the Faradaic efficiency is in the range of 18–51%. The low Faradaic efficiency in the beginning could be directly correlated to the sluggish Volmer step. This is followed by competition between desorption process and hydrogenation step. This shows that a significant portion of the surface-adsorbed hydrogen participates in HER reaction.

- ***Tafel analysis:***

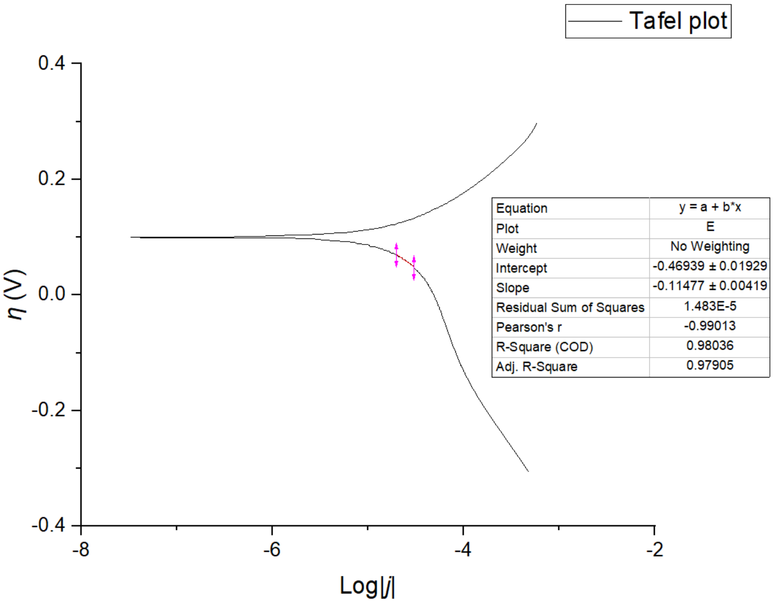

We have obtained the Tafel plot for HER reaction with Ni electrode as working electrode (to mimic the reaction conditions). The calculations are as shown below:

$$E_{RHE} = E_{Ag/Agcl} + 0.059 \times \mathrm{pH} + 0.197\ \mathrm{V}$$

With pH = 1.7,

$$E_{RHE} = E_{Ag/Agcl} + 0.297 \text{ V}$$

For HER in acid,

$$\eta = E_{RHE}$$

Tafel equation:

$$\eta = a + b \times log|j|$$

The slope (b) of the Tafel plot turns out to be 114.8 mV/dec.[31] This value suggests that the Volmer's process is the rate limiting step. It directly translates to the slow buildup of H_{ads} ($H^+ + e^- \rightarrow H_{ads}$). Once the H_{ads} is formed, the desorption *via* either Tafel or Heyrovsky is faster. This also explains the excess amount of applied charge since there is competition between desorption mechanisms or hydrogenation reaction.

7 Reusability and reproducibility study

To evaluate the reusability of the components, reactions with **1aa** were carried out consecutively ten times. The electrolyses were performed sequentially without opening the cell. Experiments in recirculating mode were performed using a modular flow cell (**Figure S2**). The cathode consisted of a 40 mm × 120 mm nickel plate electrode in contact with nickel foam ("Ni 4753," 3.0 mm thick, average pore size 0.40 mm). The foam was cut with a utility knife to fit into a 36 mm × 131 mm opening of a 3 mm thick Teflon™ spacer. The counter half-cell was equipped with a 40 mm × 120 mm DSA electrode (IrO_x on Ti).

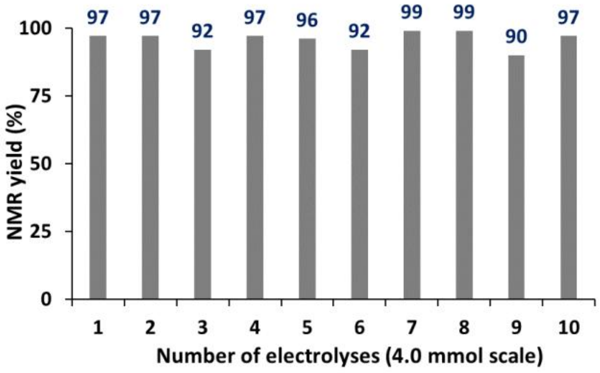

Figure S14: Reusability and reproducibility study of the reaction of **1aa** in flow-type setup.

We used the reaction setup as described and shown in **Figure S5**. For each experiment 50 mL Falcon centrifuge tube was filled with 20 mL H_2O (anolyte) and another 50 mL Falcon centrifuge tube was filled with 20 mL 3:1 MeOH/H_2O mixture (catholyte). Then H_2SO_4 (0.53 mL, 10 mmol, 2.5 equiv.) was added to each tube, and in the catholyte additionally 2,3-dimenthylquinoxaline (633 mg, 4 mmol, 1.0 equiv.) was added. The centrifuge tubes were closed, shaken briefly and then connected to the flow cell. In each electrolysis experiment, 4631 C (12 *F*) of electric charge was applied under galvanostatic conditions at 50 mA/cm2 current density which corresponds to 2.4 A in our 48 cm^2 flow electrolyser. Immediately after the set amount of electric charge has passed, the anolyte compartment was emptied to prevent diffusion of the product from catholyte into the anolyte. In the catholyte an internal standard (464 mg maleic acid) was added along with ca. 10 mL additional MeOH. The reaction mixture was pumped through the cell for approximately 10 minutes to ensure that the reaction mixture trapped in the foam got mixed with the internal standard. Afterwards the catholyte compartment was emptied, both compartments were washed by cycling reaction solvents for ca 5 min. The reactor was emptied, before the next reaction was set following the same procedure. For NMR analysis, we took 0.3 mL of the reaction mixture containing internal standard and removed solvent solvents under reduced pressure (75 mbar at 50 °C for 3 min). The resulting oil was dissolved in *d6*-DMSO and the sample was submitted for ^1H NMR analysis. In some cases, signal of water in NMR overlapped with the peak of internal standard which made integration unreliable. In these cases,

0.1 mL of trifluoroacetic was added into the NMR tube to shifted the signal of water towards higher ppm values.[32]

The results show that all the components of the electrolyser can be reused several times with only minor fluctuations in the reaction yield.

8 Surface analysis

8.1 SEM analysis

Along with the reusability and continuous operation discussion, surface characterization data is also provided after prolonged electrolysis.

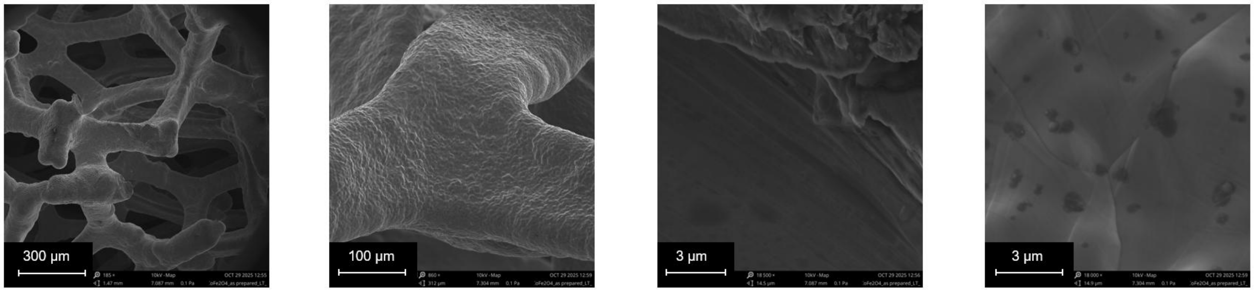

Figure 15. SEM images of surface **before reaction** (as commercially receive)

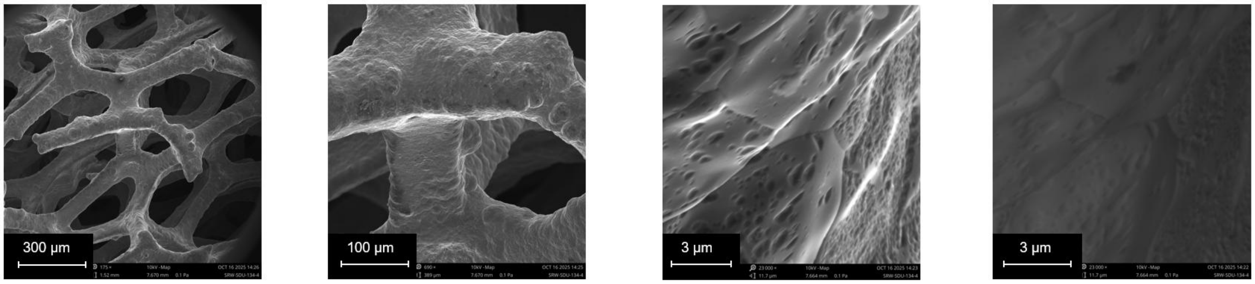

Figure 16. SEM images of surface **after electrochemical reaction** (22 h electrolysis)

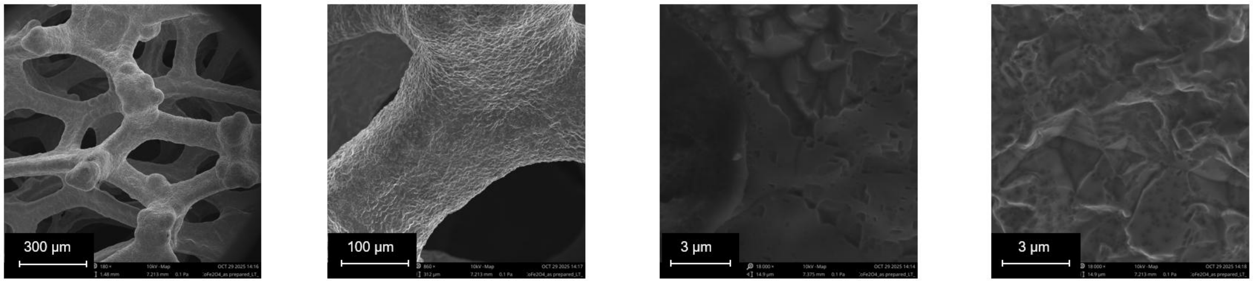

Figure 17. SEM images of surface **under acidic medium without electricity** (4.5 h operating time)

Before reaction: Ni foam shows a well-defined, three-dimensional porous surface (Figure **xx**). The interconnected ligaments are smooth and compact, with clean surfaces and no visible cracks or deposits. The pore walls show a uniform texture, confirming the pristine metallic Ni framework. At higher magnification (3 µm scale), the surface is largely featureless, indicating the absence of significant oxide formation or morphological irregularities prior to electrochemical use.

After reaction: The overall structure of the Ni foam remains intact, demonstrating good mechanical and structural stability under optimized reaction conditions (Figure **xx**). However, minor changes are visible on the surface of the ligaments – turned rough and pitted, showing small protrusions. The fine-scale images reveal the emergence of nanoscale granularity and uneven texture.

Under acidic medium without electricity: To test the durability of the Ni electrode in acidic electrolyte, a similar setup was used without electricity for the same amount of time. At higher magnification, nanoscale features such as cracks, folds, and clusters are evident, pointing to surface activation. Nevertheless, the electrode can be successfully used for electrolysis after short rinsing, or prolonged dissolution under acidic (H_2SO_4) conditions.

8.2 XPS analysis

The Ni $2p_{3/2}$ XPS spectra of the Ni foam before and after electrochemical reaction reveal distinct surface chemical changes. In the pristine state, the Ni foam predominantly exhibits metallic Ni^0 features at around 852.6 eV, accompanied by characteristic Ni^{2+} peaks corresponding to NiO and $Ni(OH)_2$ (centered near 854–856 eV) and their satellite structures.

After serving as the cathode during electrolysis in an H_2SO_4-containing electrolyte, the overall intensity of Ni^{2+} species decreases, while the relative contribution of the metallic Ni^0 component becomes more pronounced. This suggests that surface Ni^{2+} species were electrochemically reduced under cathodic polarization, regenerating metallic nickel. In addition, new features assigned to $NiSO_4$ appear near 856–857 eV, likely originating from surface interactions between nickel and sulfate anions present in the acidic electrolyte.

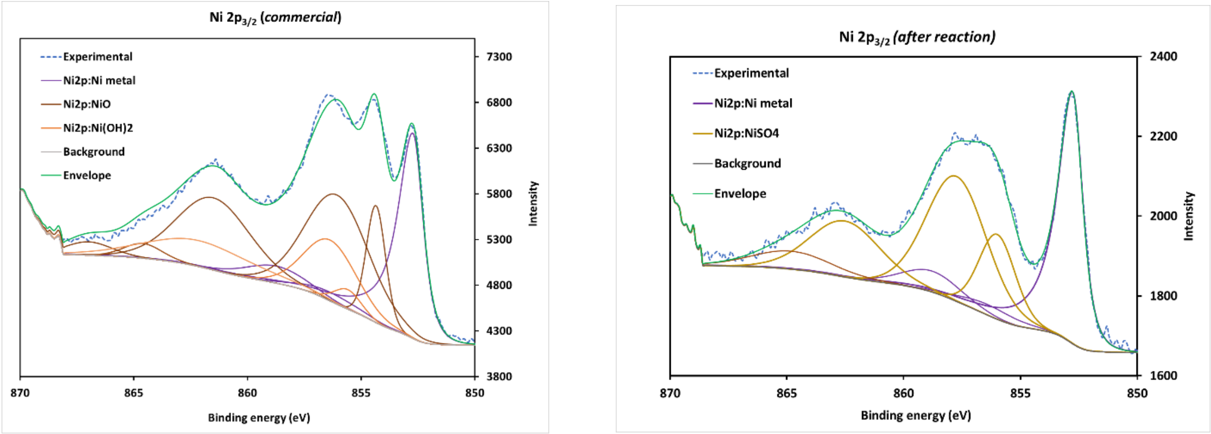

Figure 18. XPS spectra of Ni $2p_{3/2}$ region for Ni foam (a) as received and (b) after electrolysis.

Surface analysis	Ni^0 (%)	Ni^{2+} (%)		
		NiO	$Ni(OH)_2$	Total Ni^{2+}
Before reaction	**26.41**	50.67	22.92	**73.59**
After reaction	**39.51**	60.49 (as $NiSO_4$)		**60.49**

These results confirm that the Ni foam electrode maintains good chemical stability and remains active under cathodic reaction conditions.

9 Quinoxaline case study

An overall summary is provided here.

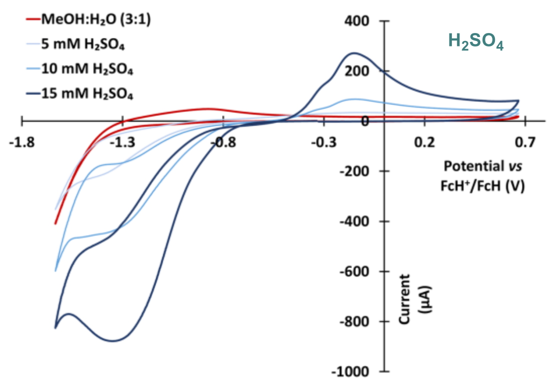

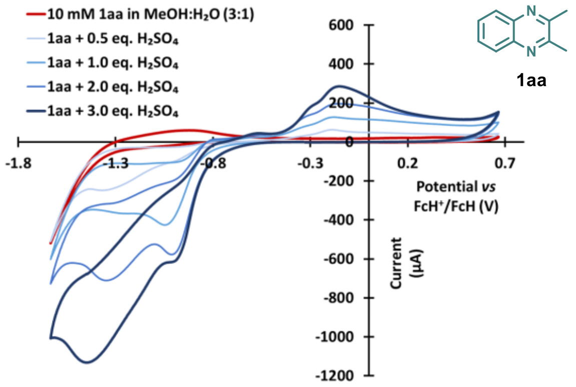

<u>Observations</u>

*CV for **H_2SO_4***	*CV for quinoxaline **1aa***
• Reduction wave at –1.3 V *vs* FcH^+/FcH<br>• Oxidation wave at –0.15 V *vs* FcH^+/FcH	• First reduction wave at –1.0 V *vs* FcH^+/FcH<br>• Second reduction wave at –1.45 V *vs* FcH^+/FcH

<u>Inference</u>: The reduction wave observed for H_2SO_4 indicates the accumulation of adsorbed hydrogen (H_{ads}) on the Ni electrode. The first reduction potential of the deprotonated quinoxaline **1aa** (–1.0 V *vs* FcH^+/FcH), which occurs prior to proton reduction, suggests a direct proton-coupled electron transfer (PCET) pathway leading to the formation of the monoprotonated intermediate **1aa′**. From this point, a competition may arise, as the proton reduction potential (–1.3 V) lies between the first (–1.0 V) and second (–1.45 V) reduction potentials of the substrate. Consequently, the intermediate can either react with the *in-situ* generated H_{ads} species or proceed through a further PCET-driven reduction. Our observations show that the two methyl groups in the aza-ring of quinoxaline predominantly adopt a *cis* configuration in the final product mixture, although a minor trans isomer is also detected. The following discussion illustrates the formation of each isomer based on these mechanistic insights:

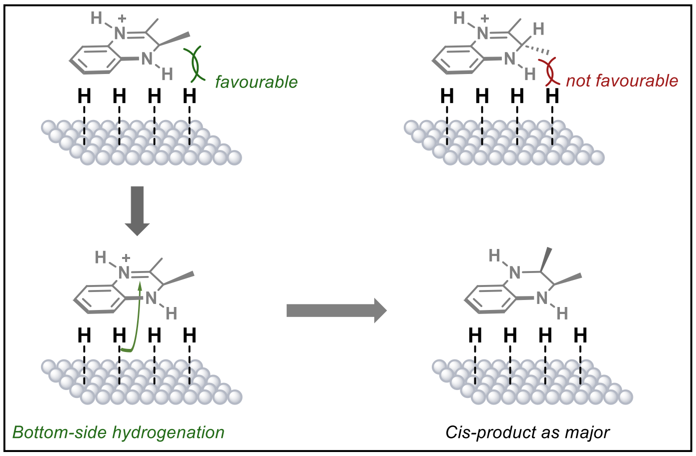

Formation of cis-product as major product

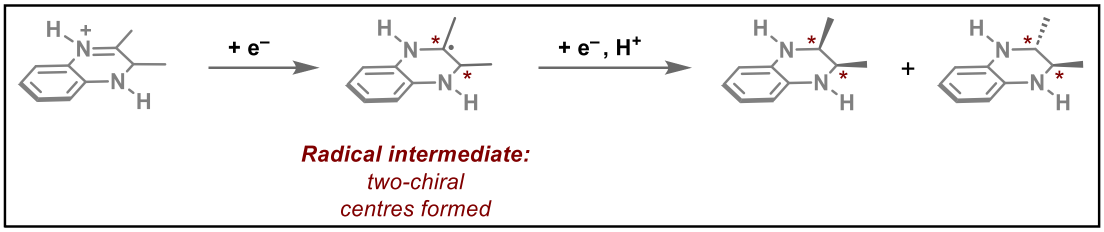

Formation of mixture of isomers with direct reduction

<u>Formation of cis-product as major product</u>: The adsorbed hydrogen (H_{ads}) has two possible pathways to hydrogenate the intermediate **1aa′**. The C2-methyl group influences the orientation of **1aa′** on the electrode surface, positioning the substituent away from it. This orientation makes the hydrogenation of the arene system from the opposite (bottom) side more favorable, leading predominantly to the formation of the *cis* product (substituents on the same side of the planar ring).

<u>Formation of mix products</u>: The direct reduction of **1aa'** produces a radical intermediate where the chirality information is lost. Further PCET produces a mixture of *cis*- and *trans*-isomers.

The whole kinetics for **1aa'**/**2aa** generation (consumption) is further analyzed. To begin with the calculations, we considered the kinetics of consecutive reaction under non-steady state approximation:

$$A \xrightarrow{k_1} B \xrightarrow{k_2} C$$

The dependence of time and initial concentration is:

$$A = A_0 e^{-k_1 t}$$

$$B = \frac{k_1 A_0}{k_2 - k_1}(e^{-k_1 t} - e^{-k_2 t})$$

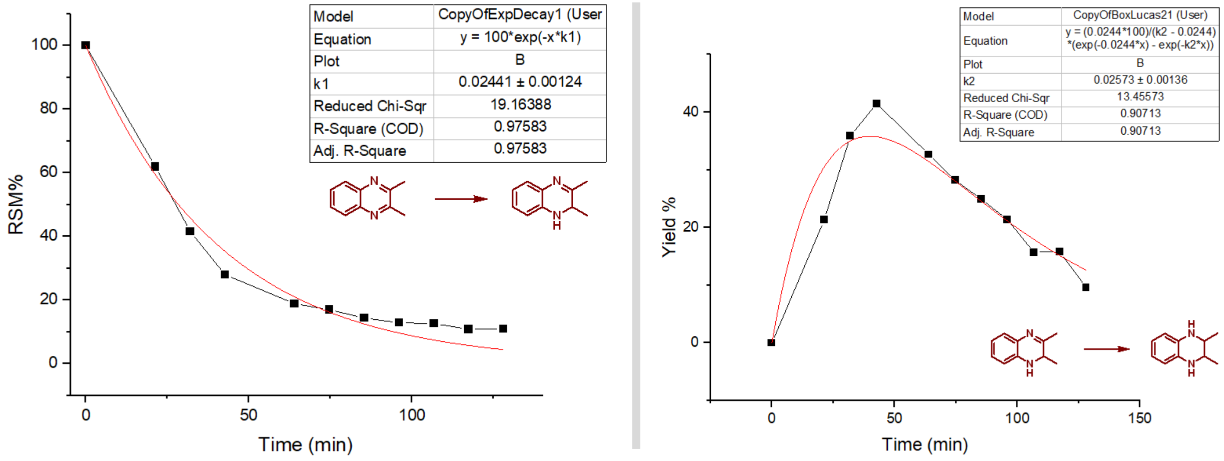

After, remodeling the equations and curve-fit (*the best approximation possible in OriginPro*), the rate constants are:

$$k_1 = 0.0244\ min^{-1} \qquad\qquad k_2 = 0.0257\ min^{-1}$$

The close magnitude of rates for the formation of **1aa** and the rates of formation/disappearance of **1aa'** shows that:

1) intermediate rises gradually and decays slowly (a non-steady regime);

2) $t_{max} = \frac{ln\frac{k_2}{k_1}}{k_2 - k_1}$. According to this equation, the intermediate concentration would go max at around **40 mins**. This is also matching with our kinetic data (41.5 mins). This shows that the curve is fitted properly.

3) Based on the similar rate constants, another alternate/plausible mechanism for quinoxaline hydrogenation is shown here. It is based on proton-coupled H_{ads} transfer. Nevertheless, the substantial stereocontrol loss (against the observed >95:5 d.r. ratio) in the second step suggests this is not the predominant pathway:

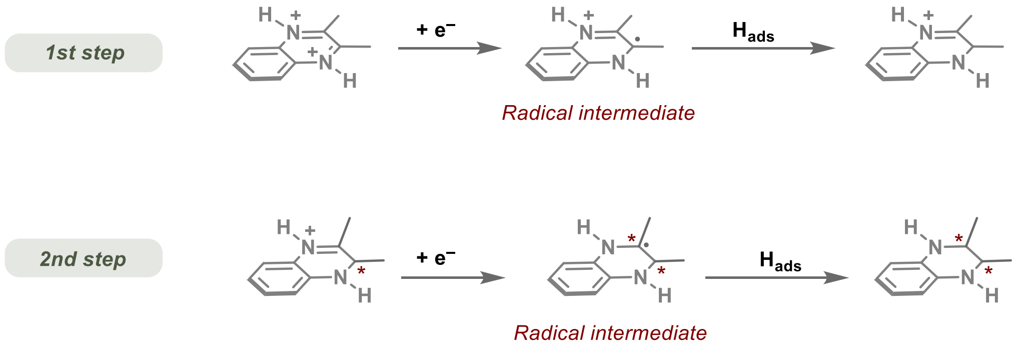

- ***Electrode comparison study:***

The role of Ni foam as cathode was further demonstrated with the diastereomeric ratios obtained for the quinoxaline hydrogenation. When the same reaction was performed with glassy carbon (GC) electrodes, the ratio dropped to 52:48 from 94:6. In addition, the yield dropped from 87% to 27%. This shows that the glassy carbon is not the proper catalyst for building adsorbed H* on the electrode surface. Moreover, this experiment further supports reactivity via H_{ads}.

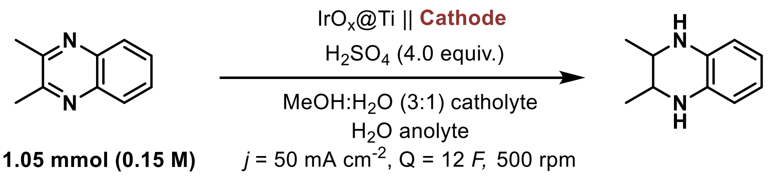

Entry	Cathode	Yield %	*d.r.*
1	Ni foam	87	94:6
2	GC	27	52:48

Linear sweep voltammetry analysis of these two electrodes were also conducted. The onset of hydrogen evolution (after H_{ads} formation) is earlier in case of Ni foam in comparison to glassy carbon. This makes the direct reduction more probable in case of GC since higher potential is required for the H_{ads} formation.

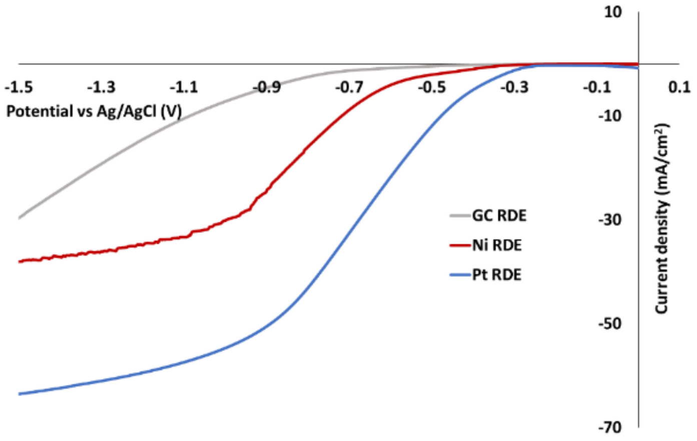

10 Comparison with the state-of-the-art methodologies

1. Electrochemical reports

Reference	Cathode material	Commercial availability	Faradaic efficiency (%)	Source of H	Current density
**1**	Rh/KB	No	13.9	H_2O	25 mA cm^{-2}
**2**	Pt/C	Yes	14.4	H_2	25 mA cm^{-2}
**3**	Modified Co-F	No	5.4	H_2O	100 mA cm^{-2}
**4**	$MoNi_4$	No	4.25	H_2O	25 mA cm^{-2}
This work	Unmodified Ni foam	Yes	13.5	H_2O	50 mA cm^{-2}

1. *J. Am. Chem. Soc.* **2024**, *146*, 30212–30221
2. *Beilstein J. Org. Chem*. **2024**, *20*, 1560–1571
3. *Nat. Commun.* **2022**, *13*, 5297
4. *Chin. J. Catal.* **2021**, *42*, 1983–1991

2. Thermochemical reports *(with noble and non-noble methods)*

Reference	Catalyst	Temperature (°C)	H_2 Pressure (bar)	Commercially available?	Yield (%)
**1**	Ru-NPs	80	10	No	99
**2**	NanoRu@hectorite	100	30–60	No	95
**3**	Fe(N-C)@C-800	130	40	No	87
**4**	$Mn(CO)_5Br$	45	15	No	>99
**5**	Co-pyromellitic acid@SiO_2	70	10	No	97

1. *Green Chem.* **2017**, *19*, 2762–2767
2. *Appl. Catal. A Gen.* **2013***, 467,* 310–314
3. *Chem. Sci*. **2018**, *9*, 8134–8141
4. *Nat. Catal.* **2020**, *3*, 135–142
5. *Angew. Chem. Int. Ed.* **2020**, *59*, 17408 –17412

It is clear from the data presented that most of the electrochemical transformations required a modified electrode which is not commercially available. Moreover, the majority of the modified electrodes suffered with low FE in comparison to our work. When compared to the thermal, even including the non-noble metals, the point of high temperature and pressure goes against the functional group tolerance and safety standards. Similar to the ECH methods, most of the catalysts are not commercially available.

11 NMR spectra

^{1}H NMR spectrum (400 MHz, $CDCl_3$) of **1l**

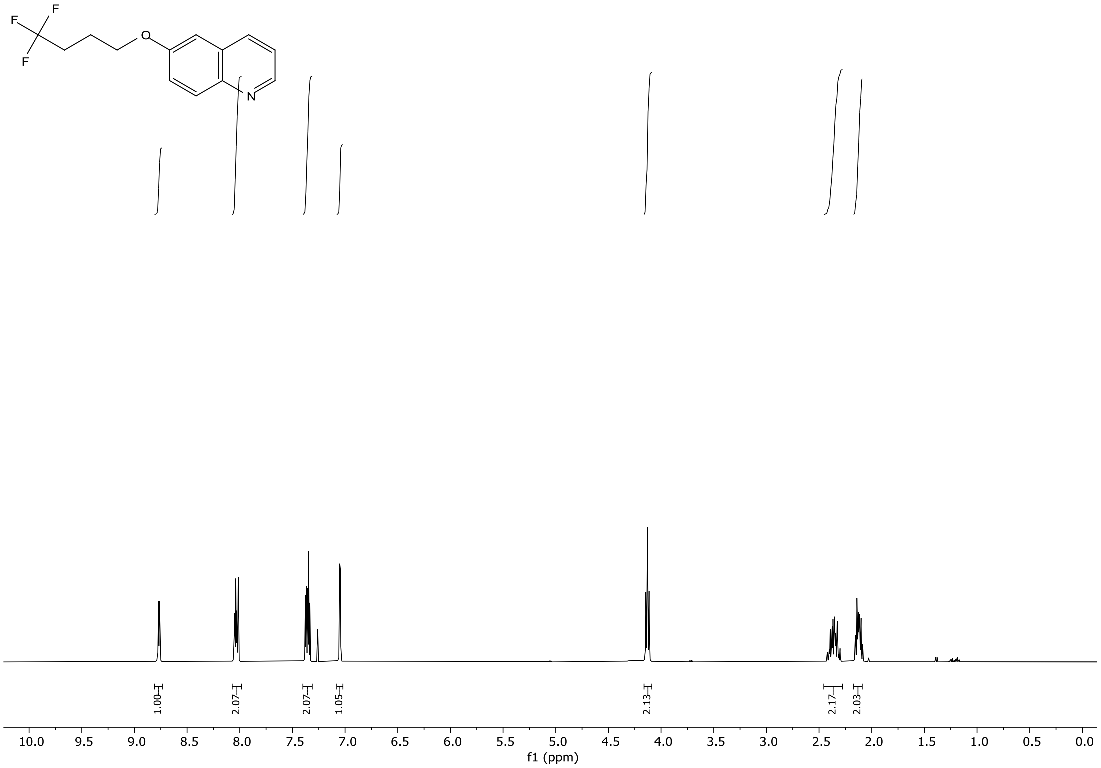

^{13}C NMR spectrum (400 MHz, $CDCl_3$) of **1l**

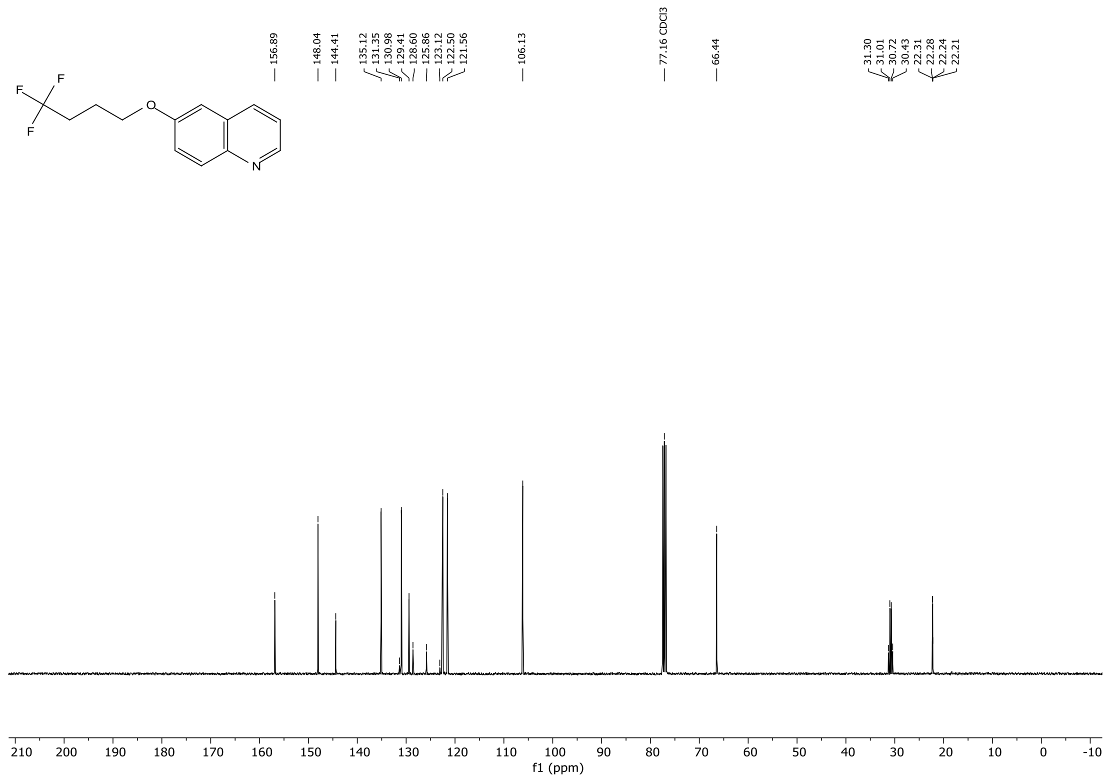

^{19}F NMR spectrum (400 MHz, $CDCl_3$) of **1l**

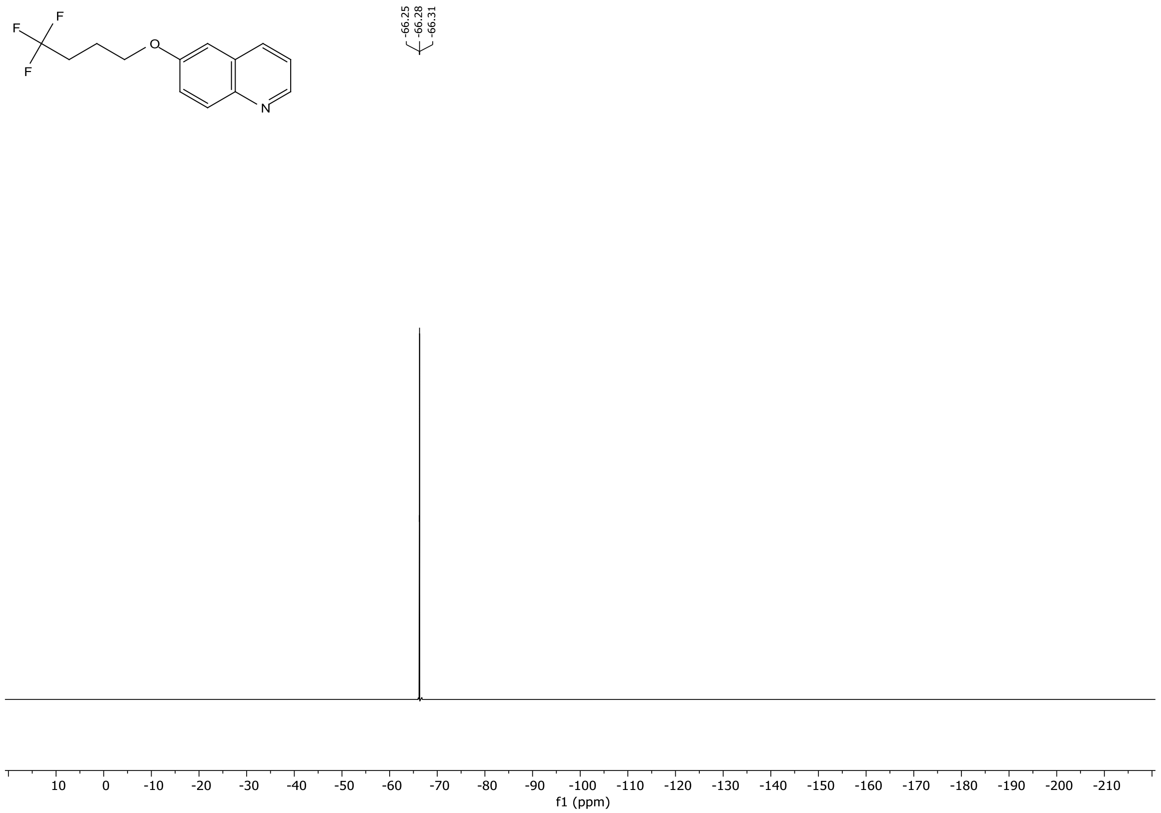

^{1}H NMR spectrum (400 MHz, CD_3OD) of **1w**

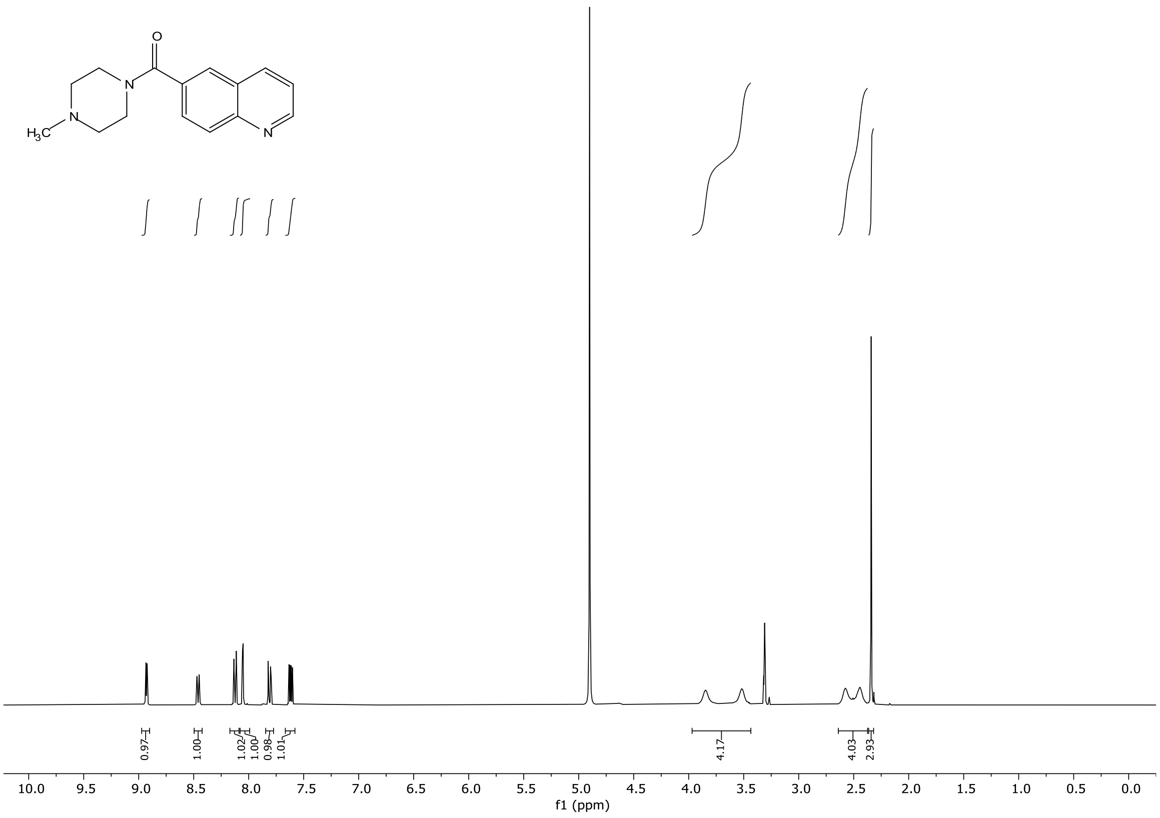

^{13}C NMR spectrum (400 MHz, CD_3OD) of **1w**

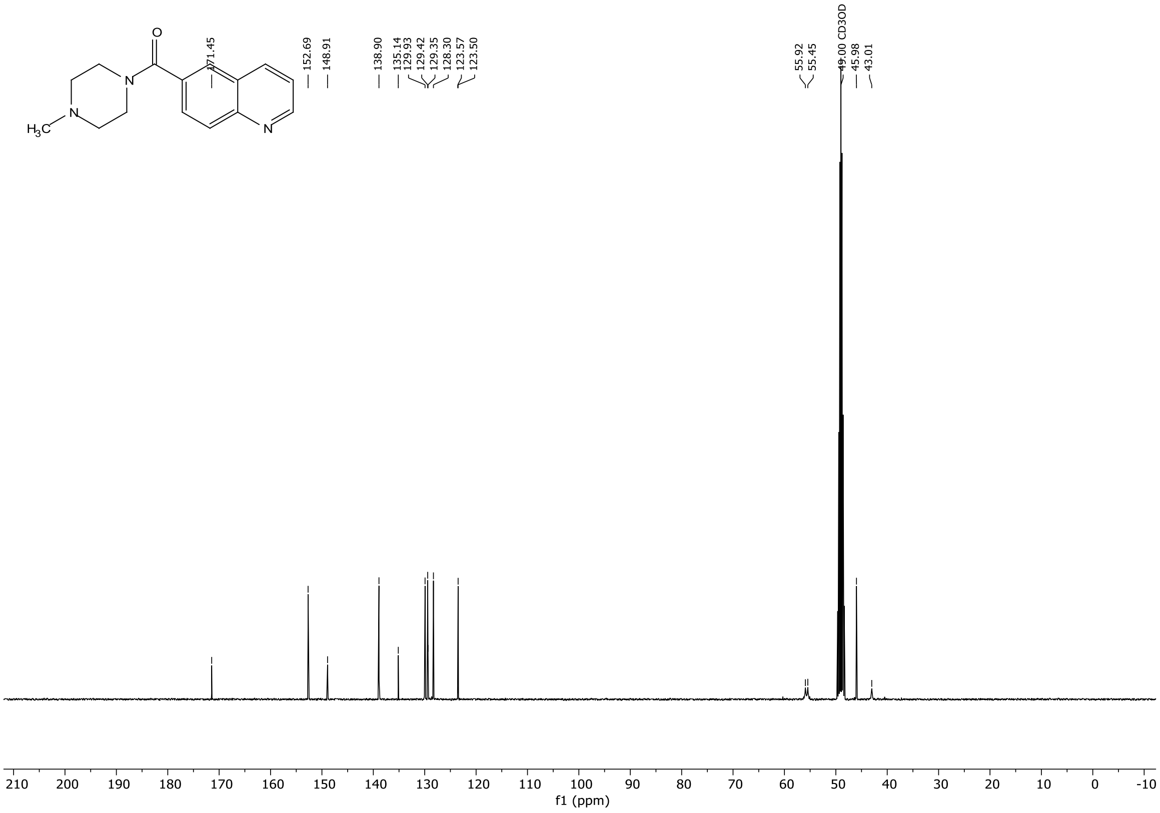

^{1}H NMR spectrum (400 MHz, CD_3OD) of **1y**

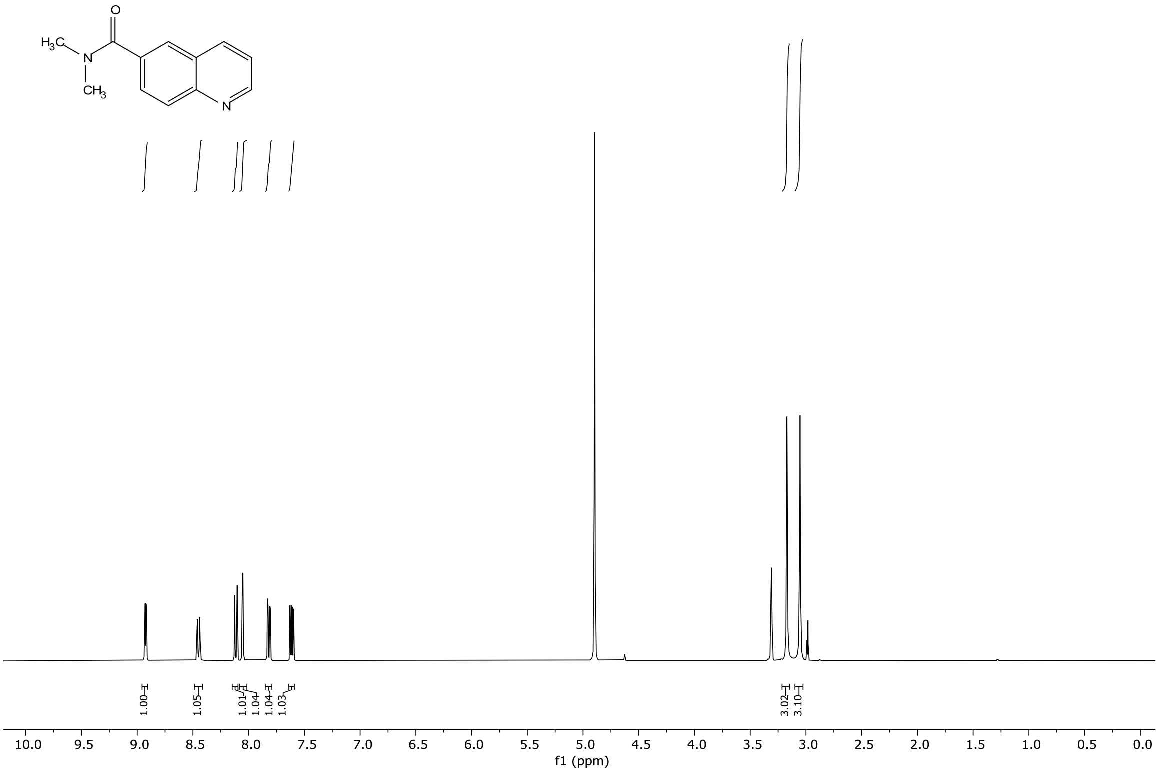

^{13}C NMR spectrum (400 MHz, CD_3OD) of **1w**

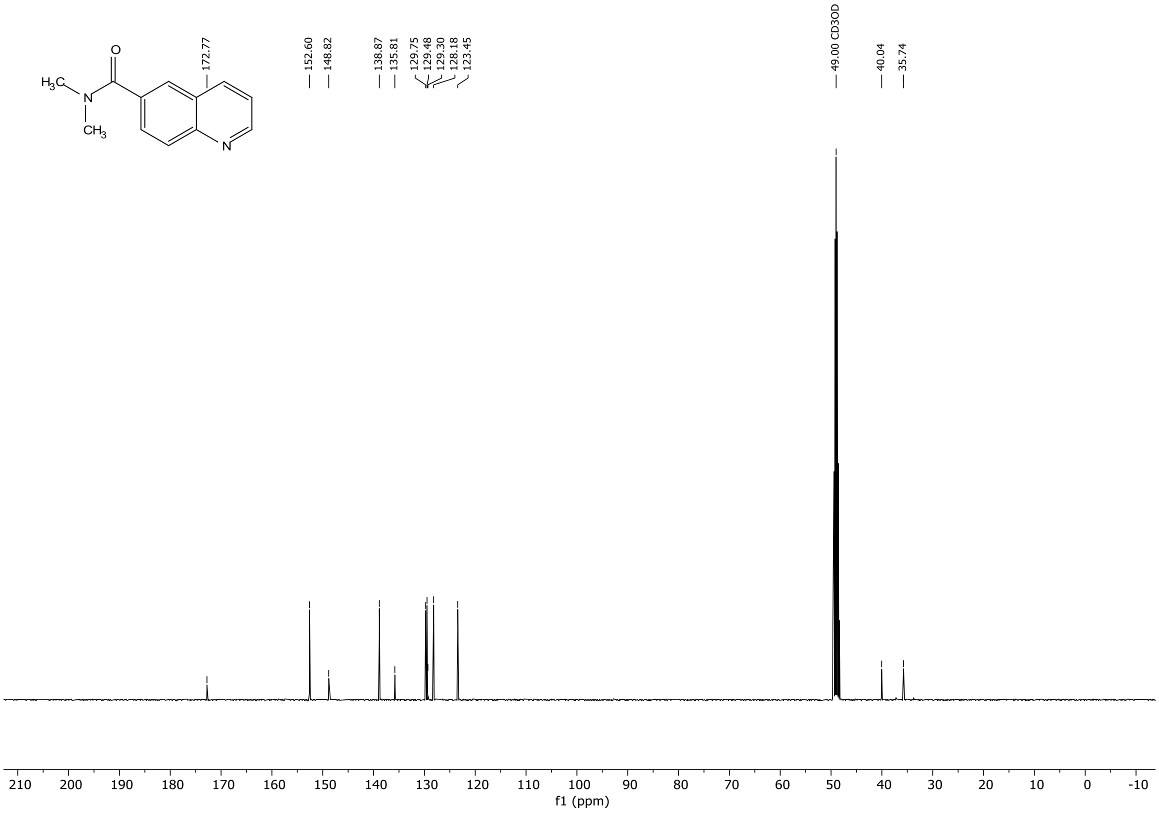

^{1}H NMR spectrum (400 MHz, $CDCl_3$) of **2c**

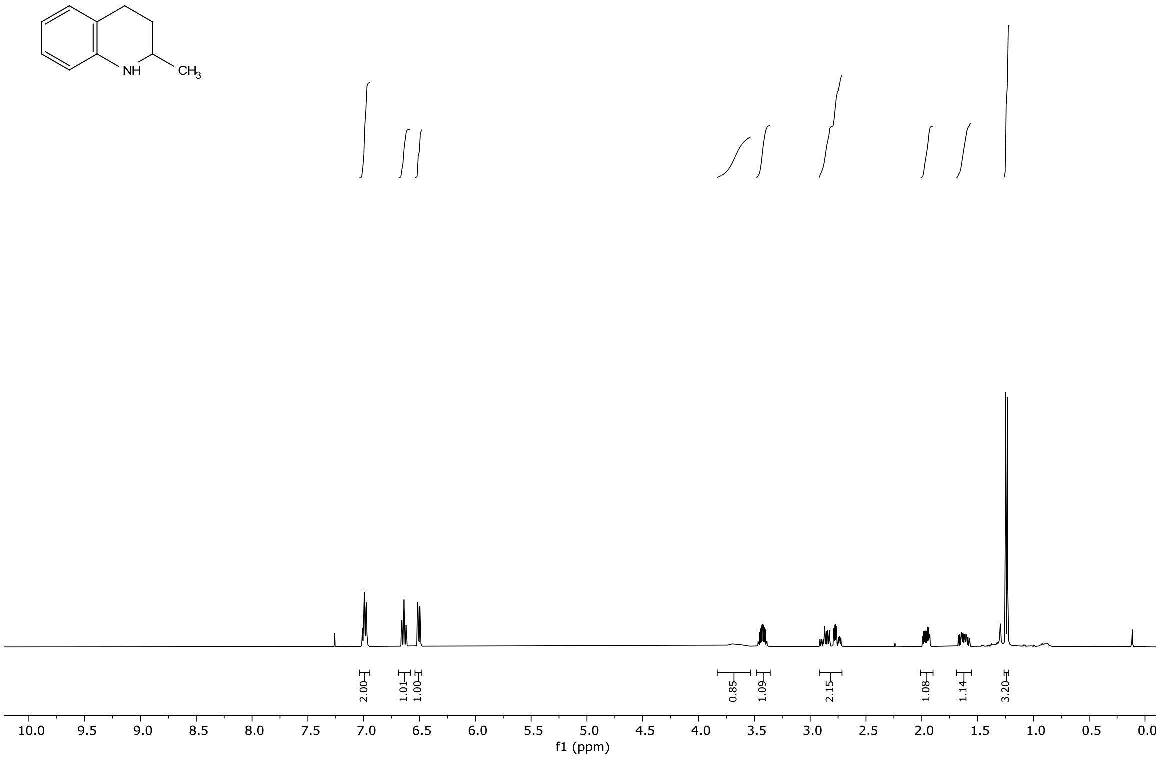

^{13}C NMR spectrum (400 MHz, $CDCl_3$) of **2c**

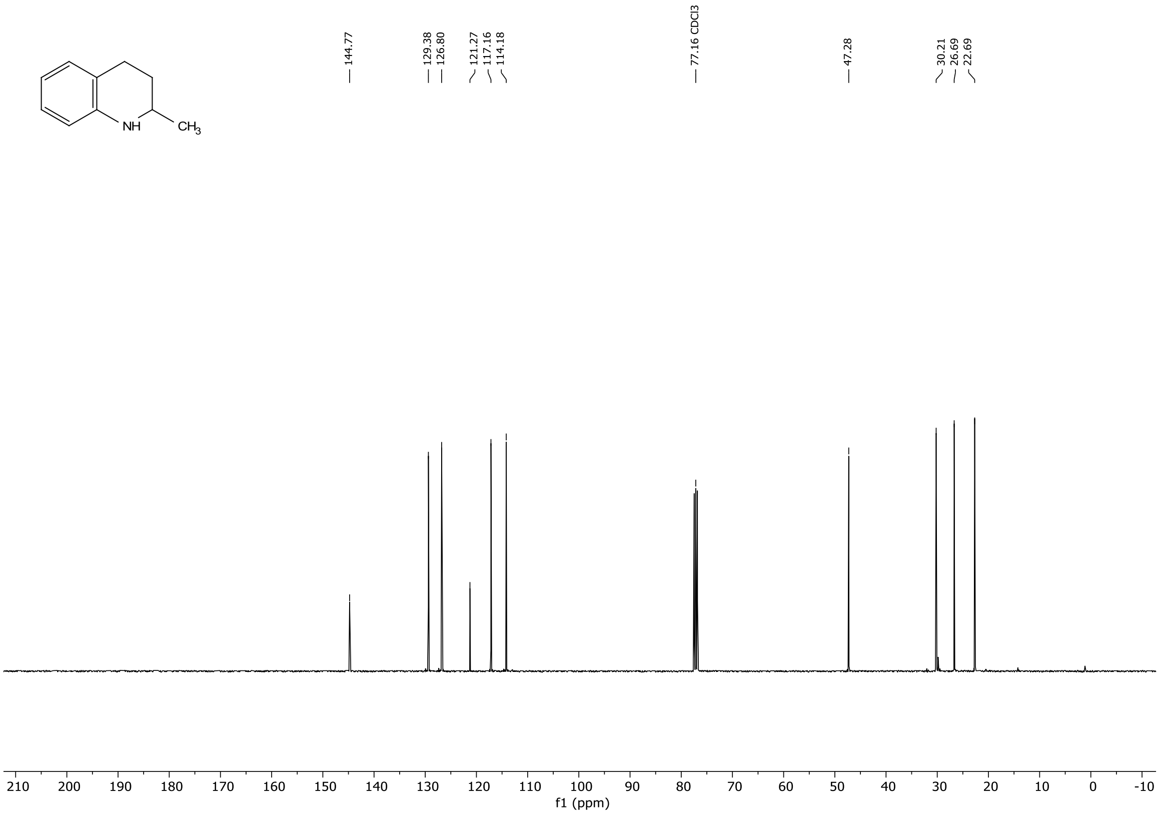

^{1}H NMR spectrum (400 MHz, $CDCl_3$) of **2d**

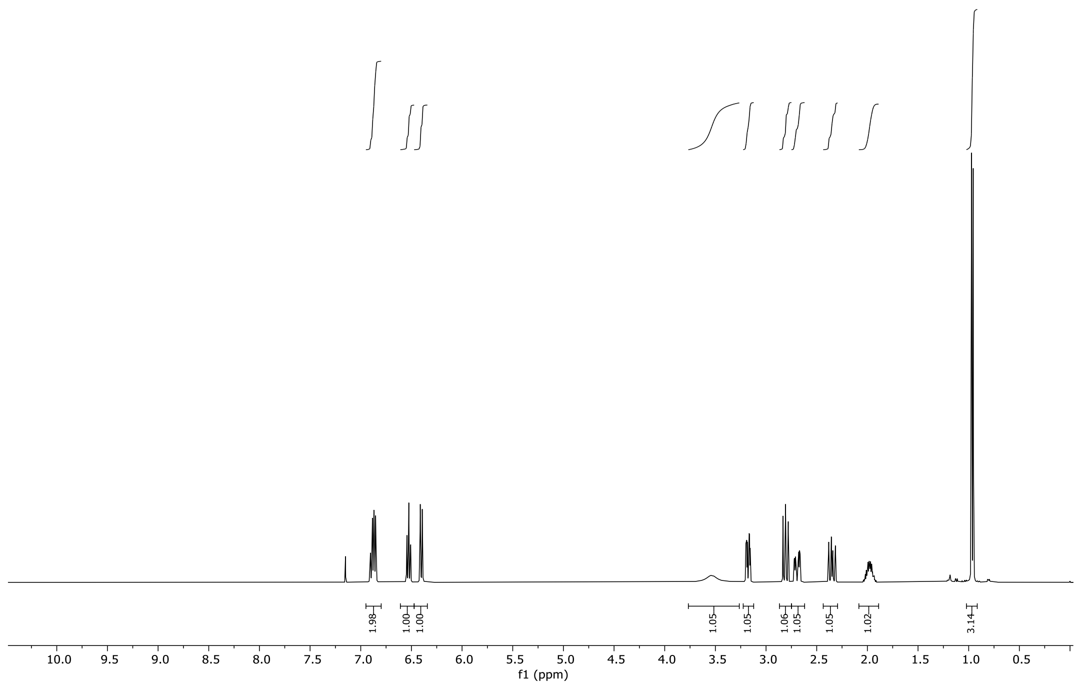

^{13}C NMR spectrum (400 MHz, $CDCl_3$) of **2d**

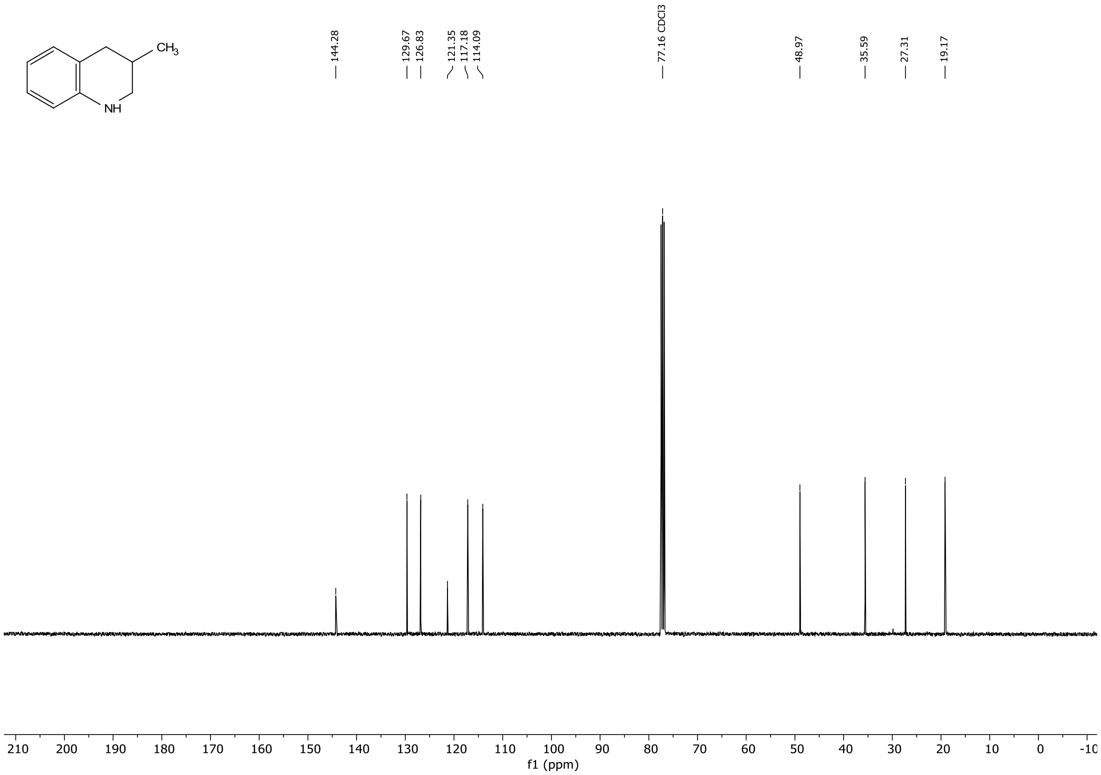

^{1}H NMR spectrum (400 MHz, $CDCl_3$) of **2e**

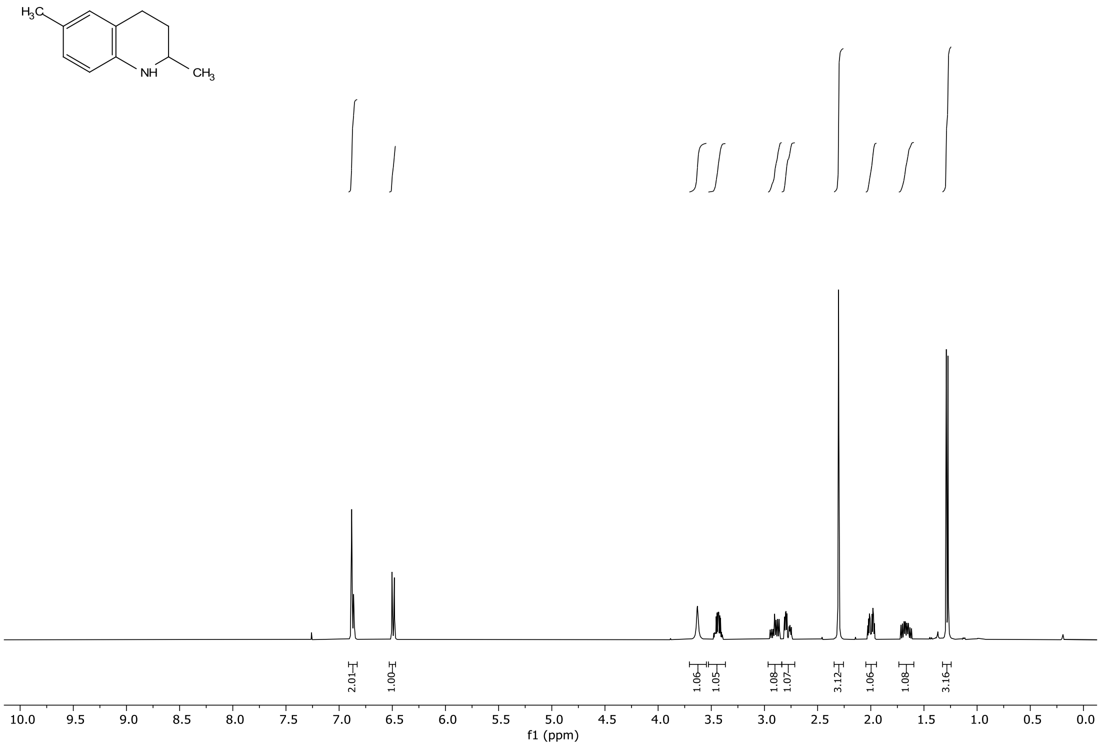

^{13}C NMR spectrum (400 MHz, $CDCl_3$) of **2e**

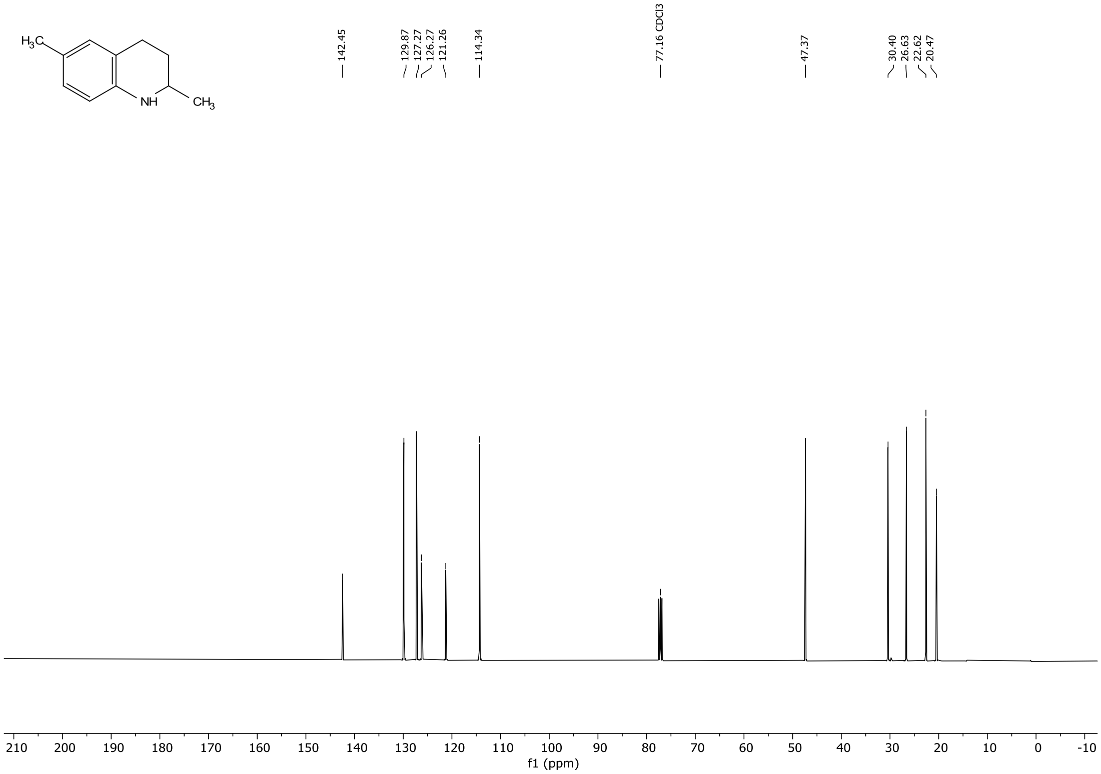

^{1}H NMR spectrum (400 MHz, CD_2Cl_2) of **2f**

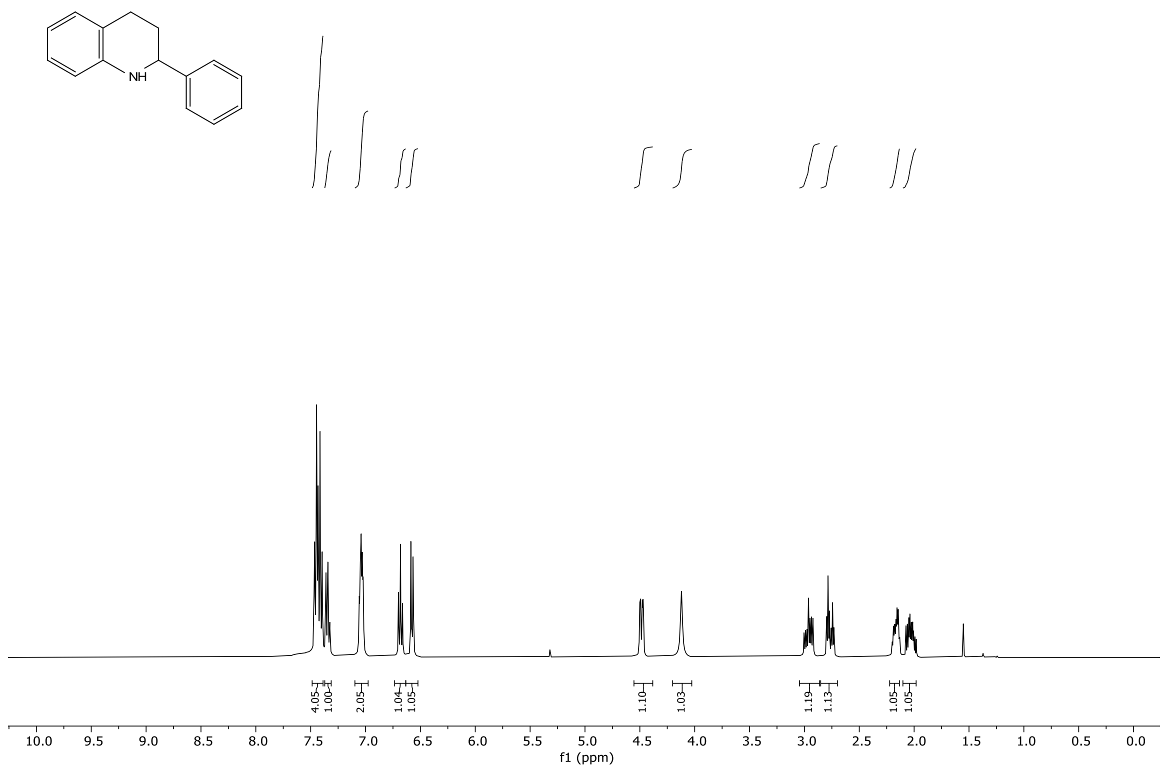

^{13}C NMR spectrum (400 MHz, CD_2Cl_2) of **2f**

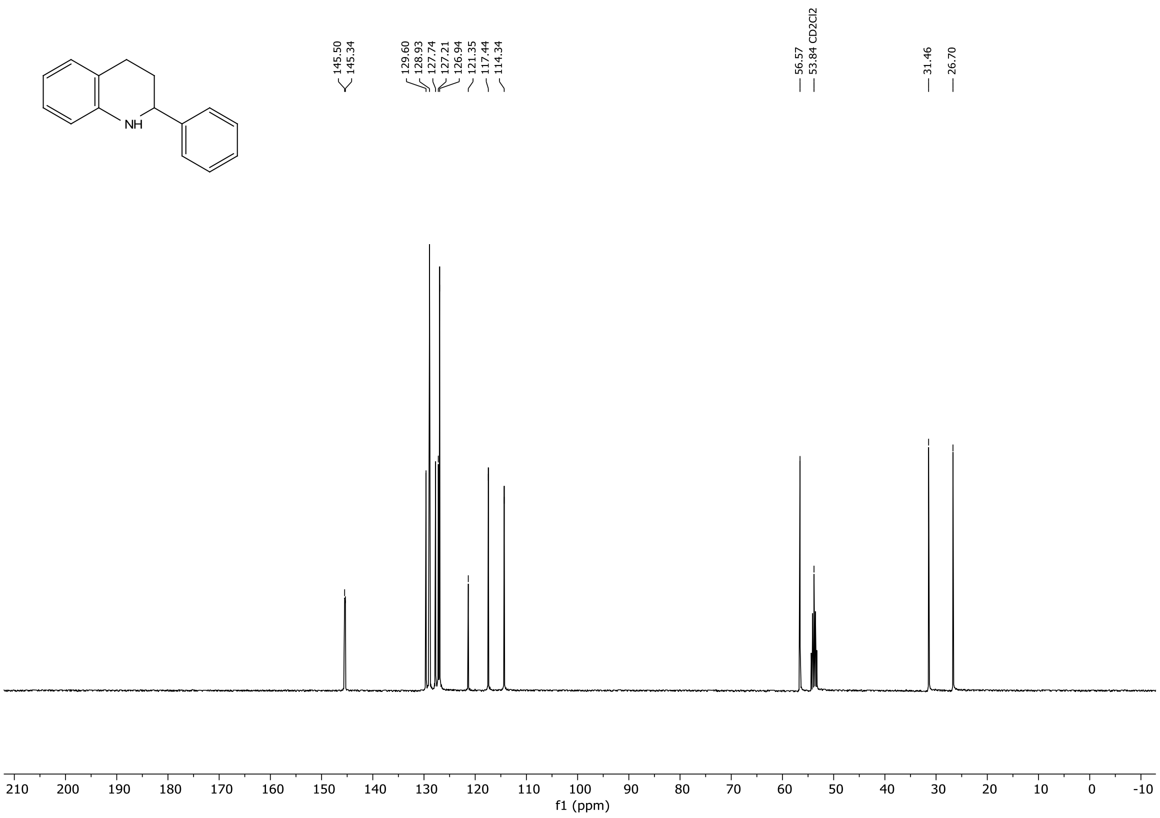

^{1}H NMR spectrum (400 MHz, $CDCl_3$) of **2g**

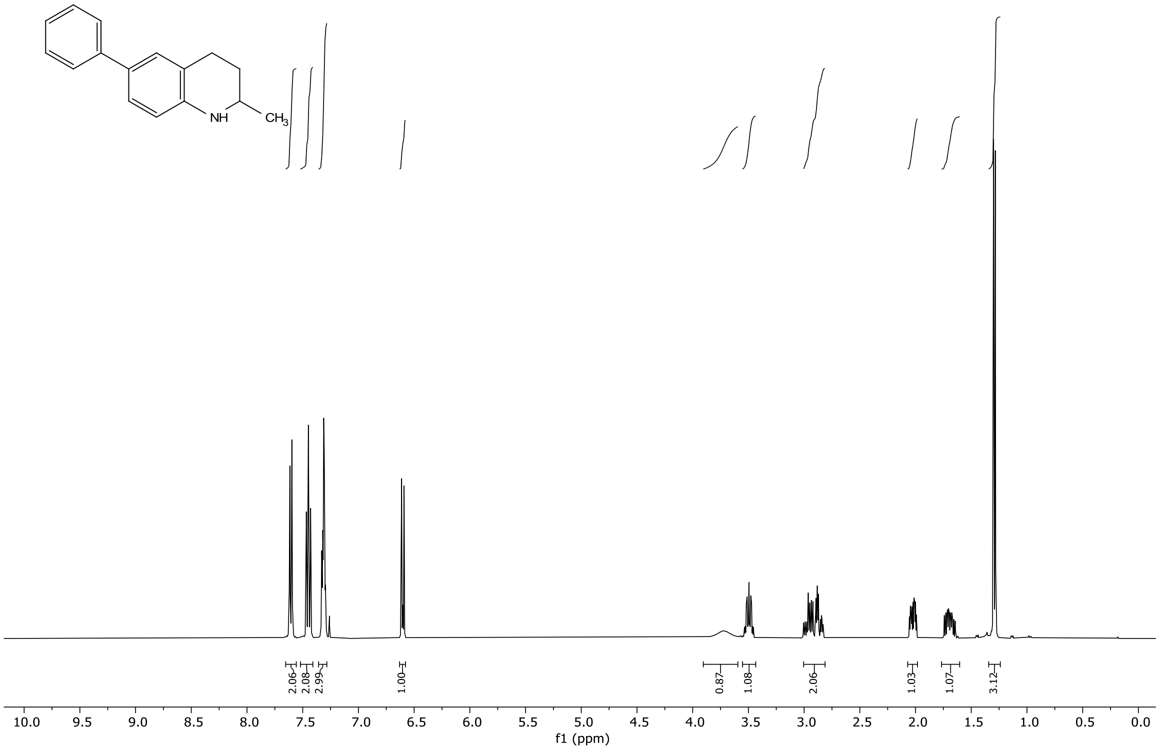

^{13}C NMR spectrum (400 MHz, $CDCl_3$) of **2g**

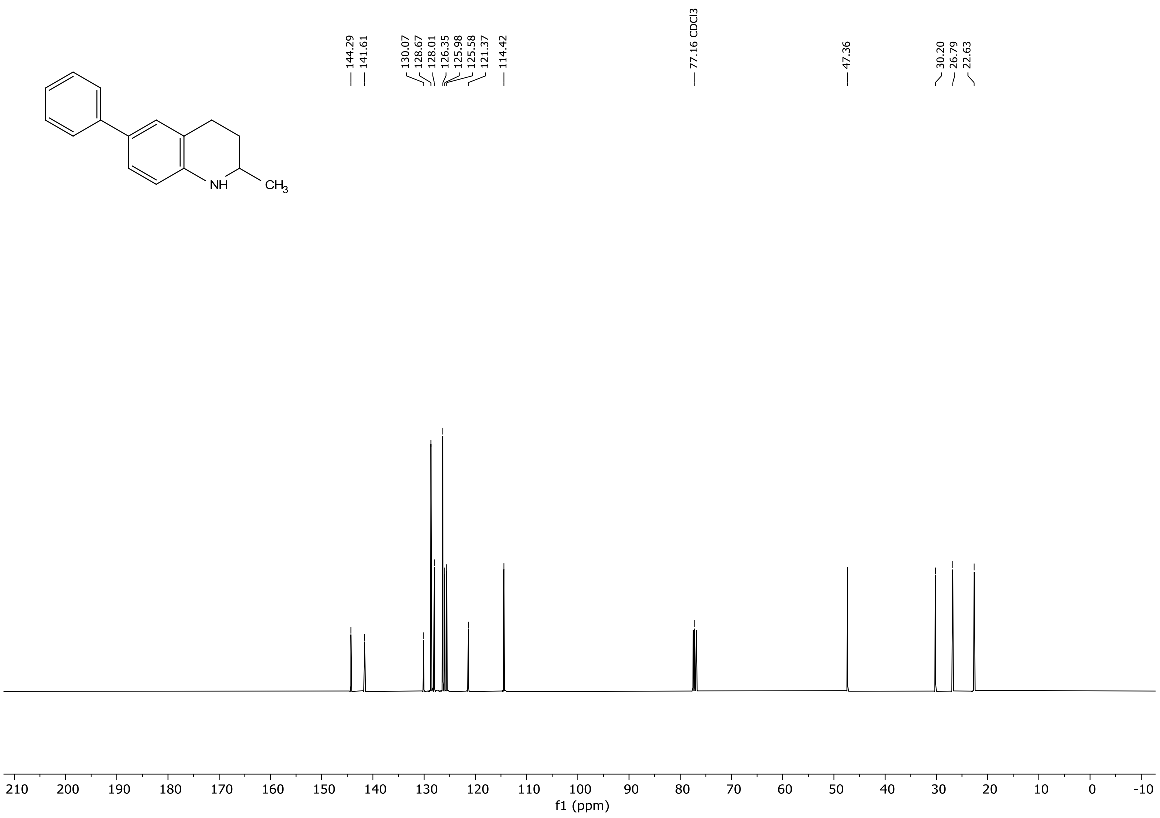

^{1}H NMR spectrum (400 MHz, $CDCl_3$) of **2i**

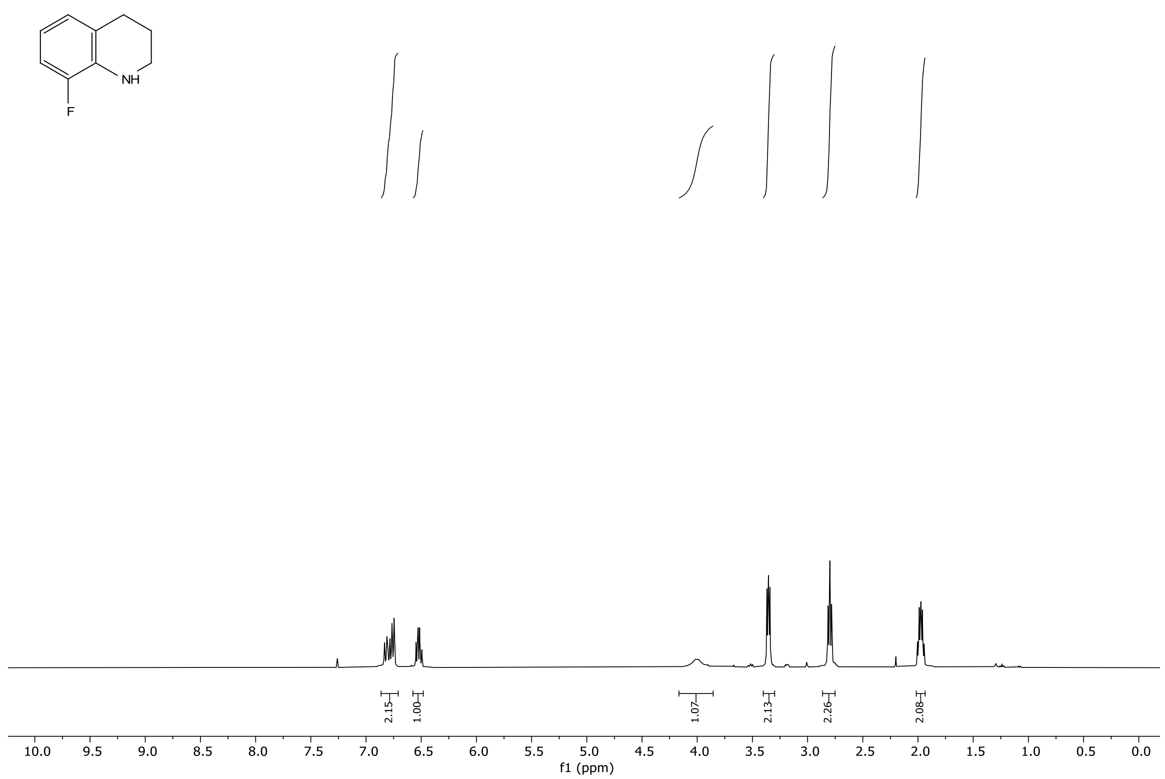

^{13}C NMR spectrum (400 MHz, $CDCl_3$) of **2i**

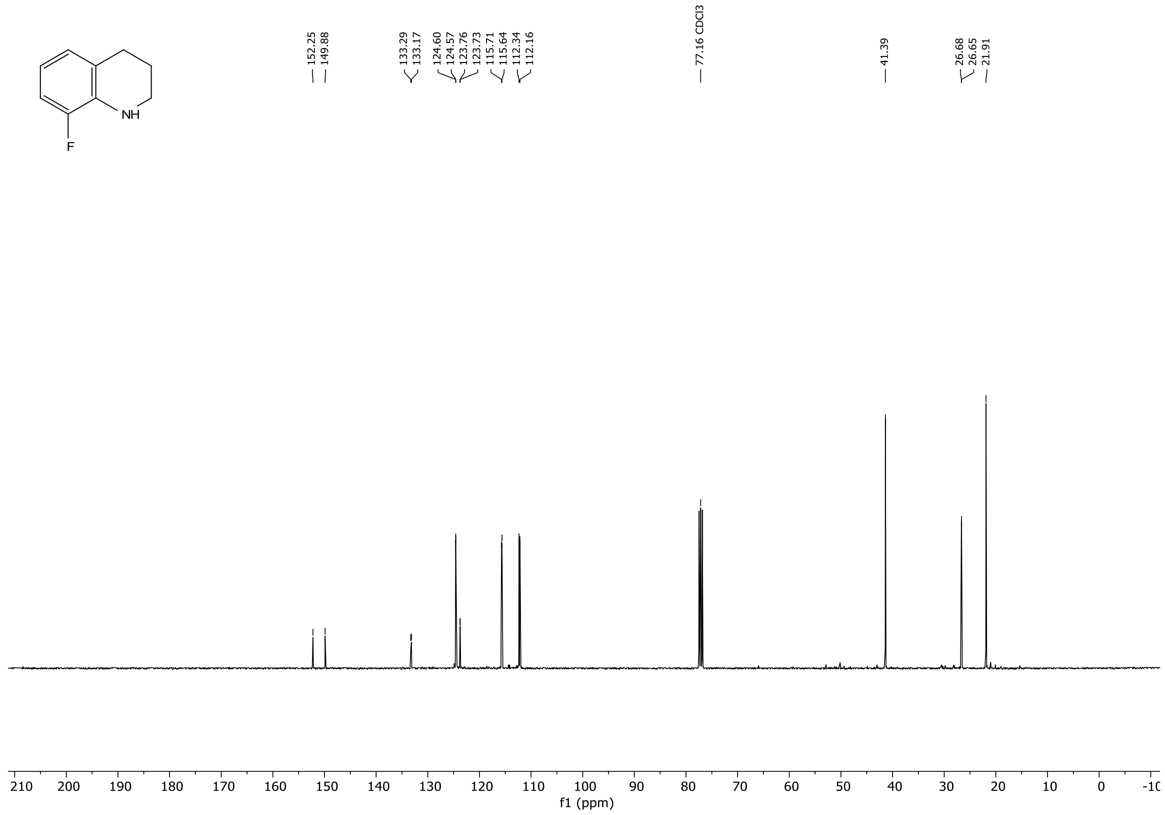

^{19}F NMR spectrum (400 MHz, $CDCl_3$) of **2i**

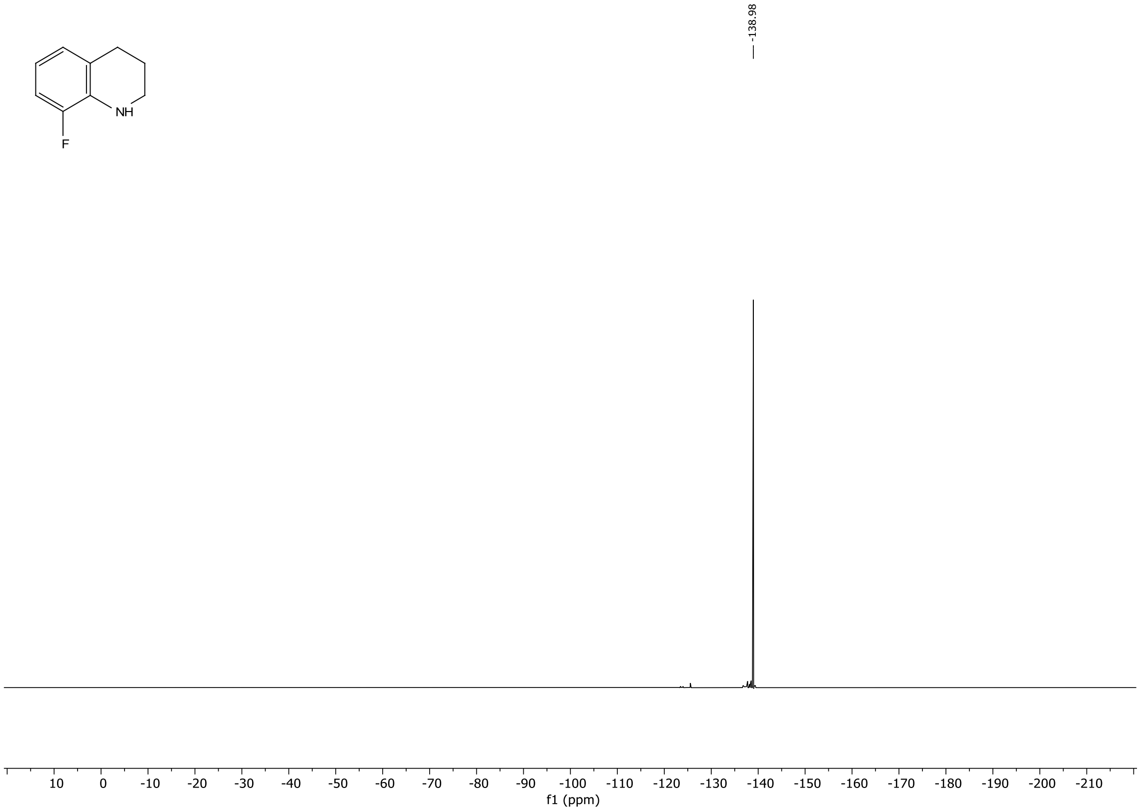

^{1}H NMR spectrum (400 MHz, $CDCl_3$) of **2j**

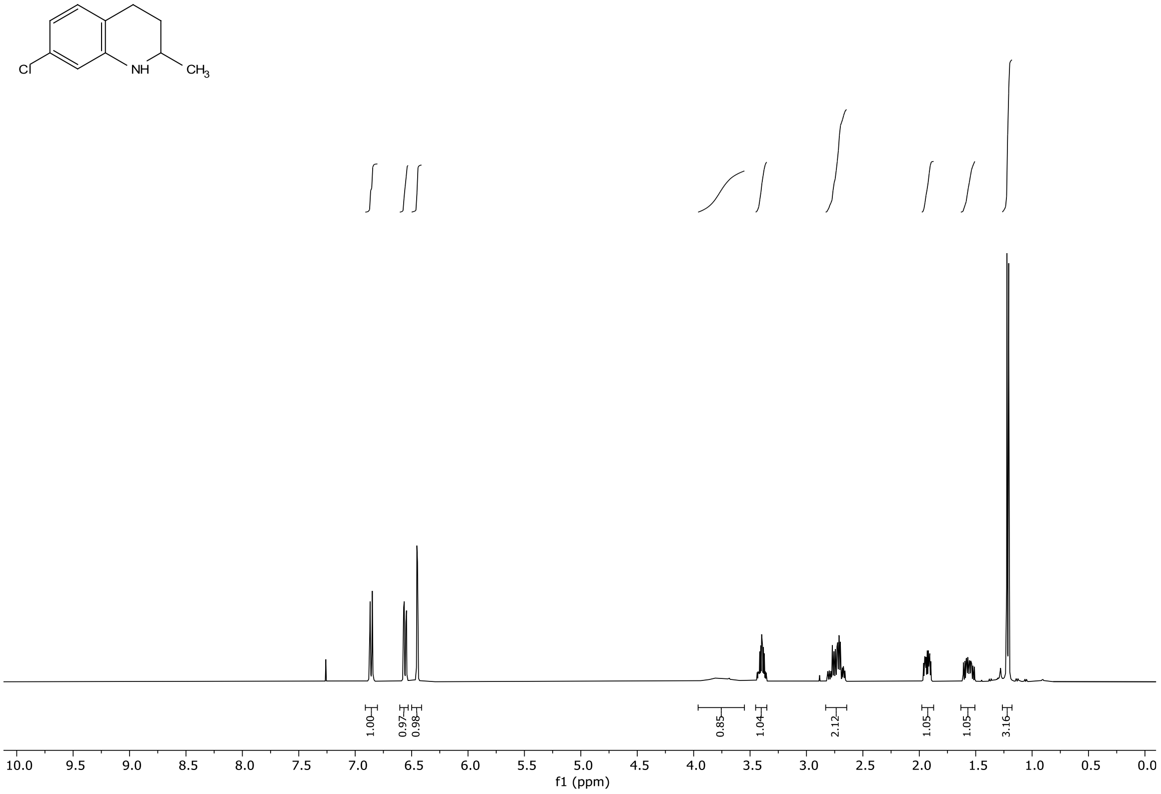

^{13}C NMR spectrum (400 MHz, $CDCl_3$) of **2j**

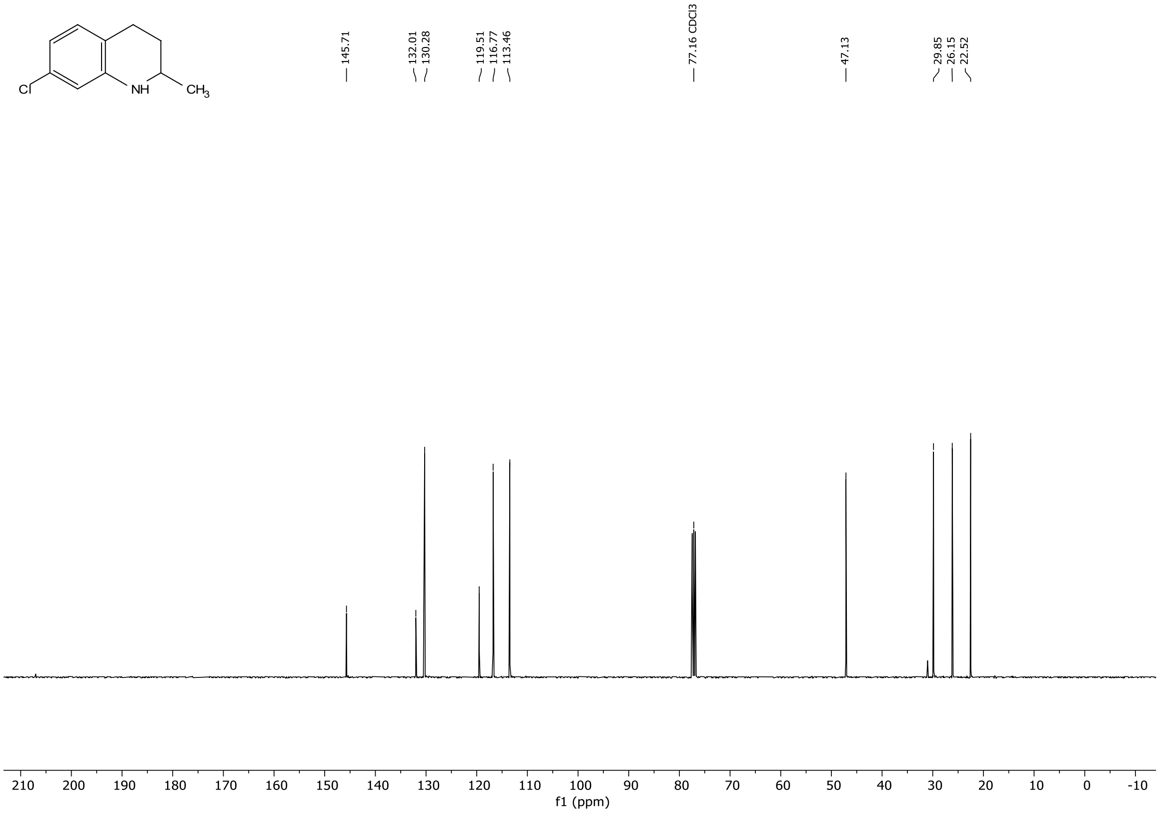

^{1}H NMR spectrum (400 MHz, CD_3CN) of **2k**

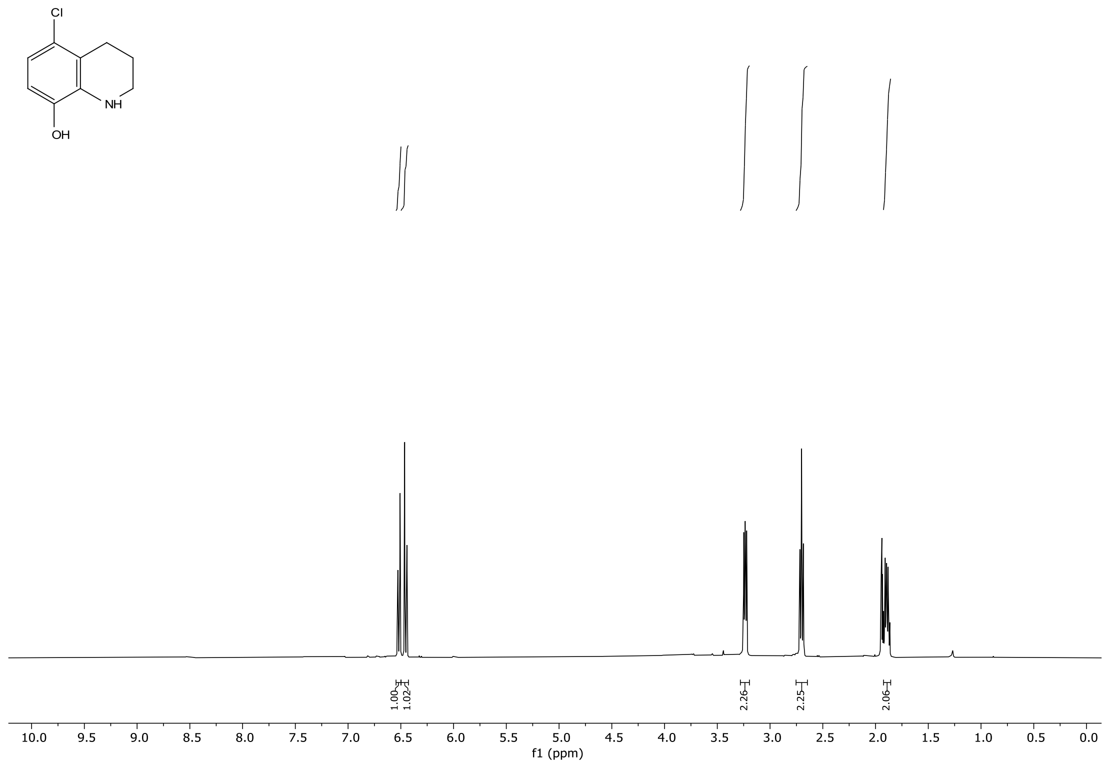

^{13}C NMR spectrum (400 MHz, CD_3CN) of **2k**

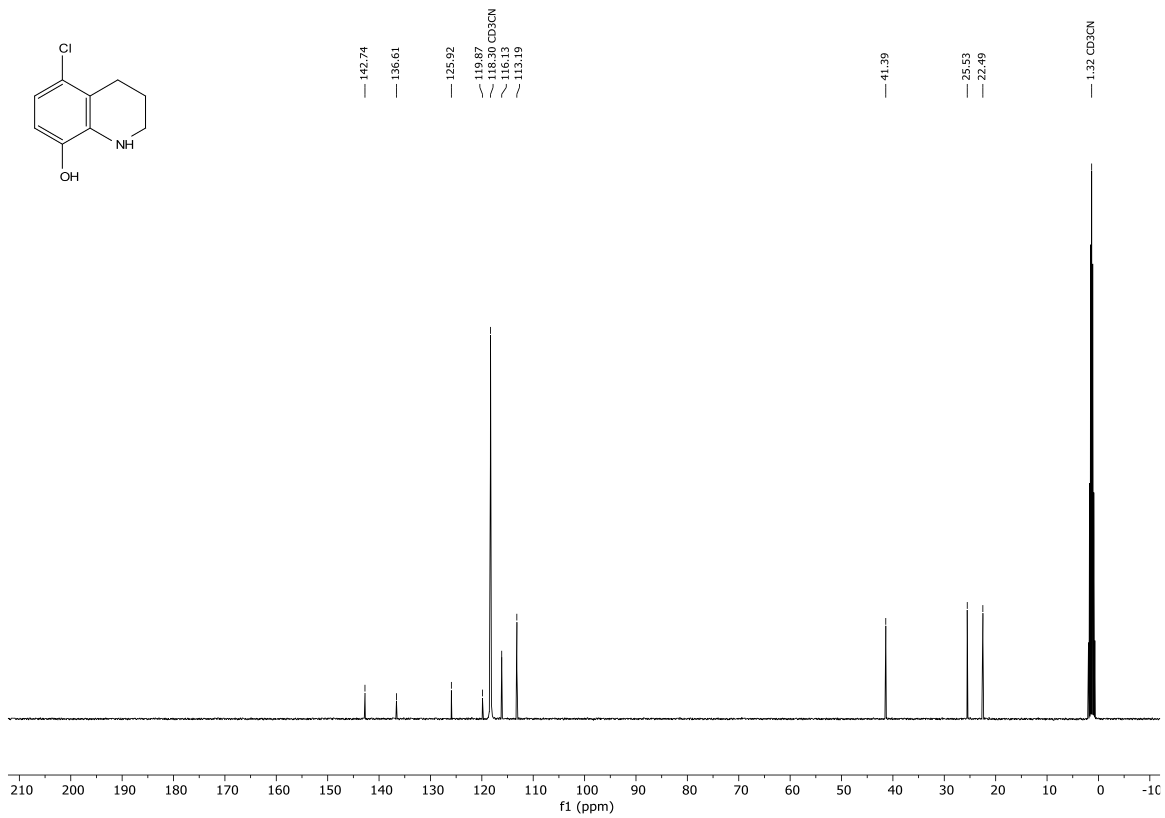

^{1}H NMR spectrum (400 MHz, $CDCl_3$) of **2l**

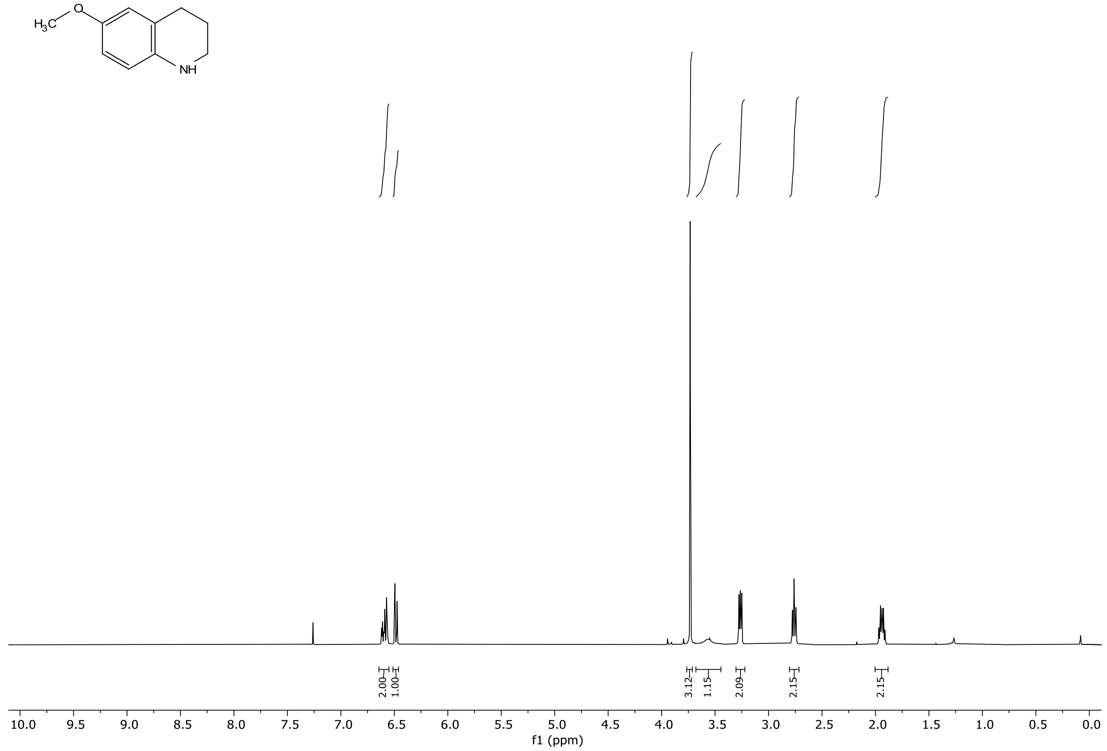

^{13}C NMR spectrum (400 MHz, $CDCl_3$) of **2l**

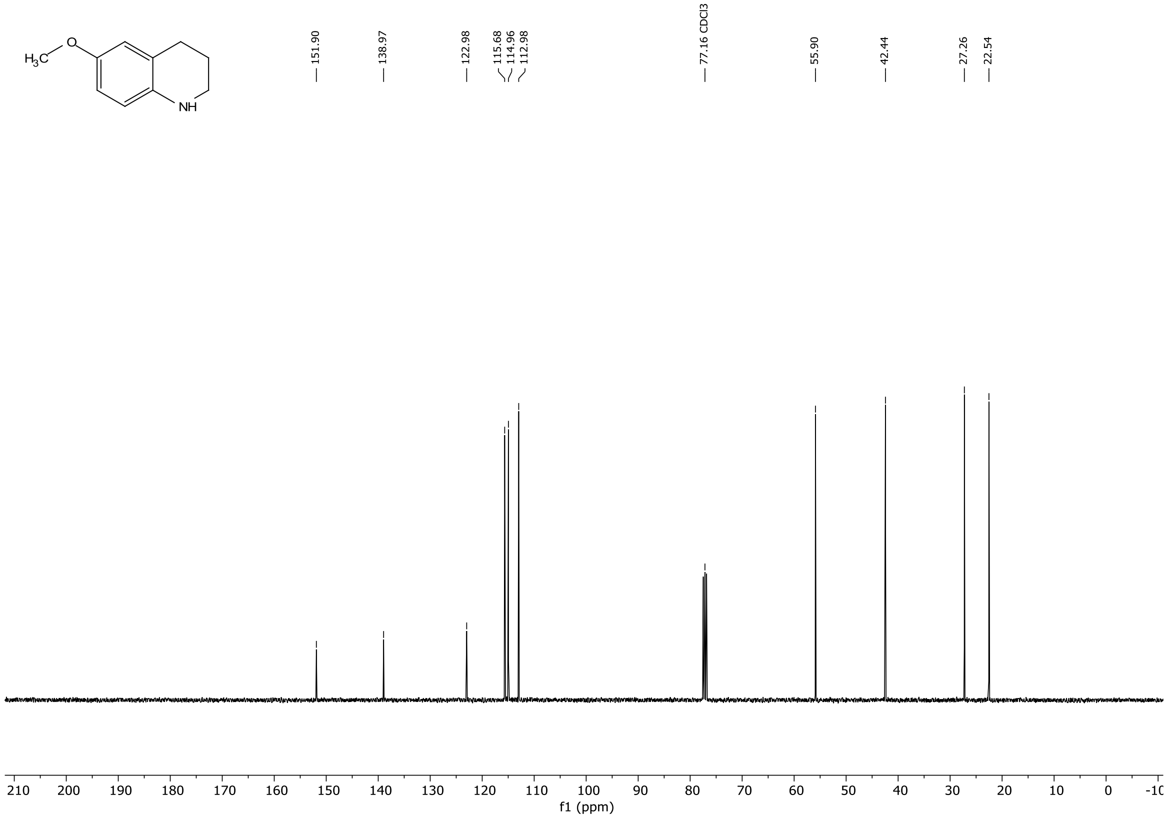

^1H NMR spectrum (400 MHz, $CDCl_3$) of **2m**

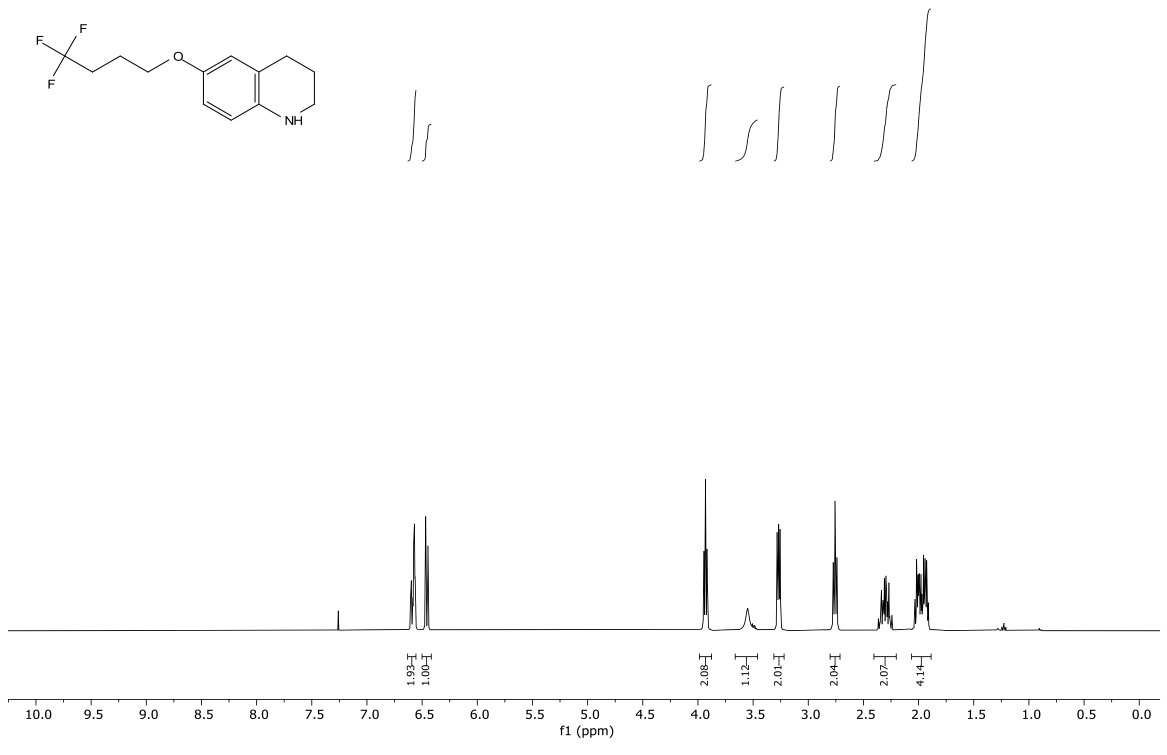

^{13}C NMR spectrum (400 MHz, $CDCl_3$) of **2m**

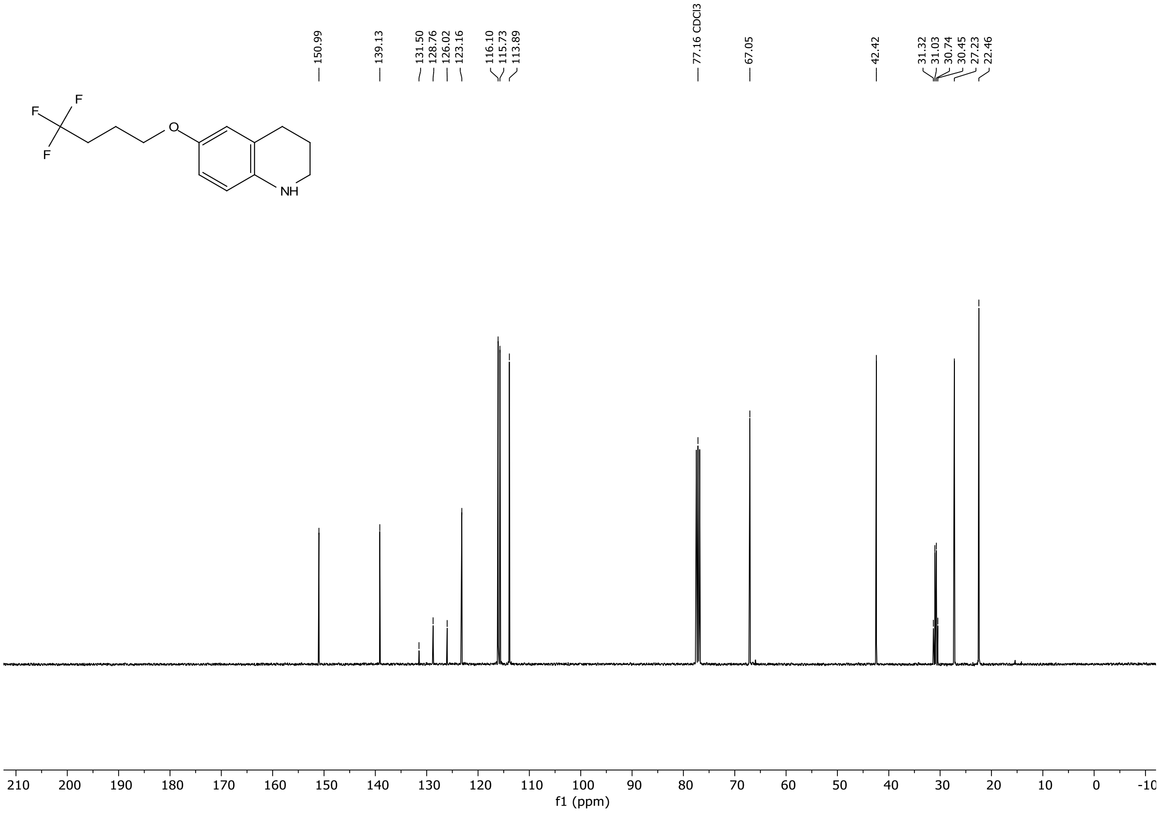

^{19}F NMR spectrum (400 MHz, $CDCl_3$) of **2m**

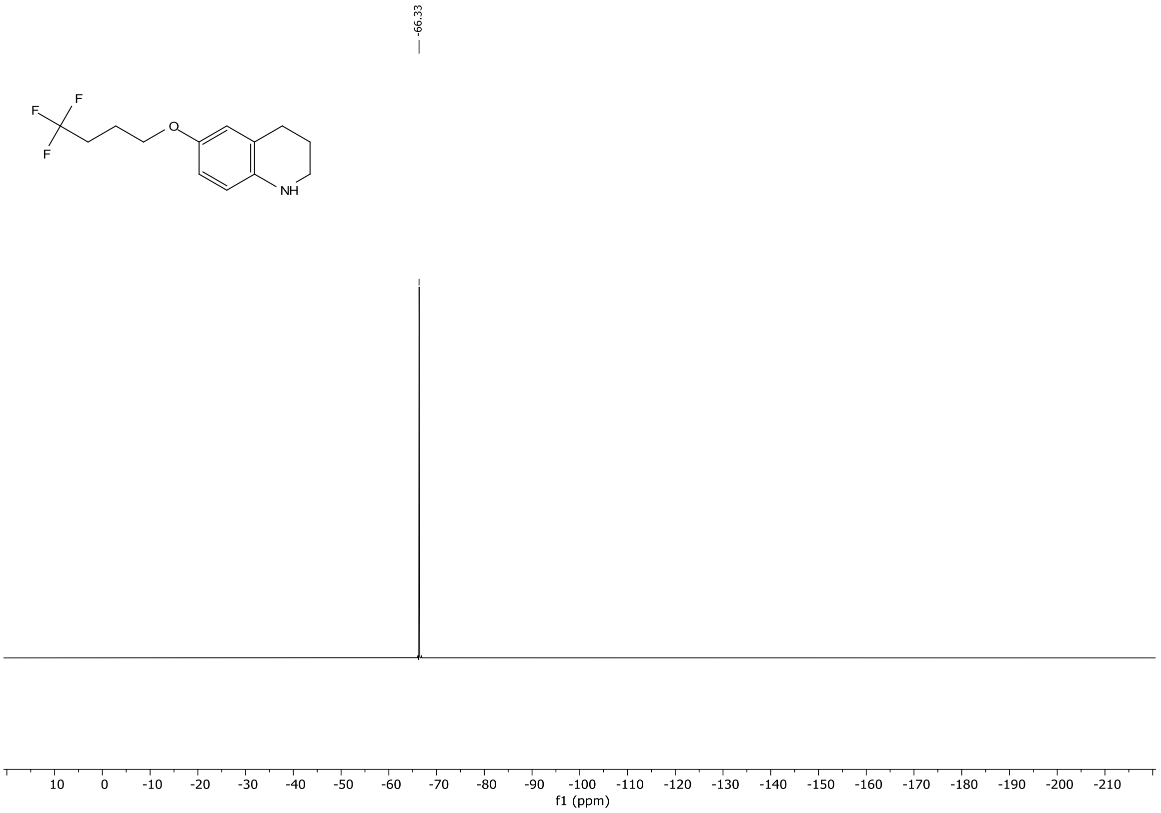

^{1}H NMR spectrum (400 MHz, CD_2Cl_2) of **2n**

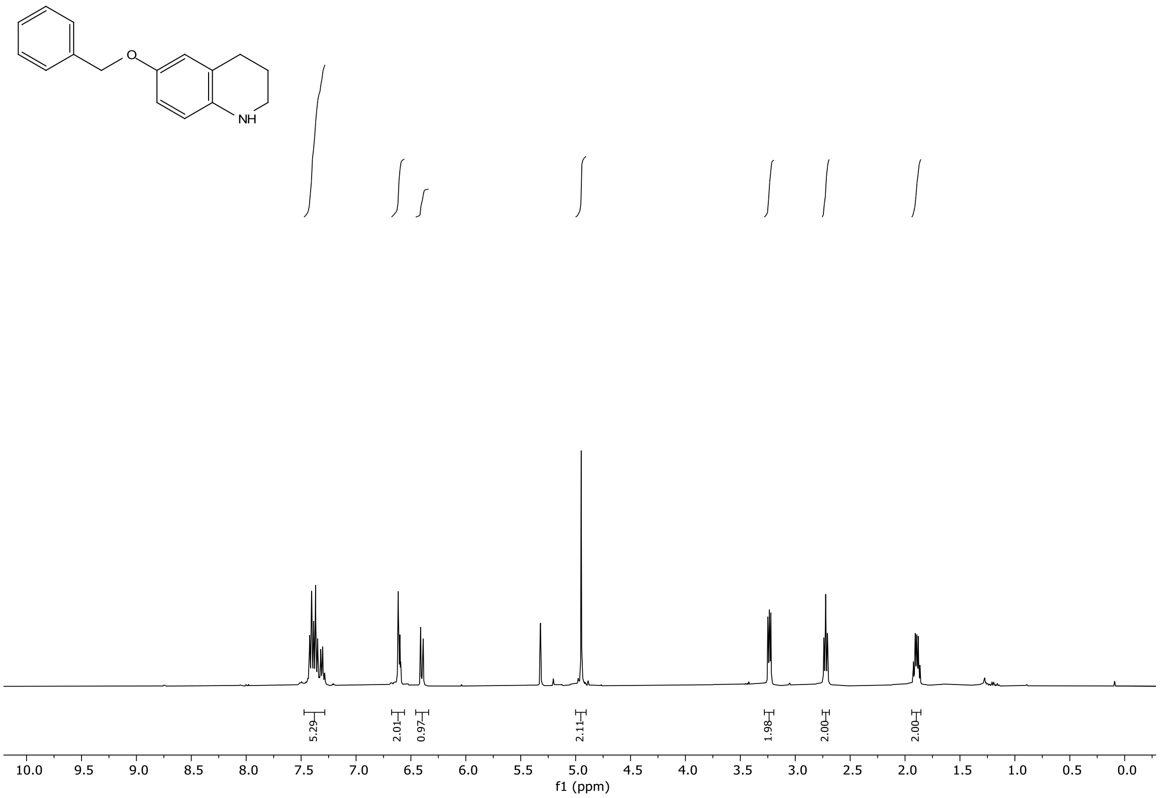

^{13}C NMR spectrum (400 MHz, CD_2Cl_2) of **2n**

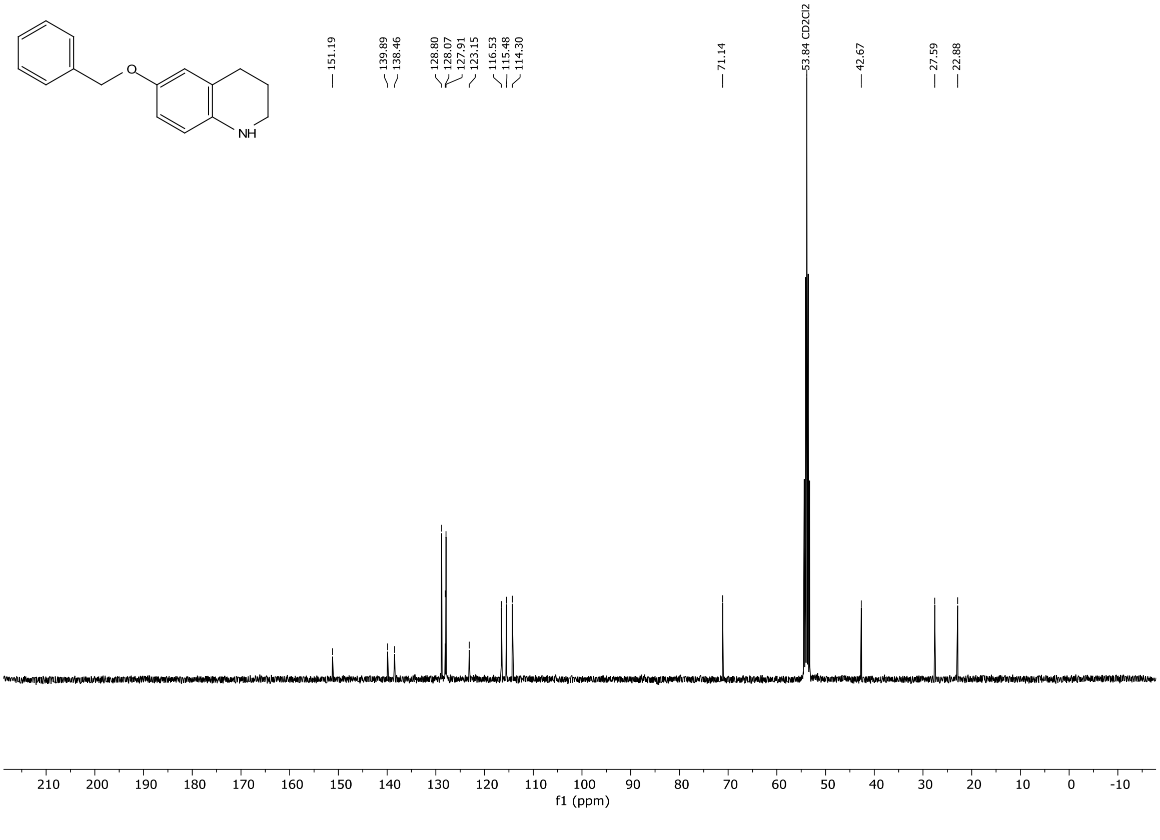

^{1}H NMR spectrum (400 MHz, $CDCl_3$) of **2o**

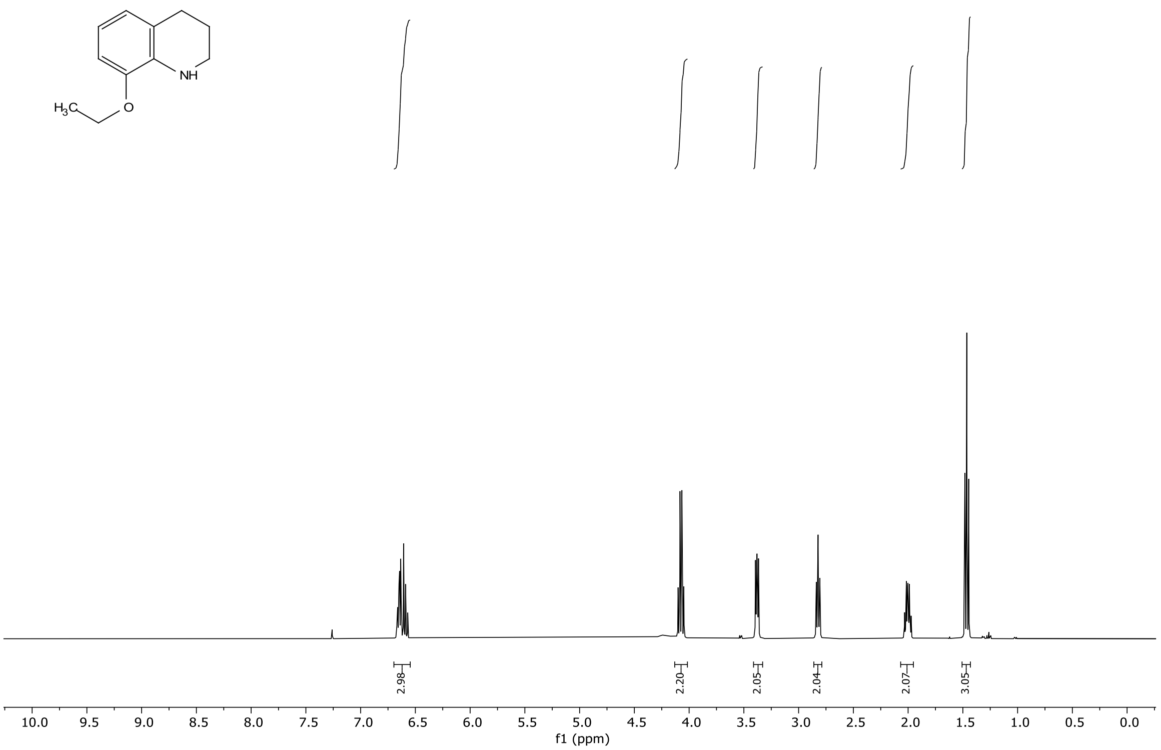

^{13}C NMR spectrum (400 MHz, $CDCl_3$) of **2o**

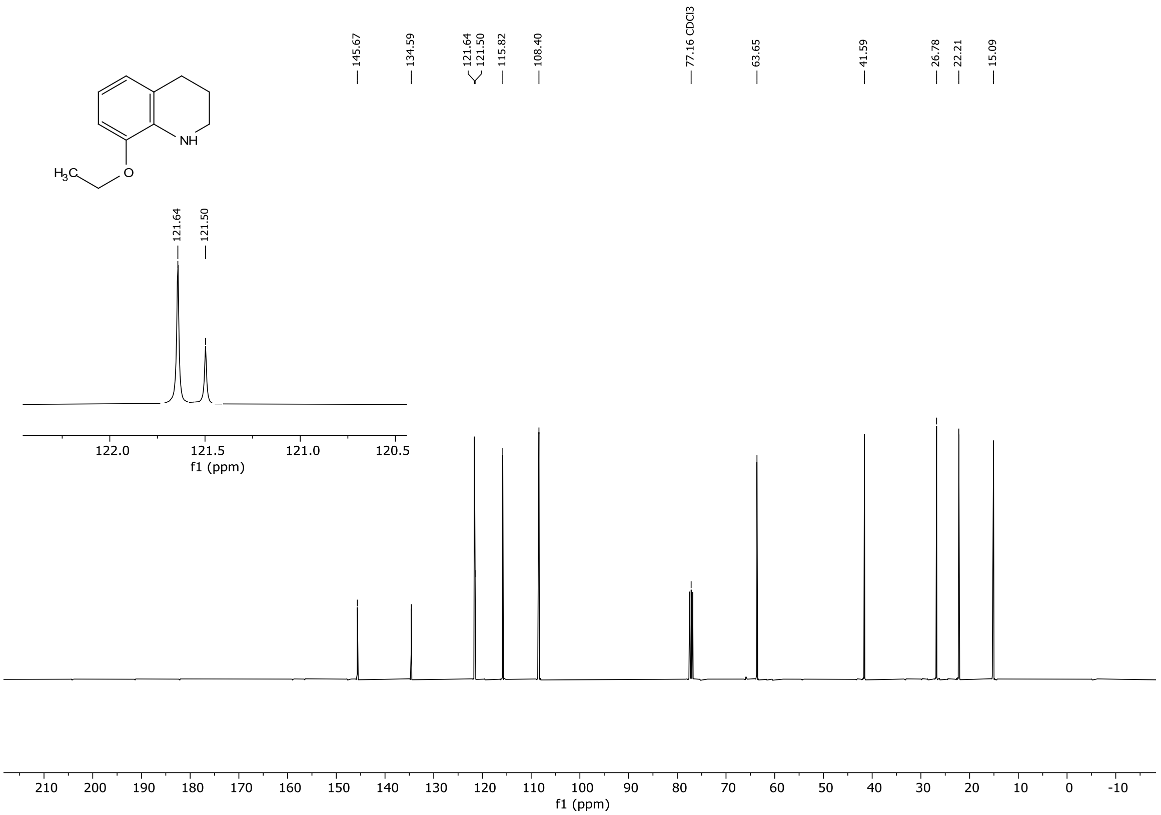

^{1}H NMR spectrum (400 MHz, CD_2Cl_2) of **2p**

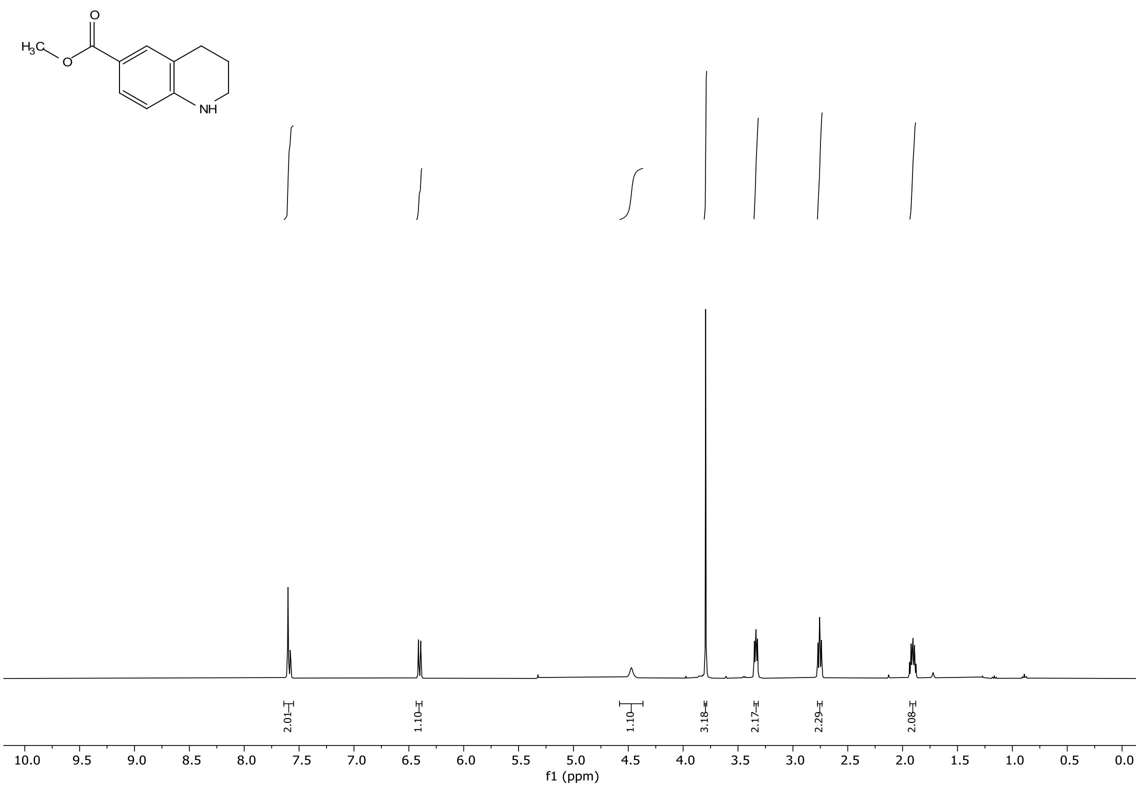

^{13}C NMR spectrum (400 MHz, CD_2Cl_2) of **2p**

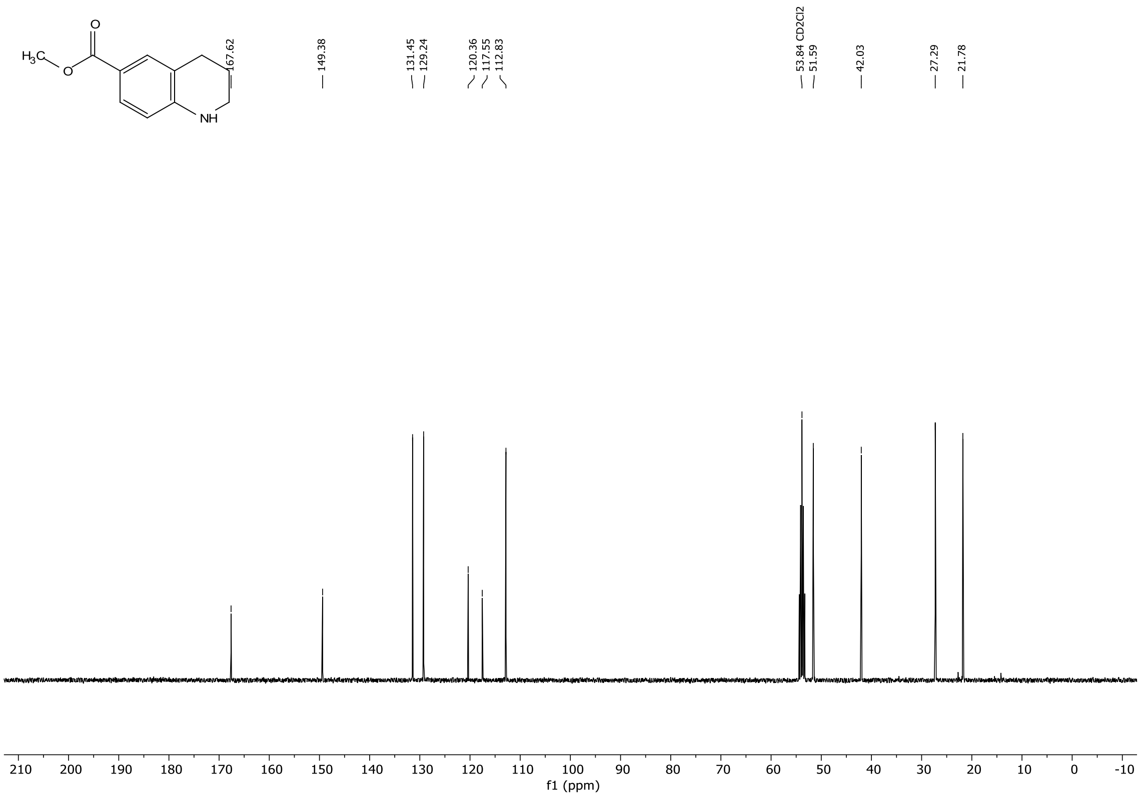

^{1}H NMR spectrum (400 MHz, CD_2Cl_2) of **2q**

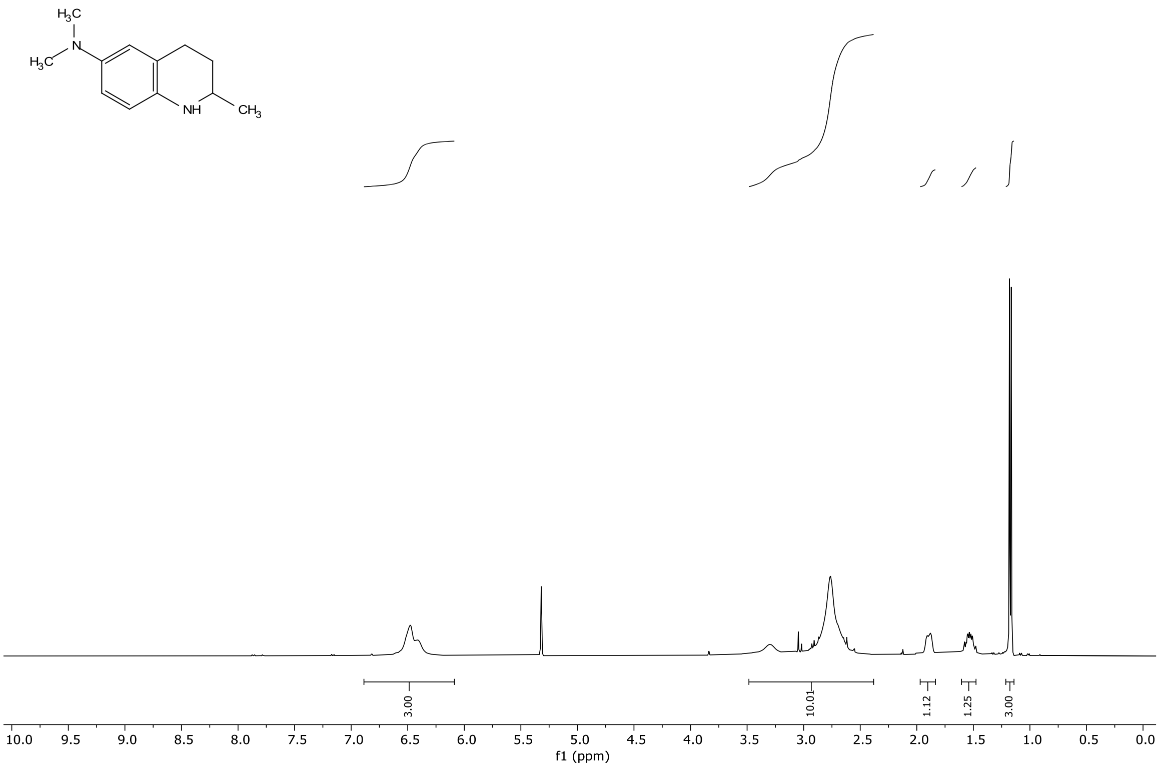

^{1}H NMR spectrum (400 MHz, CD_2Cl_2) of **2q**

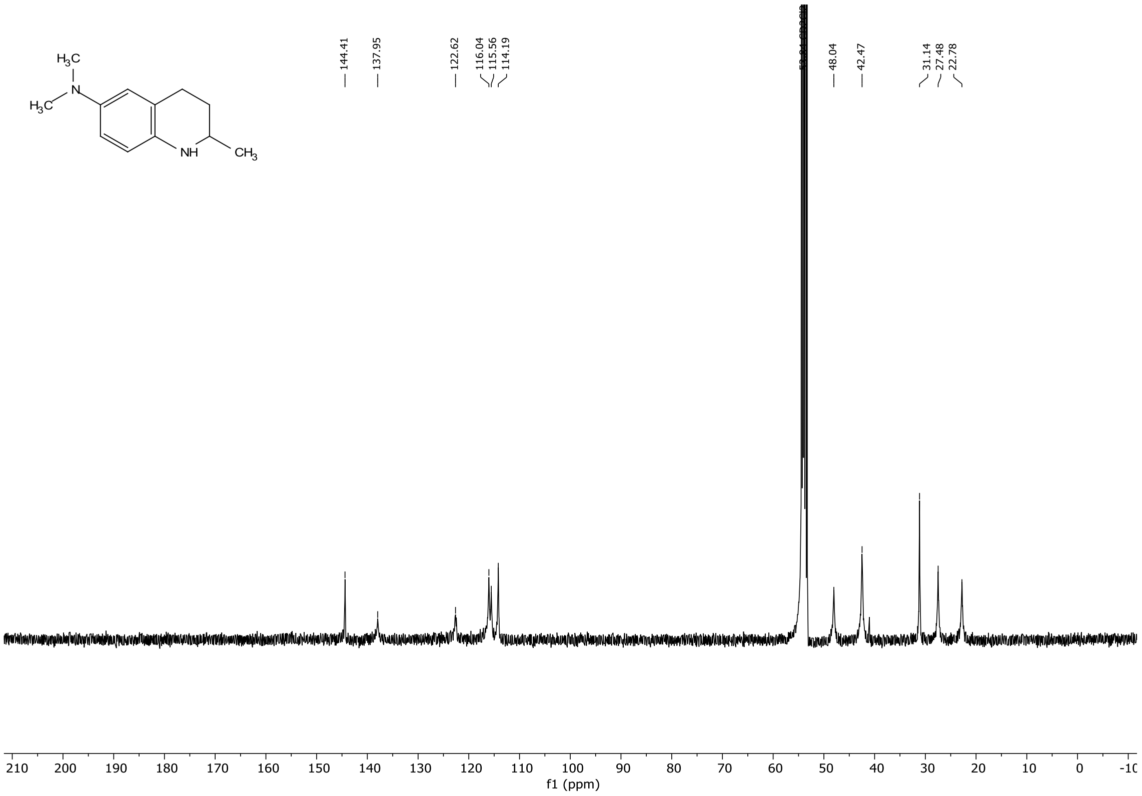

^{1}H NMR spectrum (400 MHz, CD_2Cl_2) of **2r**

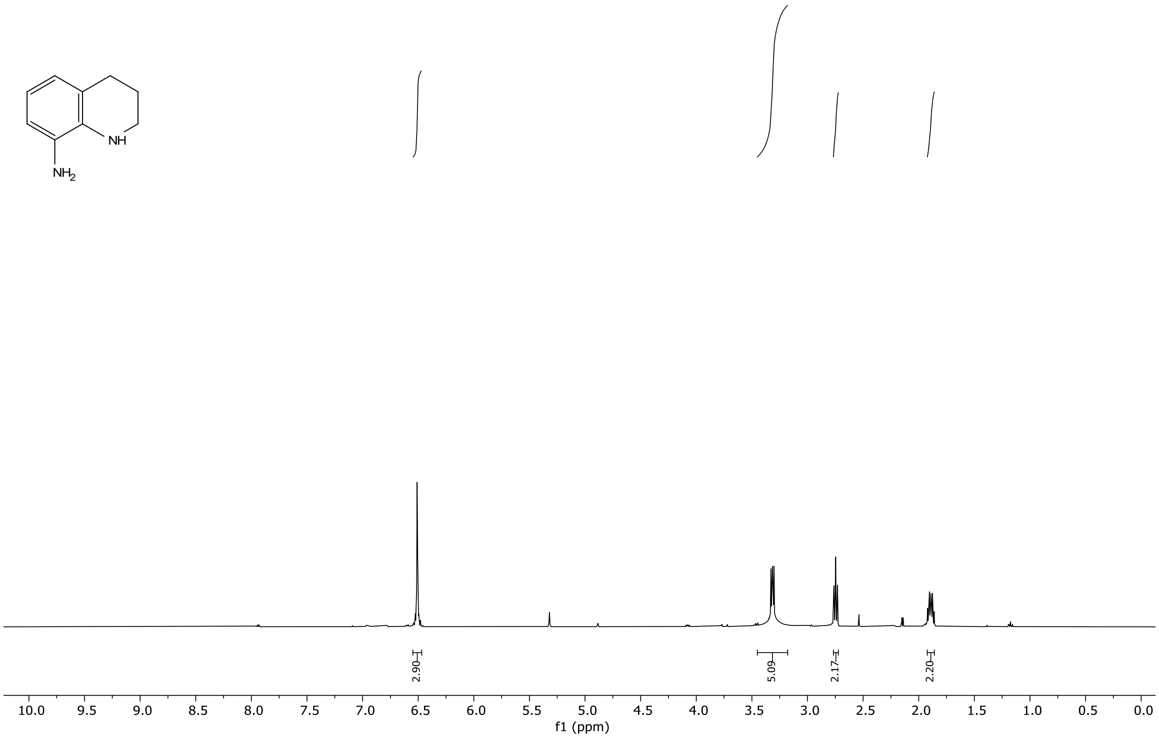

^{13}C NMR spectrum (400 MHz, CD_2Cl_2) of **2r**

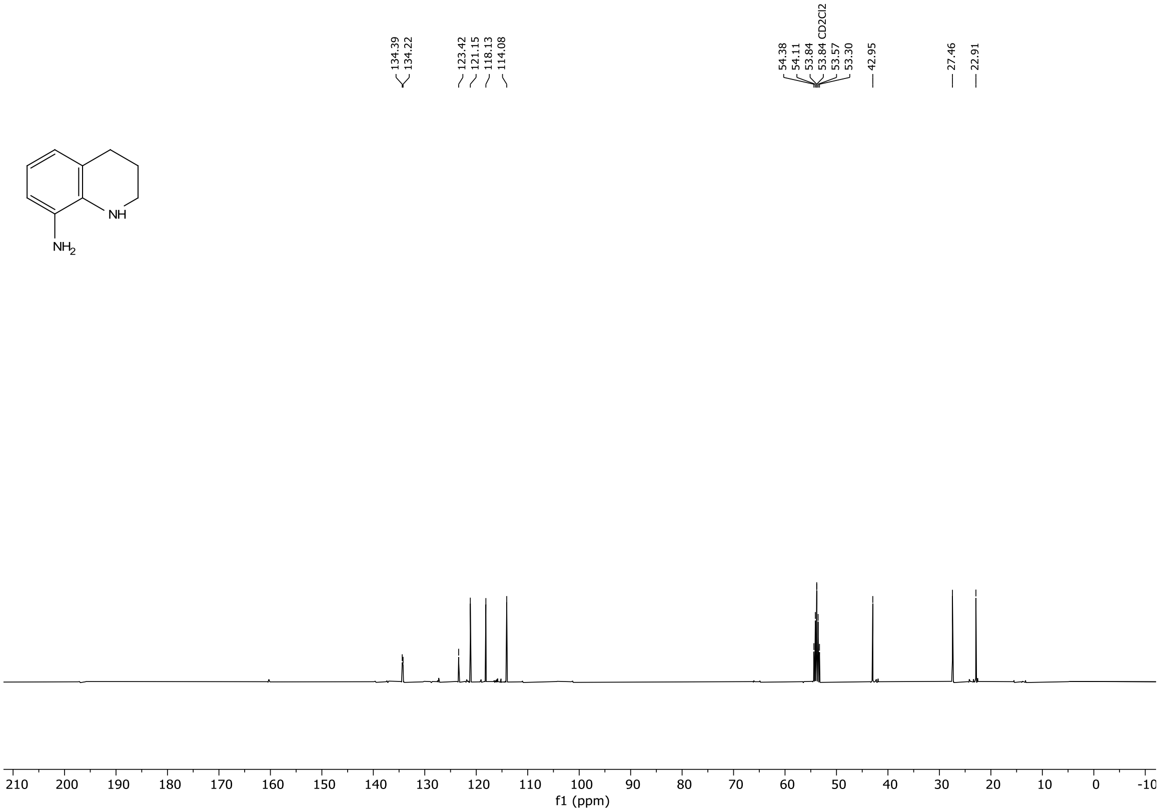

^{1}H NMR spectrum (400 MHz, CD_2Cl_2) of **2s**

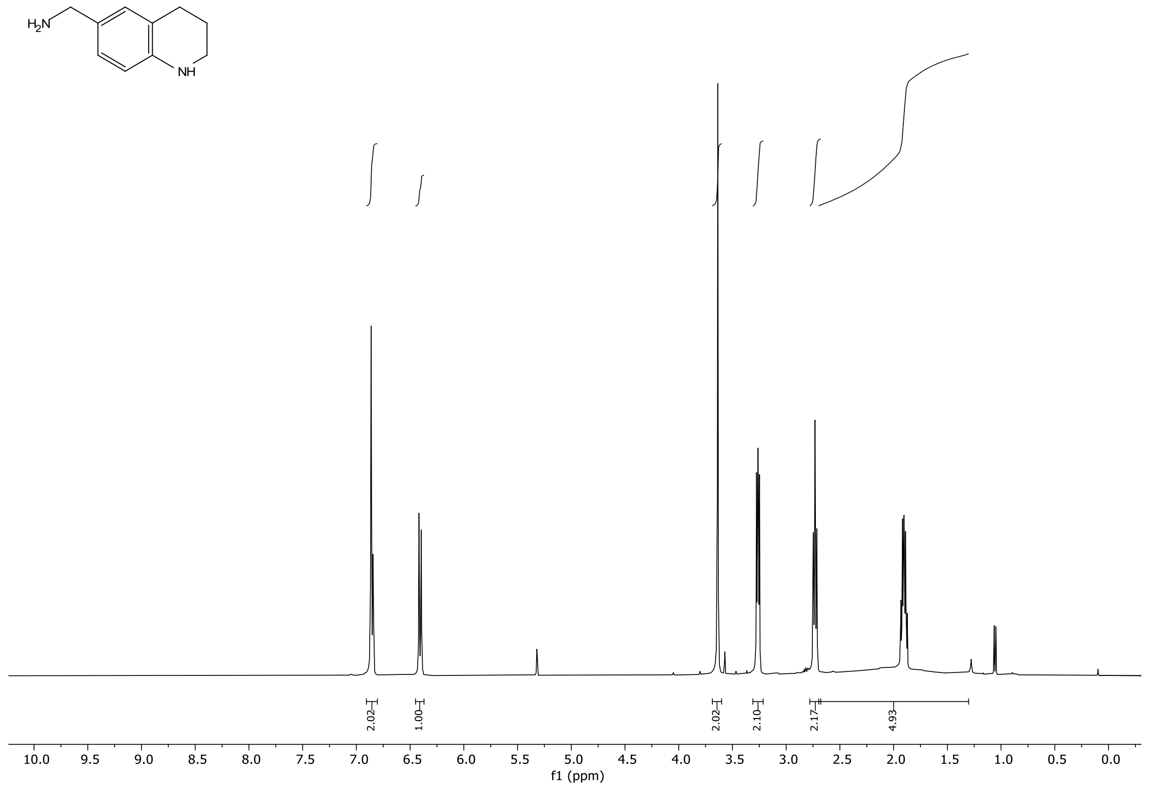

^{13}C NMR spectrum (400 MHz, CD_2Cl_2) of **2s**

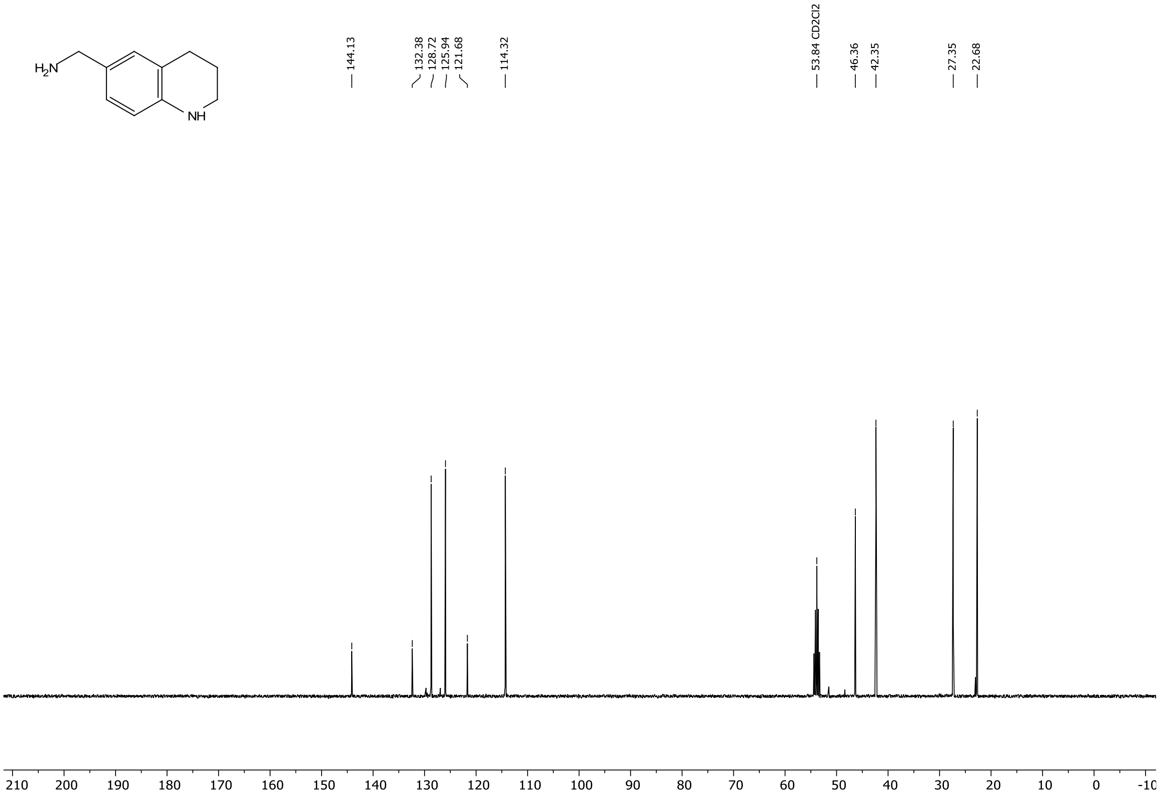

^{1}H NMR spectrum (400 MHz, $CDCl_3$) of **2t**

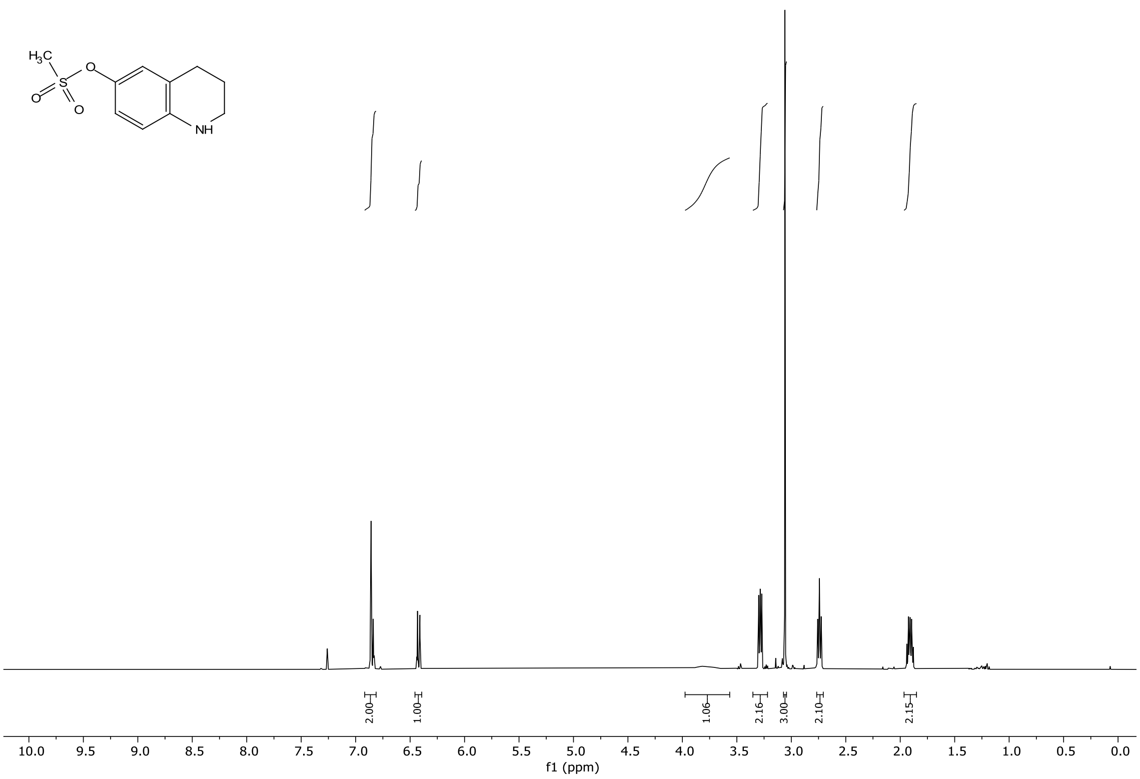

^{13}C NMR spectrum (400 MHz, $CDCl_3$) of **2t**

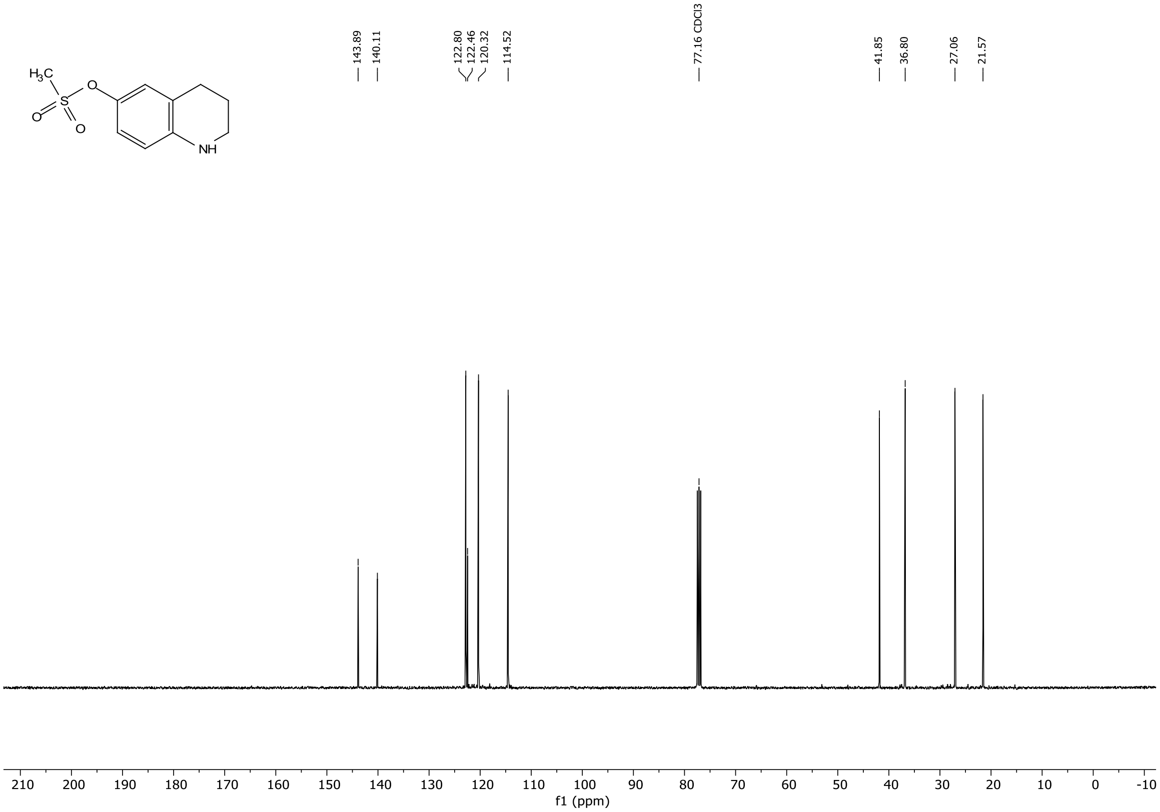

^{1}H NMR spectrum (400 MHz, CD_2Cl_2) of **2u**

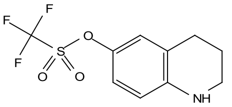

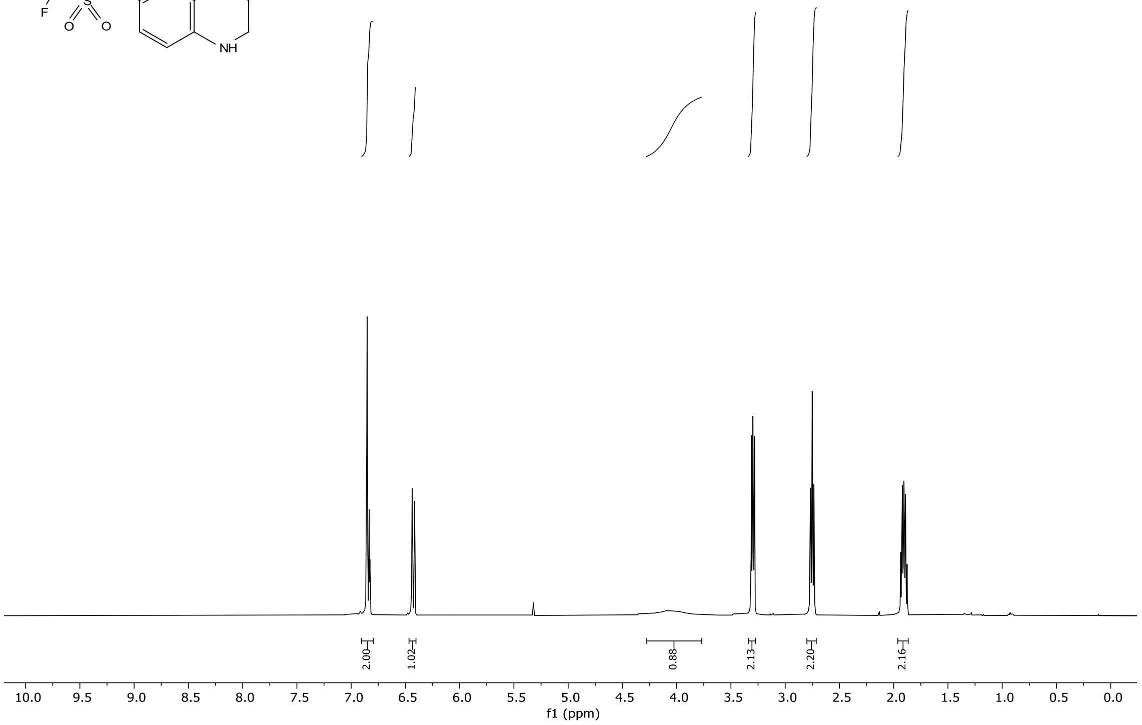

^{13}C NMR spectrum (400 MHz, CD_2Cl_2) of **2u**

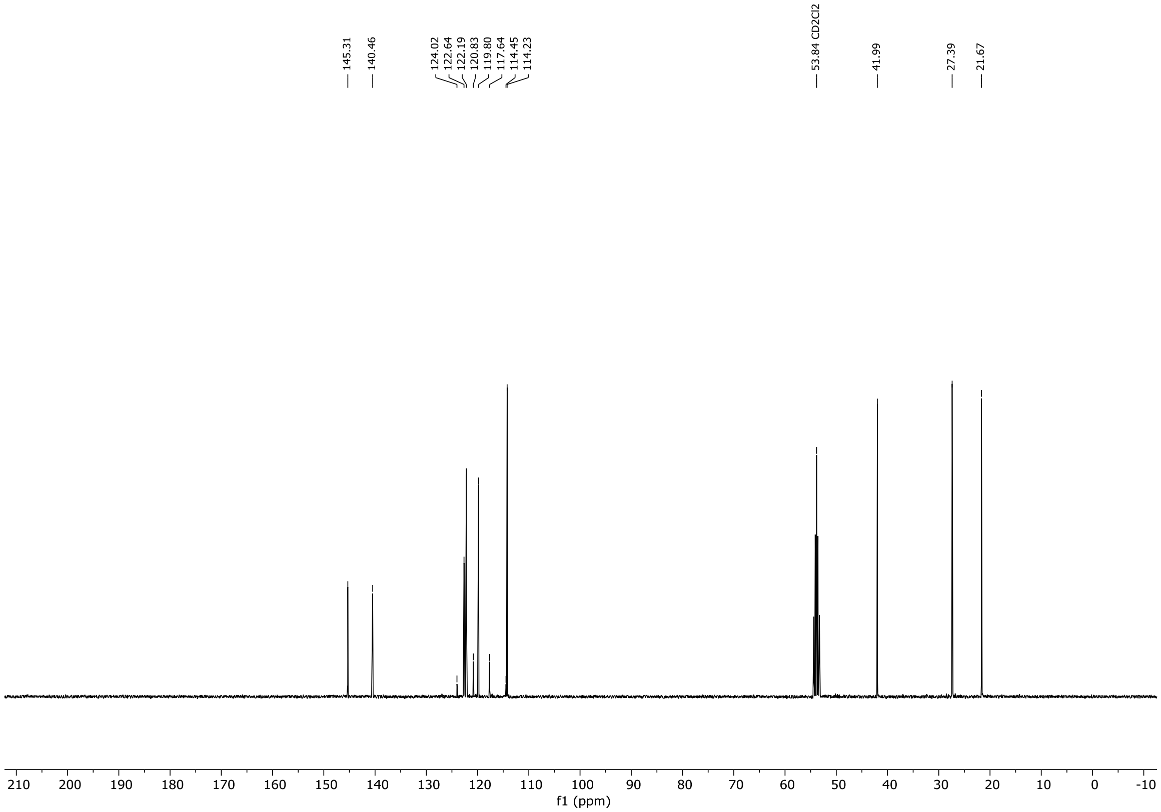

^{19}F NMR spectrum (400 MHz, CD_2Cl_2) of **2u**

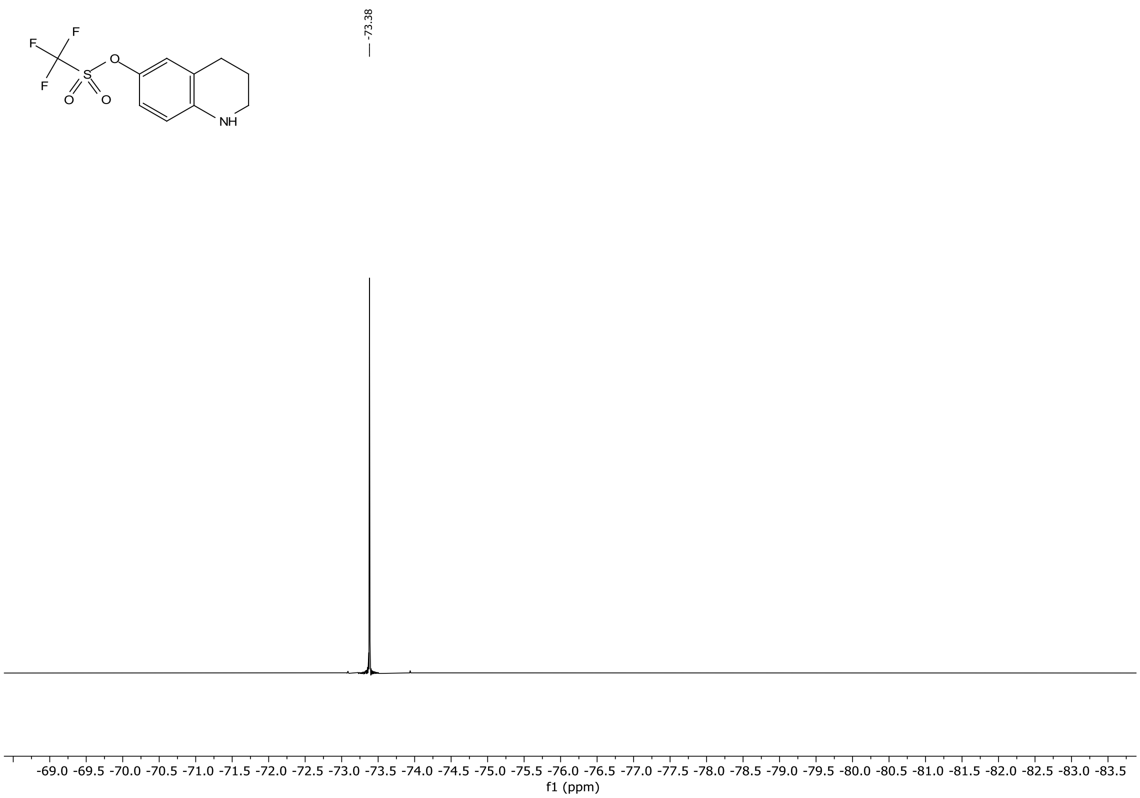

^{1}H NMR spectrum (400 MHz, $CDCl_3$) of **2v**

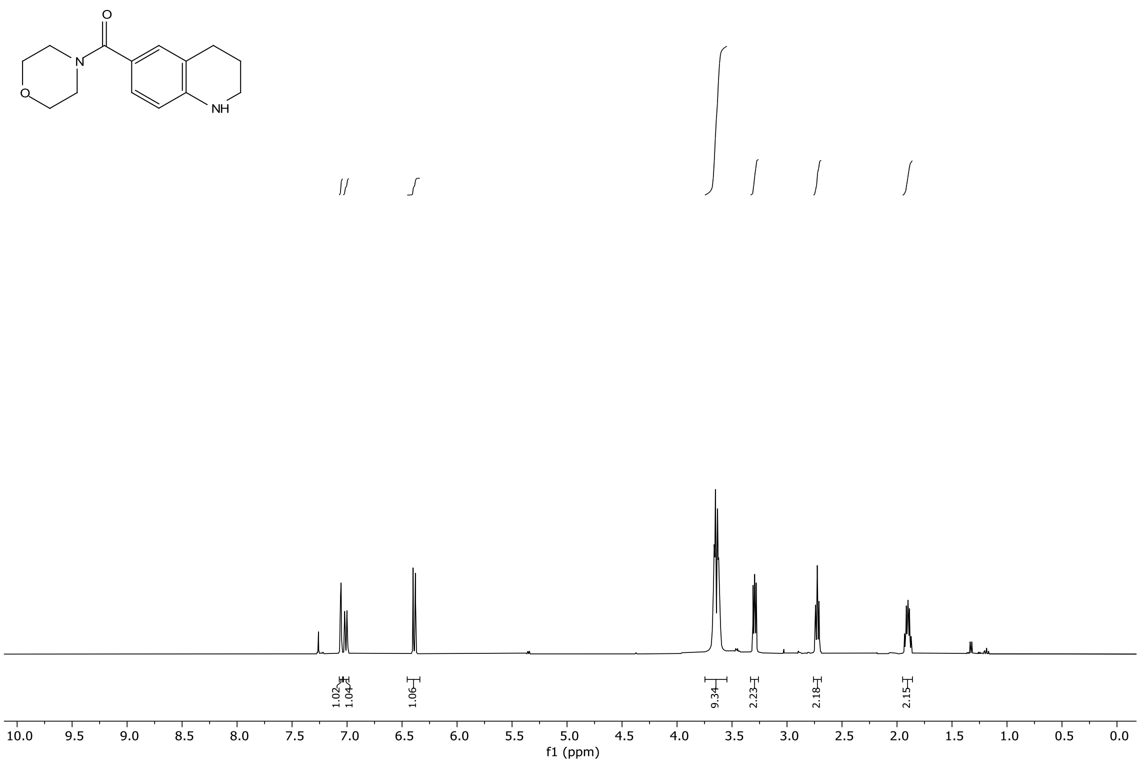

^{13}C NMR spectrum (400 MHz, $CDCl_3$) of **2v**

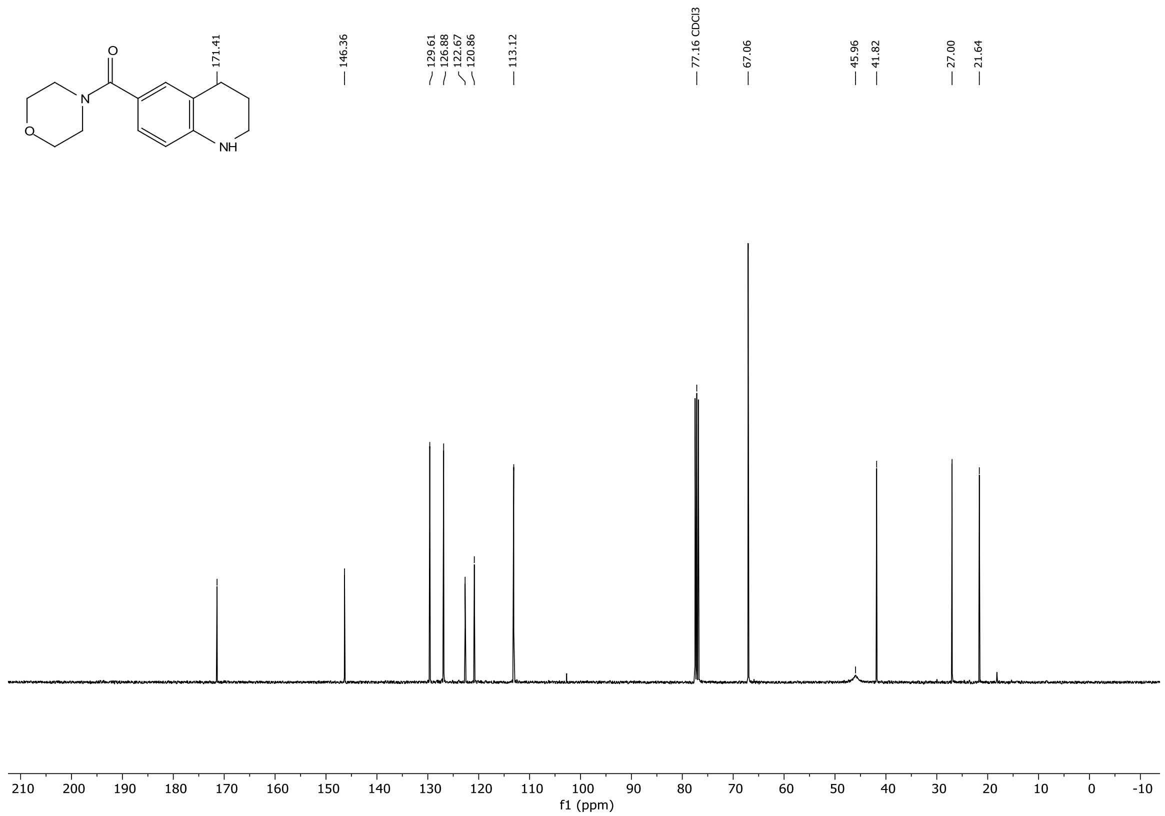

^{1}H NMR spectrum (400 MHz, CD_2Cl_2) of **2w**

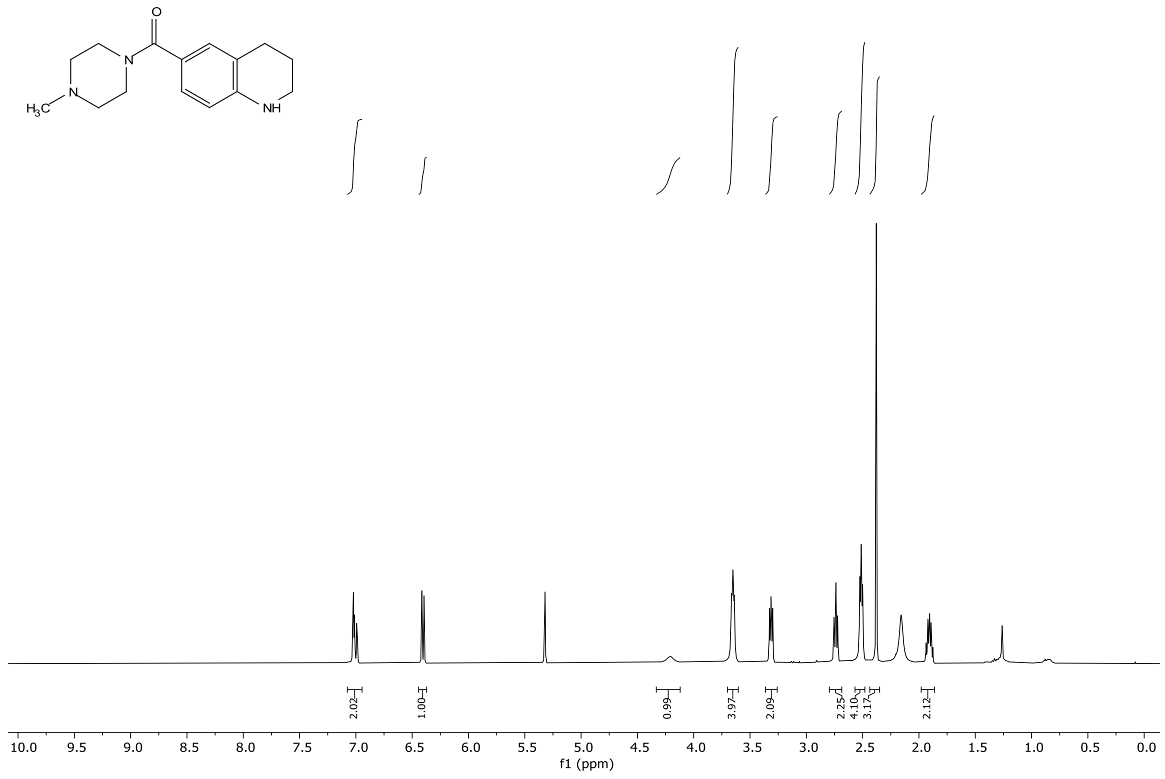

^{13}C NMR spectrum (400 MHz, CD_2Cl_2) of **2w**

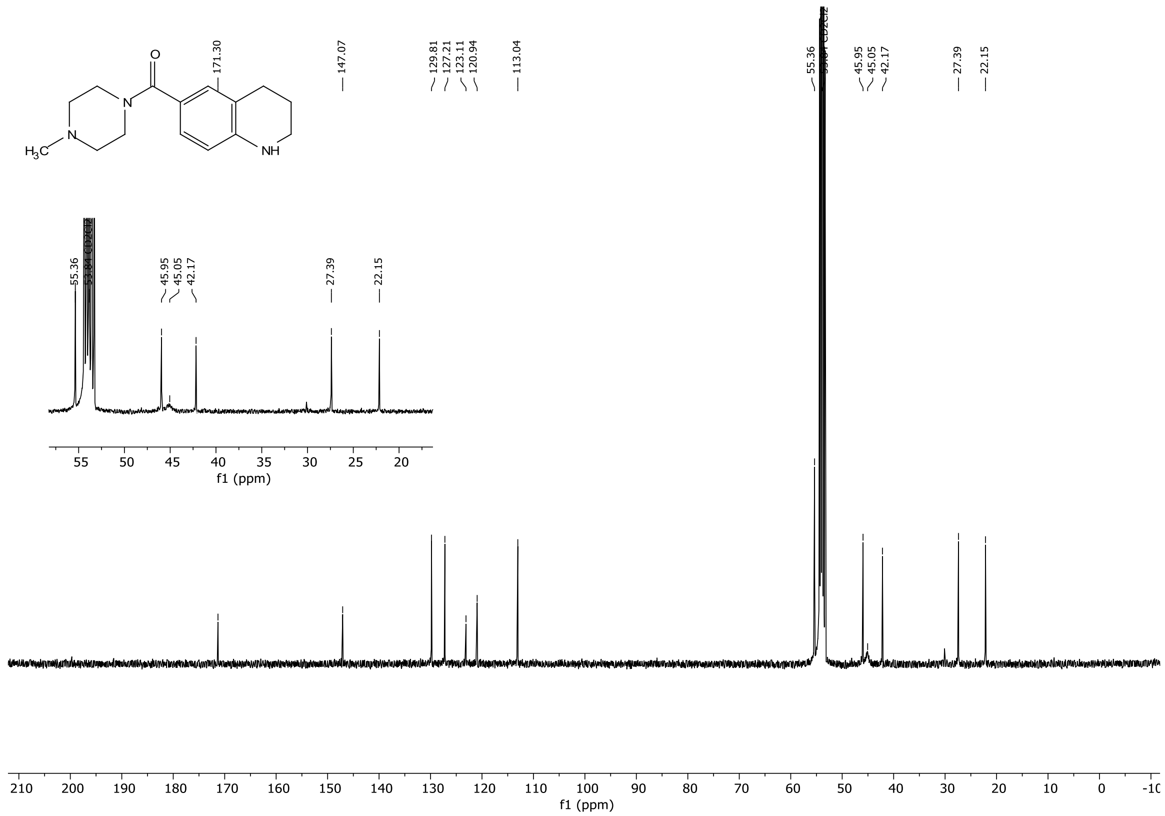

^{1}H NMR spectrum (400 MHz, MeOD) of **2x**

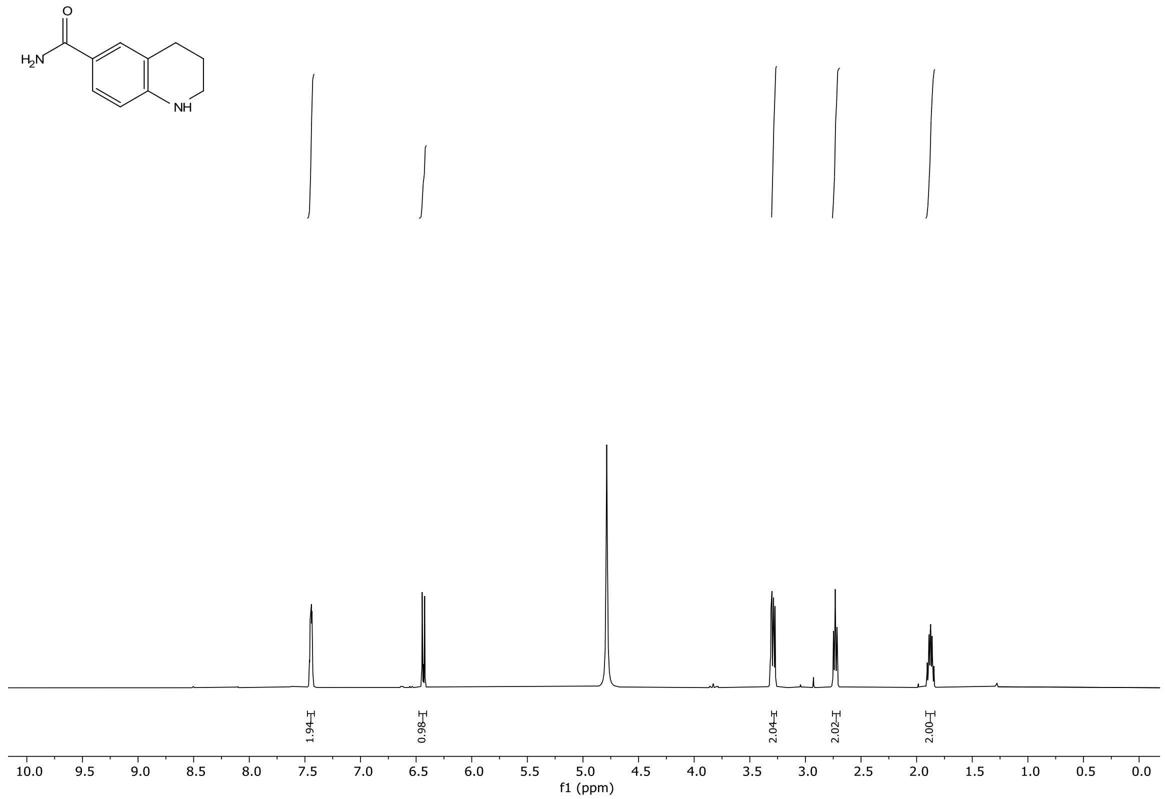

^{13}C NMR spectrum (400 MHz, MeOD) of **2x**

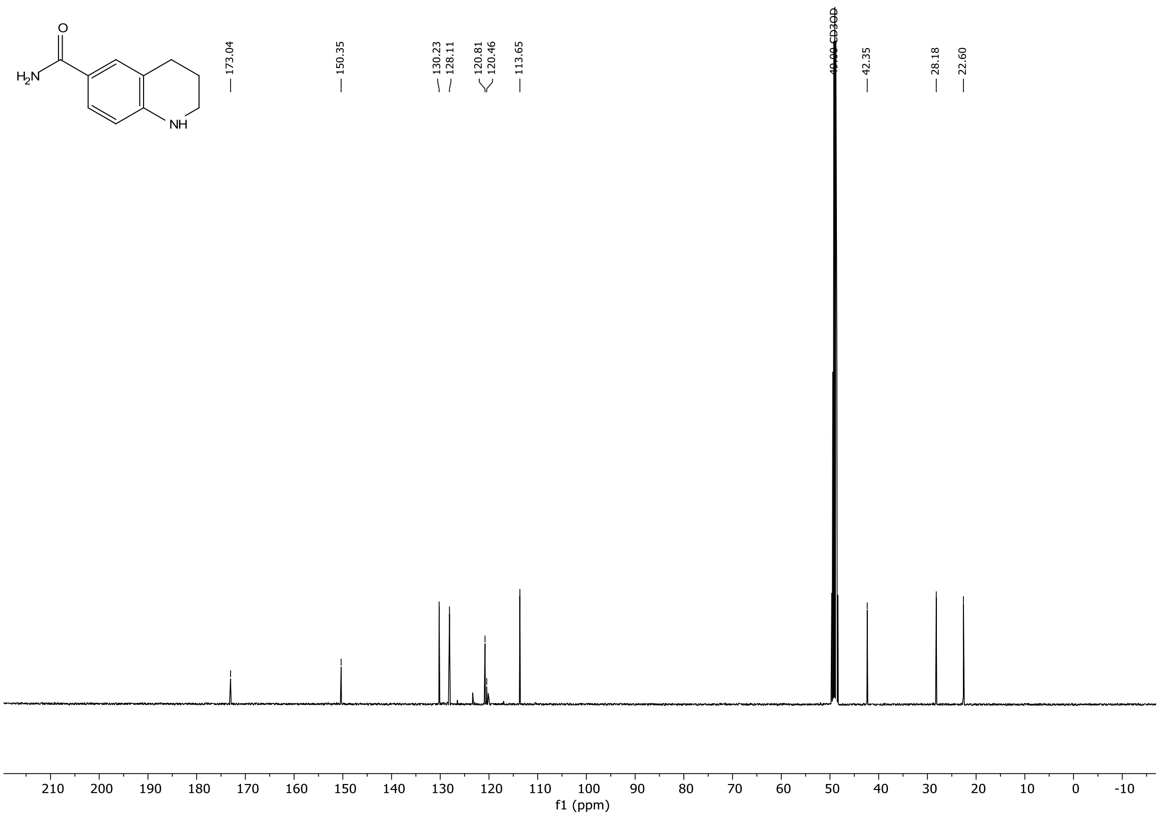

^{1}H NMR spectrum (400 MHz, CD_2Cl_2) of **2y**

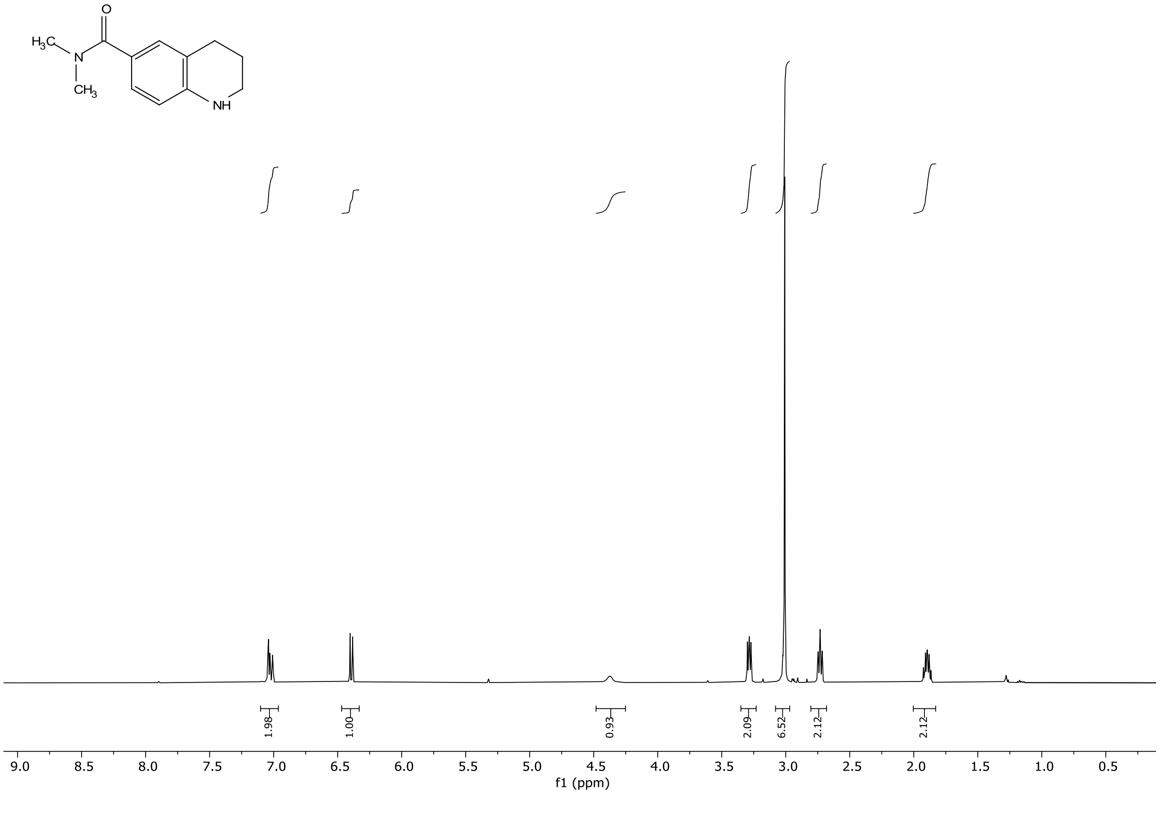

^{13}C NMR spectrum (400 MHz, CD_2Cl_2) of **2y**

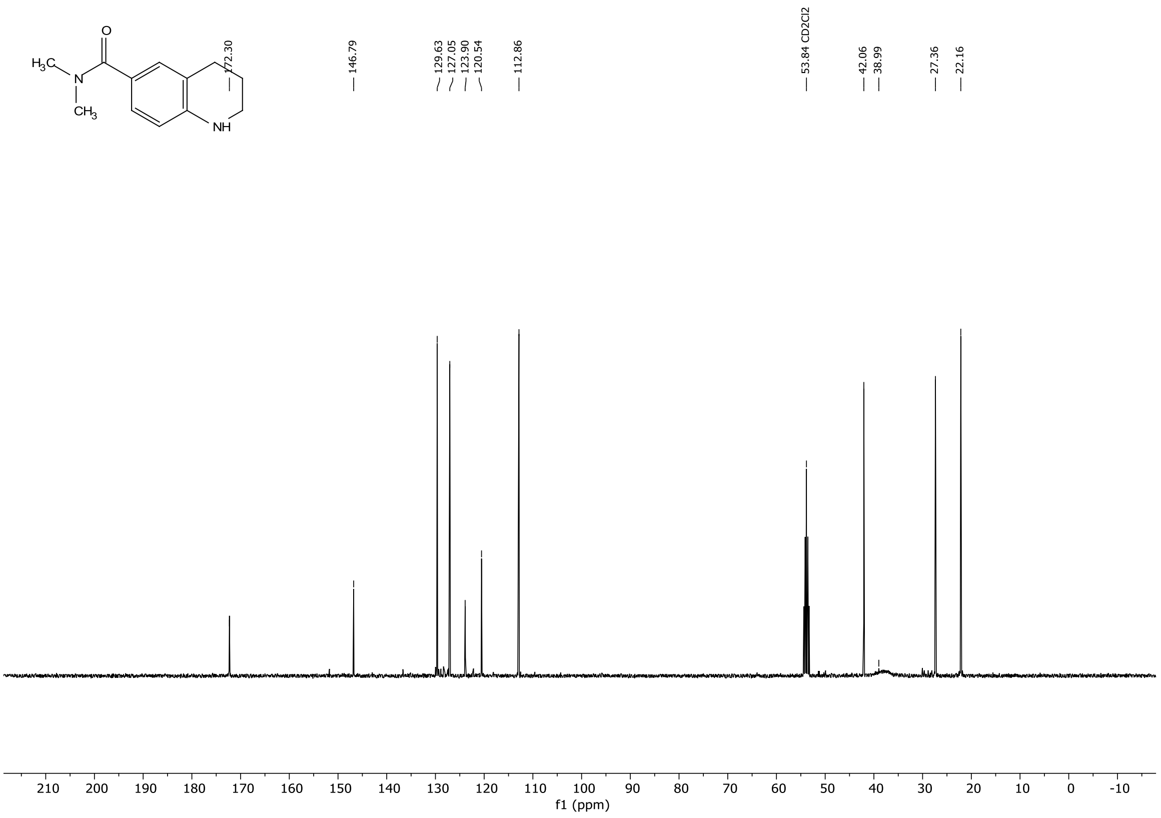

^{1}H NMR spectrum (400 MHz, $CDCl_3$) of **2z**

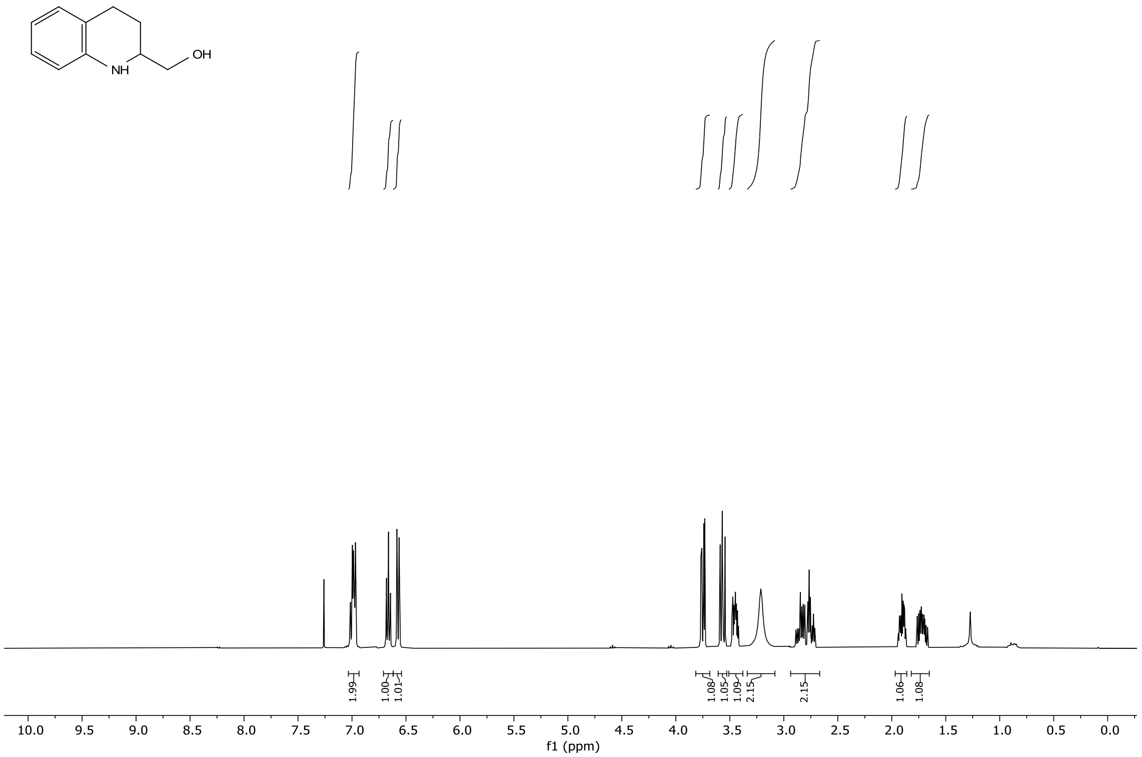

^{13}C NMR spectrum (400 MHz, $CDCl_3$) of **2z**

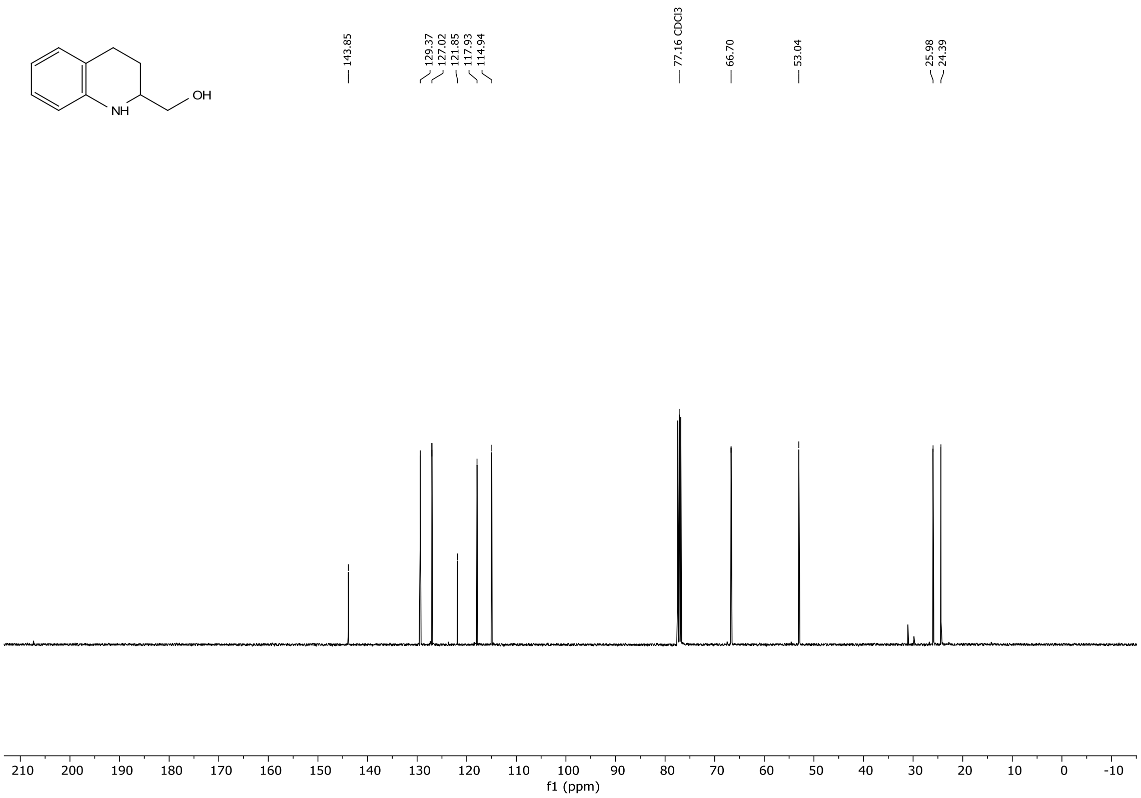

^{1}H NMR spectrum (400 MHz, CD_2Cl_2) of **2aa**

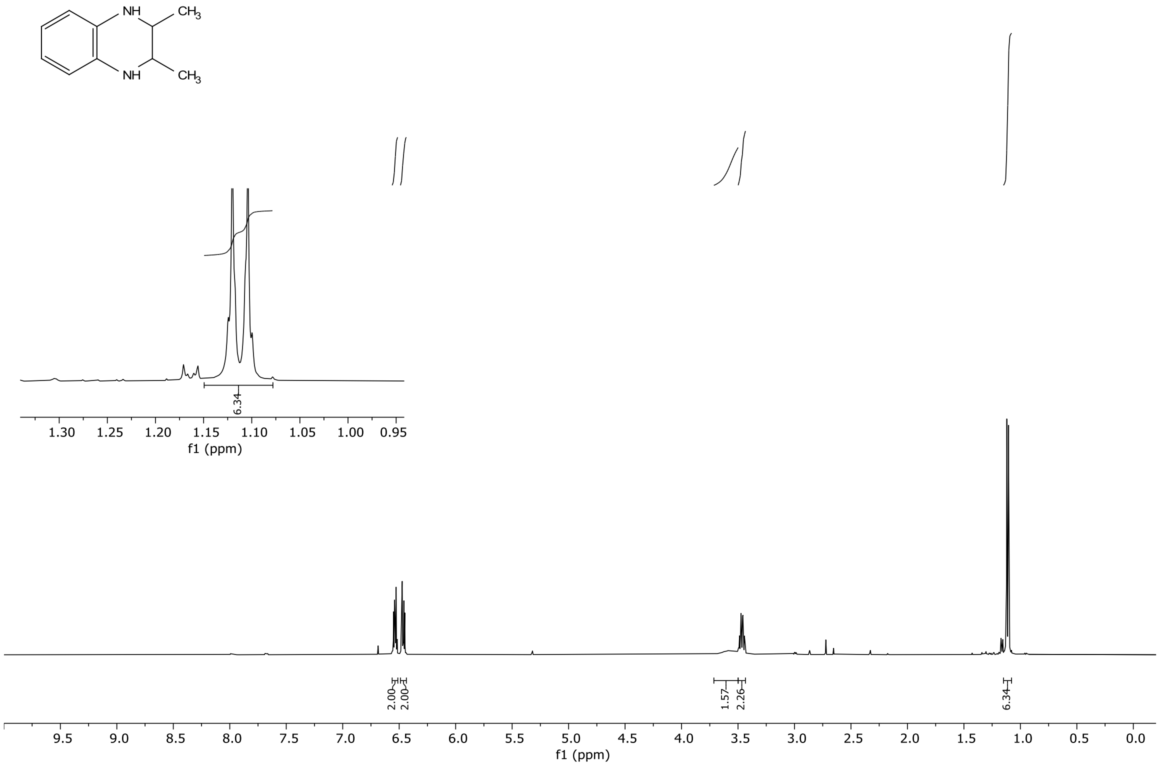

^{13}C NMR spectrum (400 MHz, CD_2Cl_2) of **2aa**

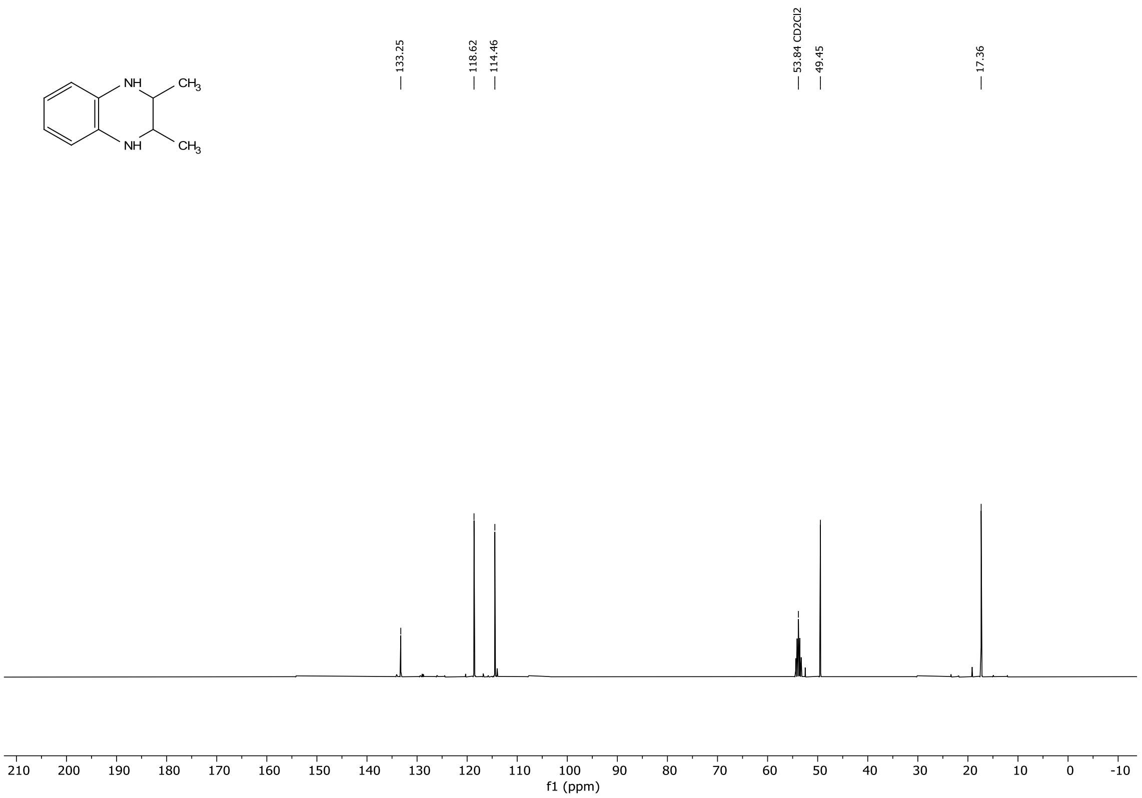

^{1}H NMR spectrum (400 MHz, CD_2Cl_2) of **2ab**

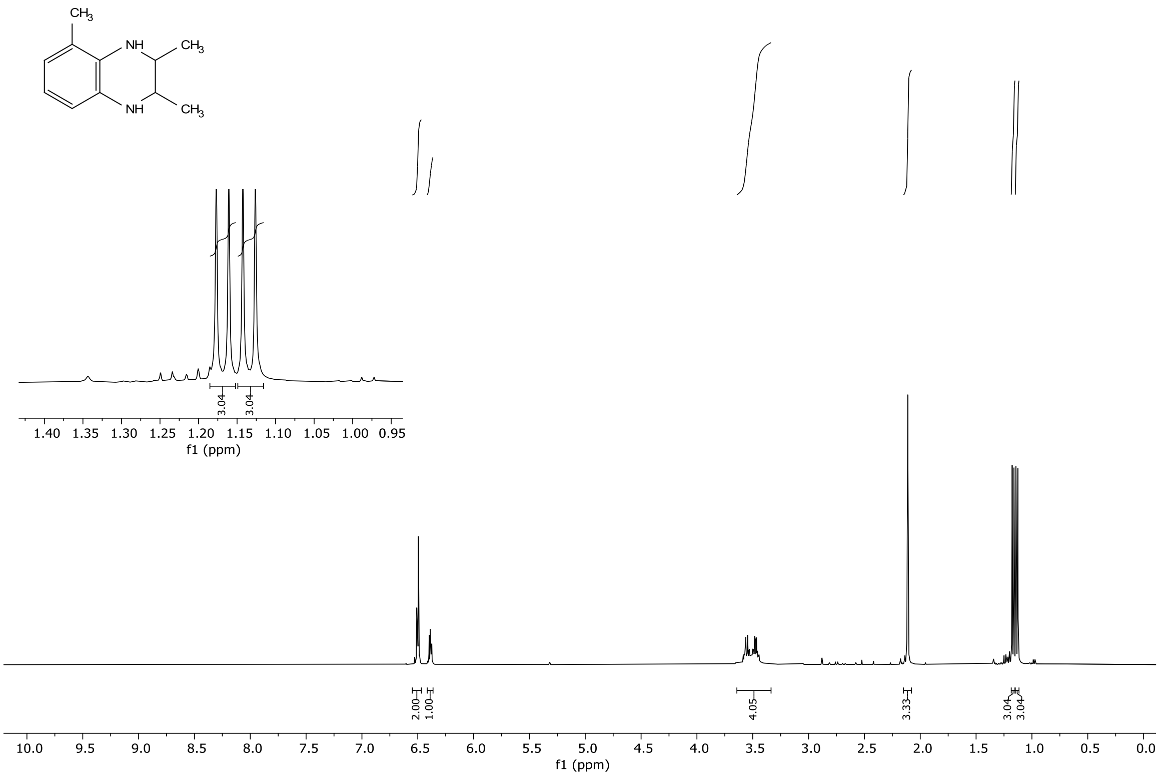

^{13}C NMR spectrum (400 MHz, CD_2Cl_2) of **2ab**

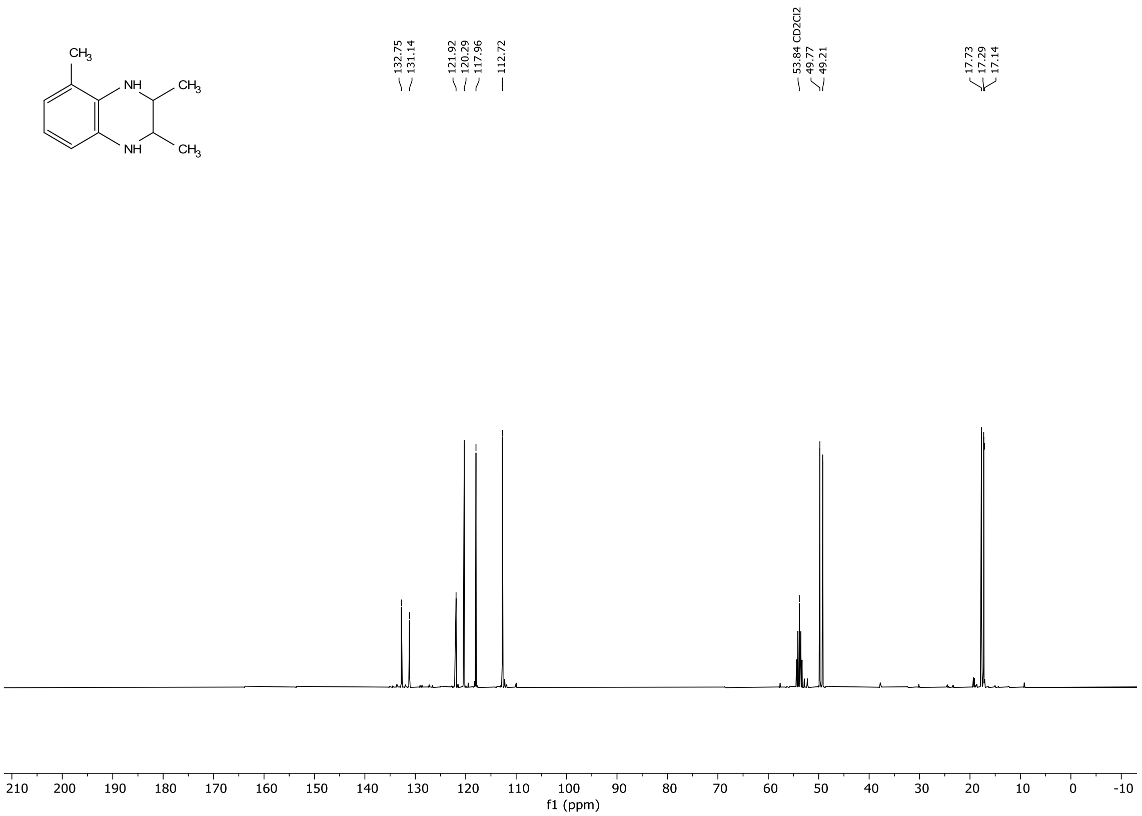

^{1}H NMR spectrum (400 MHz, CD_2Cl_2) of **2ac**

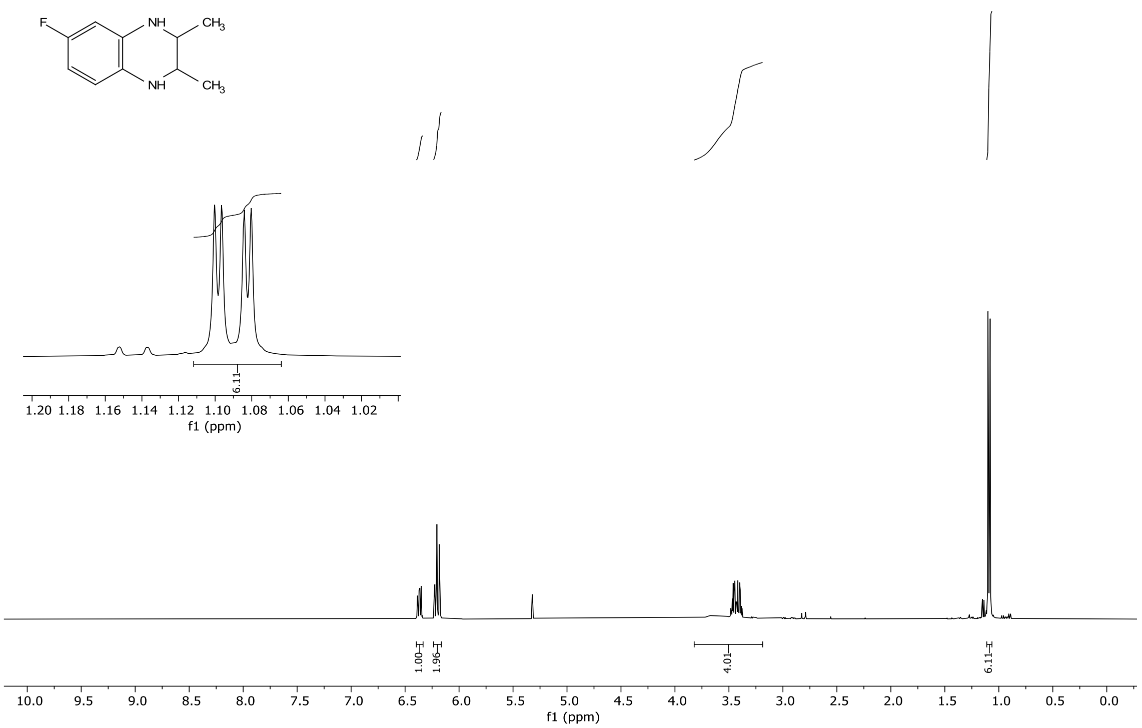

^{13}C NMR spectrum (400 MHz, CD_2Cl_2) of **2ac**

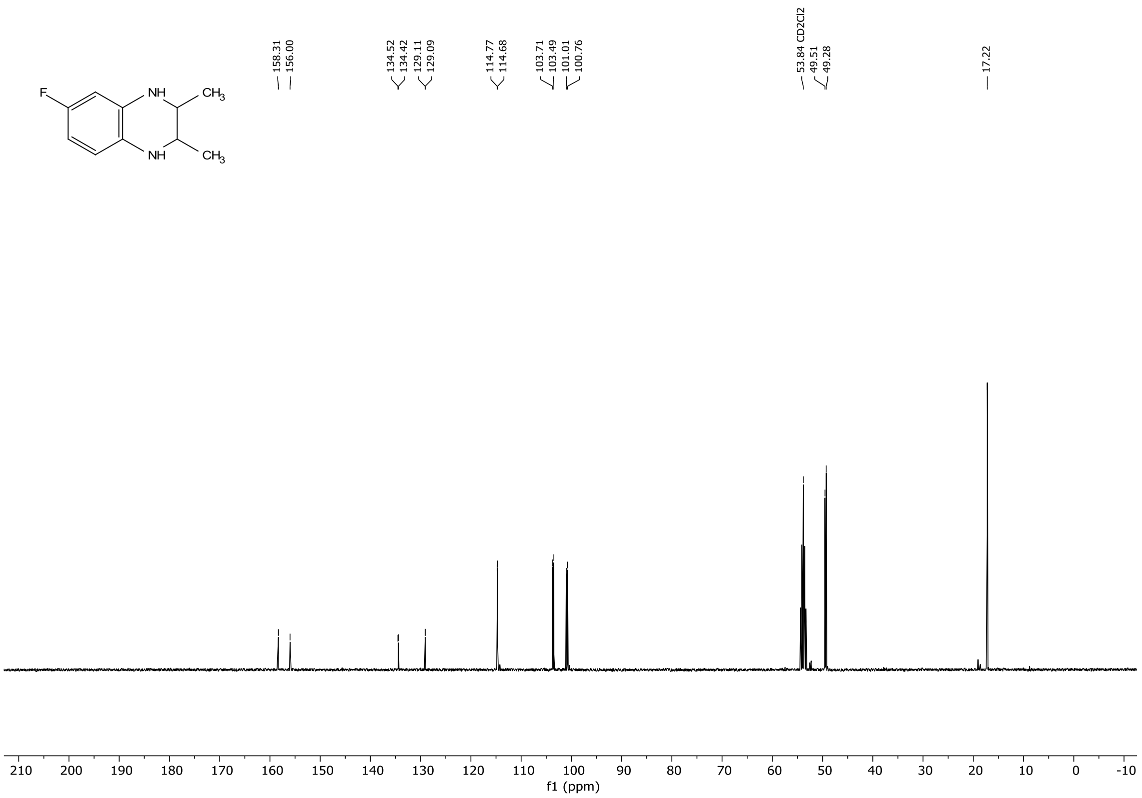

^{19}F NMR spectrum (400 MHz, CD_2Cl_2) of **2ac**

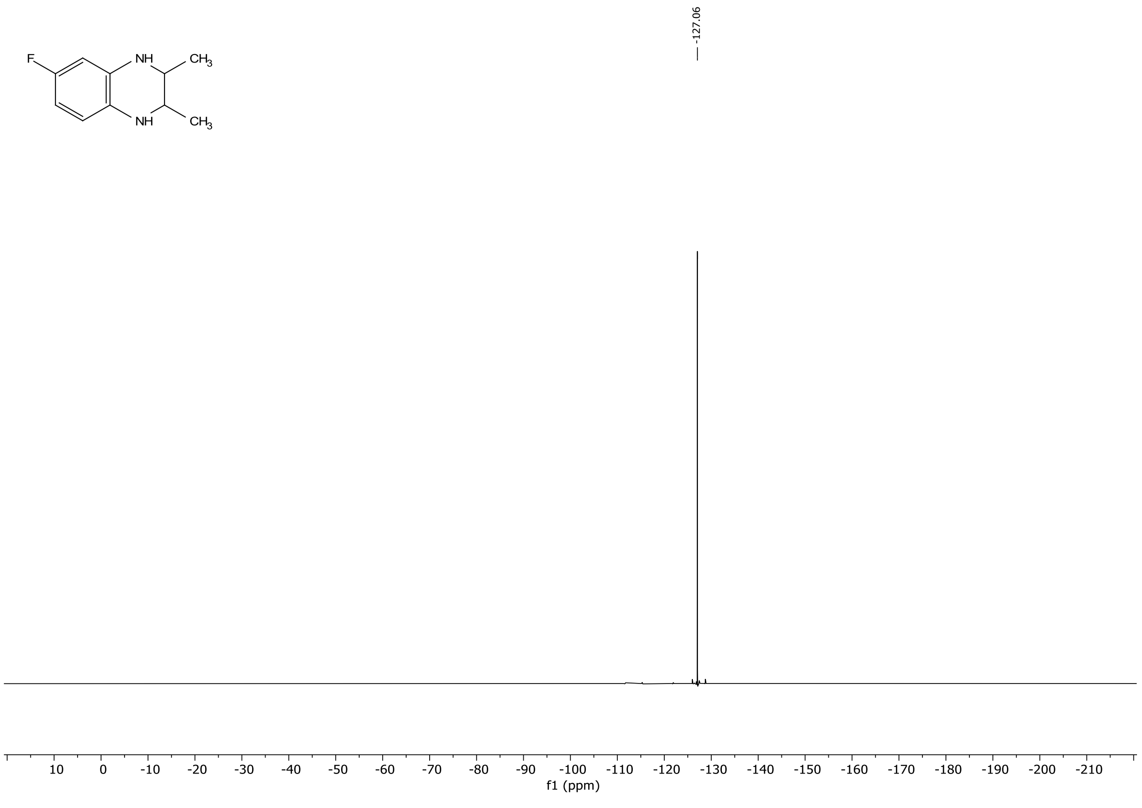

^{1}H NMR spectrum (400 MHz, CD_3CN) of **2ad**

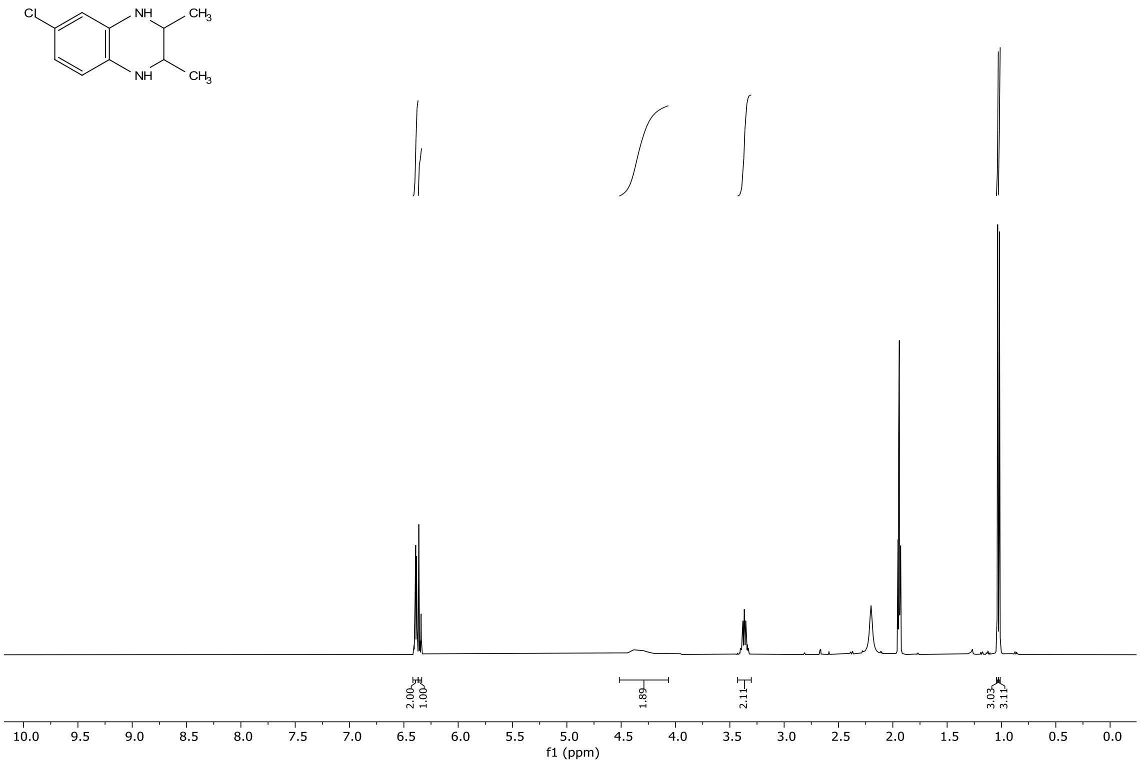

^{13}C NMR spectrum (400 MHz, CD_3CN) of **2ad**

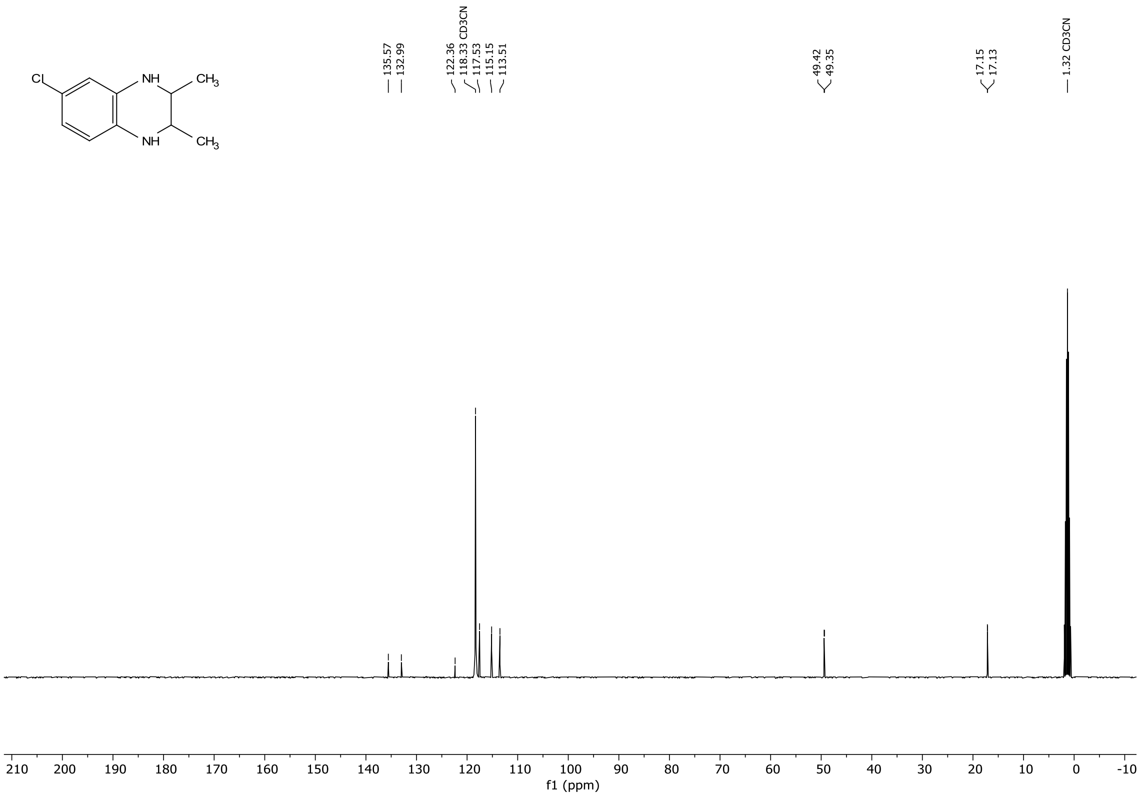

^{1}H NMR spectrum (400 MHz, CD_2Cl_2) of **2ae**

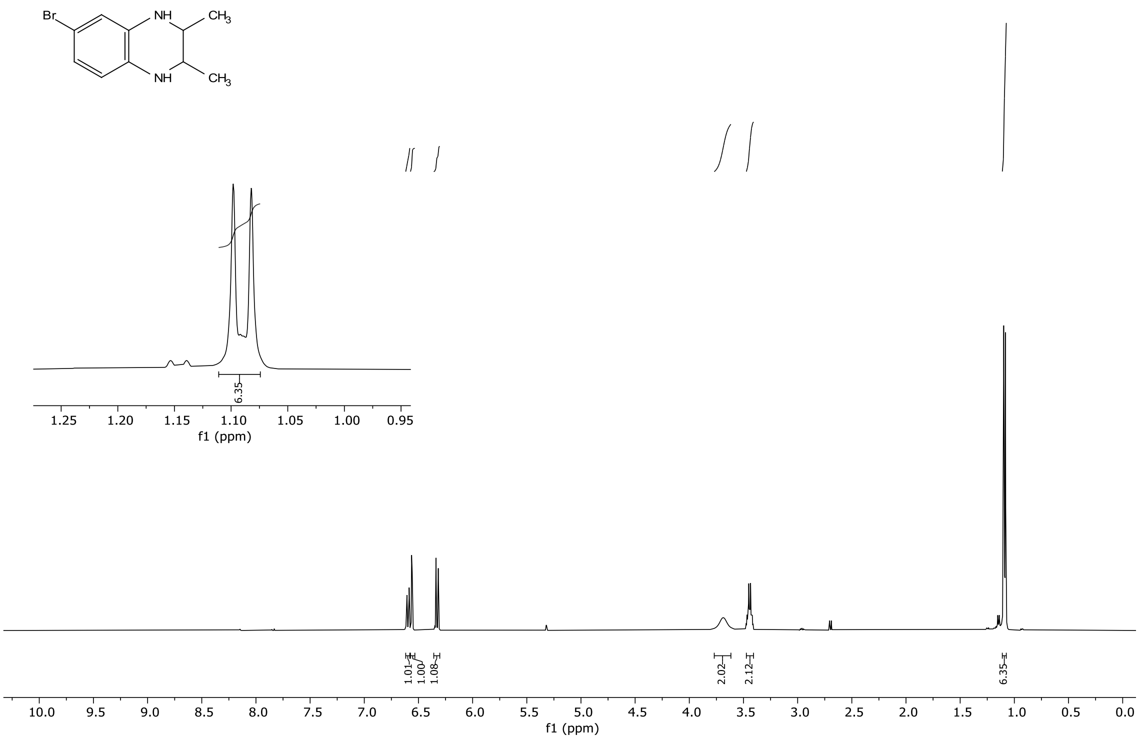

^{13}C NMR spectrum (400 MHz, CD_2Cl_2) of **2ae**

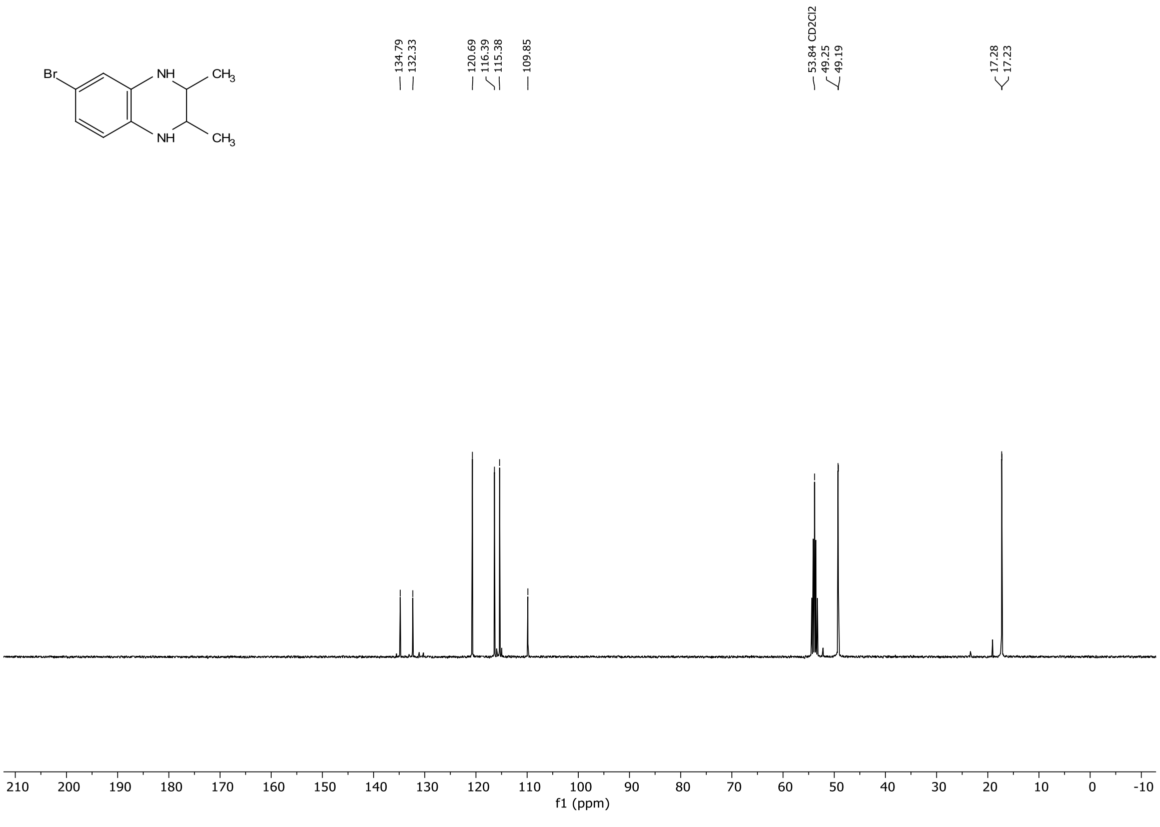

^{1}H NMR spectrum (400 MHz, CD_2Cl_2) of **2ag**

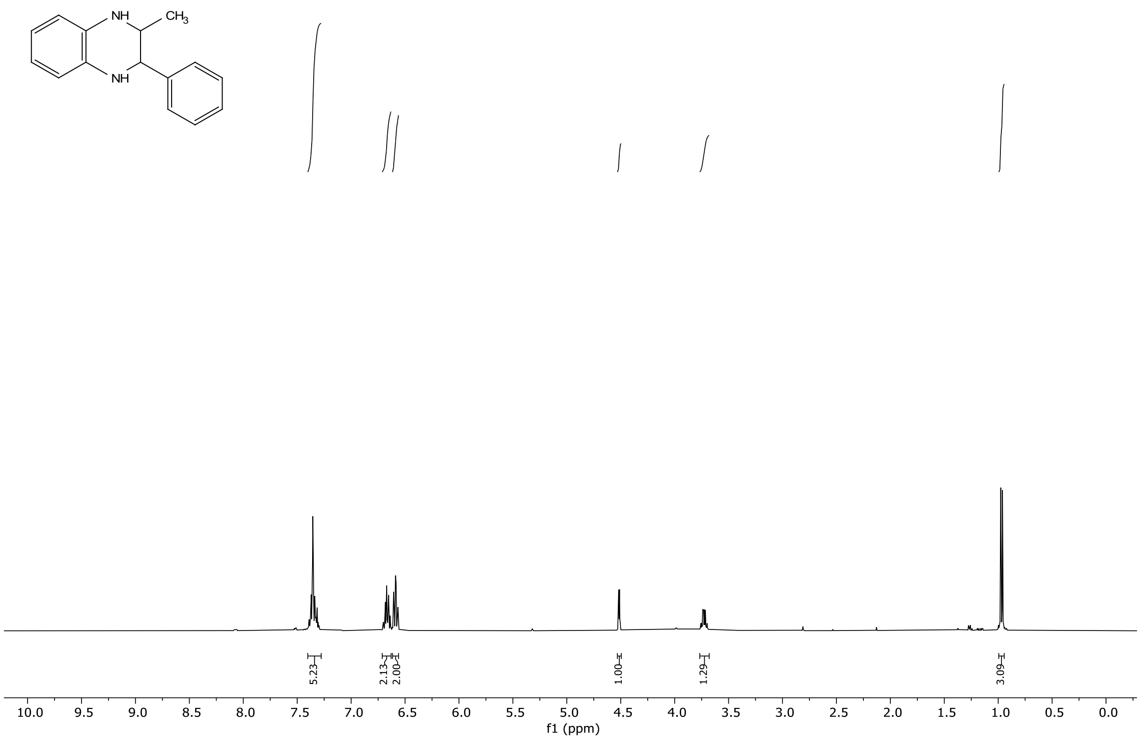

^{13}C NMR spectrum (400 MHz, CD_2Cl_2) of **2ag**

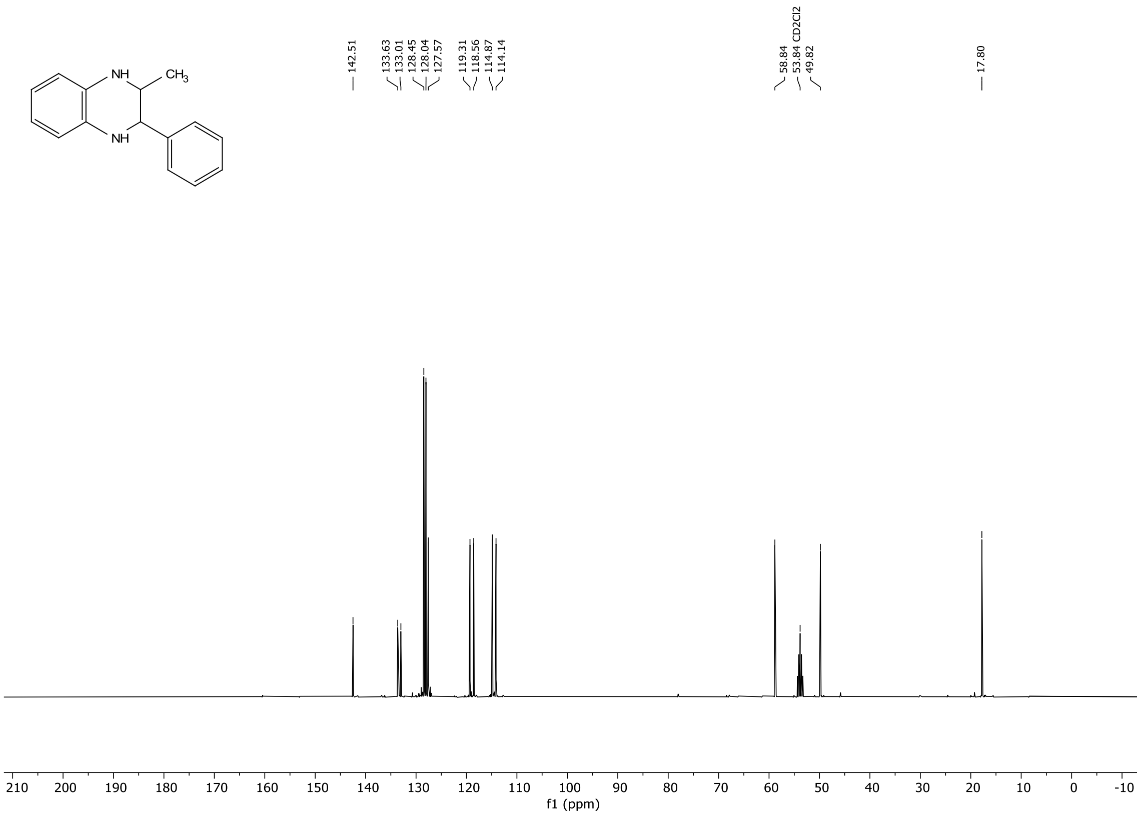

^{1}H NMR spectrum (400 MHz, DMSO) of **2ak**

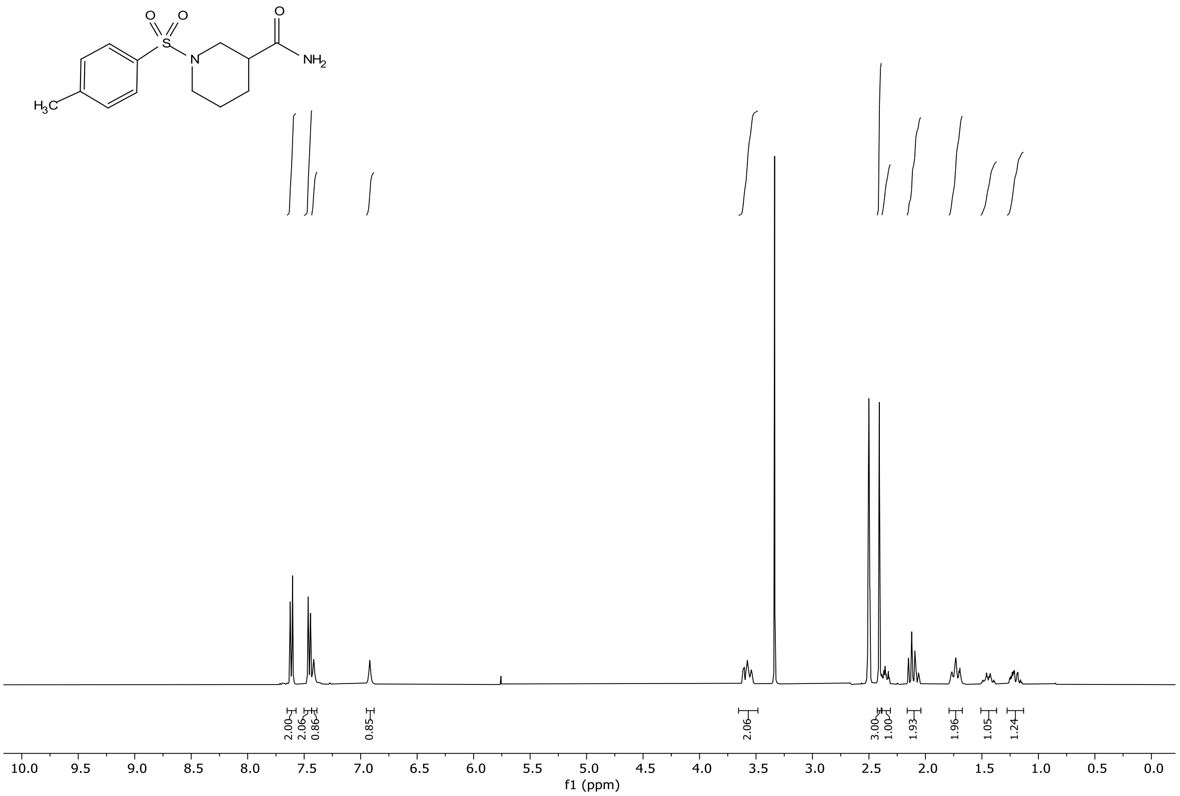

^{13}C NMR spectrum (400 MHz, DMSO) of **2ak**

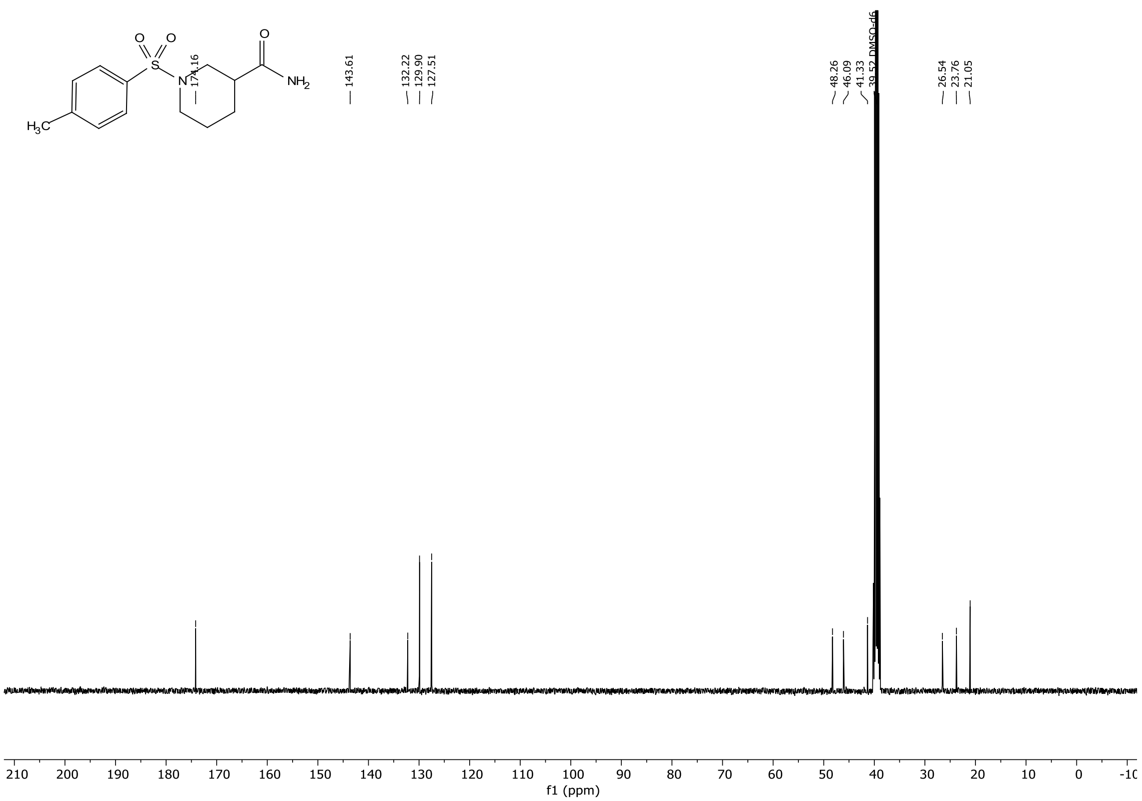

^{1}H NMR spectrum (400 MHz, CD_2Cl_2) of **2am**

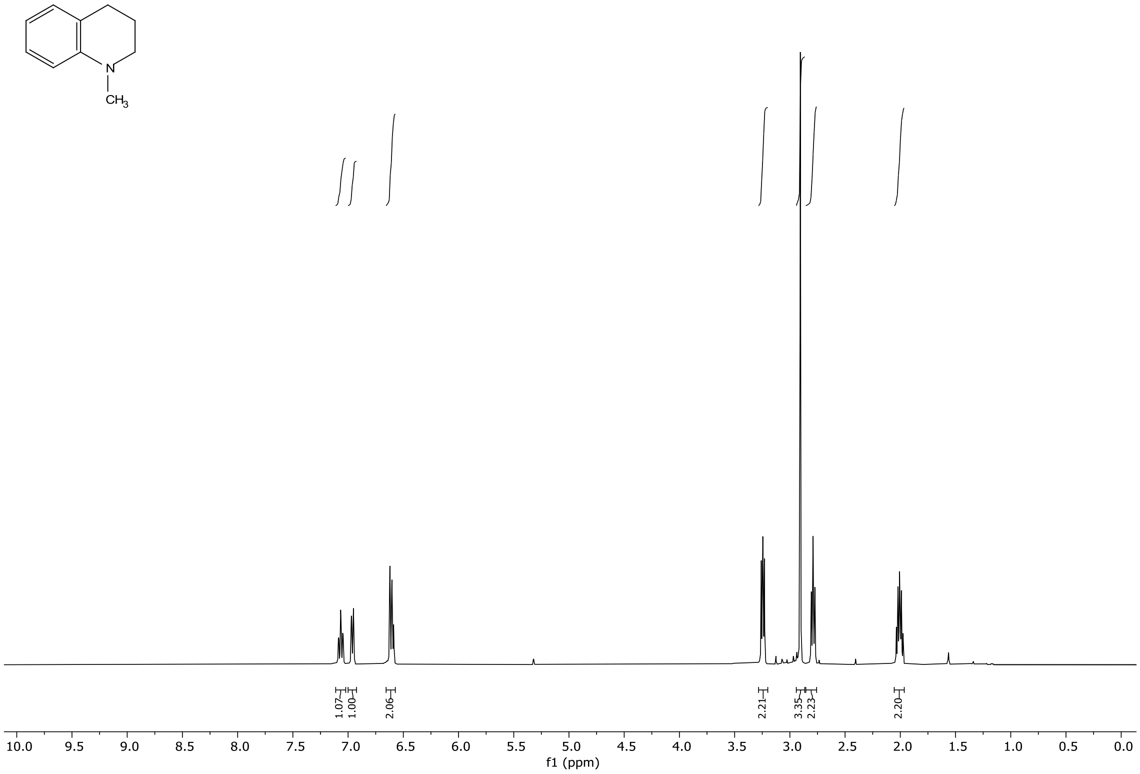

^{13}C NMR spectrum (400 MHz, CD_2Cl_2) of **2am**

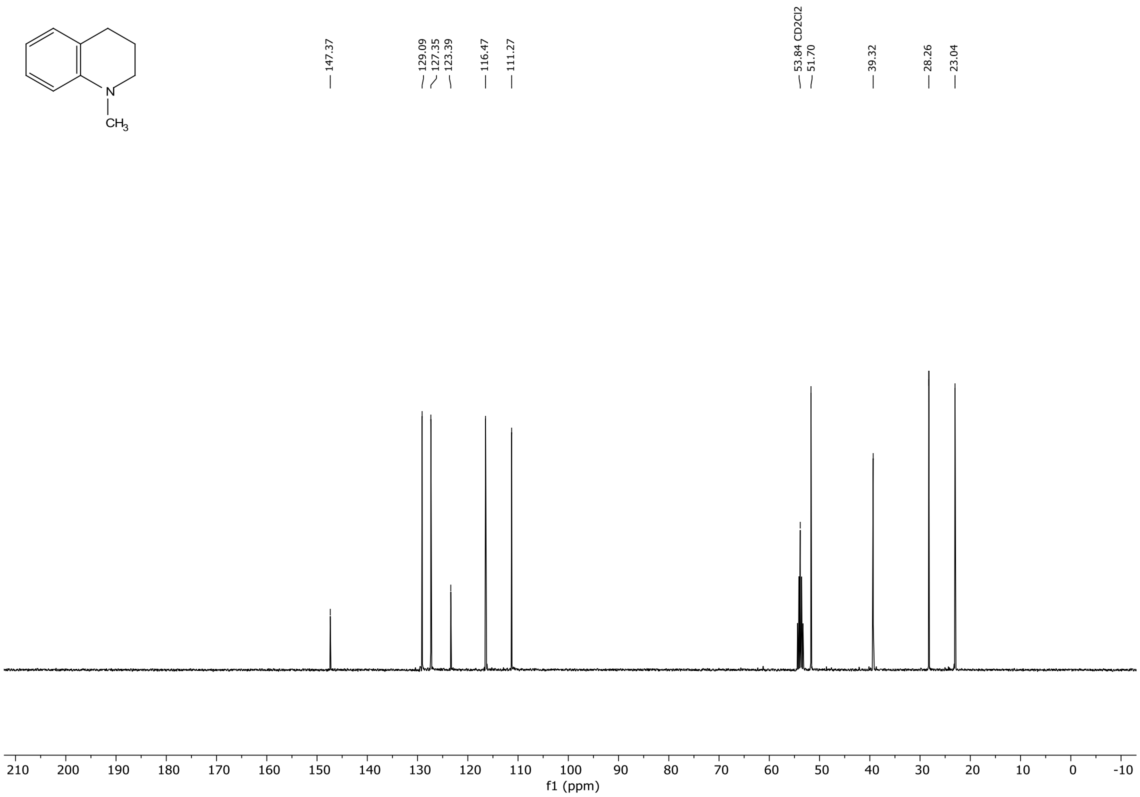

^{1}H NMR spectrum (400 MHz, CD_2Cl_2) of **2an**

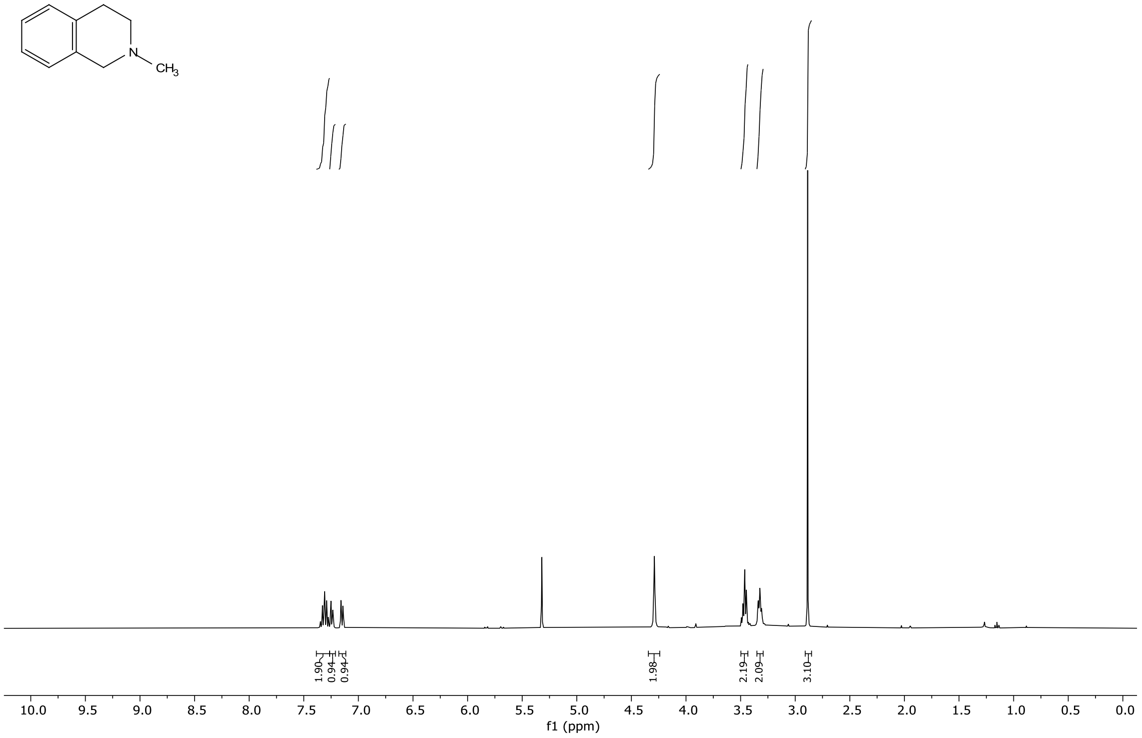

^{13}C NMR spectrum (400 MHz, CD_2Cl_2) of **2an**

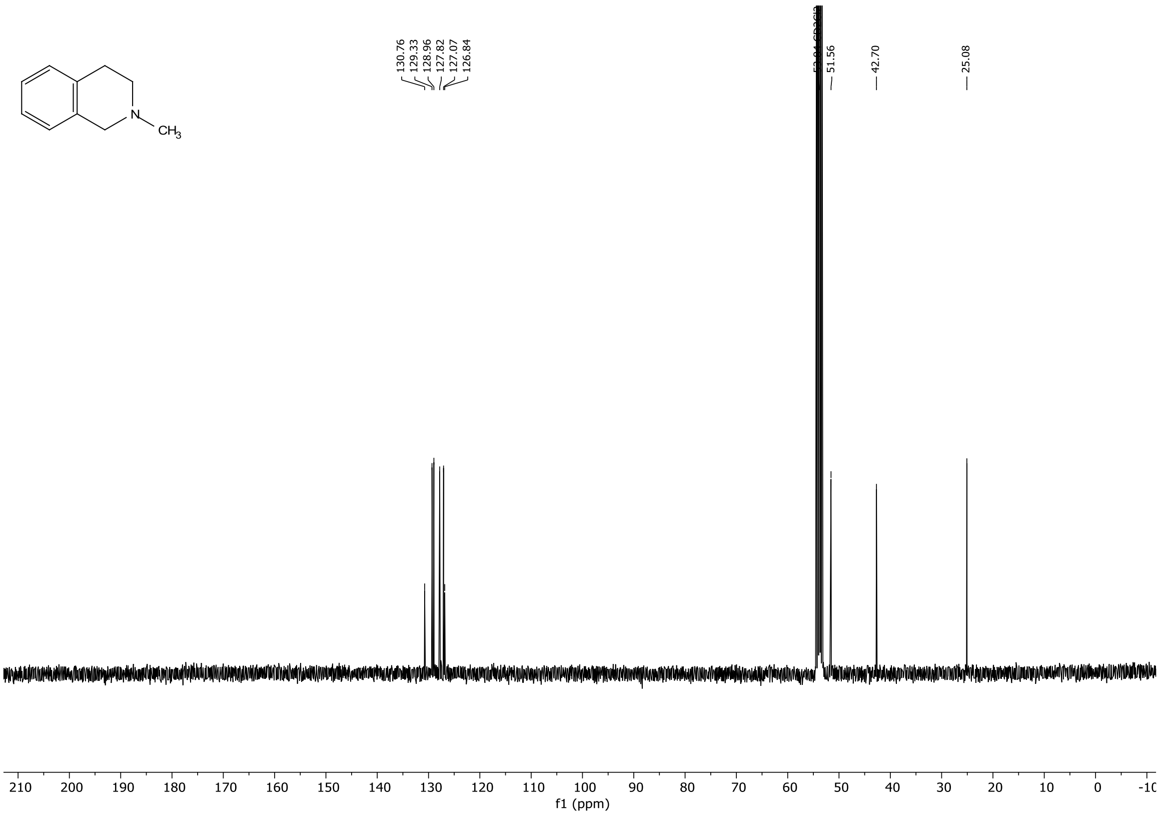

^{1}H NMR spectrum (400 MHz, CD_2Cl_2) of **3a**

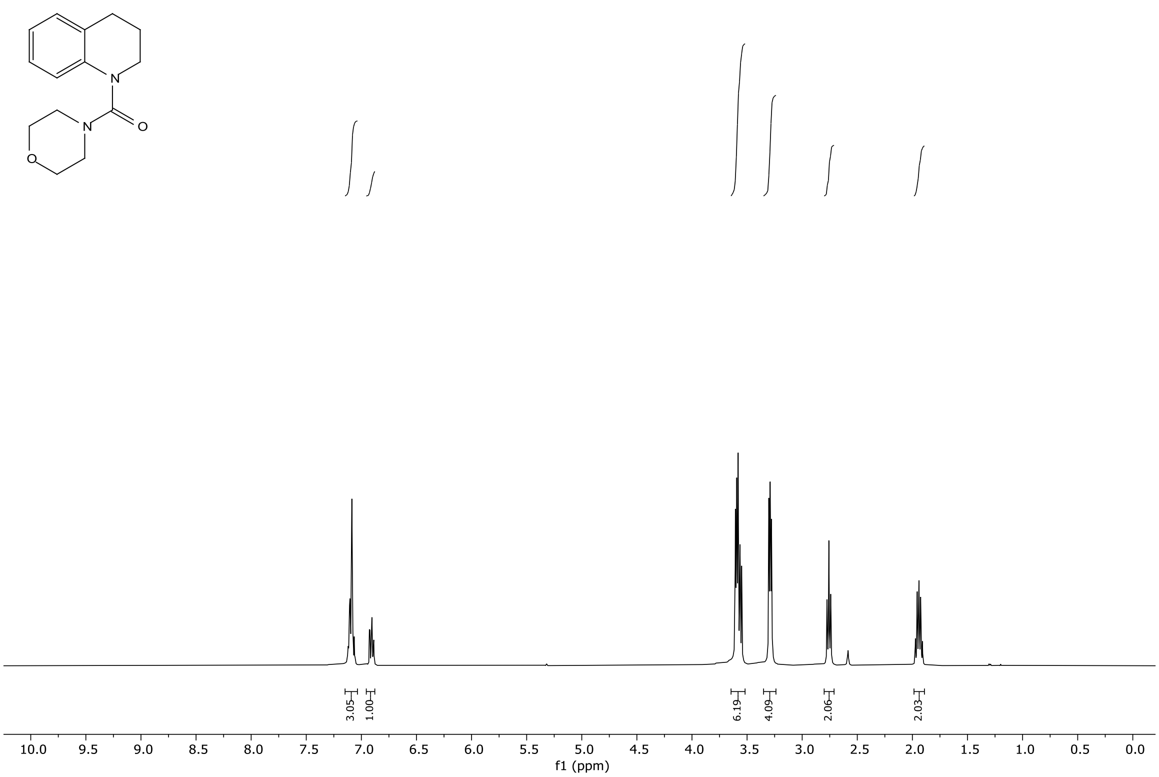

^{13}C NMR spectrum (400 MHz, CD_2Cl_2) of **3a**

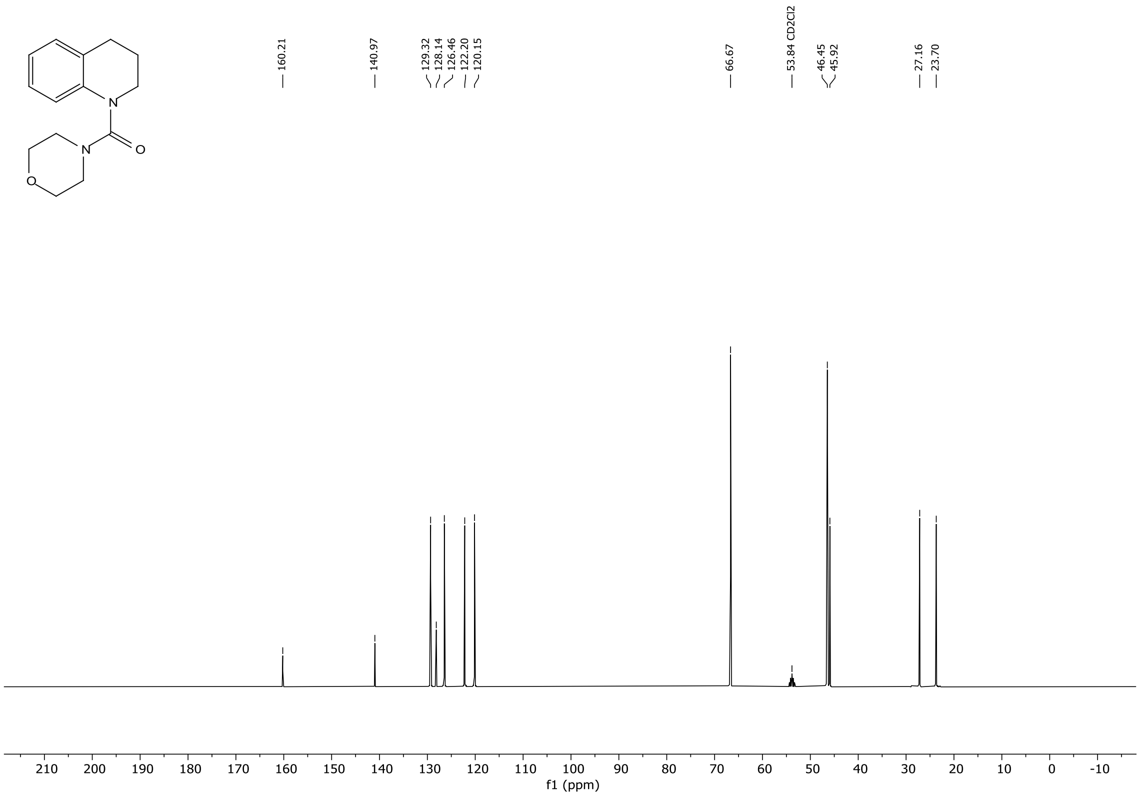

^{1}H NMR spectrum (400 MHz, CD_2Cl_2) of **3b**

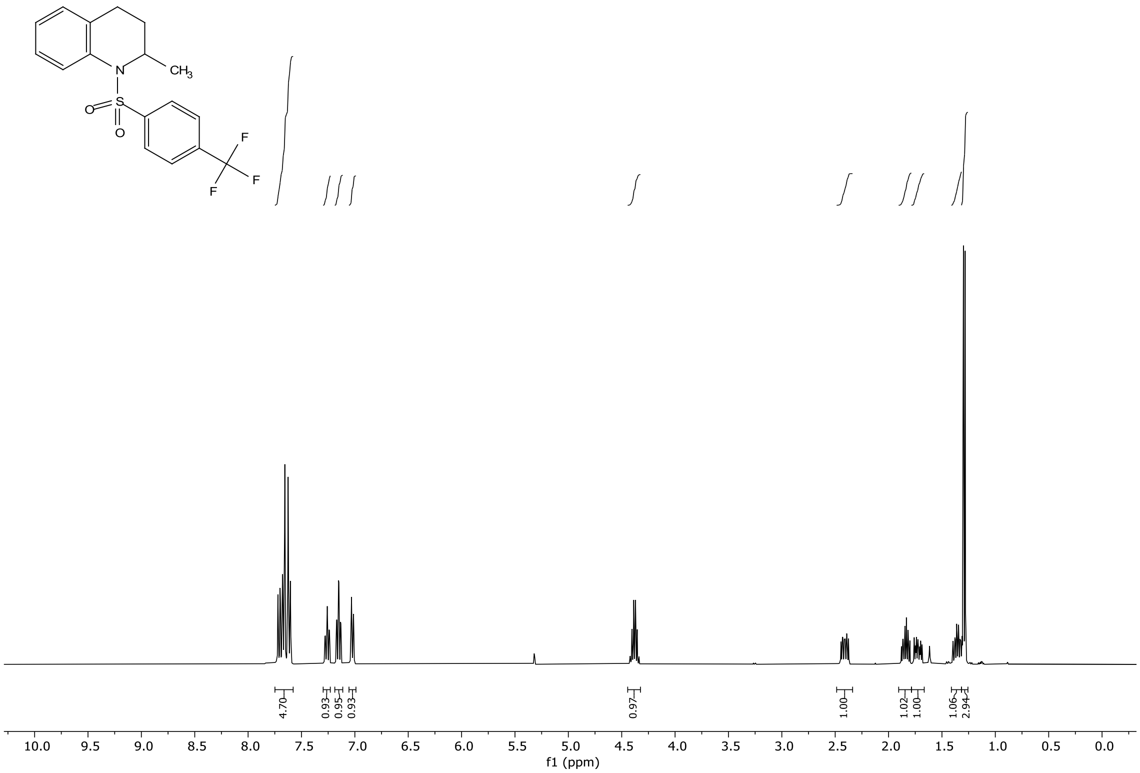

^{13}C NMR spectrum (400 MHz, CD_2Cl_2) of **3b**

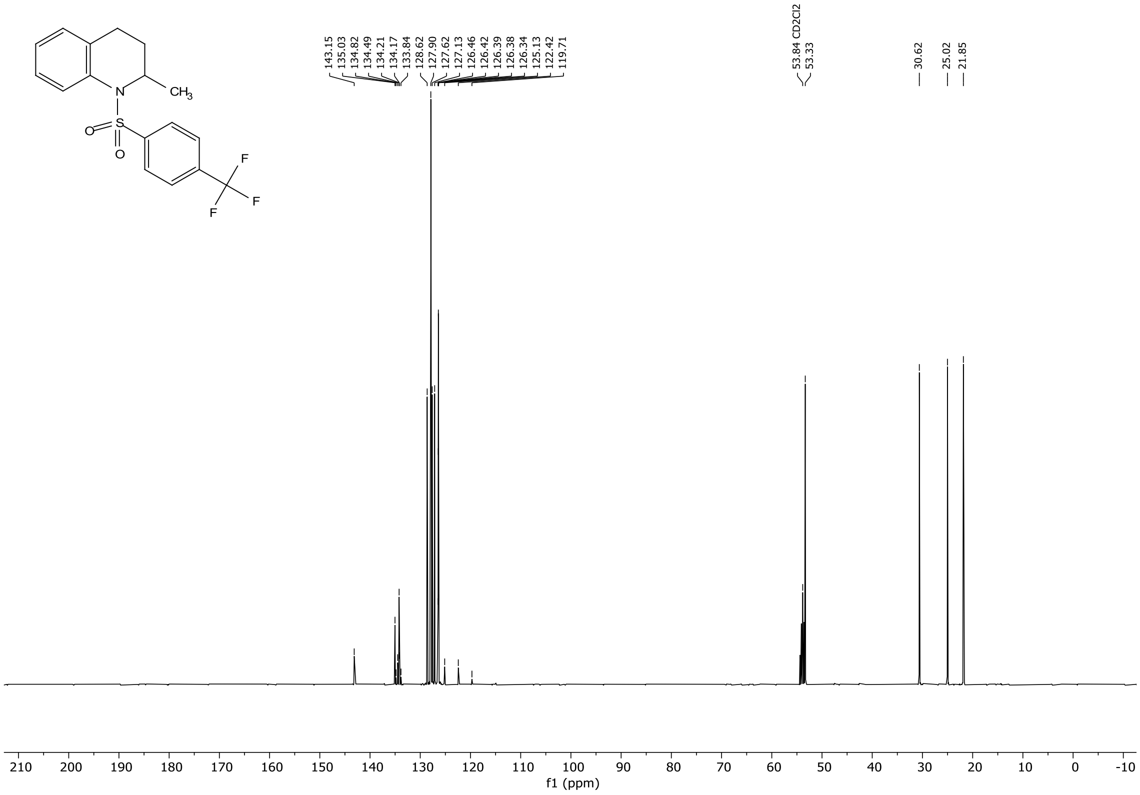

^{19}F NMR spectrum (400 MHz, CD_2Cl_2) of **3b**

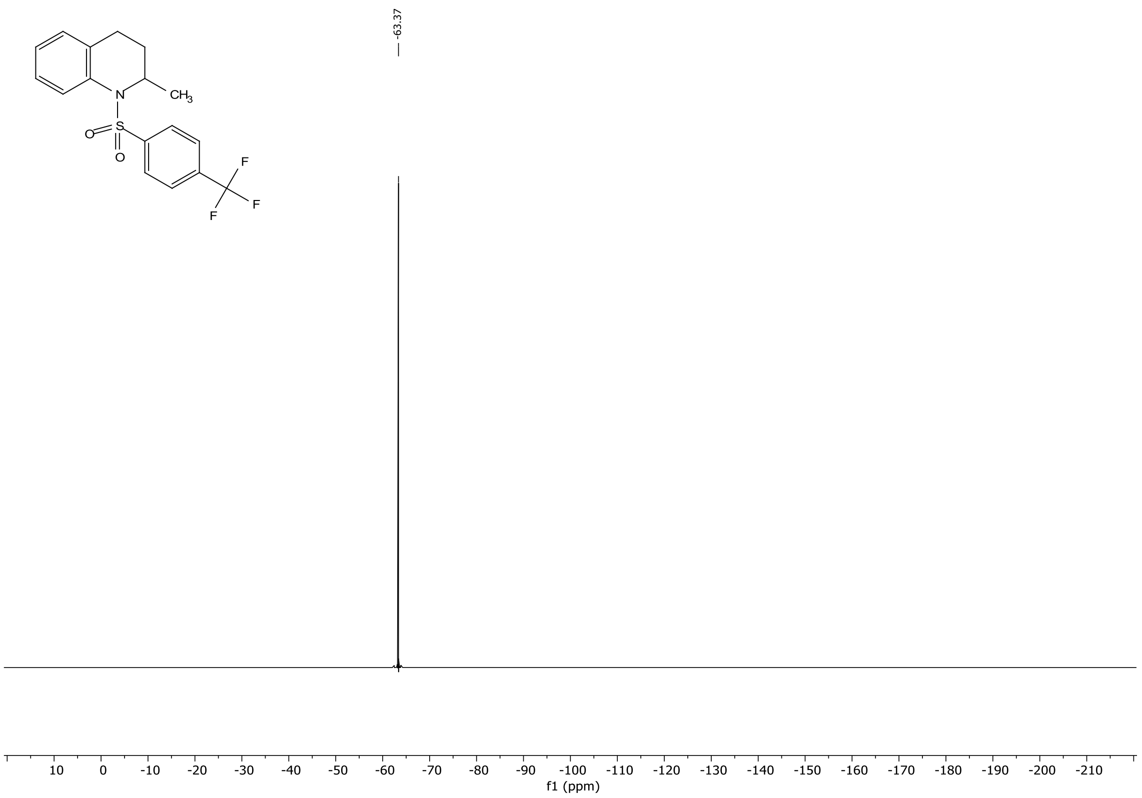

^{1}H NMR spectrum (400 MHz, CD_2Cl_2) of **3c**

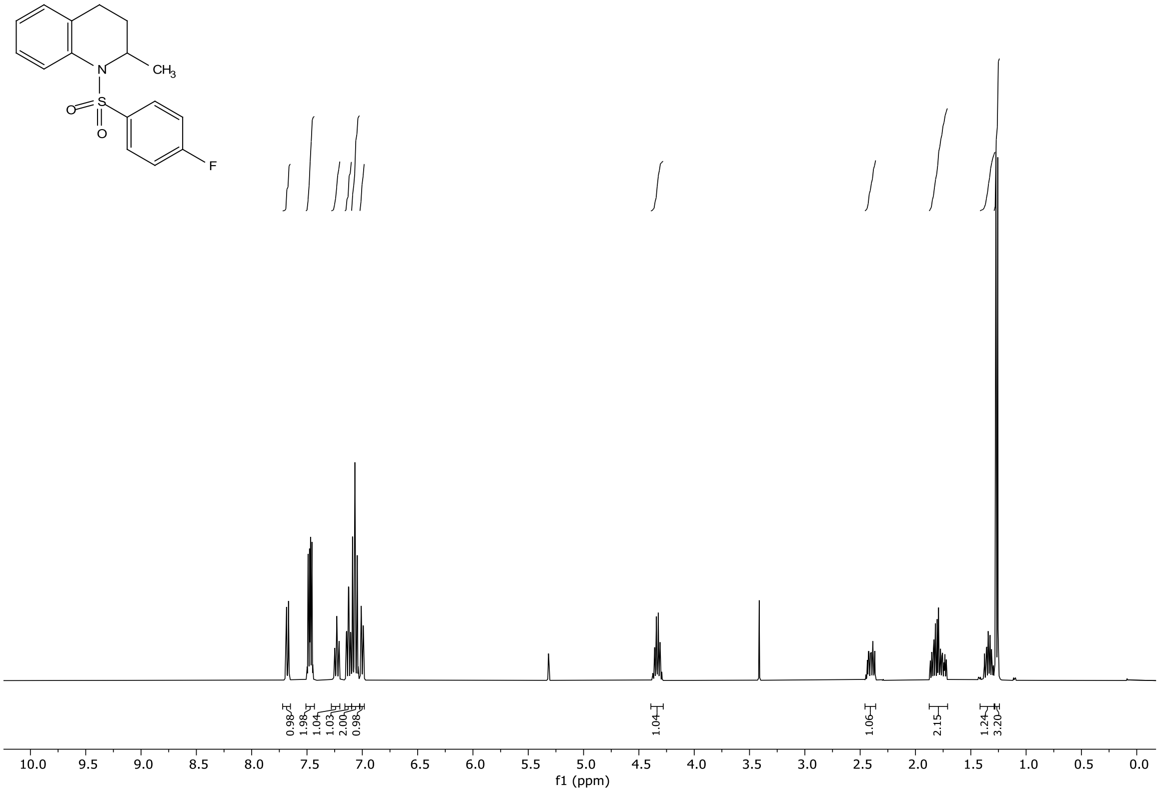

^{13}C NMR spectrum (400 MHz, CD_2Cl_2) of **3c**

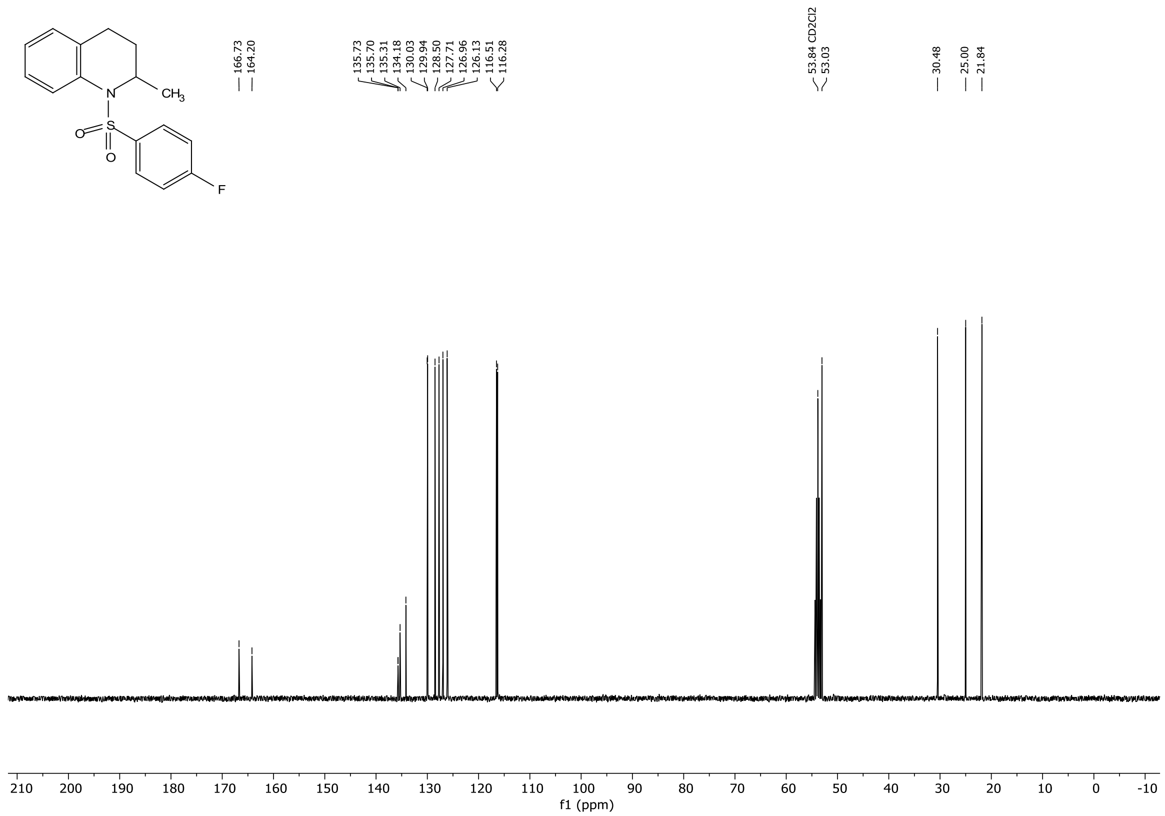

^{19}F NMR spectrum (400 MHz, CD_2Cl_2) of **3c**

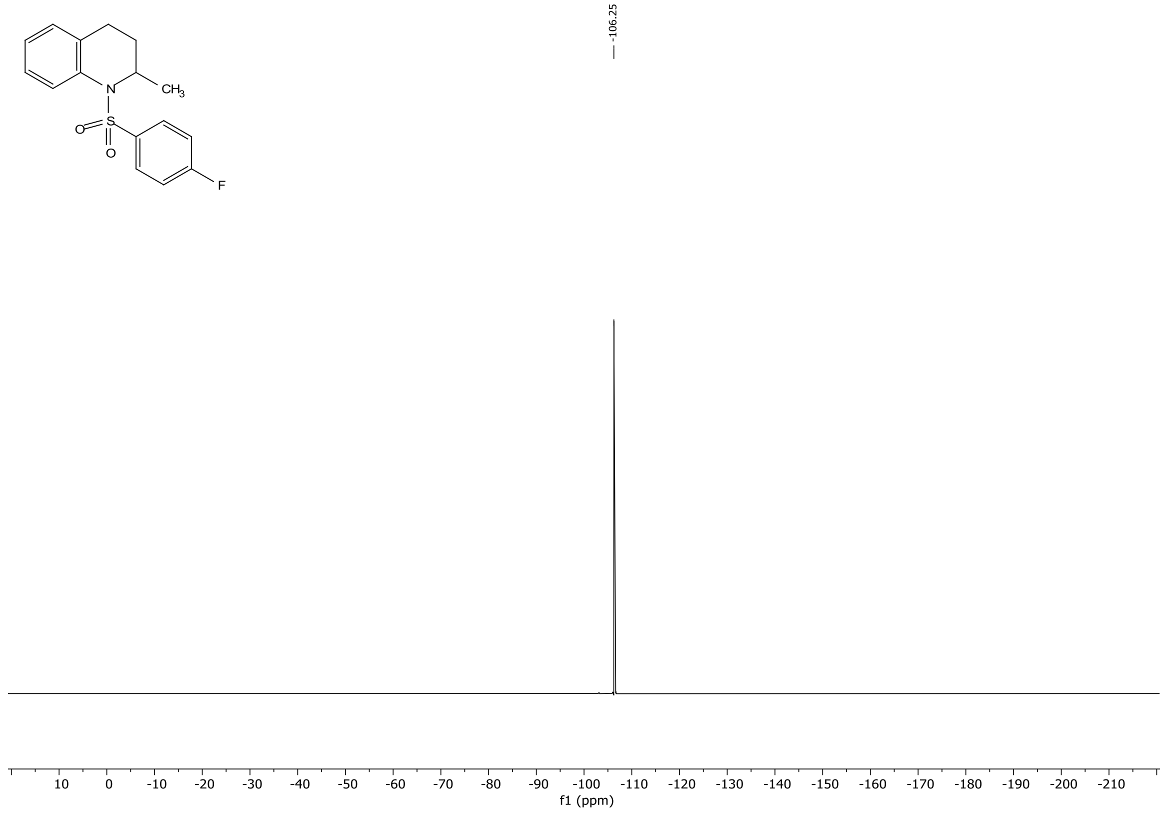

^{1}H NMR spectrum (400 MHz, CD_2Cl_2) of **3d’**

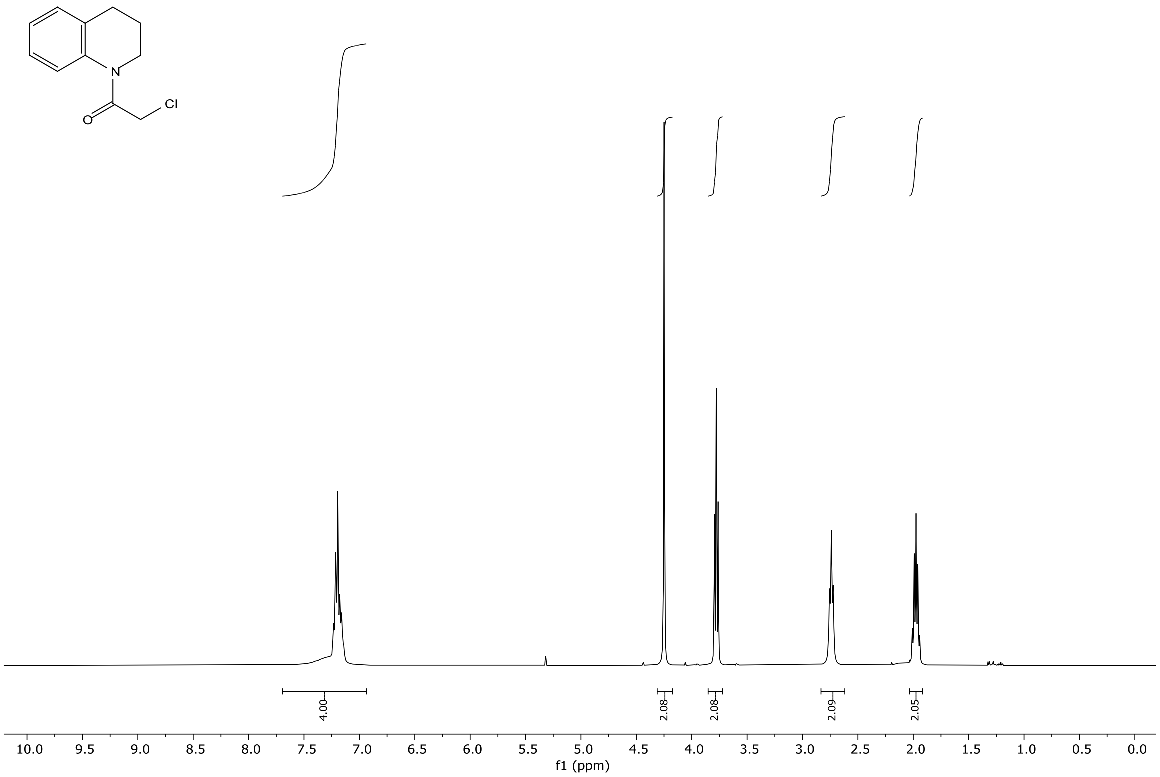

^{1}H NMR spectrum (400 MHz, CD_2Cl_2) of **3d’**

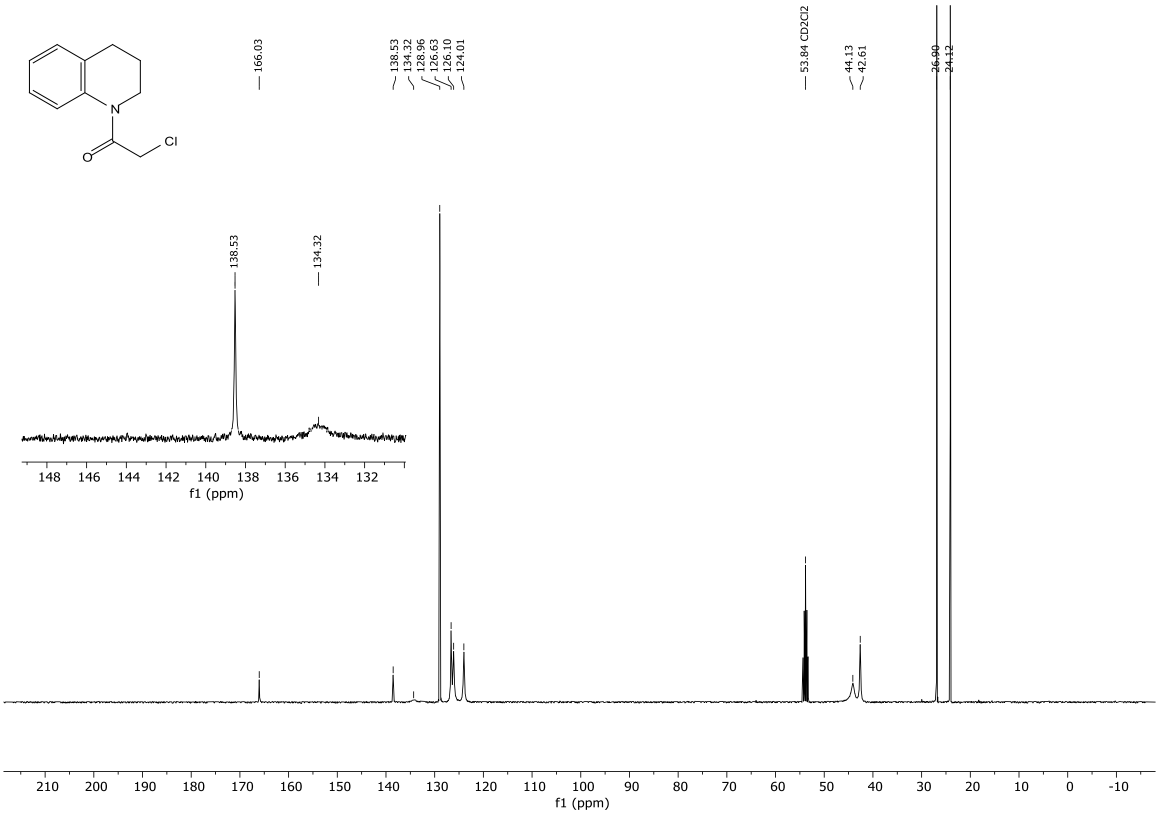

^{1}H NMR spectrum (400 MHz, CD_2Cl_2) of **3d**

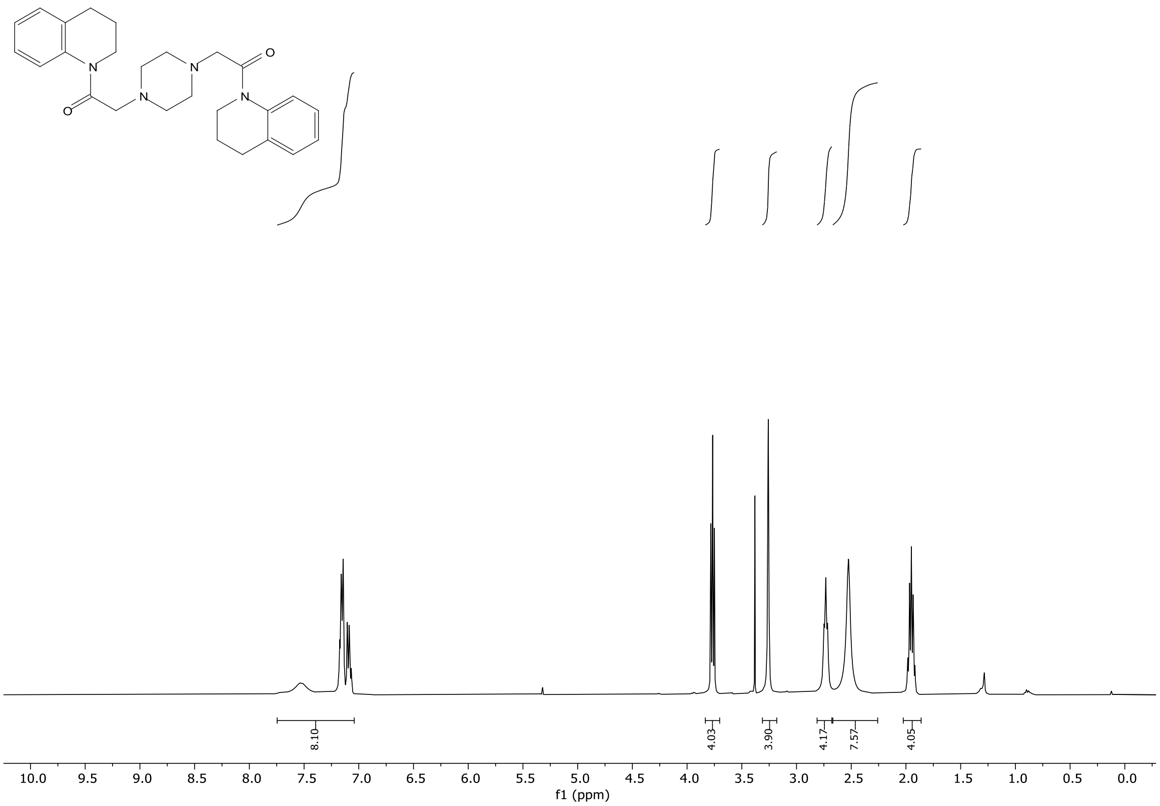

^{13}C NMR spectrum (400 MHz, CD_2Cl_2) of **3d**

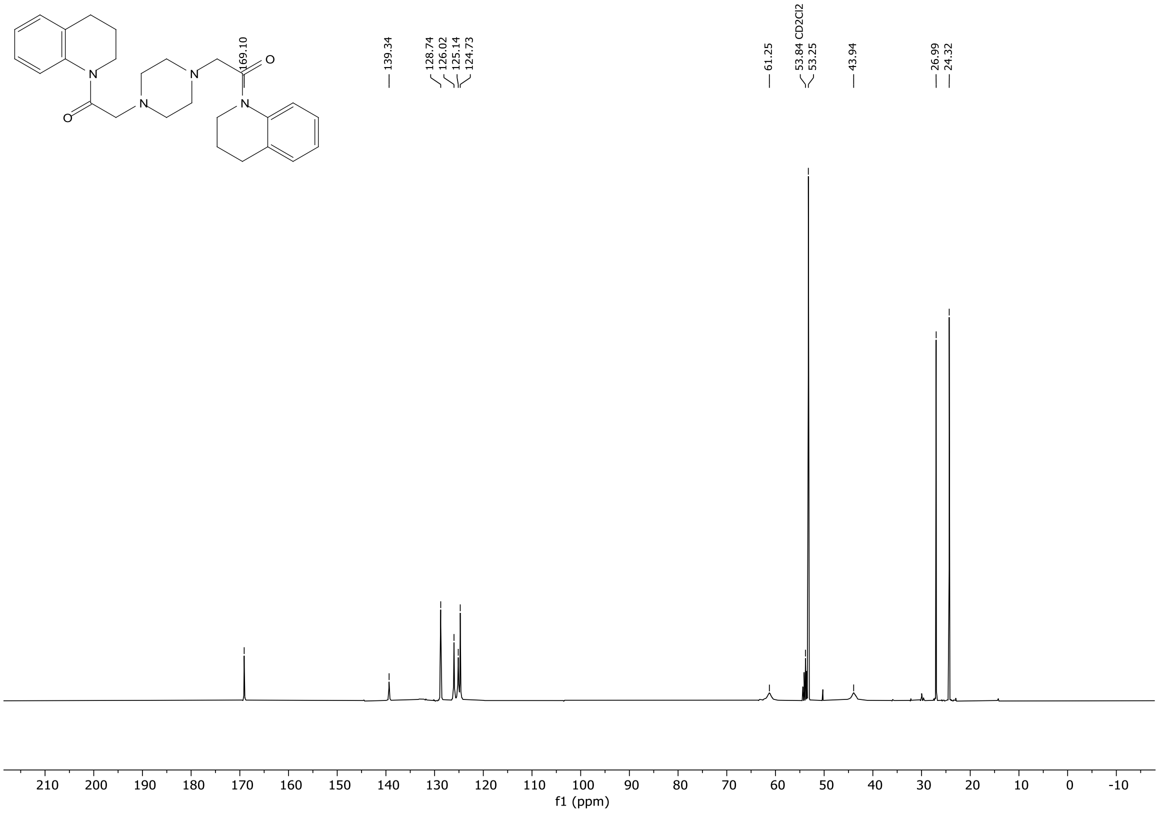

^{1}H NMR spectrum (400 MHz, CD_2Cl_2) of **3e''**

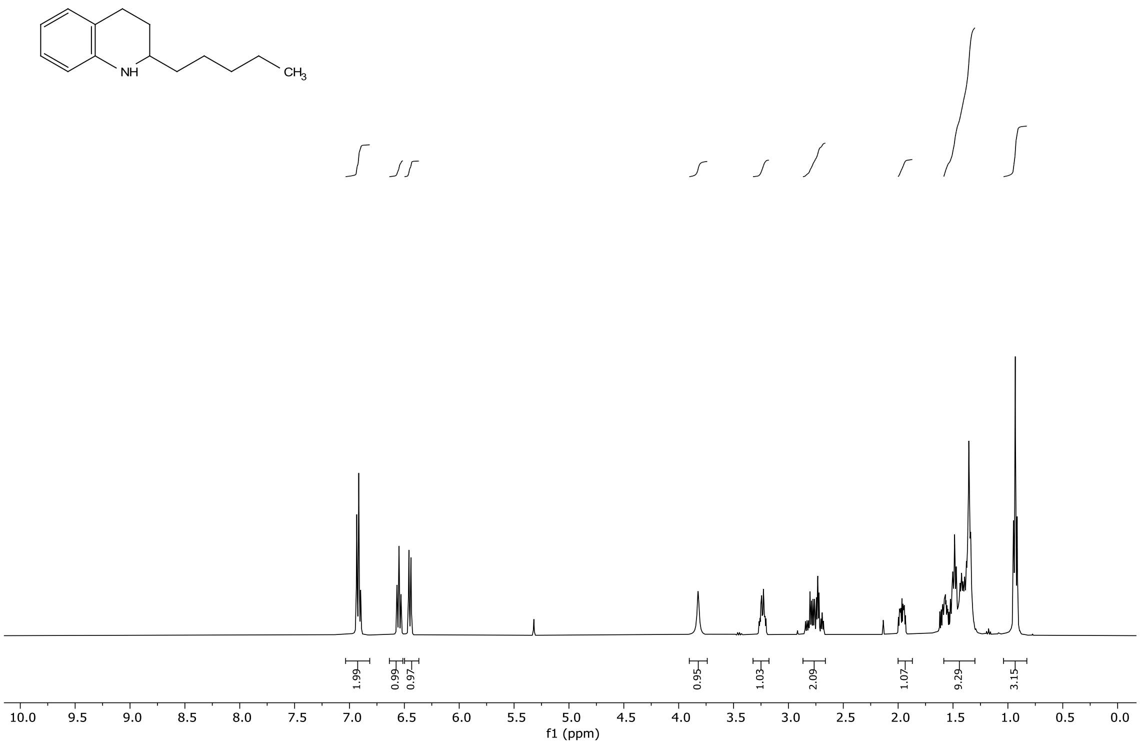

^{13}C NMR spectrum (400 MHz, CD_2Cl_2) of **3e''**

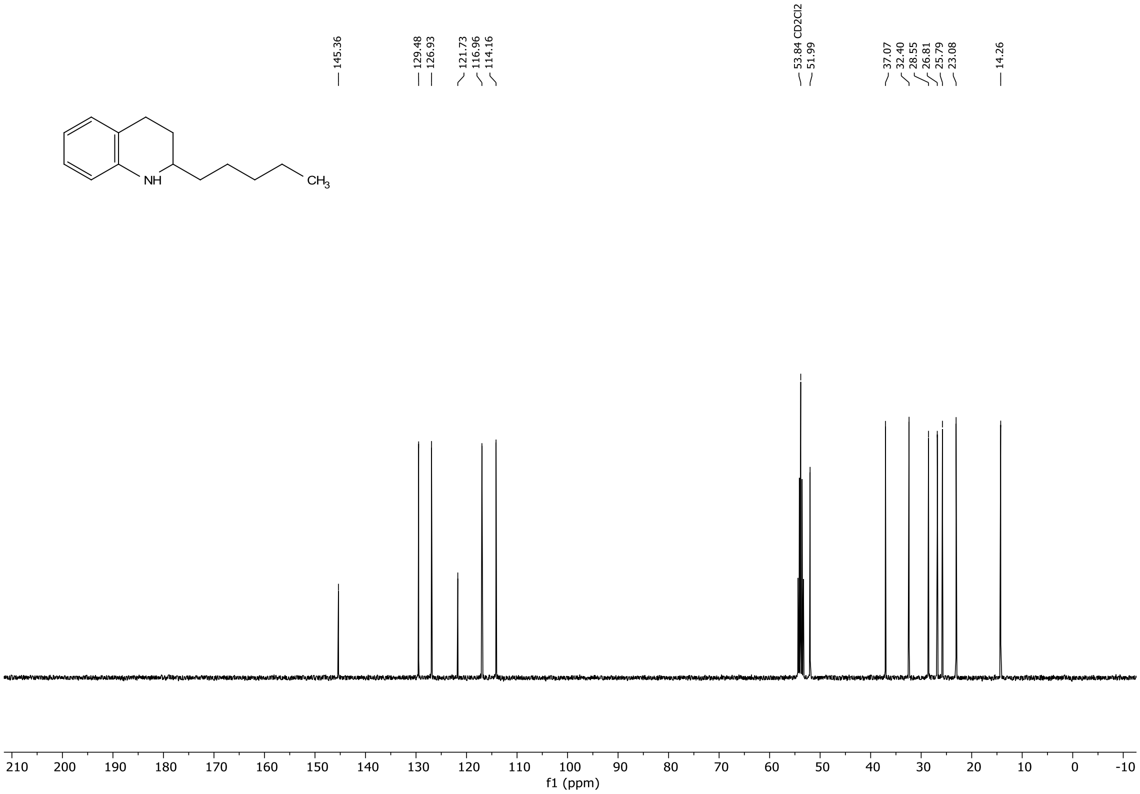

^{1}H NMR spectrum (400 MHz, CD_2Cl_2) of **3e**

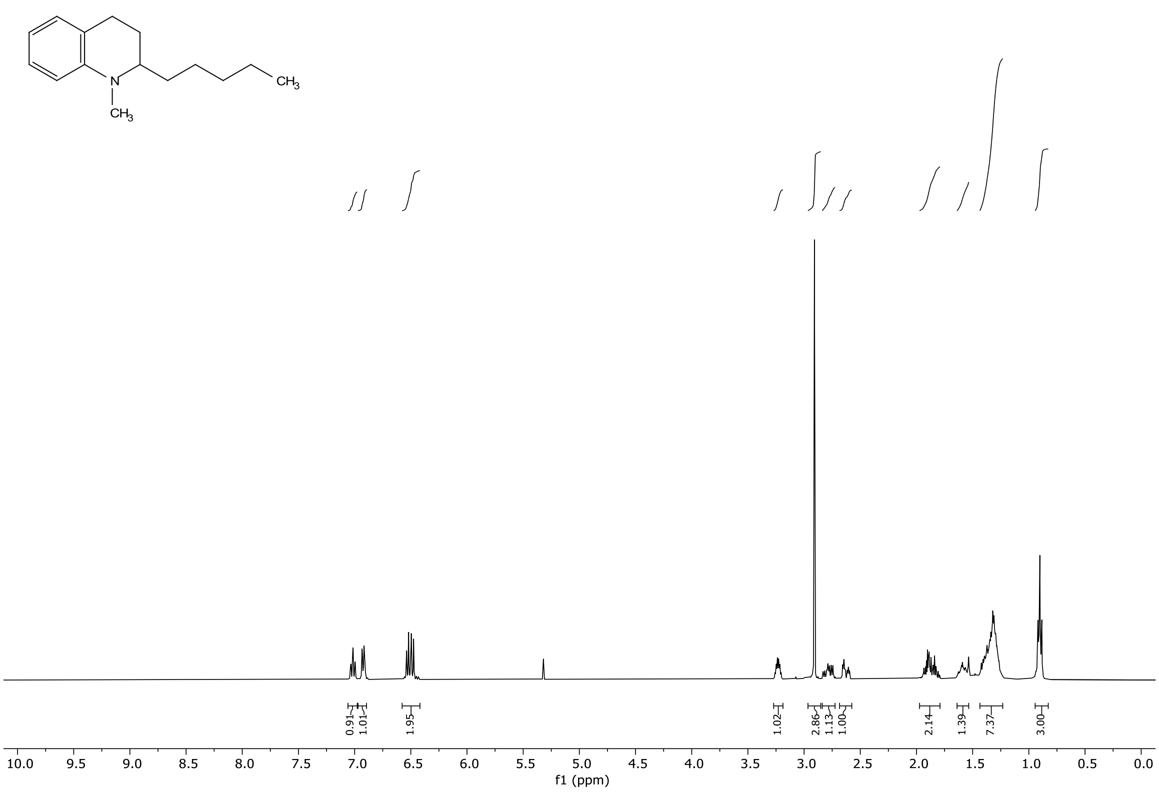

^{13}C NMR spectrum (400 MHz, CD_2Cl_2) of **3e**

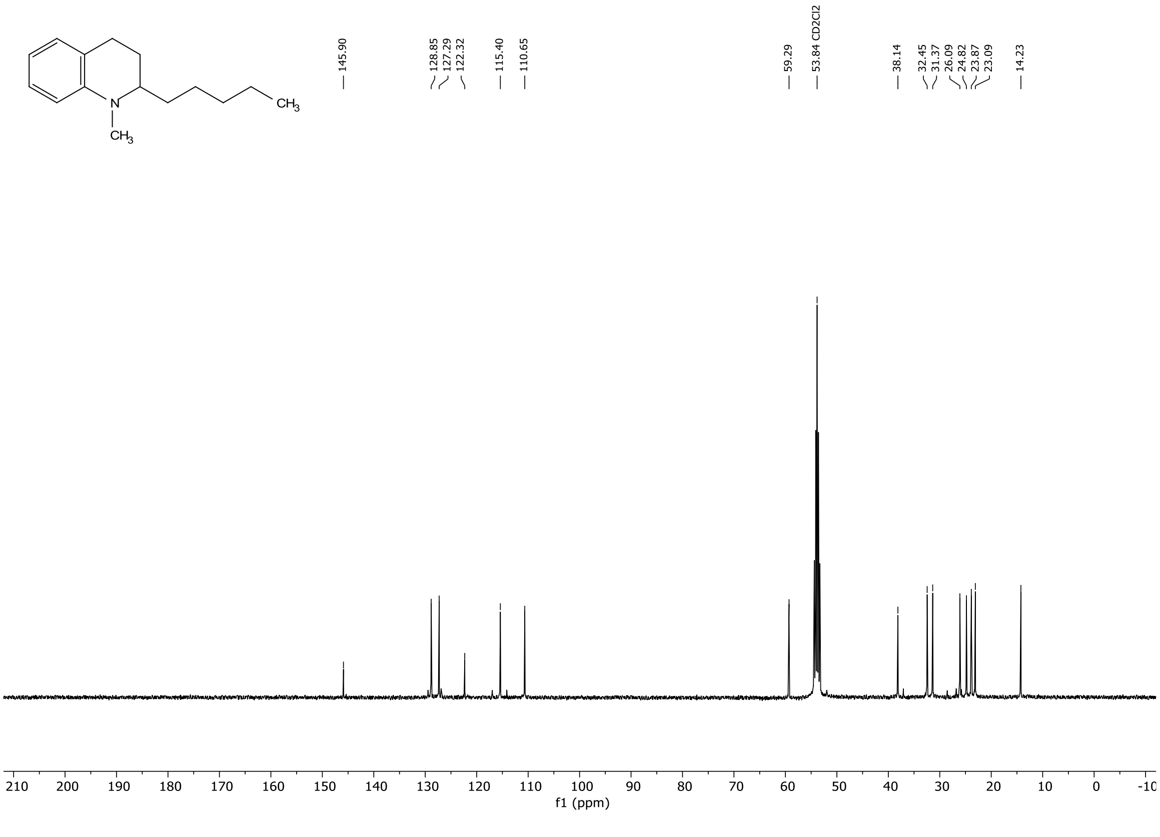

12 References

[1] a) T. Wirtanen, E. Rodrigo, S. R. Waldvogel, *Adv. Synth. Catal.* **2020**, *362*, 2088–2101; b) B. Gleede, M. Selt, C. Gütz, A. Stenglein, S. R. Waldvogel, *Org. Process Res. Dev.* **2020**, *24*, 1916–1926; c) M. Dörr, J. L. Röckl, J. Rein, D. Schollmeyer, S. R. Waldvogel, *Chem. Eur. J.* **2020**, *26*, 10195–10198.

[2] M.-R. Liang, X. Du, J. Lin, N. Rong, X. Zhan, X. Mao, H. Zhuang, T. Niu, Q. Yin, *J. Am. Chem. Soc.* **2025**, *147*, 4239–4248.

[3] R. Kleinmans, S. Dutta, K. Ozols, H. Shao, F. Schäfer, R. E. Thielemann, H. T. Chan, C. G. Daniliuc, K. N. Houk, F. Glorius, *J. Am. Chem. Soc.* **2023**, *145*, 12324–12332.

[4] L. Zhang, J. Yan, D. Ahmadli, Z. Wang, T. Ritter, *J. Am. Chem. Soc.* **2023**, *145*, 20182–20188.

[5] P. Onnuch, K. Ramagonolla, R. Y. Liu, *Science* **2024**, *383*, 1019–1024.

[6] F. Liu, X. Yan, F. Cai, W. Hou, J. Dong, S.-F. Yin, G. Huang, T. Chen, M. Szostak, Y. Zhou, *Nat. Commun.* **2025**, *16*, 1294.

[7] S. W. Krabbe, V. S. Chan, T. S. Franczyk, S. Shekhar, J. G. Napolitano, C. A. Presto, J. A. Simanis, *J. Org. Chem.* **2016**, *81*, 10688–10697.

[8] J. Qin, F. Chen, Z. Ding, Y.-M. He, L. Xu, Q.-H. Fan, *Org. Lett.* **2011**, *13*, 6568–6571.

[9] F. Schäfer, L. Lückemeier, F. Glorius, *Chem. Sci.* **2024**, *15*, 14548–14555.

[10] Y.-N. Duan, X. Du, Z. Cui, Y. Zeng, Y. Liu, T. Yang, J. Wen, X. Zhang, *J. Am. Chem. Soc.* **2019**, *141*, 20424–20433.

[11] T. Wang, L.-G. Zhuo, Z. Li, F. Chen, Z. Ding, Y. He, Q.-H. Fan, J. Xiang, Z.-X. Yu, A. S. C. Chan, *J. Am. Chem. Soc.* **2011**, *133*, 9878–9891.

[12] A. Karakulina, A. Gopakumar, İ. Akçok, B. L. Roulier, T. LaGrange, S. A. Katsyuba, S. Das, P. J. Dyson, *Angew. Chem. Int. Ed.* **2016**, *55*, 292–296.

[13] B. Sun, D. Carnevale, G. Süss-Fink, *J. Organomet. Chem.* **2016**, *821*, 197–205.

[14] F. Chen, A.-E. Surkus, L. He, M.-M. Pohl, J. Radnik, C. Topf, K. Junge, M. Beller, *J. Am. Chem. Soc.* **2015**, *137*, 11718–11724.

[15] I. Sorribes, L. Liu, A. Doménech-Carbó, A. Corma, *ACS Catal.* **2018**, *8*, 4545–4557.

[16] Y. Zhang, J. Zhu, Y.-T. Xia, X.-T. Sun, L. Wu, *Adv. Synth. Catal.* **2016**, *358*, 3039–3045.

[17] A. M. Maj, I. Suisse, C. Hardouin, F. Agbossou-Niedercorn, *Tetrahedron* **2013**, *69*, 9322–9328.

[18] Y.-G. Ji, K. Wei, T. Liu, L. Wu, W.-H. Zhang, *Adv. Synth. Catal.* **2017**, *359*, 933–940.

[19] S. Li, W. Meng, H. Du, *Org. Lett.* **2017**, *19*, 2604–2606.

[20] Z. Luo, J. Yang, Z. Yao, J. Yang, L. Xu, Q. Shi, *Adv. Synth. Catal.* **2023**, *365*, 3527–3534.

[21] S. C. Cosgrove, J. M. C. Plane, S. P. Marsden, *Chem. Sci.* **2018**, *9*, 6647–6652.

[22] A. Lator, S. Gaillard, A. Poater, J.-L. Renaud, *Org. Lett.* **2018**, *20*, 5985–5990.

[23] Q.-Z. Zheng, K. Cheng, X.-M. Zhang, K. Liu, Q.-C. Jiao, H.-L. Zhu, *Eur. J. Med. Chem.* **2010**, *45*, 3207–3212.

[24] A. Sau, D. Mahapatra, A. Maji, S. Dey, A. Roy, S. Kundu, *Org. Lett.* **2024**, *26*, 4486–4491.

[25] Y.-F. Liang, X.-F. Zhou, S.-Y. Tang, Y.-B. Huang, Y.-S. Feng, H.-J. Xu, *RSC Adv.* **2013**, *3*, 7739.

[26] Z.-J. Wang, H.-F. Zhou, T.-L. Wang, Y.-M. He, Q.-H. Fan, *Green Chem.* **2009**, *11*, 767.

[27] N. Shida, Y. Shimizu, A. Yonezawa, J. Harada, Y. Furutani, Y. Muto, R. Kurihara, J. N. Kondo, E. Sato, K. Mitsudo et al., *J. Am. Chem. Soc.* **2024**, *146*, 30212–30221.

[28] A. Fukazawa, J. Minoshima, K. Tanaka, Y. Hashimoto, Y. Kobori, Y. Sato, M. Atobe, *ACS Sustainable Chem. Eng.* **2019**, *7*, 11050–11055.

[29] R. Mondal, L. Galmidi, A. Tzaguy, T. Sason, M. Feller, M. A. Iron, L. Avram, R. Neumann, S. Gnaim, *J. Am. Chem. Soc.* **2025**, *147*, 41272–41283.

[30] a) A. Valiente, P. Martínez-Pardo, G. Kaur, M. J. Johansson, B. Martín-Matute, *ChemSusChem* **2022**, *15*, e202102221; b) W. Zheng, Y. Liu, F. Liu, Y. Wang, N. Ren, S. You, *Water research* **2022**, *223*, 118994.
[31] D. Chanda, J. Hnát, A. S. Dobrota, I. A. Pašti, M. Paidar, K. Bouzek, *Physical chemistry chemical physics : PCCP* **2015**, *17*, 26864–26874.
[32] S. A. Ross, G. Lowe, *Tetrahedron Lett.* **2000**, *41*, 3225–3227.
